# Differential Impacts on Bacterial Composition and Abundance in Rhizosphere Compartments between Al-Tolerant and Al-Sensitive Soybean Genotypes in Acidic Soil

**DOI:** 10.4014/jmb.2003.03018

**Published:** 2020-05-20

**Authors:** Zhong-Ling Wen, Min-Kai Yang, Aliya Fazal, Yong-Hui Liao, Lin-Run Cheng, Xiao-Mei Hua, Dong-Qing Hu, Ji-Sen Shi, Rong-Wu Yang, Gui-Hua Lu, Jin-Liang Qi, Zhi Hong, Qiu-Ping Qian, Yong-Hua Yang

**Affiliations:** 1 Institute for Plant Molecular Biology, State Key Laboratory of Pharmaceutical Biotechnology, School of Life Sciences, Nanjing University, Nanjing 210023, P.R. China; 2 Jinhua Academy of Agricultural Sciences, Jinhua 321017, P.R. China; 3 Research Center for Soil Pollution Prevention and Control, Nanjing Institute of Environmental Sciences, MEE, Nanjing 210042, P.R. China; 4 Co-Innovation Center for Sustainable Forestry in Southern China, Nanjing Forestry University, Nanjing 210037, P.R. China

**Keywords:** Soybean genotypes, rhizosphere compartments, bacterial communities, nitrogen fixation, aluminum tolerance

## Abstract

In this study, two soybean genotypes, *i.e*., aluminum-tolerant Baxi 10 (BX10) and aluminum- sensitive Bendi 2 (BD2), were used as plant materials and acidic red soil was used as growth medium. The soil layers from the inside to the outside of the root are: rhizospheric soil after washing (WRH), rhizospheric soil after brushing (BRH) and rhizospheric soil at two sides (SRH), respectively. The rhizosphere bacterial communities were analyzed by high-throughput sequencing of V4 hypervariable regions of 16S rRNA gene amplicons via Illumina MiSeq. The results of alpha diversity analysis showed that the BRH and SRH of BX10 were significantly lower in community richness than that of BD2, while the WRH exhibited no significant difference between BX10 and BD2. Among the three sampling compartments of the same soybean genotype, WRH had the lowest community richness and diversity while showing the highest coverage. Beta diversity analysis results displayed no significant difference for any compartment between the two genotypes, or among the three different sampling compartments for any same soybean genotype. However, the relative abundance of major bacterial taxa, specifically nitrogen-fixing and/or aluminum-tolerant bacteria, was significantly different in the compartments of the BRH and/or SRH at phylum and genus levels, indicating genotype-dependent variations in rhizosphere bacterial communities. Strikingly, as compared with BRH and SRH, the WRH within the same genotype (BX10 or BD2) always had an enrichment effect on rhizosphere bacteria associated with nitrogen fixation.

## Introduction

Acidity in soil affects about 30% of the world’s total land area and 50% of the world’s potential arable land [1]. In South China, acidic red soil is a typical soil type with low pH value, low phosphorus content and high aluminum toxicity, which are directly related to restrictive crop growth and yield [2-4]. In order to adapt to the stressful conditions, plants can release root exudates such as organic acids, which can chelate toxic Al (aluminum) and mobilize P (phosphorus) [5-7]. Soybean is an important protein and oil source in the world and can also be utilized as an excellent rotation and intercropping crop [8]. Previous studies have shown that under severe P stress, Al-tolerant soybean Baxi 10 (BX10) has a greater citrate efflux rate and nearly 2 times higher yield compared to Al- sensitive soybean Bendi 2 (BD2) [9]. Moreover, the rhizosphere soil of BX10 exhibited changes in the ratio of gram-negative/gram-positive bacteria compared with BD2 [10].

Each plant species has its own specific rhizospheric bacterial community as different rhizosphere bacteria respond differently to different compounds of root exudates [11, 12]. Previous studies have reported that plants can strongly influence the composition, structure, and activity of rhizosphere microbiota, especially with respect to the active populations [13-18]. Moreover, rhizosphere microorganisms such as bacteria, are closely related to plant health and growth [13,19-21], thus leading to a complex binary relationship between plants and their root- associated microorganisms. For example, experiments conducted by Li *et al*. showed that there are differences in the diversity and abundance of functional *nifH* gene (coding for nitrogenase iron protein) of the rhizosphere bacteria community at different growth stages between BX10 and BD2 [10, 22].

In this study, we aimed to elucidate the impacts of Al-tolerant (BX10) and Al-sensitive (BD2) soybean genotypes on the composition and abundance of rhizosphere bacterial communities at the flowering stage, among three different sampling compartments in acidic soil, via Illumina MiSeq sequencing platform.

## Materials and Methods

### Plant Materials, Sampling Methods and DNA Extraction

In this study, two soybean genotypes(*Glycine max* (L.) Merr.), BX10 (Baxi 10, aluminum (Al)-tolerance type) and BD2 (Bendi 2, Al-sensitive type), were selected as plant materials. Soybeans were planted in a rhizobox (rhizosphere box, 200 mm in length, 150 mm in width and 200 mm in depth) which was constructed with PVC material and divided into five parts by using a frame covered with nylon film, in order to prevent the root and root exudates from entering different soil compartments, but not water and nutrition [23, 24]. Finely sieved acidic red soil (pH 4.43) collected from the Ecological Experiment Station of Red Soil (28.208 N, 116.937 E) of the Chinese Academy of Sciences, Yingtan, Jiangxi Province, China [22], was evenly packed into five parts of the rhizobox while soybean was allowed to grow in the middle part.

Sampling at flowering stage was performed as described previously by Li *et al*. with some modifications [25, 26]. The soil in the compartments on both sides of the rhizobox (rhizosphere soils between two partitions in rhizobox) was named SRH (rhizospheric soil at two sides) [24]. BRH (rhizospheric soil after brushing) samples were collected by brushing off the soil tightly adhering to the root surface, and then the WRH (rhizospheric soil after washing) samples were collected by centrifugation at 4,000 ×*g* for 10 min after being washed with phosphate- buffered saline (PBS). Finally, all the samples were stored at -80oC prior to DNA extraction.

Metagenomic DNA of every biological replicate was extracted from approximately 0.30 g of soil by using the PowerSoil DNA Isolation Kit (MoBio Laboratories Inc., USA). The experimental method followed the instructions with minor modifications [27, 28]. After extraction, the quality of DNA samples was assessed on 1% agarose gel and quantified by using a Qubit Fluorometer (Qubit 2.0, Invitrogen, USA) to minimize the variability in surveys of bacterial communities [29].

### 16S rDNA Amplicon Sequencing and Analysis

We used an improved dual-index high-throughput sequencing with paired-end 250nt and amplicons of approximately 290 bp encompassing the V4 hypervariable region of the 16S rDNA [30] using the following primers: forward primer 515F (5’-GTGCCAGCMGCCGCGGTAA-3’) and reverse primer 806R (5’-GGACTA CHVGGGTWTCTAAT-3’) [31]. The concentration of each qualified metagenomic DNA was tested and ensured to be more than 0.4 ng/μl [29]. PCR amplification, product purification, library quality determination and high- throughput sequencing of the qualified libraries on the Illumina MiSeq platform (Illumina, USA) with MiSeq Reagent Kit were conducted by BGI Tech Solutions Co., Ltd. (China). A total of 18 sequencing clean data has been submitted to the Sequence Read Archive (SRA) and the SRA accession number is PRJNA574806.

In order to characterize the bacterial community composition and structure in the SRH, BRH and WRH, high- throughput sequencing of 16S rDNA amplicons using V4 region on the Illumina MiSeq platform was performed. Then, the clean tags were clustered into Operational Taxonomic Units (OTUs) with a 97% similarity by using UPARSE software (v7.0.1090) [32] and the chimeras were filtered out by using UCHIME(v4.2.40) and 16S rDNA was screened for chimeras by mapping to a gold database (v20110519) [33]. The sub-sampling of OTU was performed using the software R (v3.1.3) in I-Sanger (http://www.i-sanger.com) according to the minimum sample number sequence. A total of 1,736,758 qualified paired-end reads with an average count of 96,486 (range: 80,603–114,407) per sample were obtained from 18 samples (Table S1). The high quality paired-end reads were then connected to tags based on 250 bp overlaps and a total of 1,214,537 tags were obtained (Table S1). After the removal of chimeras, clean tags were clustered into OTUs and a total of 35,651 OTUs were obtained (Table S1). The detailed information on OTUs with/without sub-sampling is summarized in Table S2 (Table S1). Then, the alpha and beta diversity analyses were conducted based on OTUs and species annotation results.

A rank-abundance curve was used to explain species abundance and evenness, and a Venn diagram was used to count the number of common and unique OTUs in multiple samples with 97% similarity level. Pan species were the sum of all species contained in all samples while core species were the number of common species in all the samples.

### Alpha Diversity, Beta Diversity, Functional Prediction and Analysis, and Statistical Analysis

In order to analyze the complexity of species diversity in the environment, we used alpha diversity to reflect the community richness (Sobs, Chao, and Ace indices), community diversity (Shannon and Simpson indices) and community coverage (coverage index), while beta diversity analysis was calculated by using QIIME (v 1.8.0). We then evaluated differences of samples in species complexity by using different distance matrices [34, 35]. In this study, the results of three representative indices (Chao, Shannon, and Good’s coverage) were shown to reflect the number of species, the uniformity of individual distribution in community and the coverage of all species, respectively.

Principal Component Analysis (PCA), a technique for analyzing and simplifying data sets using species abundances, was drawn by ‘prcomp’ in software R (v3.1.3). Principal Co-ordinates Analysis (PCoA) is a non- constrained data dimensionality reduction analysis method, which can be used to find similarities or differences in sample community composition and is based on selected distance matrix (Bray-Curtis, weighted-UniFrac and unweighted-UniFrac). Both of these can identify potential principal components which can affect the diversity of sample community composition by dimensionality reduction. Unweighted Unifrac is affected by the number of rare OTUs in samples while Bray-Curtis and weighted Unifrac are based on the calculation of microbial evenness (the relative abundance of OTU), so the rare OTUs with low relative abundance have little impact on the results. Meanwhile, UniFrac distance matrices (including weighted Unifrac and unweighted Unifrac) are based on the comparison with phylogenetic tree, while other distance matrices like Bray-Curtis and Euclidean are based on the comparison without a phylogenetic tree. The PCoA was drawn by software R (v3.1.3) using vegan package. Non- metric multidimensional scaling analysis (NMDS) is a data analysis method that simplifies the object of study (sample or variable), and NMDS can retain the original relationship between objects. NMDS involved the use of QIIME (v 1.8.0) to calculate the distance matrix and was also drawn by software R (v3.1.3) using vegan package. Partial Least Squares Discriminant Analysis (PLS-DA) effectively finds the influencing variables leading to the differences between groups by rotating the principal components. Unweighted Pair-group Method with Arithmetic Mean (UPGMA) is a clustering analysis method drawn by software R (v3.1.3) and was used to construct a tree structure to visualize the degree of difference in microbial evolution in different samples. Heatmaps aggregate species with high/low abundance and ours were drawn with the NMF package software R (v3.1.3). A ternary plot is an equilateral triangle used to compare three groups of samples and the proportion and relationship of different species in samples can be visually displayed and analyzed following methods by Bulgarelli *et al.*[36]. All these analyses were performed in platform I-Sanger (http://www.i-sanger.com). The COG function classification was also performed in platform I-Sanger and descriptive information and functional information of each COG can be parsed from eggNOG (evolutionary genealogy of genes: Non-supervised Orthologous Groups, http://eggnog.embl.de/) database while the abundance of each functional category can be calculated according to OTU abundance.

For statistical analysis, one-way ANOVA was used to evaluate the significance of samples in different alpha diversity indices. The analysis of similarities (ANOSIM) and PERMANOVA analysis (Adonis) were performed using vegan package of software R (v3.1.3) based on different distance metrics (Bray-Curtis, weighted-UniFrac and unweighted-UniFrac) by using databases Silva128/16s (SILVA database, http://www.arb-silva.de) [37].

## Results

### Basic Data of Samples

**Fig. 1:**
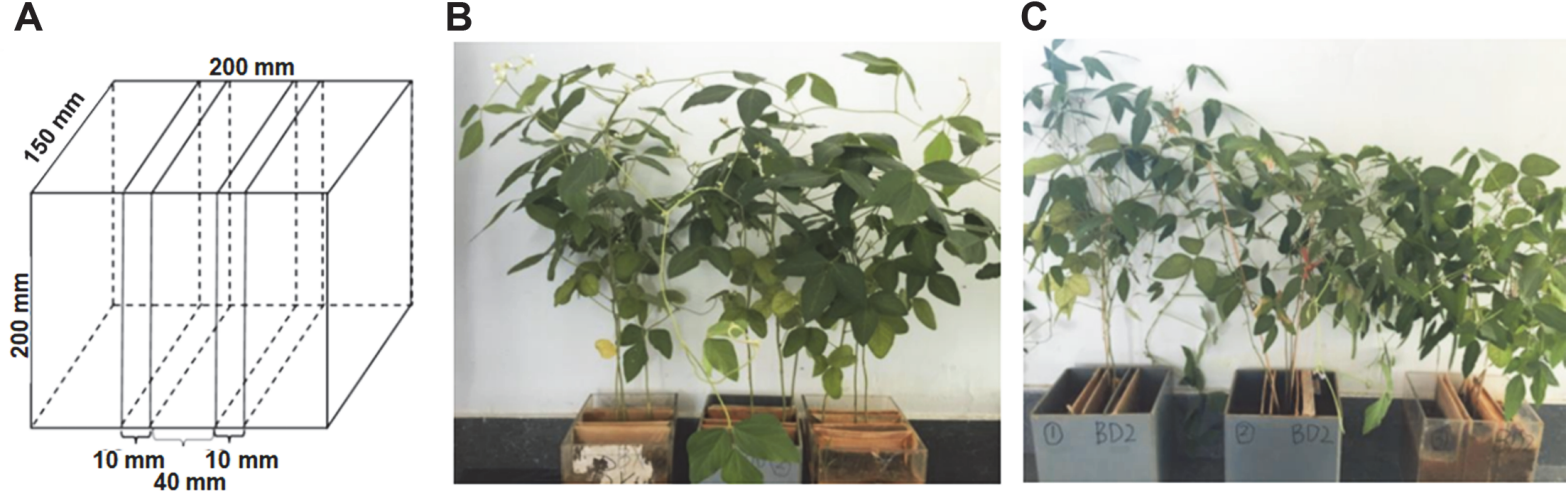
Rhizobox diagram (A) and the rhizoboxes for soybean cultivation of BX10 (B) and BD2 (C).

The rhizobox used in this study was designed as previously reported with minor modifications (Fig. 1) [10, 23]. The shape of the Rank-Abundance curve was gentle, and the curve also had a wide range on the horizontal axis. It shows that the abundance and evenness of 18 samples were relatively higher, and the trend of each sample was similar, which proves that the quality of samples and the depth and quality of sequencing were also relatively higher, indicating that the OTU coverage of the samples included sufficient detectable species in bacterial communities (Fig. S1). As shown in the Venn diagram, the OTU numbers of each sample were similar while the total number of shared OTUs of SRH, BRH and WRH was 1,227 (Fig. S2). According to the size of each list, the BRH samples have the most OTU numbers while the WRH samples have the fewest OTU numbers. Furthermore, as shown in Pan analysis and Core analysis, the number of core species (*i.e*., the number of species shared by all samples) is 331, while the total number of species contained by all samples is 5,829 (Fig. S3).

### Comparative Analysis of the Alpha Diversity of Rhizosphere Bacterial Communities between BX10 and BD2

**Fig. 2:**
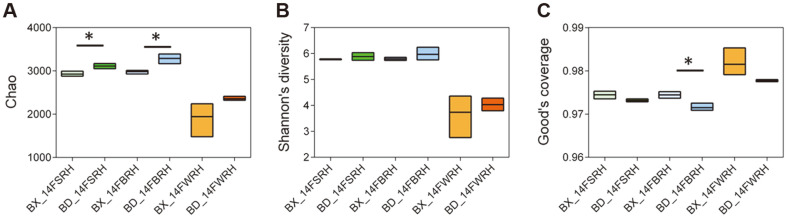
The boxplot of alpha diversity of Chao index(A), Shannon index (B) and Good’s coverage (C). SD represents standard deviation. Values were mean ± SD (*n* = 3). BX and BD represent the Al-tolerance soybean BX10 and Al- sensitive soybean BD2, respectively. F represents the flowering stages. SRH, BRH and WRH represent rhizospheric soil at two sides, rhizospheric soil after brushing, and rhizospheric soil after washing, respectively. The significance test method was performed using one-way ANOVA. The value in bold indicate the significant difference (*p* < 0.05) between the BX10 and BD2 groups by the tests.

The rarefaction curves of alpha diversity indicated that the amount of sequencing data was enough to reflect the vast majority, and the community coverage was good. We therefore conducted comparative analysis of the alpha diversity through three different indices (Chao, Shannon, and Good’s coverage). As shown in Fig. 2, for different sampling compartments of the same genotype (BX10 or BD2), the WRH has the lowest community richness and diversity, but meanwhile exhibited the highest coverage (Fig. 2). From the intergroup t-test of alpha diversity, we observed that, for WRH, there was no significant difference between the two genotypes in the six indices. However, the community richness in BRH was significantly lower in BX10 than that in BD2 (Fig. 2A), while community coverage in BX10 was higher than that in BD2 (Fig. 2C). Furthermore, SRH sample also showed significantly lower community richness in BX10 than that in BD2 (Fig. 2A).

### Comparative Analysis of the Beta Diversity of Rhizosphere Bacterial Communities between BX10 and BD2

In order to study the similarities and differences in species composition and structure of different samples, a distance matrices and hierarchical clustering trees were constructed by using UPGMA. Cluster trees of beta diversity based on the Bray-Curtis distance (Fig. S4A), weighted-Unifrac distance (Fig. S4B) and unweighted- Unifrac distance (Fig. S4C) showed that the samples from the same genotype of soybean and the same sampling compartment were clustered into one group. Furthermore, similar hierarchical clustering trees were obtained by three distance matrices above.

As shown in Fig. S5, samples from the same genotype or same sampling compartment (layer of soil) were clustered into one group via heat maps, which was consistent with the result of cluster trees shown above. Among different sampling compartments (layer of soil), high similarity of bacterial communities existed between BRH and WRH (Fig. S5), which has the same trend as the alpha diversity displaying richness of bacterial community (Fig. 2A).

**Fig. 3:**
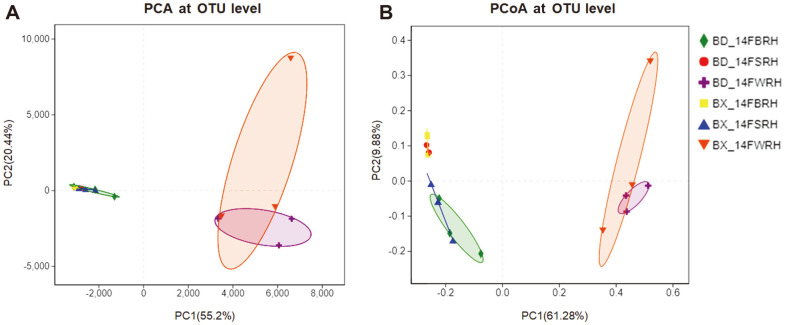
PCA based on OTU abundance of bacterial communities (A). PCoA based on Bray-Curtis distance of the rhizosphere bacterial communities between BX10 and BD2 (B). Treatment’s details were as in Fig. 2.

Next, the PCA of OTU was done to examine the differences in the OTU composition between the SRH, BRH and WRH samples of BX10 and BD2 at the flowering stage (Fig. 3A). As shown in Fig. 3A, the samples from the same compartments (SRH, BRH or WRH) were clustered into one group, and there was no significant distinction among different soybean genotypes. Similar results were obtained from a PCoA chart based on Bray-Curtis (Fig. 3B). Similarly, the PCoA based on weighted-Unifrac distance and unweighted-Unifrac distance was consistent with the results based on Bray-Curtis (Fig. S6). As shown in Fig. S6, the rhizosphere bacterial communities from different compartments of BX10 were not distinct from those of BD2. The distance between BRH and SRH was close and indicated that high similarity of species composition existed between these two compartments. Furthermore, WRH was far away from BRH and SRH on the figures (Figs. 3 and S6).

We next performed ANOSIM and Adonis analysis of bacterial communities based on different distance metrics. The results of ANOSIM and Adonis displayed no significant difference between the beta diversity of rhizosphere bacterial communities of BX10 and BD2 (*p*-value > 0.05) (Table 1). Moreover, the ANOSIM and Adonis analysis of bacterial community structure between different sampling compartments based on the Bray- Curtis matrix indicated no significant difference between SRH and BRH, SRH and WRH, BRH and WRH (*p*- value > 0.05) (Table S3).

**Fig. 4:**
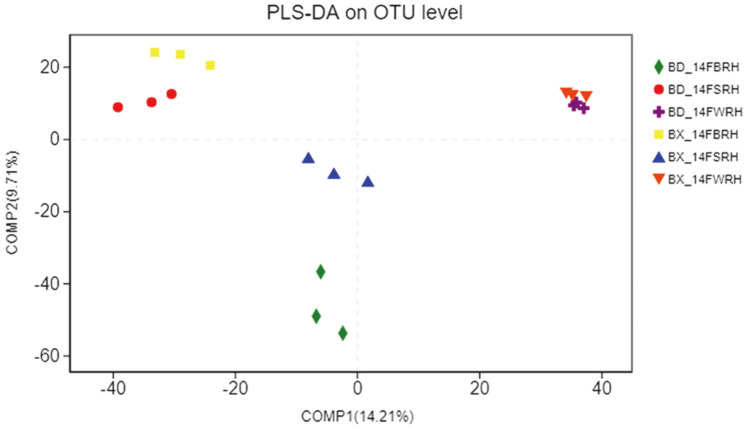
PLS-DA on OTU level of the rhizosphere bacterial communities between BX10 and BD2. Treatment’s details were as in Fig. 2.

As shown in Fig. S7, samples from the same sampling compartments clustered into one group while there also existed distinction in distance between different sampling compartments via NMDS. Similarly, samples were clustered by different sampling compartments and soybean genotypes via PLS-DA (Fig. 4).

### Comparison of the Composition of the Major Bacterial Taxa

The results of taxonomic analysis helped us identify the community structure and composition of different samples at different taxonomic levels (phylum, class, order, family, genus and species). In this way, the information of composition and abundance for each sample could be presented intuitively. The results of ternary plots were shown in Fig. S8. The relative abundance and proportional contribution of OTUs in three compartments indicated that *Psedumonas* and *Pseudarthrobacteroxydans*, which belong to phyla Proteobacteria and Actinobacteria, have the highest abundance, while *Candidatus* Nitrosotalea, which belong to phylum Tenericutes, has the lowest abundance in WRH compared to BRH and SRH.

The taxonomic composition distribution histogram of each sample was displayed at phylum, class, order, family, genus and species level (Fig. S9). Then we compared the eight major phyla in each sample. As shown in Table 2, the most abundant phylum was Acidobacteria followed by Proteobacteria, Chloroflexi, and Thaumarchaeotain in SRH and BRH. However, in WRH, the most abundant phylum was Proteobacteria followed by Actinobacteria and Firmicutes.

**Fig. 5:**
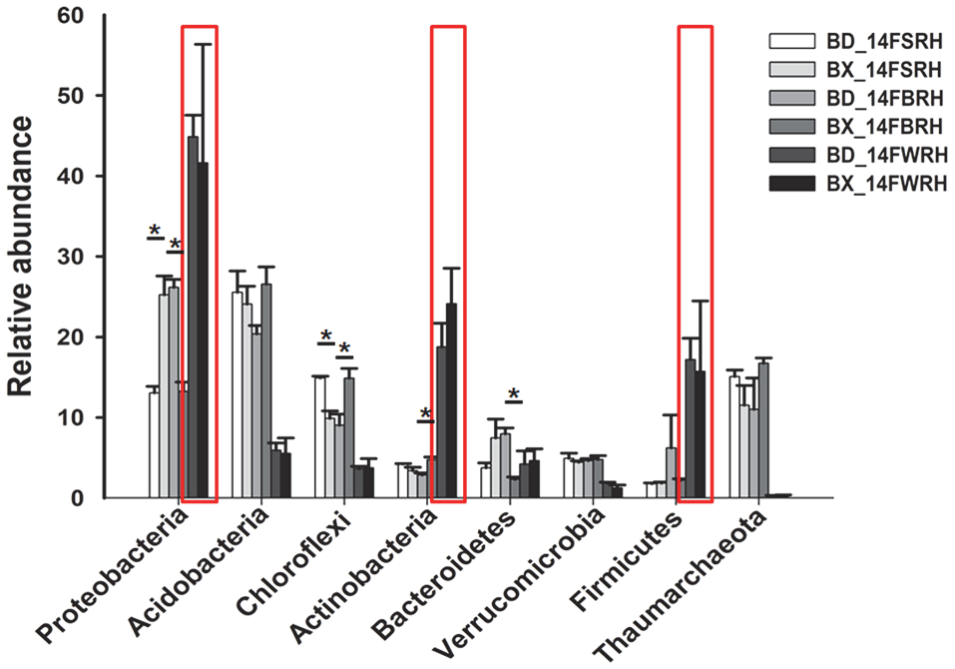
Relative abundances of eight major phyla in each sample. The relative abundances of phyla Proteobacteria, Actinobacteria and Firmicutes in WRH were significantly higher than that in SRH and BRH and were marked in red boxes. Asterisk (*p < 0.05) indicates significant difference according to Student’s *t*-test. Treatment’s details were as in Fig. 2.

As shown in Table 2, Figs. 5 and S9, for the same sampling compartment, the relative abundances of Proteobacteria in BX_14FSRH, Chloroflexi in BX_14FBRH and Actinobacteria in BX_14FBRH were significantly higher than that in BD2, meanwhile the Proteobacteria in BX_14FBRH, Chloroflexi in BX_14FSRH and Bacteroidetes in BX_14FBRH were significantly lower than that in BD2. Further analysis of the differences in bacteria composition on genus level indicated that BX_14FSRH was enriched for *Burkholderia* (belonging to phylum Proteobacteria) and depleted for *Ktedonobacter* (belonging to Chloroflexi). Similarly, BX_14FSRH was enriched for *Ktedonobacter* and *Acidothermus* (belonging to Actinobacteria), while depleted for*Burkholderia* and *Mucilaginibacter* (belonging to Bacteroidetes), as compared to the same sampling compartment in BD2. Furthermore, the species composition and abundance of WRH exhibited no significant different between BX10 and BD2. Moreover, the species composition and abundance of WRH were different from those of SRH and BRH at different taxonomic levels of the same soybean genotypes (BX10 or BD2). Comparing the community classification and abundance of samples at phylum level, the relative abundance of phyla Proteobacteria, Actinobacteria and Firmicutes in WRH were significantly higher than that in SRH and BRH (Fig. 3).

Next, we further compared the composition of the major bacterial taxa at the class, order or family levels (Fig. S9), especially the difference between WRH and the other two compartments. At class level, the relative abundances of Bacilli, Gammaproteobacteria and Actinobacteria in the WRH were significantly higher than that in SRH and BRH. At order level, the relative abundances of Bacillales, Pseudomonadales and Micrococcales in the WRH were significantly higher than that in SRH and BRH. At family level, the relative abundances of Bacillacea, Paenibacillacea, Pseudomonadaceae and Micrococcaceae in the WRH were significantly higher than that in SRH and BRH. At genus level, the relative abundances of *Bacillus*, *Paenibacillus*, *Pseudomonas* and *Pseudarthrobacter* in the WRH were significantly higher than that in SRH and BRH. At species level, the relative abundances of *Paenibacillusodorifer*, *Paenibacilluscastaneae*, *Paenibacilluspectinilyticus*, *Pseudomonas brassicacearum* and *Pseudarthrobacteroxydans* in the WRH were significantly higher than that in SRH and BRH.

### Functional Prediction and Analysis of 16S rDNA

**Fig. 6:**
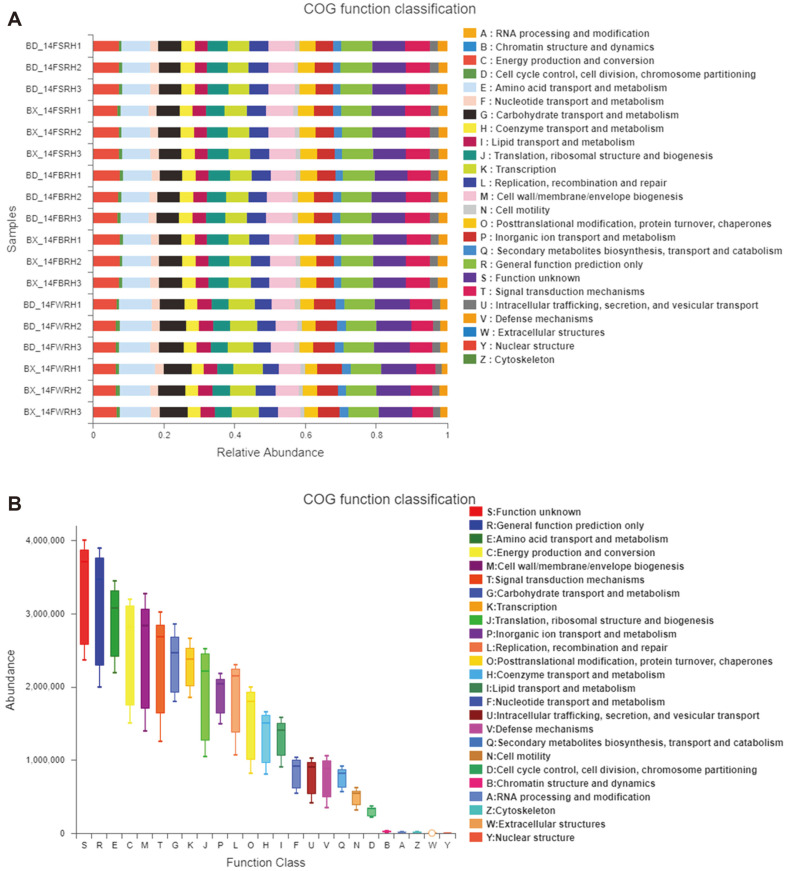
COG functional prediction. Treatment’s details were as in Fig. 2.

Functional prediction of 16S rDNA was conducted by standardizing OTU abundance using the software PICRUSt, and then obtaining COG family information corresponding to OTU by Greengene ID, and calculating the COG abundance of each sample. As shown in Fig. 6A, the composition and abundance of COG function classification of each sample was consistent with the 16S functional prediction. We therefore concluded the relative abundances of some main COG functional classes (Fig. 6B), and found that the functional OTUs related to amino acid transport and metabolism, cell wall/ membrane/ envelope biogenesis, signal transduction mechanism, energy production and conversion, carbohydrate transport and metabolism, transcription, inorganic ion transport and metabolism, replication, and recombination and repair were relatively higher in abundance (abundance > 2,000,000).

According to the relative abundance of COG functional classification, we found that five COGs, *i.e*., COG1348, COG5420, COG5554, COG5456 and COG4656, are directly related to nitrogen-fixation (Table S4). Then, the statistics were analyzed by using one-way ANOVA (Table 3). As shown in Table 3, the relative abundances of COG5554, COG5456 and COG4656 were significantly lower in BX_14FBRH than that in BD_14FBRH. Moreover, the relative abundance of COG5554 was significantly lower in BX_14FSRH than that in BD_14FSRH. The three COGs, i.e., COG5554, COG5456 and COG4656, were described as nitrogen fixation protein, nitrogen fixation protein FixH, and required for nitrogen fixation, which may be part of a membrane complex functioning as an intermediate in the electron transport to nitrogenase, respectively. Furthermore, when comparing these five COG function classifications in the same soybean genotype (BD2 or BX10) among the three sampling compartments, the COG5554 was higher in WRH than in SRH and BRH, while the COG4656 was lower in WRH than in SRH and BRH in BD2.

We then compared the relative abundance of functional genes related to aluminum resistance in the same compartment between the two soybean genotypes and found that COG4100, which was recognized as Al- resistance protein, had no significant difference between BX10 and BD2 in any compartments. Similarly, the statistics for COG4100 also showed no significant difference among the three different compartments of the same soybean genotype.

## Discussion

In this study, we provided an overall framework through comparison of the previous opinions [24, 38] on the concept of rhizosphere microbiota with modifications [26, 28]. Different layers of soil were divided into three types, SRH, BRH and WRH. The SRH compartment was separated by a nylon net, the plant roots could not enter other sections although root exudates could invade the soil, meaning that the soil of other compartments may also be affected by the plants. We then aimed to find the effects of the different soybean genotypes on the rhizosphere bacteria community and to explore the relationship between aluminum-tolerance and root bacterial community in plants.

Analysis of alpha diversity of rhizosphere bacterial communities between BX10 and BD2 showed that SRH from BX10 had significantly lower community richness, while the BRH showed significantly lower community richness and higher community coverage in BX10 than that in BD2. The alpha diversity of WRH had no significant difference between the two genotypes. However, compared with SRH and BRH, the WRH had the lowest community richness and diversity along with the highest coverage. From the results above, we conclude that soybean genotypes and sampling compartments exerted some influence on the alpha diversity of rhizosphere bacteria, where the genotype BX10 and the sampling compartment WRH had a significantly lower species richness. The previous studies indicated that the BX10 and BD2 exhibited distinct rhizosphere microbial communities [10], and differences existed in the community structure between BX10 and BD2 at different sampling stages [22]. We compared the beta diversity of rhizosphere bacterial communities between BX10 and BD2. ANOSIM and Adonis analysis were used to assess the variations in bacterial rhizosphere diversity between BX10 and BD2 groups, and the results showed no significant difference. It was reported that uncultured *Acidobacterium*, Chloroflexi, and actinomycete enriched in BD2, and BD2 was affiliated with *Rhizobium* sp. and *Azospirillumbrasilense*, which have the potential to promote plant growth [10, 22]. Thus, we compared the composition of the major bacterial taxa to find out the difference in the composition of rhizosphere bacterial community between BX10 and BD2. For the same sampling compartment, some significant difference in community classification and abundance existed between the samples from two genotypes of soybean, for example, the SRH of BX10 was enriched for *Burkholderia* and depleted for *Ktedonobacter* as compared with BD2. On the contrary, the BRH of BX10 was enriched for *Ktedonobacter* and depleted for *Burkholderia*. These two genera are reported to be associated with nitrogen-fixation [39, 40]. As compared to BD2, the genus *Burkholderia* in BX10 was enriched in SRH while depleted in BRH. Previous studies have proved that some species of *Burkholderia* are aluminum-tolerant, so the distribution of Al-tolerant bacteria in different root compartments of different genotypes of soybean in acidic red soil is a subject worthy of further attention [41]. Root exudation of organic acids is the main mechanism of aluminum-tolerance in plants as they cause Al-detoxification by their Al- chelating ability. In our study, the reason for the differences in bacterial communities corroborate with the previous studies where, two soybean cultivars (Al-tolerant BX10 and Al-sensitive BD2) responsive to Al-stress have different root exudate compositions that resulted in a variable diazotrophic community [22, 23].

The results of cluster trees and heatmaps showed that, the samples from one genotypes of soybean and from one sampling compartment were clustered into one group. Moreover, WRH was clustered far away from BRH and SRH according to the results of PCA, PCoA and PLS-DA, indicating that differences may exist in species composition between them. However, the results of ANOSIM and Adonis (Table S3) indicated that no significant difference existed among the three sampling compartments. When comparing the composition of the major bacterial taxa for the same genotype among the three sampling compartments, we found that the three major phyla Proteobacteria, Actinobacteria and Firmicutes, were significantly higher in the WRH than that in SRH and BRH (Table 2, Figs. 5, S8, and S9). Among them, we focused on the composition of nitrogen-fixing bacteria. The results of ternary plots showed that *Psedumonas* and *Pseudarthrobacteroxydans*, which belong to phyla Proteobacteria and Actinobacteria, had the highest abundance in WRH. The relative abundances of some major bacterial taxa were significantly higher in WRH than that in SRH and BRH. For example, some of the species of *Bacillus* belong to a plant growth-promoting bacteria (PGPB) class. The species *Paenibacillusodorifer*, *Paenibacilluscastaneae* and *Paenibacillus pectinilyticus* belong to the genus *Paenibacillus,* which was originally included within the genus *Bacillus* and reclassified as a separate genus in 1993 [42], and has been detected in a variety of environments including soil, water, rhizosphere, vegetable matter, and so on [43-45]. *P. odorifer* and *P. castaneae* belong to the nitrogen-fixing bacteria [46, 47]. *Pseudomonas brassicacearum* belong to the genus *Pseudomonas* and gets its name because it the roots of *Brassica napus* [48], and *P. brassicacearum* exhibits both pathogenic and growth-promoting properties in its interaction with tomato [49]. The species *Pseudarthrobacteroxydans* belongs to the genus *Pseudarthrobacter,* which is a new genus from the original genus *Arthrobacter*, so *P. oxydans* is also known as *Arthrobacteroxydans*. The model strain *Arthrobacteroxydans* DSM20119 T (GenBank: X83408) was reported to have the highest sequence similarity (99. 132%) with a nitrogen-fixing bacterium with ACC deaminase activity [50]. In summary, the rhizosphere bacteria associated with nitrogen- fixation were significantly higher in WRH than that in SRH and BRH. Thus, we can tentatively draw conclusions that the distance from the sampling compartments to the roots can dramatically impact the species composition of rhizosphere bacterial communities, especially those associated with nitrogen-fixation.

Previous studies reported that the rhizosphere show enrichment processes in rhizosphere microbes with particular functional genes [51]. Thus, we compared the COG function classification and the relative abundance of COG functional classification. Functional prediction analysis of 16S rDNA gene showed that the relative abundances of three special COGs; COG5554, COG5456, and COG4656, were significantly lower in BX_14FBRH than that in BD_14FBRH, while the relative abundance of COG5554 was significantly lower in BX_14FSRH than that in BD_14FSRH. The three COGs are related to nitrogen-fixation, indicating that the genotypes of soybean have impacts on rhizosphere bacterial communities, especially on microbes with particular functional genes associated with nitrogen-fixation. The WRH of BD2 was also higher in COG5554 and lower in COG4656 when compared among the three sampling compartments. These results are consistent with the results of composition of the major bacterial taxa. Major differences existed in the abundance and dominance of rhizosphere microorganisms, which is directly related to the changes in the composition of exudates released by plant roots. The difference in the abundance of nitrogen-fixing genes between BX10 and BD2 is possibly due to differential composition of the exudates secreted by the roots, as it is well known that there are differences in organic acid secretion between the two soybean cultivars under aluminum stress in acidic soil [22, 23].On the other hand, the role of nitrogen-fixing genes in the soil is supposed to be directly related to the activity of nitrogen- fixing bacterial communities. Once rhizosphere nitrogen-fixing bacteria are enriched, they would contribute to the growth of host plants by fixing atmospheric nitrogen into nitrates that are then taken up by plants, and also to help enhance the host tolerance to aluminum stress [13,19-21]. In turn, host plants with better growth under aluminum stress might secrete more root exudates to the rhizosphere, which would help alleviate the aluminum toxicity in soils, and meanwhile, would further shape the composition and diversity of soil microbial communities [11-18].

Because the DNA extraction and sequencing was carried out earlier in this paper, the primers we used were 515f Original and 806r Original as proposed by Caporaso *et al.* in 2012 [31]. Subsequently, Parada *et al*. and Apprill *et al*. proved that the 515f Modified and 806r Modified not only corrected the detection of bacterial communities, but also facilitated the detection of archaeological communities, which has been widely used nowadays [52-55].

In conclusion, in the comparison of alpha diversity, the BRH and SRH of BX10 were significantly lower on community richness than that of BD2, while the WRH showed no significant difference between BX10 and BD2. Among the three sampling compartments of the same soybean genotype (BX10 or BD2), WRH had the lowest community richness and diversity while showed the highest coverage. No significant difference in the overall rhizosphere bacterial communities existed for any compartment between the two genotypes in the comparison of beta diversity. However, compared with BD2, the SRH of BX10 was enriched for Proteobacteria (especially in the genus *Burkholderia*), while BRH of BX10 depleted Proteobacteria. The genus *Burkholderia* is reported to be associated with nitrogen-fixation and also contains bacterial species with aluminum-tolerance. In our study, no significant differences in beta diversity and in the relative abundances of major bacterial taxa were found at phylum level of WRH between BX10 and BD2. However, as compared with BRH and SRH, WRH across the same genotype (BX10 or BD2) always had an enrichment effect on rhizosphere bacteria associated with nitrogen-fixation. Lastly, the relative abundance of COG function classification also showed that BD2 exerted enrichment effects on some COG functional genes that were directly related to nitrogen-fixation, while there were no significant difference in the relative abundance of functional genes related to aluminum resistance between BX10 and BD2.

Finally, our results demonstrate that the genotype of soybean dramatically affected the rhizosphere communities, especially those associated with nitrogen- fixation and/or aluminum-tolerance in the compartments of the BRH and/or SRH, but not in the WRH. However, WRH had the highest abundance of nitrogen-fixing bacteria compared to the two compartments across the two genotypes. The interaction of rhizosphere microbiome and plants is a complex phenomenon which needs to be explored in detail. Overall, our results indicate that soybean genotype and sampling compartments had differential impacts on the composition and structure of rhizosphere communities.

## Supplemental Materials



Supplementary data for this paper are available on-line only at http://jmb.or.kr.

## Figures and Tables

**Table 1:** Statistical analysis of bacterial community structure between the soybean line BX10 and BD2 with three different approaches.

Distance metrics	Group vs. Group	Adonis		ANOSIM	
	
		R^2^	*p-*value	Statistic	*p*-value
Bray-Curtis	BX_14FSRH vs BD_14FSRH	0.4380	0.1	0.7037	0.093
	BX_14FBRH vs BD_14FBRH	0.4949	0.1	0.7778	0.094
	BX_14FWRH vs BD_14FWRH	0.2225	0.3	0.0741	0.295
weighted_Unifrac	BX_14FSRH vs BD_14FSRH	0.4924	0.1	0.5556	0.108
	BX_14FBRH vs BD_14FBRH	0.6024	0.1	0.7778	0.102
	BX_14FWRH vs BD_14FWRH	0.0736	0.9	0.1852	0.197
unweighted_Unifrac	BX_14FSRH vs BD_14FSRH	0.2348	0.1	0.8889	0.108
	BX_14FBRH vs BD_14FBRH	0.2776	0.1	1	0.095
	BX_14FWRH vs BD_14FWRH	0.2199	0.2	0.1481	0.302

ANOSIM and Adonis based on the Bray-Curtis, weighted-Unifrac distance and unweighted-Unifrac distance metrics. The *p*- values (*p* > 0.05) indica X10 and BD2 groups by the tests. Treatment’s details were as in Fig. 2.

**Table 2:** The relative abundance of eight major phyla in each sample (%).

Group	Proteobacteria	Acidobacteria	Chloroflexi	Actinobacteria	Bacteroidetes	Verrucomicrobia	Firmicutes	Thaumarchaeota
BD_14FSRH	**13.04 ± 1.39**	25.55 ± 4.56	**14.89 ± 0.39**	4.22 ± 0.09	3.74 ± 1.06	4.92 ± 1.09	1.76 ± 0.18	15.08 ± 1.37
BX_14FSRH	**25.23 ± 4.07**	24.08 ± 3.87	**9.86 ± 1.63**	3.39 ± 0.73	7.46 ± 4.03	4.42 ± 0.29	1.84 ± 0.24	11.51 ± 4.28
BD_14FBRH	**26.16 ± 1.69**	20.36 ± 1.86	**9.03 ± 2.37**	**2.85 ± 0.46**	**7.97 ± 1.25**	4.61 ± 0.50	6.21 ± 7.08	10.99 ± 6.80
BX_14FBRH	**13.24 ± 2.00**	26.55 ± 3.74	**14.87 ± 2.12**	**4.73 ± 0.61**	**2.31 ± 0.50**	4.77 ± 0.81	2.21 ± 0.25	16.75 ± 1.10
BD_14FWRH	44.89 ± 4.59	5.92 ± 1.57	3.59 ± 0.56	18.76 ± 5.09	4.20 ± 2.85	1.84 ± 0.17	17.20 ± 4.56	0.24 ± 0.10
BX_14FWRH	41.64 ± 25.51	5.54 ± 3.30	3.71 ± 2.04	24.12 ± 7.64	4.62 ± 2.55	1.25 ± 0.62	15.71 ± 15.18	0.25 ± 0.18

SD represents standard deviation. Values were mean ± SD (*n* = 3). The values in bold indicate the significant difference [*p* < 0.05 (*)] between the BX10 and BD2 ing to Student's t-test. Treatment’s details were as in Fig. 2.

**Table 3:** Statistics of COG function classification related to nitrogen-fixation or aluminum resistance.

	BX_14F SRH	BD_14F SRH	BX_14F BRH	BD_14F BRH	BX_14F WRH	BD_14F WRH
COG1348	1968.67	2328.67	1877.33	2270.33	1314.33	1059.67
COG5420	132.33	131.33	129.67	204.67	142.67	185
COG5554	181	143.33	**136.67**	**263.67**	871.67	422.33
COG5456	450.333	351	**346.67**	**575.33**	459.67	392.67
COG4656	**1079**	**1321.67**	**1207.67**	**1354.33**	1047	732.33
COG4100	534.67	541	365.67	578.67	479.67	529.67

Values were mean of 3 replications. Treatment’s details were as in Fig. 2. The significance test method was performed using oneway ANOVA. The values in bold indicate the significant difference (*p* < 0.05) between the BX10 and BD2 groups by the tests.
