# Supplementary material for: Differential Impacts on Bacterial Composition and Abundance in Rhizosphere Compartments between Al-Tolerant and Al-Sensitive Soybean Genotypes in Acidic Soil
Source: J Microbiol Biotechnol. 2020 May 20;30(8):1169–79. doi: 10.4014/jmb.2003.03018 (PMC9728403; doi:10.4014/jmb.2003.03018)
Supplement: Supplementary file 1 [file JMB-30-8-1169-supple.pdf]

J. Microbiol. Biotechnol.
https://doi.org/10.4014/jmb.2003.03018

Supplementary Material

Differential impacts on bacterial composition and abundance in rhizosphere compartments between Al-tolerant and Al-sensitive soybean genotypes in acidic soil

Supplementary Figures and Tables

1. Supplementary Figures

Supplementary Figure S1 Rank abundance curves. The OTU Rank Curve based on operational taxonomic unit (OTU) abundance of bacterial communities. BX and BD represent the Al-tolerance soybean BX10 and Al-sensitive soybean BD2, respectively. F represents the flowering stages. SRH, BRH and WRH represent rhizospheric soil at two sides, rhizospheric soil after brush and rhizospheric soil after wash, respectively.

Supplementary Figure S2 OTU Venn diagram. Treatment's details were as in Figure S1.

Supplementary Figure S3 Pan analysis and Core analysis. Display the common (A) and the unique (B) OTU of samples. Treatment's details were as in Figure S1.

Supplementary Figure S4 Cluster trees of samples. Cluster trees of beta diversity based on the Bray-Curtis distance (A), weighted-unifrac distance (B) and unweighted-unifrac distance (C) of root-associated bacterial communities. Treatment's details were as in Figure S1.

Supplementary Figure S5 Heatmaps of samples. Heatmaps of samples based on the Bray-Curtis distance (A), weighted-unifrac distance (B) and unweighted-unifrac distance (C) of root-associated bacterial communities. Treatment's details were as in Figure S1.

Supplementary Figure S6 PCoA based on weighted unifrac and unweighted unifrac. Principal co-ordinates analysis (PCoA) based on weighted unifrac distance (A) and unweighted unifrac distance (B). Treatment's details were as in Figure S1.

Supplementary Figure S7 NMDS plot. Non-metric multidimensional scaling analysis (NMDS) based on the Bray-Curtis distance (A), weighted-unifrac distance (B) and unweighted-unifrac distance (C) of root-associated bacterial communities. Treatment's details were as in Figure S1.

Supplementary Figure S8 Ternary plots. Ternary plot of samples from three compartments based on OUT level. Treatment's details were as in Figure S1.

Supplementary Figure S9 Taxonomic analysis of samples at different levels. Treatment's details were as in Figure S1.

2. Supplementary Tables

Supplementary Table S1 Statistics of sequencing samples. BX and BD represent the Al-tolerance soybean BX10 and Al-sensitive soybean BD2, respectively. F represents the flowering stages. SRH, BRH and WRH represent rhizospheric soil at two sides, rhizospheric soil after brush and rhizospheric soil after wash, respectively.

Supplementary Table S2 Statistics of OUT of samples. Treatment's details were as in Table S1.

Supplementary Table S3 Statistical analysis of bacterial community structure between the three different sampling compartments based on the Bray–Curtis. Treatment's details were as in Table S1.

Table S4 Relative abundance of COG function classification. Treatment's details were as in Table S1.

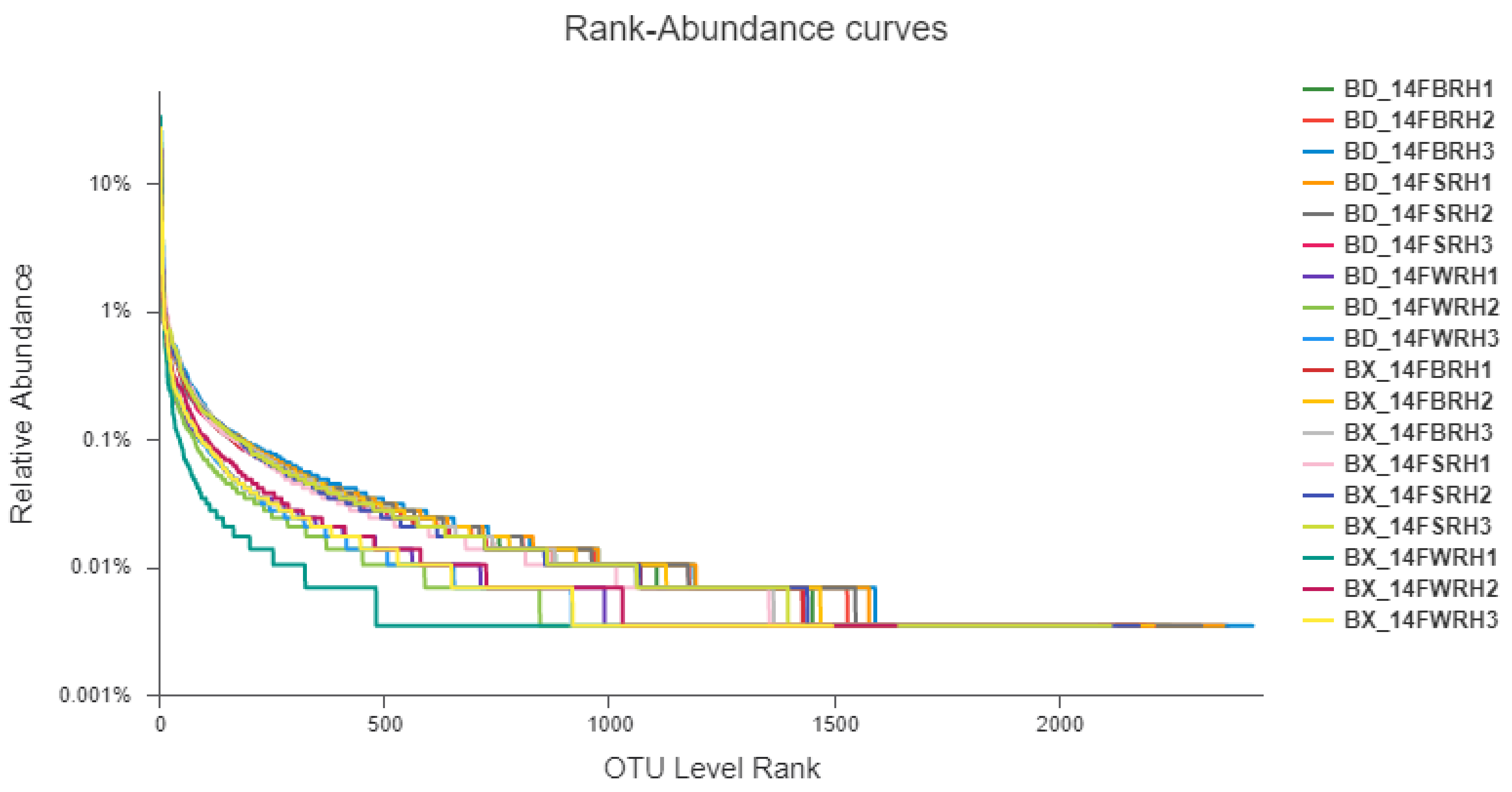

Rank-Abundance curves
Relative Abundance
10%
1%
0.1%
0.01%
0.001%
0
500
1000
1500
2000
OTU Level Rank
BD_14FBRH1
BD_14FBRH2
BD_14FBRH3
BD_14FSRH1
BD_14FSRH2
BD_14FSRH3
BD_14FWRH1
BD_14FWRH2
BD_14FWRH3
BX_14FBRH1
BX_14FBRH2
BX_14FBRH3
BX_14FSRH1
BX_14FSRH2
BX_14FSRH3
BX_14FWRH1
BX_14FWRH2
BX_14FWRH3

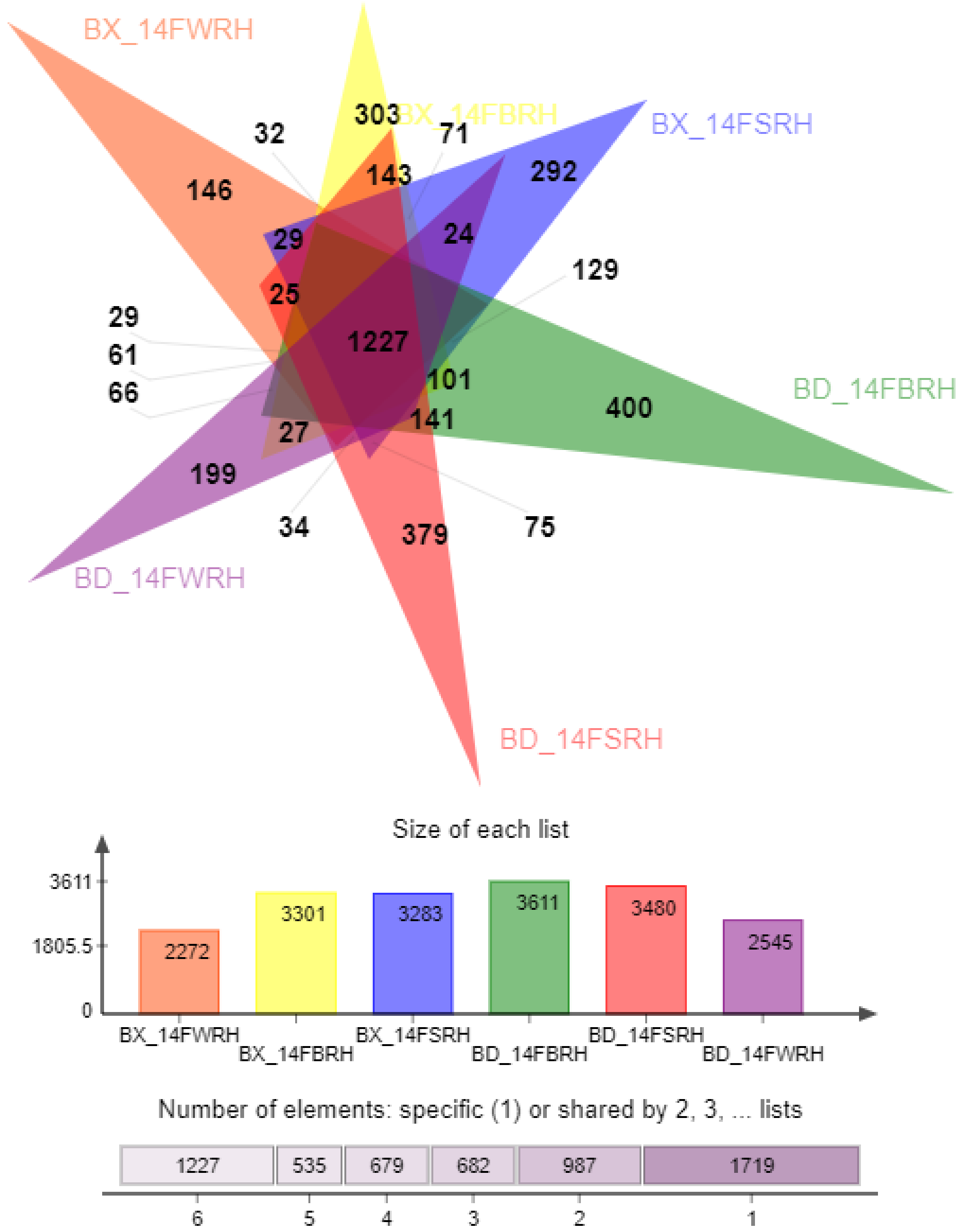
BX_14FWRH
BX_14FBRH
BX_14FSRH
BD_14FBRH
BD_14FSRH
BD_14FWRH
303
32
71
143
146
292
29
24
129
25
1227
29
61
66
101
400
141
27
199
34
379
75
Size of each list
3611
1805.5
0
2272
3301
3283
3611
3480
2545
BX_14FWRH
BX_14FBRH
BX_14FSRH
BD_14FBRH
BD_14FSRH
BD_14FWRH
Number of elements: specific (1) or shared by 2, 3, ... lists
1227
535
679
682
987
1719
6
5
4
3
2
1

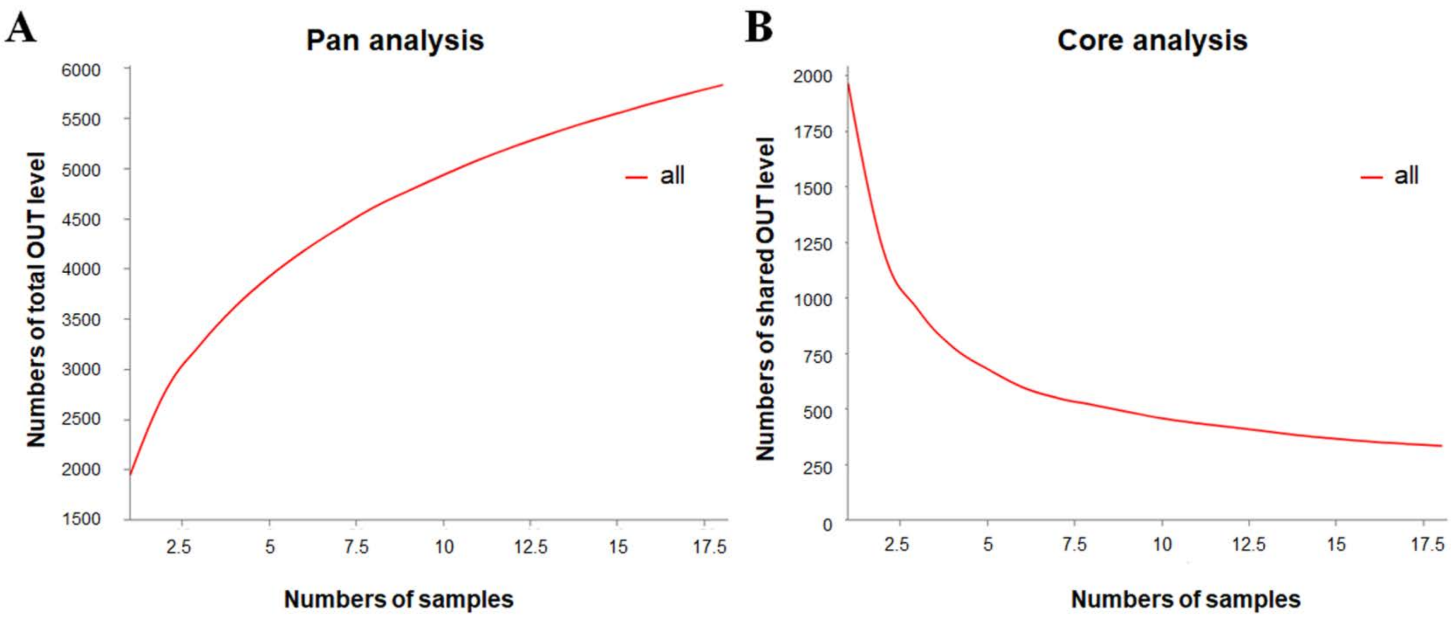
A
Pan analysis
Numbers of total OUT level
6000
5500
5000
4500
4000
3500
3000
2500
2000
1500
2.5
5
7.5
10
12.5
15
17.5
Numbers of samples
all
B
Core analysis
Numbers of shared OUT level
2000
1750
1500
1250
1000
750
500
250
0
2.5
5
7.5
10
12.5
15
17.5
Numbers of samples
all

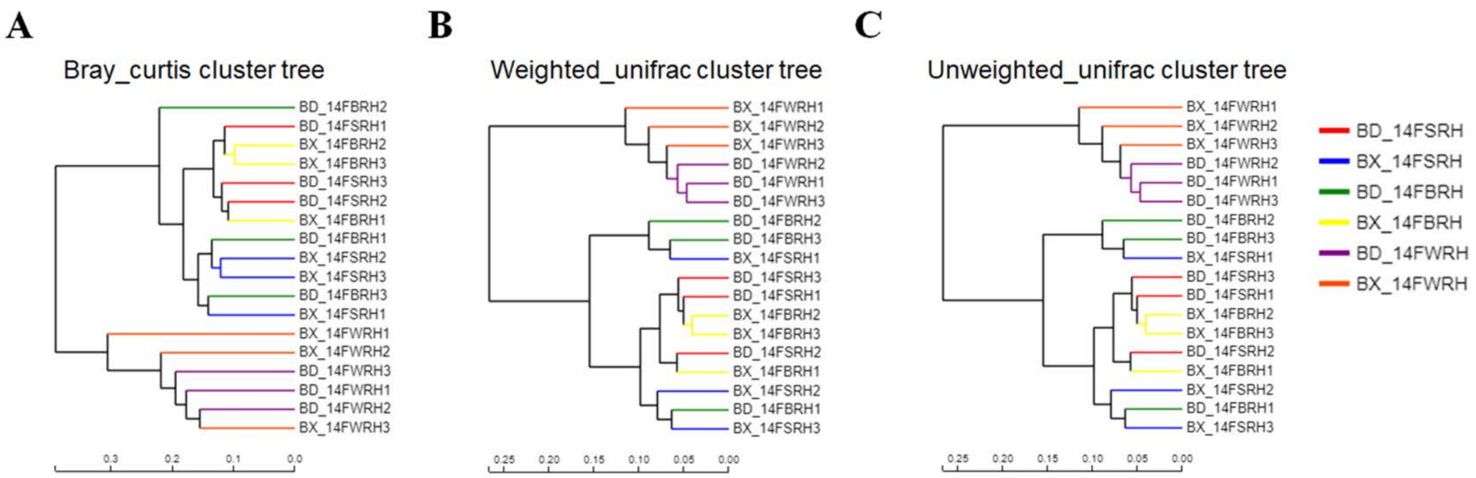
A
Bray_curtis cluster tree
BD_14FBRH2
BD_14FSRH1
BX_14FBRH2
BX_14FBRH3
BD_14FSRH3
BD_14FSRH2
BX_14FBRH1
BD_14FBRH1
BX_14FSRH2
BX_14FSRH3
BD_14FBRH3
BX_14FSRH1
BX_14FWRH1
BX_14FWRH2
BD_14FWRH3
BD_14FWRH1
BD_14FWRH2
BX_14FWRH3
0.3
0.2
0.1
0.0
B
Weighted_unifrac cluster tree
BX_14FWRH1
BX_14FWRH2
BX_14FWRH3
BD_14FWRH2
BD_14FWRH1
BD_14FWRH3
BD_14FBRH2
BD_14FBRH3
BX_14FSRH1
BD_14FSRH3
BD_14FSRH1
BX_14FBRH2
BX_14FBRH3
BD_14FSRH2
BX_14FBRH1
BX_14FSRH2
BD_14FBRH1
BX_14FSRH3
0.25
0.20
0.15
0.10
0.05
0.00
C
Unweighted_unifrac cluster tree
BX_14FWRH1
BX_14FWRH2
BX_14FWRH3
BD_14FWRH2
BD_14FWRH1
BD_14FWRH3
BD_14FBRH2
BD_14FBRH3
BX_14FSRH1
BD_14FSRH3
BD_14FSRH1
BX_14FBRH2
BX_14FBRH3
BD_14FSRH2
BX_14FBRH1
BX_14FSRH2
BD_14FBRH1
BX_14FSRH3
0.25
0.20
0.15
0.10
0.05
0.00
BD_14FSRH
BX_14FSRH
BD_14FBRH
BX_14FBRH
BD_14FWRH
BX_14FWRH

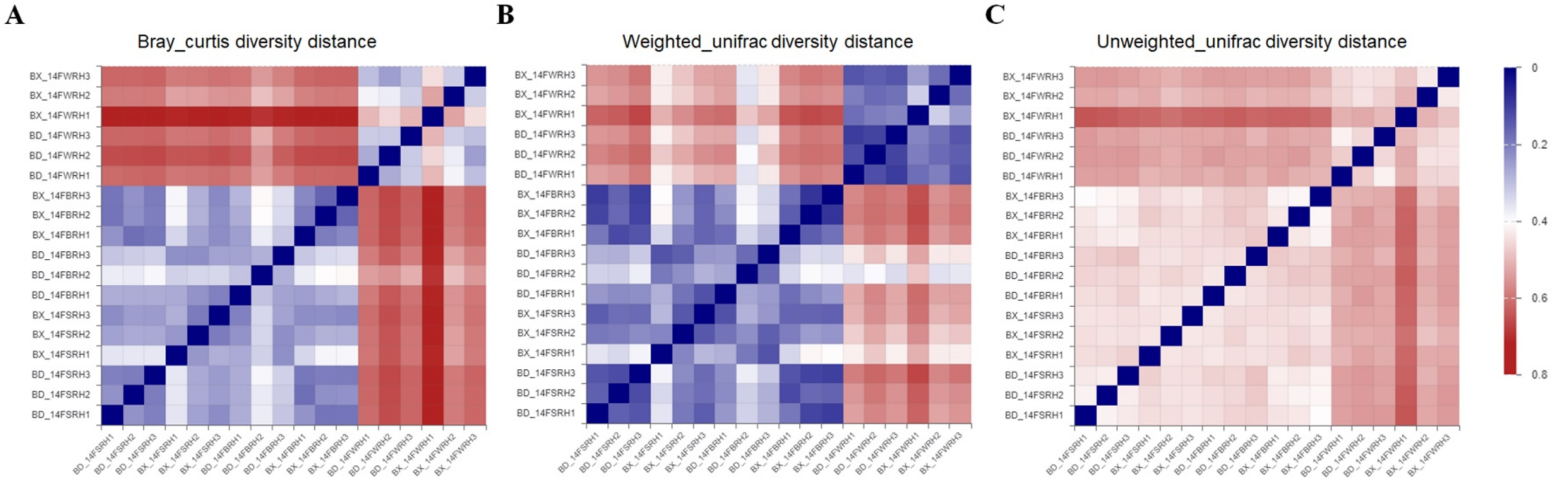
A
Bray_curtis diversity distance
B
Weighted_unifrac diversity distance
C
Unweighted_unifrac diversity distance
BX_14FWRH3
BX_14FWRH2
BX_14FWRH1
BD_14FWRH3
BD_14FWRH2
BD_14FWRH1
BX_14FBRH3
BX_14FBRH2
BX_14FBRH1
BD_14FBRH3
BD_14FBRH2
BD_14FBRH1
BX_14FSRH3
BX_14FSRH2
BX_14FSRH1
BD_14FSRH3
BD_14FSRH2
BD_14FSRH1
0
0.2
0.4
0.6
0.8

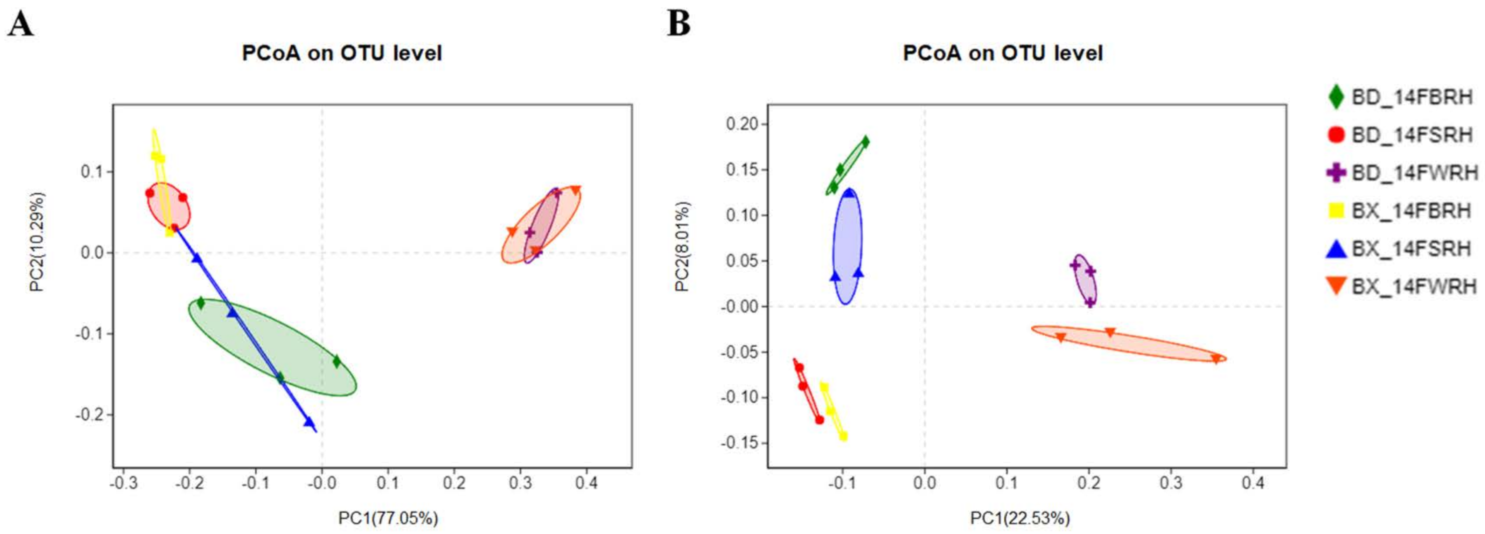
A
PCoA on OTU level
PC2(10.29%)
PC1(77.05%)
B
PCoA on OTU level
PC2(8.01%)
PC1(22.53%)
BD_14FBRH
BD_14FSRH
BD_14FWRH
BX_14FBRH
BX_14FSRH
BX_14FWRH

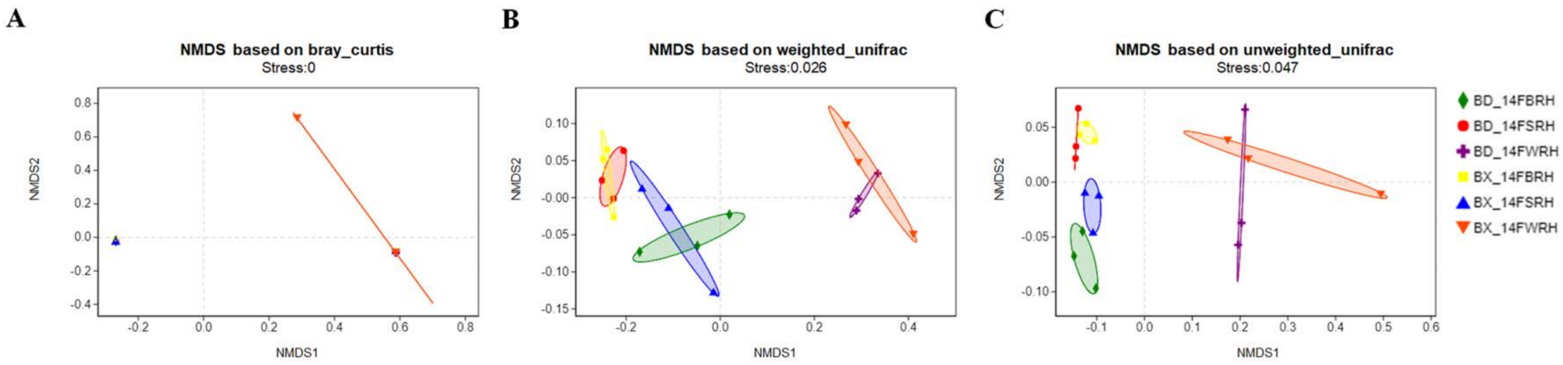
A
NMDS based on bray_curtis
Stress:0
B
NMDS based on weighted_unifrac
Stress:0.026
C
NMDS based on unweighted_unifrac
Stress:0.047
NMDS1
NMDS2
BD_14FBRH
BD_14FSRH
BD_14FWRH
BX_14FBRH
BX_14FSRH
BX_14FWRH

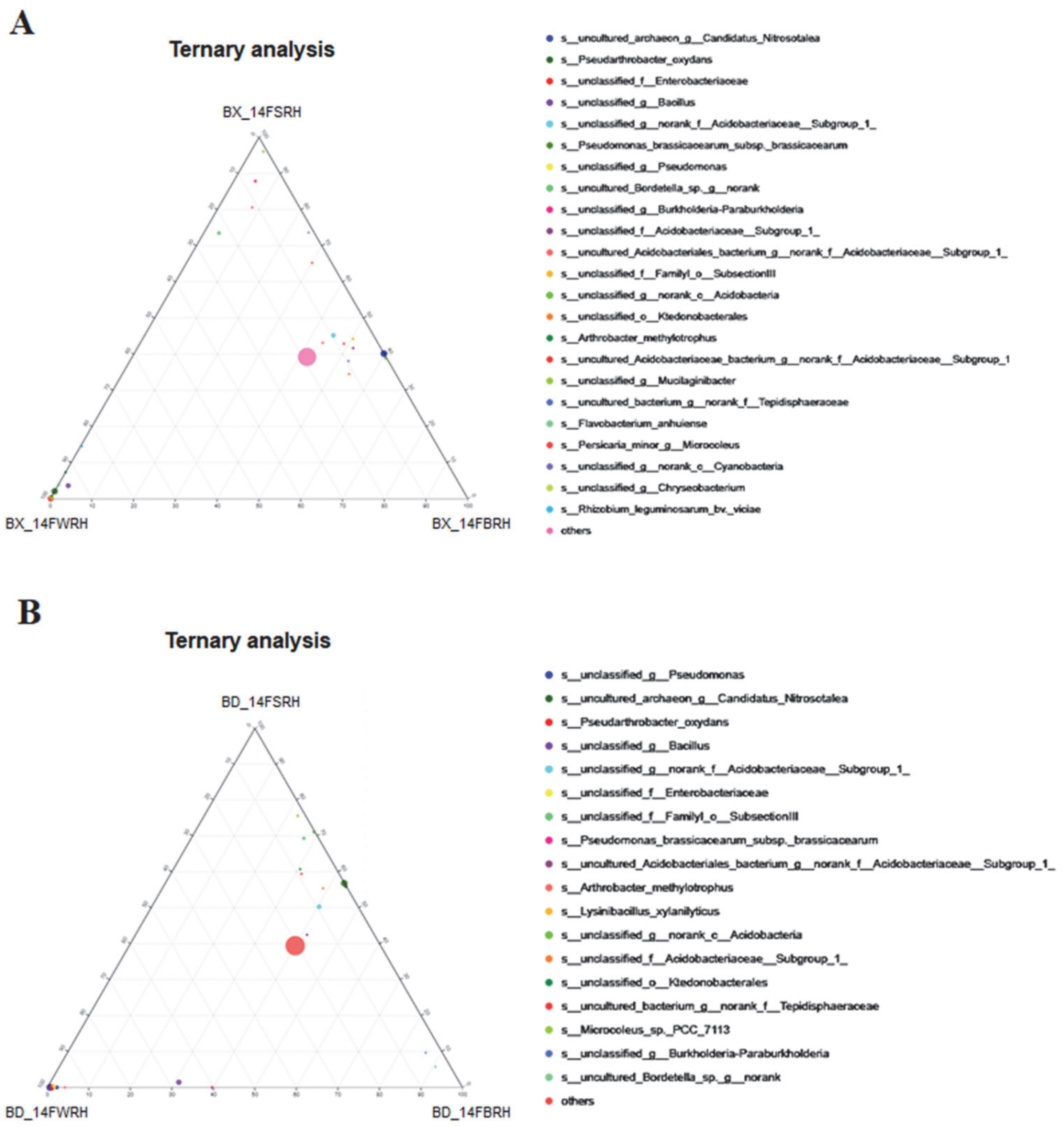
A
Ternary analysis
BX_14FSRH
BX_14FWRH
BX_14FBRH
s__uncultured_archaeon_g__Candidatus_Nitrosotalea
s__Pseudarthrobacter_oxydans
s__unclassified_f__Enterobacteriaceae
s__unclassified_g__Bacillus
s__unclassified_g__norank_f__Acidobacteriaceae__Subgroup_1_
s__Pseudomonas_brassicacearum_subsp._brassicacearum
s__unclassified_g__Pseudomonas
s__uncultured_Bordetella_sp._g__norank
s__unclassified_g__Burkholderia-Paraburkholderia
s__unclassified_f__Acidobacteriaceae__Subgroup_1_
s__uncultured_Acidobacteriales_bacterium_g__norank_f__Acidobacteriaceae__Subgroup_1_
s__unclassified_f__FamilyI_o__SubsectionIII
s__unclassified_g__norank_c__Acidobacteria
s__unclassified_o__Ktedonobacterales
s__Arthrobacter_methylotrophus
s__uncultured_Acidobacteriaceae_bacterium_g__norank_f__Acidobacteriaceae__Subgroup_1
s__unclassified_g__Mucilaginibacter
s__uncultured_bacterium_g__norank_f__Tepidisphaeraceae
s__Flavobacterium_anhuiense
s__Persicaria_minor_g__Microcoleus
s__unclassified_g__norank_c__Cyanobacteria
s__unclassified_g__Chryseobacterium
s__Rhizobium_leguminosarum_bv._viciae
others
B
Ternary analysis
BD_14FSRH
BD_14FWRH
BD_14FBRH
s__unclassified_g__Pseudomonas
s__uncultured_archaeon_g__Candidatus_Nitrosotalea
s__Pseudarthrobacter_oxydans
s__unclassified_g__Bacillus
s__unclassified_g__norank_f__Acidobacteriaceae__Subgroup_1_
s__unclassified_f__Enterobacteriaceae
s__unclassified_f__FamilyI_o__SubsectionIII
s__Pseudomonas_brassicacearum_subsp._brassicacearum
s__uncultured_Acidobacteriales_bacterium_g__norank_f__Acidobacteriaceae__Subgroup_1_
s__Arthrobacter_methylotrophus
s__Lysinibacillus_xylanilyticus
s__unclassified_g__norank_c__Acidobacteria
s__unclassified_f__Acidobacteriaceae__Subgroup_1_
s__unclassified_o__Ktedonobacterales
s__uncultured_bacterium_g__norank_f__Tepidisphaeraceae
s__Microcoleus_sp._PCC_7113
s__unclassified_g__Burkholderia-Paraburkholderia
s__uncultured_Bordetella_sp._g__norank
others

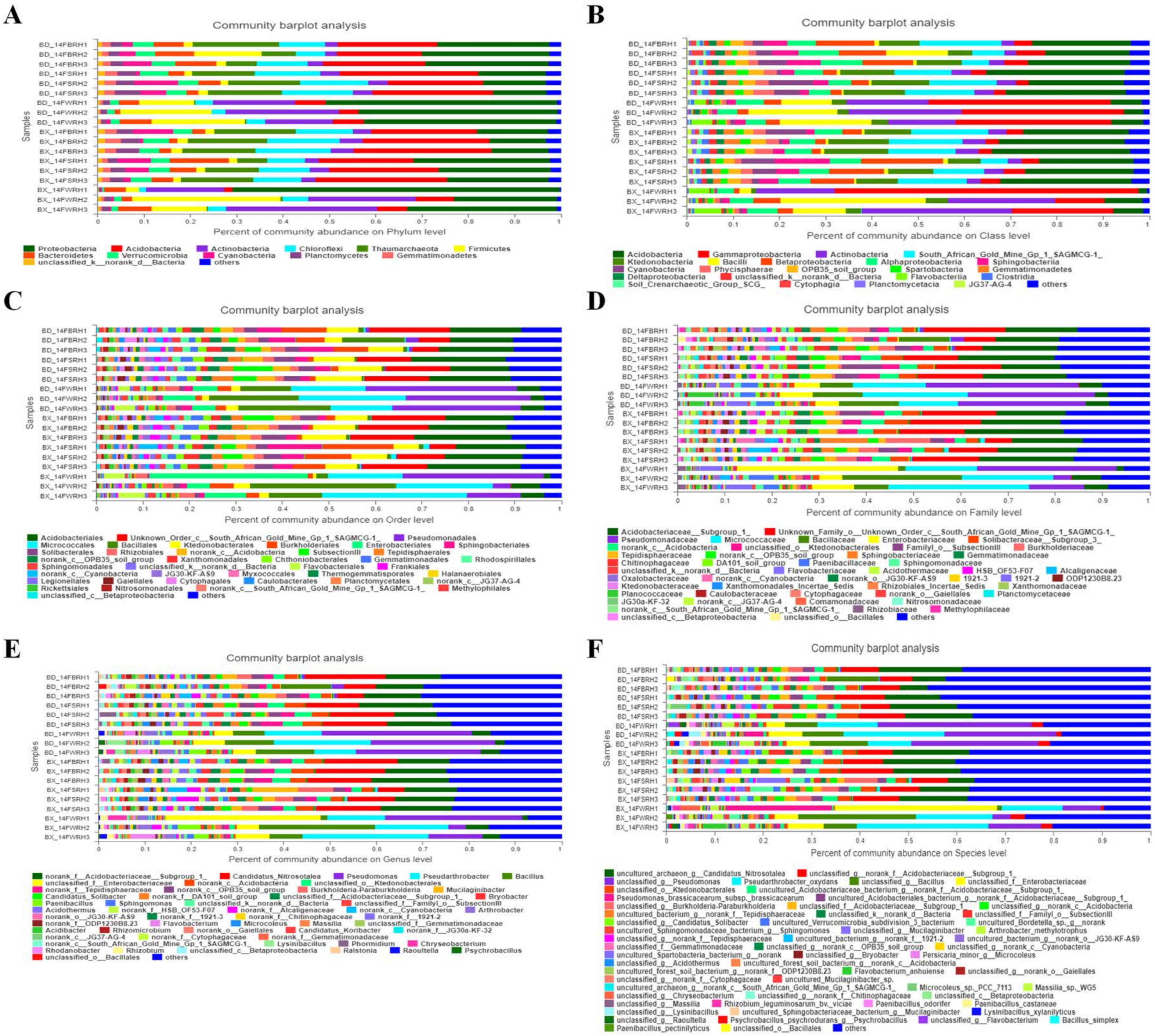
A
Community barplot analysis
BD_14FBRH1
BD_14FBRH2
BD_14FBRH3
BD_14FSRH1
BD_14FSRH2
BD_14FSRH3
BD_14FWRH1
BD_14FWRH2
BD_14FWRH3
BX_14FBRH1
BX_14FBRH2
BX_14FBRH3
BX_14FSRH1
BX_14FSRH2
BX_14FSRH3
BX_14FWRH1
BX_14FWRH2
BX_14FWRH3
Samples
0 0.1 0.2 0.3 0.4 0.5 0.6 0.7 0.8 0.9 1
Percent of community abundance on Phylum level
Proteobacteria Acidobacteria Actinobacteria Chloroflexi Thaumarchaeota Firmicutes
Bacteroidetes Verrucomicrobia Cyanobacteria Planctomycetes Gemmatimonadetes
unclassified_k__norank_d__Bacteria others
B
Community barplot analysis
BD_14FBRH1
BD_14FBRH2
BD_14FBRH3
BD_14FSRH1
BD_14FSRH2
BD_14FSRH3
BD_14FWRH1
BD_14FWRH2
BD_14FWRH3
BX_14FBRH1
BX_14FBRH2
BX_14FBRH3
BX_14FSRH1
BX_14FSRH2
BX_14FSRH3
BX_14FWRH1
BX_14FWRH2
BX_14FWRH3
Samples
0 0.1 0.2 0.3 0.4 0.5 0.6 0.7 0.8 0.9 1
Percent of community abundance on Class level
Acidobacteria Gammaproteobacteria Actinobacteria South_African_Gold_Mine_Gp_1_SAGMCG-1_
Ktedonobacteria Bacilli Betaproteobacteria Alphaproteobacteria Sphingobacteriia
Cyanobacteria Phycisphaerae OPB35_soil_group Spartobacteria Gemmatimonadetes
Deltaproteobacteria unclassified_k__norank_d__Bacteria Flavobacteriia Clostridia
Soil_Crenarchaeotic_Group_SCG_ Cytophagia Planctomycetacia JG37-AG-4 others
C
Community barplot analysis
BD_14FBRH1
BD_14FBRH2
BD_14FBRH3
BD_14FSRH1
BD_14FSRH2
BD_14FSRH3
BD_14FWRH1
BD_14FWRH2
BD_14FWRH3
BX_14FBRH1
BX_14FBRH2
BX_14FBRH3
BX_14FSRH1
BX_14FSRH2
BX_14FSRH3
BX_14FWRH1
BX_14FWRH2
BX_14FWRH3
Samples
0 0.1 0.2 0.3 0.4 0.5 0.6 0.7 0.8 0.9 1
Percent of community abundance on Order level
Acidobacteriales Unknown_Order_c__South_African_Gold_Mine_Gp_1_SAGMCG-1_ Pseudomonadales
Micrococcales Bacillales Ktedonobacterales Burkholderiales Enterobacteriales Sphingobacteriales
Solibacterales Rhizobiales norank_c__Acidobacteria SubsectionIII Tepidisphaerales
norank_c__OPB35_soil_group Xanthomonadales Chthoniobacterales Gemmatimonadales Rhodospirillales
Sphingomonadales unclassified_k__norank_d__Bacteria Flavobacteriales Frankiales
norank_c__Cyanobacteria JG30-KF-AS9 Myxococcales Thermogemmatisporales Halanaerobiales
Legionellales Gaiellales Cytophagales Caulobacterales Planctomycetales norank_c__JG37-AG-4
Rickettsiales Nitrosomonadales norank_c__South_African_Gold_Mine_Gp_1_SAGMCG-1_ Methylophilales
unclassified_c__Betaproteobacteria others
D
Community barplot analysis
BD_14FBRH1
BD_14FBRH2
BD_14FBRH3
BD_14FSRH1
BD_14FSRH2
BD_14FSRH3
BD_14FWRH1
BD_14FWRH2
BD_14FWRH3
BX_14FBRH1
BX_14FBRH2
BX_14FBRH3
BX_14FSRH1
BX_14FSRH2
BX_14FSRH3
BX_14FWRH1
BX_14FWRH2
BX_14FWRH3
Samples
0 0.1 0.2 0.3 0.4 0.5 0.6 0.7 0.8 0.9 1
Percent of community abundance on Family level
Acidobacteriaceae__Subgroup_1_ Unknown_Family_o__Unknown_Order_c__South_African_Gold_Mine_Gp_1_SAGMCG-1_
Pseudomonadaceae Micrococcaceae Bacillaceae Enterobacteriaceae Solibacteraceae__Subgroup_3_
norank_c__Acidobacteria unclassified_o__Ktedonobacterales FamilyI_o__SubsectionIII Burkholderiaceae
Tepidisphaeraceae norank_c__OPB35_soil_group Sphingobacteriaceae Gemmatimonadaceae
Chitinophagaceae DA101_soil_group Paenibacillaceae Sphingomonadaceae
unclassified_k__norank_d__Bacteria Flavobacteriaceae Acidothermaceae HSB_OF53-F07 Alcaligenaceae
Oxalobacteraceae norank_c__Cyanobacteria norank_o__JG30-KF-AS9 1921-3 1921-2 ODP1230B8.23
Ktedonobacteraceae Xanthomonadales_Incertae_Sedis Rhizobiales_Incertae_Sedis Xanthomonadaceae
Planococcaceae Caulobacteraceae Cytophagaceae norank_o__Gaiellales Planctomycetaceae
JG30a-KF-32 norank_c__JG37-AG-4 Comamonadaceae Nitrosomonadaceae
norank_c__South_African_Gold_Mine_Gp_1_SAGMCG-1_ Rhizobiaceae Methylophilaceae
unclassified_c__Betaproteobacteria unclassified_o__Bacillales others
E
Community barplot analysis
BD_14FBRH1
BD_14FBRH2
BD_14FBRH3
BD_14FSRH1
BD_14FSRH2
BD_14FSRH3
BD_14FWRH1
BD_14FWRH2
BD_14FWRH3
BX_14FBRH1
BX_14FBRH2
BX_14FBRH3
BX_14FSRH1
BX_14FSRH2
BX_14FSRH3
BX_14FWRH1
BX_14FWRH2
BX_14FWRH3
Samples
0 0.1 0.2 0.3 0.4 0.5 0.6 0.7 0.8 0.9 1
Percent of community abundance on Genus level
norank_f__Acidobacteriaceae__Subgroup_1_ Candidatus_Nitrosotalea Pseudomonas Pseudarthrobacter Bacillus
unclassified_f__Enterobacteriaceae norank_c__Acidobacteria unclassified_o__Ktedonobacterales
norank_f__Tepidisphaeraceae norank_c__OPB35_soil_group Burkholderia-Paraburkholderia Mucilaginibacter
Candidatus_Solibacter norank_f__DA101_soil_group unclassified_f__Acidobacteriaceae__Subgroup_1_ Bryobacter
Paenibacillus Sphingomonas unclassified_k__norank_d__Bacteria unclassified_f__FamilyI_o__SubsectionIII
Acidothermus norank_f__HSB_OF53-F07 norank_f__Alcaligenaceae norank_c__Cyanobacteria Arthrobacter
norank_o__JG30-KF-AS9 norank_f__1921-3 norank_f__Chitinophagaceae norank_f__1921-2
norank_f__ODP1230B8.23 Flavobacterium Microcoleus Massilia unclassified_f__Gemmatimonadaceae
Acidibacter Rhizomicrobium norank_o__Gaiellales Candidatus_Koribacter norank_f__JG30a-KF-32
norank_c__JG37-AG-4 norank_f__Cytophagaceae norank_f__Gemmatimonadaceae
norank_c__South_African_Gold_Mine_Gp_1_SAGMCG-1_ Lysinibacillus Phormidium Chryseobacterium
Rhodanobacter Rhizobium unclassified_c__Betaproteobacteria Ralstonia Raoultella Psychrobacillus
unclassified_o__Bacillales others
F
Community barplot analysis
BD_14FBRH1
BD_14FBRH2
BD_14FBRH3
BD_14FSRH1
BD_14FSRH2
BD_14FSRH3
BD_14FWRH1
BD_14FWRH2
BD_14FWRH3
BX_14FBRH1
BX_14FBRH2
BX_14FBRH3
BX_14FSRH1
BX_14FSRH2
BX_14FSRH3
BX_14FWRH1
BX_14FWRH2
BX_14FWRH3
Samples
0 0.1 0.2 0.3 0.4 0.5 0.6 0.7 0.8 0.9 1
Percent of community abundance on Species level
uncultured_archaeon_g__Candidatus_Nitrosotalea unclassified_g__norank_f__Acidobacteriaceae__Subgroup_1_
unclassified_g__Pseudomonas Pseudarthrobacter_oxydans unclassified_g__Bacillus unclassified_f__Enterobacteriaceae
unclassified_o__Ktedonobacterales uncultured_Acidobacteriaceae_bacterium_g__norank_f__Acidobacteriaceae__Subgroup_1_
Pseudomonas_brassicacearum_subsp._brassicacearum uncultured_Acidobacteriales_bacterium_g__norank_f__Acidobacteriaceae__Subgroup_1_
unclassified_g__Burkholderia-Paraburkholderia unclassified_f__Acidobacteriaceae__Subgroup_1_ unclassified_g__norank_c__Acidobacteria
uncultured_bacterium_g__norank_f__Tepidisphaeraceae unclassified_k__norank_d__Bacteria unclassified_f__FamilyI_o__SubsectionIII
unclassified_g__Candidatus_Solibacter uncultured_Verrucomicrobia_subdivision_3_bacterium uncultured_Bordetella_sp._g__norank
uncultured_Sphingomonadaceae_bacterium_g__Sphingomonas unclassified_g__Mucilaginibacter Arthrobacter_methylotrophus
unclassified_g__norank_f__Tepidisphaeraceae uncultured_bacterium_g__norank_f__1921-2 uncultured_bacterium_g__norank_o__JG30-KF-AS9
unclassified_f__Gemmatimonadaceae unclassified_g__norank_c__OPB35_soil_group unclassified_g__norank_c__Cyanobacteria
uncultured_Spartobacteria_bacterium_g__norank unclassified_g__Bryobacter Persicaria_minor_g__Microcoleus
unclassified_g__Acidothermus uncultured_forest_soil_bacterium_g__norank_c__Acidobacteria
uncultured_forest_soil_bacterium_g__norank_f__ODP1230B8.23 Flavobacterium_anhuiense unclassified_g__norank_o__Gaiellales
unclassified_g__norank_f__Cytophagaceae uncultured_Mucilaginibacter_sp.
uncultured_archaeon_g__norank_c__South_African_Gold_Mine_Gp_1_SAGMCG-1_ Microcoleus_sp._PCC_7113 Massilia_sp._WG5
unclassified_g__Chryseobacterium unclassified_g__norank_f__Chitinophagaceae unclassified_c__Betaproteobacteria
unclassified_g__Massilia Rhizobium_leguminosarum_bv._viciae Paenibacillus_odorifer Paenibacillus_castaneae
unclassified_g__Lysinibacillus uncultured_Sphingobacteriaceae_bacterium_g__Mucilaginibacter Lysinibacillus_xylanilyticus
unclassified_g__Raoultella Psychrobacillus_psychrodurans_g__Psychrobacillus unclassified_g__Flavobacterium Bacillus_simplex
Paenibacillus_pectinilyticus unclassified_o__Bacillales others

Table S1 Statistics of sequencing samples.

Sample Name	Seq_num	Base_num	Mean_length	Min_length	Max_length	Total Pairs Read Number	Connect Tag Number	Tag number	OTU number	Read Number
BD_14FSRH1	107089	27106461	253.1208714	220	487	108201	107287	70903	2434	64710
BD_14FSRH2	91060	23049259	253.121667	241	487	92184	91283	59890	2308	64710
BD_14FSRH3	109478	27710087	253.1110086	220	487	110653	109675	71653	2363	64710
BX_14FSRH1	89632	22685655	253.0977218	238	489	90903	89880	61453	2143	64710
BX_14FSRH2	91623	23189745	253.0996038	220	487	93064	91821	64228	2193	64710
BX_14FSRH3	96108	24326217	253.1133412	220	487	97241	96297	63913	2174	64710
BD_14FBRH1	93909	23768451	253.1008849	220	487	95152	94156	59844	2308	64710
BD_14FBRH2	93491	23661653	253.0901691	241	487	94854	93715	64894	2366	64710
BD_14FBRH3	100432	25422498	253.1314521	220	487	102422	100687	71289	2474	64710
BX_14FBRH1	98024	24812254	253.1242757	220	487	99032	98212	63626	2290	64710
BX_14FBRH2	98288	24880769	253.141472	236	489	99445	98519	62813	2273	64710
BX_14FBRH3	96934	24537038	253.1313884	221	487	97975	97121	64424	2145	64710
BD_14FWRH1	94585	23930955	253.0100439	245	487	95600	94811	73742	1501	64710
BD_14FWRH2	82108	20776147	253.0343815	250	487	82998	82275	65300	1365	64710
BD_14FWRH3	79740	20175615	253.0174944	248	487	80603	79898	63384	1397	64710
BX_14FWRH1	113472	28710635	253.0195555	250	489	114407	113688	92813	1051	64710
BX_14FWRH2	84112	21280352	253.0001902	243	487	85286	84397	64989	1456	64710
BX_14FWRH3	95675	24207217	253.0150719	248	487	96738	95900	75379	1410	64710
total	1715760	434231008	4555.480593	4201	8772	1736758	1719622	1214537	35651	1164780
average	95320	24123944.89	253.0822552	233.3888889	487.3333333	96486.55556	95534.55556	67474.27778	1980.611111	64710

Table S2 Statistics of OTU of samples.

Domain	Kingdom	Phylum	Class	Order	Family	Genus	Species	OTU	BD_14FBRH1	BD_14FBRH2	BD_14FBRH3	BD_14FSRH1	BD_14FSRH2	BD_14FSRH3	BD_14FWRH1	BD_14FWRH2	BD_14FWRH3	BX_14FBRH1	BX_14FBRH2	BX_14FBRH3	BX_14FSRH1	BX_14FSRH2	BX_14FSRH3	BX_14FWRH1	BX_14FWRH2	BX_14FWRH3	SUM
d__Archaea	k__norank_d__Archaea	p__Bathyarchaeota	c__norank_p__Bathyarchaeota	o__norank_p__Bathyarchaeota	f__norank_p__Bathyarchaeota	g__norank_p__Bathyarchaeota	s__unclassified_g__norank_p__Bathyarchaeota	OTU5074	0	1	1	3	2	0	0	0	0	1	0	0	0	1	1	0	0	0	10
d__Archaea	k__norank_d__Archaea	p__Thaumarchaeota	c__South_African_Gold_Mine_Gp_1_SAGMCG-1_	o__norank_c__South_African_Gold_Mine_Gp_1_SAGMCG-1_	f__norank_c__South_African_Gold_Mine_Gp_1_SAGMCG-1_	g__norank_c__South_African_Gold_Mine_Gp_1_SAGMCG-1_	s__uncultured_archaeon_SAGMA-V	OTU5153	0	0	0	2	2	0	0	0	0	1	2	1	1	2	1	0	0	0	12
d__Archaea	k__norank_d__Archaea	p__Thaumarchaeota	c__Soil_Crenarchaeotic_Group_SCG_	o__Unknown_Order_c__Soil_Crenarchaeotic_Group_SCG_	f__Unknown_Family_o__Unknown_Order_c__Soil_Crenarchaeotic_Group_SCG_	g__Candidatus_Nitrososphaera	s__uncultured_bacterium_g__Candidatus_Nitrososphaera	OTU4255	308	187	172	457	358	368	53	12	30	319	373	277	193	180	236	14	58	40	3635
d__Archaea	k__norank_d__Archaea	p__Thaumarchaeota	c__FHMa11_terrestrial_group	o__norank_c__FHMa11_terrestrial_group	f__norank_c__FHMa11_terrestrial_group	g__norank_c__FHMa11_terrestrial_group	s__uncultured_archaeon_g__norank_c__FHMa11_terrestrial_group	OTU3746	0	6	0	7	12	1	0	0	0	1	25	2	5	2	1	0	0	0	62
d__Archaea	k__norank_d__Archaea	p__Euryarchaeota	c__Thermoplasmata	o__Thermoplasmatales	f__Terrestrial_Miscellaneous_Gp_TMEG_	g__norank_f__Terrestrial_Miscellaneous_Gp_TMEG_	s__unclassified_g__norank_f__Terrestrial_Miscellaneous_Gp_TMEG_	OTU1516	0	0	1	0	0	0	0	0	0	0	0	0	0	0	0	0	0	0	1
d__Archaea	k__norank_d__Archaea	p__Thaumarchaeota	c__Soil_Crenarchaeotic_Group_SCG_	o__norank_c__Soil_Crenarchaeotic_Group_SCG_	f__norank_c__Soil_Crenarchaeotic_Group_SCG_	g__norank_c__Soil_Crenarchaeotic_Group_SCG_	s__uncultured_archaeon_g__norank_c__Soil_Crenarchaeotic_Group_SCG_	OTU4742	2	0	2	0	1	1	1	0	0	5	2	2	4	0	5	0	0	0	25
d__Archaea	k__norank_d__Archaea	p__Thaumarchaeota	c__South_African_Gold_Mine_Gp_1_SAGMCG-1_	o__Unknown_Order_c__South_African_Gold_Mine_Gp_1_SAGMCG-1_	f__Unknown_Family_o__Unknown_Order_c__South_African_Gold_Mine_Gp_1_SAGMCG-1_	g__Candidatus_Nitrosotalea	s__uncultured_archaeon_g__Candidatus_Nitrosotalea	OTU3821	9117	3471	3198	6094	6919	7764	15	1	12	8263	7752	7080	3016	5497	7001	1	13	3	75217
d__Archaea	k__norank_d__Archaea	p__Bathyarchaeota	c__norank_p__Bathyarchaeota	o__norank_p__Bathyarchaeota	f__norank_p__Bathyarchaeota	g__norank_p__Bathyarchaeota	s__unclassified_g__norank_p__Bathyarchaeota	OTU5619	1	0	0	1	0	0	0	0	0	1	0	0	0	0	2	0	0	0	5
d__Archaea	k__norank_d__Archaea	p__Thaumarchaeota	c__Marine_Group_I	o__Unknown_Order_c__Marine_Group_I	f__Unknown_Family_o__Unknown_Order_c__Marine_Group_I	g__Candidatus_Nitrosoarchaeum	s__uncultured_archaeon_g__Candidatus_Nitrosoarchaeum	OTU1976	0	0	0	0	0	0	2	0	0	0	0	0	0	0	0	0	0	0	2
d__Archaea	k__norank_d__Archaea	p__Euryarchaeota	c__Thermoplasmata	o__Thermoplasmatales	f__Marine_Group_II	g__norank_f__Marine_Group_II	s__uncultured_archaeon_g__norank_f__Marine_Group_II	OTU1659	0	0	0	1	1	0	0	0	0	1	0	0	0	0	0	0	0	0	3
d__Archaea	k__norank_d__Archaea	p__Thaumarchaeota	c__Marine_Group_I	o__norank_c__Marine_Group_I	f__norank_c__Marine_Group_I	g__norank_c__Marine_Group_I	s__Thaumarchaeota_archaeon_MY2	OTU2592	0	0	0	0	0	0	2	0	1	0	0	0	0	0	0	0	0	0	3
d__Archaea	k__norank_d__Archaea	p__Thaumarchaeota	c__Marine_Group_I	o__Unknown_Order_c__Marine_Group_I	f__Unknown_Family_o__Unknown_Order_c__Marine_Group_I	g__Candidatus_Nitrosopumilus	s__uncultured_archaeon_g__Candidatus_Nitrosopumilus	OTU931	1	0	0	1	0	0	0	0	0	0	1	0	0	0	1	0	0	0	4
d__Archaea	k__norank_d__Archaea	p__Woesearchaeota__DHVEG-6_	c__norank_p__Woesearchaeota__DHVEG-6_	o__norank_p__Woesearchaeota__DHVEG-6_	f__norank_p__Woesearchaeota__DHVEG-6_	g__norank_p__Woesearchaeota__DHVEG-6_	s__unclassified_g__norank_p__Woesearchaeota__DHVEG-6_	OTU5014	1	0	1	0	17	5	0	0	0	1	0	0	1	0	1	0	0	0	27
d__Archaea	k__norank_d__Archaea	p__Thaumarchaeota	c__FHMa11_terrestrial_group	o__norank_c__FHMa11_terrestrial_group	f__norank_c__FHMa11_terrestrial_group	g__norank_c__FHMa11_terrestrial_group	s__unclassified_g__norank_c__FHMa11_terrestrial_group	OTU5207	3	0	4	5	0	4	3	0	1	0	10	5	1	0	3	1	3	2	45
d__Archaea	k__norank_d__Archaea	p__Thaumarchaeota	c__South_African_Gold_Mine_Gp_1_SAGMCG-1_	o__norank_c__South_African_Gold_Mine_Gp_1_SAGMCG-1_	f__norank_c__South_African_Gold_Mine_Gp_1_SAGMCG-1_	g__norank_c__South_African_Gold_Mine_Gp_1_SAGMCG-1_	s__uncultured_thaumarchaeote_g__norank_c__South_African_Gold_Mine_Gp_1_SAGMCG-1_	OTU5506	3	3	4	7	2	4	2	0	1	6	5	8	2	1	12	0	3	3	66
d__Archaea	k__norank_d__Archaea	p__Euryarchaeota	c__Thermoplasmata	o__Thermoplasmatales	f__Marine_Group_II	g__norank_f__Marine_Group_II	s__unclassified_g__norank_f__Marine_Group_II	OTU3941	0	0	0	0	0	2	0	0	0	1	1	0	1	0	0	0	0	0	5
d__Archaea	k__norank_d__Archaea	p__Thaumarchaeota	c__Soil_Crenarchaeotic_Group_SCG_	o__Unknown_Order_c__Soil_Crenarchaeotic_Group_SCG_	f__Unknown_Family_o__Unknown_Order_c__Soil_Crenarchaeotic_Group_SCG_	g__Candidatus_Nitrososphaera	s__uncultured_archaeon_g__Candidatus_Nitrososphaera	OTU1092	22	7	36	30	13	45	23	4	16	22	35	38	33	47	34	1	23	25	454
d__Archaea	k__norank_d__Archaea	p__Thaumarchaeota	c__South_African_Gold_Mine_Gp_1_SAGMCG-1_	o__Unknown_Order_c__South_African_Gold_Mine_Gp_1_SAGMCG-1_	f__Unknown_Family_o__Unknown_Order_c__South_African_Gold_Mine_Gp_1_SAGMCG-1_	g__Candidatus_Nitrosotalea	s__uncultured_archaeon_g__Candidatus_Nitrosotalea	OTU5412	2081	893	511	1356	2046	1238	3	0	2	1970	1958	1853	598	1164	2092	0	4	1	17770
d__Archaea	k__norank_d__Archaea	p__Thaumarchaeota	c__South_African_Gold_Mine_Gp_1_SAGMCG-1_	o__Unknown_Order_c__South_African_Gold_Mine_Gp_1_SAGMCG-1_	f__Unknown_Family_o__Unknown_Order_c__South_African_Gold_Mine_Gp_1_SAGMCG-1_	g__Candidatus_Nitrosotalea	s__uncultured_archaeon_g__Candidatus_Nitrosotalea	OTU4031	0	3	3	0	3	6	0	0	0	3	5	4	5	3	5	0	0	0	40
d__Archaea	k__norank_d__Archaea	p__Thaumarchaeota	c__South_African_Gold_Mine_Gp_1_SAGMCG-1_	o__unclassified_c__South_African_Gold_Mine_Gp_1_SAGMCG-1_	f__unclassified_c__South_African_Gold_Mine_Gp_1_SAGMCG-1_	g__unclassified_c__South_African_Gold_Mine_Gp_1_SAGMCG-1_	s__unclassified_c__South_African_Gold_Mine_Gp_1_SAGMCG-1_	OTU4862	0	0	0	0	0	0	0	0	0	0	0	0	0	2	0	0	0	0	2
d__Archaea	k__norank_d__Archaea	p__Thaumarchaeota	c__Soil_Crenarchaeotic_Group_SCG_	o__norank_c__Soil_Crenarchaeotic_Group_SCG_	f__norank_c__Soil_Crenarchaeotic_Group_SCG_	g__norank_c__Soil_Crenarchaeotic_Group_SCG_	s__unclassified_g__norank_c__Soil_Crenarchaeotic_Group_SCG_	OTU2996	0	0	7	7	1	6	1	3	1	2	3	2	0	1	1	0	0	4	39
d__Archaea	k__norank_d__Archaea	p__Bathyarchaeota	c__norank_p__Bathyarchaeota	o__norank_p__Bathyarchaeota	f__norank_p__Bathyarchaeota	g__norank_p__Bathyarchaeota	s__unclassified_g__norank_p__Bathyarchaeota	OTU5548	1	0	1	2	7	1	0	0	0	4	5	4	0	0	1	0	0	0	26
d__Archaea	k__norank_d__Archaea	p__Thaumarchaeota	c__Soil_Crenarchaeotic_Group_SCG_	o__norank_c__Soil_Crenarchaeotic_Group_SCG_	f__norank_c__Soil_Crenarchaeotic_Group_SCG_	g__norank_c__Soil_Crenarchaeotic_Group_SCG_	s__uncultured_archaeon_g__norank_c__Soil_Crenarchaeotic_Group_SCG_	OTU4254	52	45	56	87	58	67	34	26	46	68	110	131	51	43	77	21	47	36	1055
d__Archaea	k__norank_d__Archaea	p__Thaumarchaeota	c__FHMa11_terrestrial_group	o__norank_c__FHMa11_terrestrial_group	f__norank_c__FHMa11_terrestrial_group	g__norank_c__FHMa11_terrestrial_group	s__uncultured_crenarchaeote_g__norank_c__FHMa11_terrestrial_group	OTU3238	1	3	3	7	2	5	2	2	1	5	5	5	2	1	2	1	2	2	51
d__Archaea	k__norank_d__Archaea	p__Thaumarchaeota	c__South_African_Gold_Mine_Gp_1_SAGMCG-1_	o__norank_c__South_African_Gold_Mine_Gp_1_SAGMCG-1_	f__norank_c__South_African_Gold_Mine_Gp_1_SAGMCG-1_	g__norank_c__South_African_Gold_Mine_Gp_1_SAGMCG-1_	s__uncultured_thaumarchaeote_g__norank_c__South_African_Gold_Mine_Gp_1_SAGMCG-1_	OTU3602	5	5	4	29	9	16	0	0	1	6	18	15	8	9	7	0	0	1	133
d__Archaea	k__norank_d__Archaea	p__Thaumarchaeota	c__South_African_Gold_Mine_Gp_1_SAGMCG-1_	o__norank_c__South_African_Gold_Mine_Gp_1_SAGMCG-1_	f__norank_c__South_African_Gold_Mine_Gp_1_SAGMCG-1_	g__norank_c__South_African_Gold_Mine_Gp_1_SAGMCG-1_	s__uncultured_archaeon_g__norank_c__South_African_Gold_Mine_Gp_1_SAGMCG-1_	OTU5212	22	12	32	50	90	79	0	0	0	93	40	79	6	54	87	0	0	0	644
d__Archaea	k__norank_d__Archaea	p__Thaumarchaeota	c__FHMa11_terrestrial_group	o__norank_c__FHMa11_terrestrial_group	f__norank_c__FHMa11_terrestrial_group	g__norank_c__FHMa11_terrestrial_group	s__unclassified_g__norank_c__FHMa11_terrestrial_group	OTU3411	0	0	0	2	2	1	0	0	0	0	2	0	0	0	0	0	0	0	7
d__Archaea	k__norank_d__Archaea	p__Thaumarchaeota	c__South_African_Gold_Mine_Gp_1_SAGMCG-1_	o__norank_c__South_African_Gold_Mine_Gp_1_SAGMCG-1_	f__norank_c__South_African_Gold_Mine_Gp_1_SAGMCG-1_	g__norank_c__South_African_Gold_Mine_Gp_1_SAGMCG-1_	s__uncultured_archaeon_g__norank_c__South_African_Gold_Mine_Gp_1_SAGMCG-1_	OTU3735	175	46	72	319	250	417	2	1	0	364	317	287	115	255	316	0	3	1	2940
d__Archaea	k__norank_d__Archaea	p__Thaumarchaeota	c__Soil_Crenarchaeotic_Group_SCG_	o__norank_c__Soil_Crenarchaeotic_Group_SCG_	f__norank_c__Soil_Crenarchaeotic_Group_SCG_	g__norank_c__Soil_Crenarchaeotic_Group_SCG_	s__unidentified_archaeon_g__norank_c__Soil_Crenarchaeotic_Group_SCG_	OTU3179	41	22	53	42	29	32	11	9	12	37	40	52	22	39	37	7	27	16	528
d__Archaea	k__norank_d__Archaea	p__Parvarchaeota	c__Parvarchaeota_Incertae_Sedis	o__Unknown_Order_c__Parvarchaeota_Incertae_Sedis	f__Unknown_Family_o__Unknown_Order_c__Parvarchaeota_Incertae_Sedis	g__Candidatus_Micrarchaeum	s__unclassified_g__Candidatus_Micrarchaeum	OTU1796	2	4	0	6	6	1	0	0	0	3	5	4	1	0	1	0	0	0	33
d__Archaea	k__norank_d__Archaea	p__Thaumarchaeota	c__FHMa11_terrestrial_group	o__norank_c__FHMa11_terrestrial_group	f__norank_c__FHMa11_terrestrial_group	g__norank_c__FHMa11_terrestrial_group	s__unclassified_g__norank_c__FHMa11_terrestrial_group	OTU4396	0	0	0	0	0	0	0	0	0	0	0	2	0	0	0	0	0	0	2
d__Archaea	k__norank_d__Archaea	p__Euryarchaeota	c__Thermoplasmata	o__Thermoplasmatales	f__Marine_Group_II	g__norank_f__Marine_Group_II	s__uncultured_euryarchaeote_g__norank_f__Marine_Group_II	OTU899	0	1	0	0	1	0	0	0	0	0	1	0	0	0	0	0	0	0	3
d__Archaea	k__norank_d__Archaea	p__Thaumarchaeota	c__Soil_Crenarchaeotic_Group_SCG_	o__Unknown_Order_c__Soil_Crenarchaeotic_Group_SCG_	f__Unknown_Family_o__Unknown_Order_c__Soil_Crenarchaeotic_Group_SCG_	g__Candidatus_Nitrososphaera	s__unclassified_g__Candidatus_Nitrososphaera	OTU1017	14	10	17	15	10	7	7	3	10	11	15	10	17	16	11	1	5	10	189
[illegible]	[illegible]	[illegible]	[illegible]	[illegible]	[illegible]	[illegible]	[illegible]	[illegible]	[illegible]	[illegible]	[illegible]	[illegible]	[illegible]	[illegible]	[illegible]	[illegible]	[illegible]	[illegible]	[illegible]	[illegible]	[illegible]	[illegible]	[illegible]	[illegible]	[illegible]	[illegible]	[illegible]

d__Bacteria	k__norank_d__Bacteria	p__Proteobacteria	c__Betaproteobacteria	o__Neisseriales	f__Neisseriaceae	g__norank_f__Neisseriaceae	s__uncultured_beta_proteobacterium_g__norank_f__Neisseriaceae	OTU5220	0	8	4	0	16	2	0	0	0	0	0	0	2	2	2	0	0	0	36
d__Bacteria	k__norank_d__Bacteria	p__Proteobacteria	c__Gammaproteobacteria	o__Legionellales	f__Coxiellaceae	g__Aquicella	s__uncultured_bacterium_g__Aquicella	OTU4776	2	0	0	0	0	1	1	0	0	0	0	1	0	5	0	0	5	2	17
d__Bacteria	k__norank_d__Bacteria	p__Cyanobacteria	c__Cyanobacteria	o__norank_c__Cyanobacteria	f__norank_c__Cyanobacteria	g__norank_c__Cyanobacteria	s__unclassified_g__norank_c__Cyanobacteria	OTU2258	1	0	3	0	3	1	0	0	1	2	2	1	0	0	0	0	1	0	15
d__Bacteria	k__norank_d__Bacteria	p__Proteobacteria	c__Deltaproteobacteria	o__Bdellovibrionales	f__Bdellovibrionaceae	g__Bdellovibrio	s__unclassified_g__Bdellovibrio	OTU1942	0	0	0	0	0	0	3	0	0	0	0	0	0	0	0	0	0	0	3
d__Bacteria	k__norank_d__Bacteria	p__Parcubacteria	c__norank_p__Parcubacteria	o__norank_p__Parcubacteria	f__norank_p__Parcubacteria	g__norank_p__Parcubacteria	s__uncultured_bacterium_g__norank_p__Parcubacteria	OTU423	0	1	0	0	0	0	0	0	0	0	0	0	0	0	3	0	0	0	4
d__Bacteria	k__norank_d__Bacteria	p__Proteobacteria	c__Gammaproteobacteria	o__Legionellales	f__Coxiellaceae	g__Aquicella	s__unclassified_g__Aquicella	OTU5314	0	1	2	0	0	0	2	0	2	0	0	1	1	0	5	0	1	1	16
d__Bacteria	k__norank_d__Bacteria	p__Chloroflexi	c__Anaerolineae	o__Anaerolineales	f__Anaerolineaceae	g__norank_f__Anaerolineaceae	s__uncultured_Gemmatimonadetes_bacterium_g__norank_f__Anaerolineaceae	OTU3464	7	0	6	12	6	11	0	0	0	18	30	4	10	5	7	0	0	0	116
d__Bacteria	k__norank_d__Bacteria	p__Bacteroidetes	c__Cytophagia	o__Cytophagales	f__Cytophagaceae	g__norank_f__Cytophagaceae	s__uncultured_Ohtaekwangia_sp.	OTU2022	0	0	0	0	0	0	2	0	0	0	0	0	0	0	0	0	0	0	2
d__Bacteria	k__norank_d__Bacteria	p__Proteobacteria	c__Gammaproteobacteria	o__X35	f__norank_o__X35	g__norank_o__X35	s__uncultured_bacterium_g__norank_o__X35	OTU2054	2	0	0	1	0	2	2	0	2	0	0	1	0	0	1	0	0	4	15
d__Bacteria	k__norank_d__Bacteria	p__Chloroflexi	c__Thermomicrobia	o__Sphaerobacterales	f__Sphaerobacteraceae	g__Nitrolancea	s__uncultured_bacterium_g__Nitrolancea	OTU916	0	0	0	0	0	0	2	0	0	0	0	0	0	0	0	0	0	0	2
d__Bacteria	k__norank_d__Bacteria	p__Firmicutes	c__Clostridia	o__Clostridiales	f__Clostridiaceae_1	g__Fonticella	s__unclassified_g__Fonticella	OTU3991	0	0	0	0	0	0	0	0	0	0	1	0	0	0	0	0	0	0	1
d__Bacteria	k__norank_d__Bacteria	p__Proteobacteria	c__Gammaproteobacteria	o__unclassified_c__Gammaproteobacteria	f__unclassified_c__Gammaproteobacteria	g__unclassified_c__Gammaproteobacteria	s__unclassified_c__Gammaproteobacteria	OTU1811	0	0	0	0	0	2	0	0	0	0	0	1	0	0	0	0	0	0	3
d__Bacteria	k__norank_d__Bacteria	p__Chloroflexi	c__Ktedonobacteria	o__unclassified_c__Ktedonobacteria	f__unclassified_c__Ktedonobacteria	g__unclassified_c__Ktedonobacteria	s__unclassified_c__Ktedonobacteria	OTU580	0	4	0	3	3	0	1	0	0	1	3	1	0	0	0	2	2	0	20
d__Bacteria	k__norank_d__Bacteria	p__Proteobacteria	c__Deltaproteobacteria	o__Myxococcales	f__mle1-27	g__norank_f__mle1-27	s__unclassified_g__norank_f__mle1-27	OTU474	0	2	0	0	0	0	0	0	0	0	0	0	0	0	0	0	0	0	2
d__Bacteria	k__norank_d__Bacteria	p__Proteobacteria	c__Alphaproteobacteria	o__Caulobacterales	f__Caulobacteraceae	g__Asticcacaulis	s__uncultured_Asticcacaulis_sp.	OTU102	10	0	1	0	0	0	0	0	0	0	0	0	0	0	0	0	0	0	11
d__Bacteria	k__norank_d__Bacteria	p__Chloroflexi	c__Ktedonobacteria	o__Ktedonobacterales	f__1921-3	g__norank_f__1921-3	s__unclassified_g__norank_f__1921-3	OTU1834	2	0	2	1	4	2	1	0	2	0	1	1	0	2	1	0	0	1	20
d__Bacteria	k__norank_d__Bacteria	p__Parcubacteria	c__norank_p__Parcubacteria	o__norank_p__Parcubacteria	f__norank_p__Parcubacteria	g__norank_p__Parcubacteria	s__unclassified_g__norank_p__Parcubacteria	OTU5494	0	0	0	0	0	0	0	0	0	0	0	0	1	0	1	0	0	0	2
d__Bacteria	k__norank_d__Bacteria	p__Planctomycetes	c__Planctomycetacia	o__Planctomycetales	f__Planctomycetaceae	g__Gemmata	s__unclassified_g__Gemmata	OTU1597	0	0	0	0	1	0	0	0	0	0	0	0	0	0	0	0	0	1	2
d__Bacteria	k__norank_d__Bacteria	p__Chlamydiae	c__Chlamydiae	o__Chlamydiales	f__unclassified_o__Chlamydiales	g__unclassified_o__Chlamydiales	s__unclassified_o__Chlamydiales	OTU4247	0	8	7	0	0	0	0	0	0	0	8	0	0	0	0	0	0	0	23
d__Bacteria	k__norank_d__Bacteria	p__Chloroflexi	c__TK10	o__norank_c__TK10	f__norank_c__TK10	g__norank_c__TK10	s__bacterium_Ellin6519	OTU4001	10	5	4	8	13	8	5	1	7	12	15	14	6	0	5	3	3	2	121
d__Bacteria	k__norank_d__Bacteria	p__Proteobacteria	c__Deltaproteobacteria	o__Myxococcales	f__KD3-10	g__norank_f__KD3-10	s__uncultured_bacterium_g__norank_f__KD3-10	OTU4568	1	2	0	0	0	3	0	0	0	1	0	1	1	0	0	0	0	0	9
d__Bacteria	k__norank_d__Bacteria	p__Cyanobacteria	c__Cyanobacteria	o__norank_c__Cyanobacteria	f__norank_c__Cyanobacteria	g__norank_c__Cyanobacteria	s__unclassified_g__norank_c__Cyanobacteria	OTU2783	0	0	0	0	0	0	0	0	0	2	0	0	0	0	0	0	0	0	2
d__Bacteria	k__norank_d__Bacteria	p__Chlamydiae	c__Chlamydiae	o__Chlamydiales	f__Simkaniaceae	g__norank_f__Simkaniaceae	s__uncultured_Chlamydiales_bacterium_g__norank	OTU3987	56	30	58	88	40	78	0	0	0	33	48	36	35	35	52	1	0	0	590
d__Bacteria	k__norank_d__Bacteria	p__Acidobacteria	c__Acidobacteria	o__Acidobacteriales	f__Acidobacteriaceae__Subgroup_1_	g__norank_f__Acidobacteriaceae__Subgroup_1_	s__unclassified_g__norank_f__Acidobacteriaceae__Subgroup_1_	OTU4720	2	12	1	3	0	1	3	0	3	0	1	3	3	3	1	0	10	5	51
d__Bacteria	k__norank_d__Bacteria	p__Acidobacteria	c__Acidobacteria	o__Acidobacteriales	f__Acidobacteriaceae__Subgroup_1_	g__norank_f__Acidobacteriaceae__Subgroup_1_	s__unclassified_g__norank_f__Acidobacteriaceae__Subgroup_1_	OTU4729	0	0	1	1	0	2	0	0	0	2	2	4	8	0	2	0	0	0	22
d__Bacteria	k__norank_d__Bacteria	p__Elusimicrobia	c__Elusimicrobia	o__Lineage_IIa	f__norank_o__Lineage_IIa	g__norank_o__Lineage_IIa	s__unclassified_g__norank_o__Lineage_IIa	OTU3313	0	2	1	0	0	3	0	0	0	2	3	0	1	4	0	0	2	0	18
d__Bacteria	k__norank_d__Bacteria	p__TM6__Dependentiae_	c__norank_p__TM6__Dependentiae_	o__norank_p__TM6__Dependentiae_	f__norank_p__TM6__Dependentiae_	g__norank_p__TM6__Dependentiae_	s__uncultured_bacterium_g__norank_p__TM6__Dependentiae_	OTU872	0	0	0	2	0	0	0	0	0	0	0	1	0	0	2	0	0	0	5
d__Bacteria	k__norank_d__Bacteria	p__Proteobacteria	c__Gammaproteobacteria	o__Legionellales	f__Coxiellaceae	g__Aquicella	s__unclassified_g__Aquicella	OTU2338	0	6	2	0	1	0	4	1	6	1	3	4	6	4	0	2	6	0	46
d__Bacteria	k__norank_d__Bacteria	p__Chloroflexi	c__Ktedonobacteria	o__JG30-KF-AS9	f__norank_o__JG30-KF-AS9	g__norank_o__JG30-KF-AS9	s__unclassified_g__norank_o__JG30-KF-AS9	OTU3443	0	0	0	0	0	0	0	0	1	0	2	0	0	1	0	0	0	0	4
d__Bacteria	k__norank_d__Bacteria	p__Armatimonadetes	c__Fimbriimonadia	o__Fimbriimonadales	f__Fimbriimonadaceae	g__norank_f__Fimbriimonadaceae	s__unclassified_g__norank_f__Fimbriimonadaceae	OTU644	2	1	3	0	2	0	0	1	1	0	0	0	3	0	1	4	1	1	20
d__Bacteria	k__norank_d__Bacteria	p__Nitrospirae	c__Nitrospira	o__norank_c__Nitrospira	f__norank_c__Nitrospira	g__Nitrospira	s__uncultured_Nitrospirales_bacterium	OTU4016	80	65	112	99	72	166	70	32	63	99	106	156	66	86	116	7	45	47	1487
d__Bacteria	k__norank_d__Bacteria	p__Chloroflexi	c__Anaerolineae	o__Anaerolineales	f__Anaerolineaceae	g__norank_f__Anaerolineaceae	s__uncultured_Gemmatimonadetes_bacterium_g__norank_f__Anaerolineaceae	OTU986	0	0	0	1	0	0	0	0	0	0	0	0	0	1	0	0	0	0	2
d__Bacteria	k__norank_d__Bacteria	p__Proteobacteria	c__Alphaproteobacteria	o__Sphingomonadales	f__Sphingomonadaceae	g__Sphingobium	s__Sphingobium_yanoikuyae	OTU6018	3	0	0	0	0	0	0	0	0	1	0	0	0	0	0	7	2	5	18
d__Bacteria	k__norank_d__Bacteria	p__Chloroflexi	c__Ktedonobacteria	o__C0119	f__norank_o__C0119	g__norank_o__C0119	s__uncultured_bacterium_g__norank_o__C0119	OTU3498	0	2	1	0	1	2	2	0	1	4	1	4	0	0	2	0	0	1	21
d__Bacteria	k__norank_d__Bacteria	p__Elusimicrobia	c__Elusimicrobia	o__Lineage_IIb	f__norank_o__Lineage_IIb	g__norank_o__Lineage_IIb	s__uncultured_bacterium_g__norank_o__Lineage_IIb	OTU4618	4	0	4	1	0	2	0	0	0	4	1	1	2	1	1	0	0	0	21
[illegible]																											

d__Bacteria	k__norank_d__Bacteria	p__Actinobacteria	c__Actinobacteria	o__Solirubrobacterales	f__0319-6M6	g__norank_f__0319-6M6	s__unclassified_g__norank_f__0319-6M6	OTU1891	0	1	0	0	0	2	0	0	0	0	0	0	0	0	0	0	1	0	4
d__Bacteria	k__norank_d__Bacteria	p__Proteobacteria	c__unclassified_p__Proteobacteria	o__unclassified_p__Proteobacteria	f__unclassified_p__Proteobacteria	g__unclassified_p__Proteobacteria	s__unclassified_p__Proteobacteria	OTU2162	0	0	0	0	0	0	0	2	0	0	0	0	0	0	0	0	0	0	2
d__Bacteria	k__norank_d__Bacteria	p__unclassified_k__norank_d__Bacteria	c__unclassified_k__norank_d__Bacteria	o__unclassified_k__norank_d__Bacteria	f__unclassified_k__norank_d__Bacteria	g__unclassified_k__norank_d__Bacteria	s__unclassified_k__norank_d__Bacteria	OTU912	2	5	1	3	10	2	1	1	0	4	1	2	2	0	7	0	1	1	43
d__Bacteria	k__norank_d__Bacteria	p__Armatimonadetes	c__norank_p__Armatimonadetes	o__norank_p__Armatimonadetes	f__norank_p__Armatimonadetes	g__norank_p__Armatimonadetes	s__uncultured_bacterium_g__norank_p__Armatimonadetes	OTU5443	0	0	0	0	0	0	0	0	0	0	0	0	0	0	2	0	0	0	2
d__Bacteria	k__norank_d__Bacteria	p__unclassified_k__norank_d__Bacteria	c__unclassified_k__norank_d__Bacteria	o__unclassified_k__norank_d__Bacteria	f__unclassified_k__norank_d__Bacteria	g__unclassified_k__norank_d__Bacteria	s__unclassified_k__norank_d__Bacteria	OTU5036	0	2	5	3	7	4	5	3	0	11	1	6	4	3	0	0	1	0	55
d__Bacteria	k__norank_d__Bacteria	p__Chloroflexi	c__Ktedonobacteria	o__Ktedonobacterales	f__Thermosporotrichaceae	g__norank_f__Thermosporotrichaceae	s__unclassified_g__norank_f__Thermosporotrichaceae	OTU524	21	7	25	28	22	10	2	2	2	30	55	26	9	18	21	0	2	4	284
d__Bacteria	k__norank_d__Bacteria	p__Proteobacteria	c__Alphaproteobacteria	o__Rhodospirillales	f__Rhodospirillaceae	g__Inquilinus	s__unclassified_g__Inquilinus	OTU2158	0	0	0	0	1	0	0	2	0	0	0	0	0	0	0	0	0	0	3
d__Bacteria	k__norank_d__Bacteria	p__Chloroflexi	c__Ktedonobacteria	o__Ktedonobacterales	f__unclassified_o__Ktedonobacterales	g__unclassified_o__Ktedonobacterales	s__unclassified_o__Ktedonobacterales	OTU5546	0	2	0	3	7	0	2	0	0	5	5	5	3	2	2	0	1	1	38
d__Bacteria	k__norank_d__Bacteria	p__Actinobacteria	c__Actinobacteria	o__Micromonosporales	f__Micromonosporaceae	g__Micromonospora	s__unclassified_g__Micromonospora	OTU1425	1	1	2	1	3	0	1	0	1	3	0	1	1	0	0	0	0	0	15
d__Bacteria	k__norank_d__Bacteria	p__Chloroflexi	c__Ktedonobacteria	o__B12-WMSP1	f__norank_o__B12-WMSP1	g__norank_o__B12-WMSP1	s__uncultured_bacterium_g__norank_o__B12-WMSP1	OTU5649	11	6	9	14	11	16	3	2	1	36	27	19	10	14	14	8	6	5	212
d__Bacteria	k__norank_d__Bacteria	p__Proteobacteria	c__Alphaproteobacteria	o__Rhodospirillales	f__KCM-B-15	g__norank_f__KCM-B-15	s__uncultured_bacterium_g__norank_f__KCM-B-15	OTU500	1	10	14	6	1	0	0	0	1	1	3	0	0	1	0	0	0	3	41
d__Bacteria	k__norank_d__Bacteria	p__Proteobacteria	c__Gammaproteobacteria	o__Xanthomonadales	f__Xanthomonadales_Incertae_Sedis	g__Acidibacter	s__uncultured_bacterium_g__Acidibacter	OTU5039	1	0	0	1	2	0	0	0	0	1	0	1	0	1	0	0	0	0	7
d__Bacteria	k__norank_d__Bacteria	p__Firmicutes	c__Clostridia	o__Clostridiales	f__Lachnospiraceae	g__unclassified_f__Lachnospiraceae	s__unclassified_f__Lachnospiraceae	OTU2548	0	0	0	0	2	0	0	0	1	1	0	0	0	0	0	0	2	0	6
d__Bacteria	k__norank_d__Bacteria	p__Planctomycetes	c__Planctomycetacia	o__Planctomycetales	f__Planctomycetaceae	g__norank_f__Planctomycetaceae	s__uncultured_soil_bacterium_g__norank_f__Planctomycetaceae	OTU4157	0	0	5	0	1	4	1	0	0	2	3	3	0	0	0	0	0	0	19
d__Bacteria	k__norank_d__Bacteria	p__Proteobacteria	c__Deltaproteobacteria	o__Bdellovibrionales	f__Bdellovibrionaceae	g__Bdellovibrio	s__uncultured_bacterium_g__Bdellovibrio	OTU5460	2	0	0	5	4	1	0	0	0	0	0	0	0	0	1	0	0	0	13
d__Bacteria	k__norank_d__Bacteria	p__Proteobacteria	c__Deltaproteobacteria	o__Oligoflexales	f__0319-6G20	g__norank_f__0319-6G20	s__unclassified_g__norank_f__0319-6G20	OTU1650	0	1	0	0	4	0	0	0	0	0	0	0	0	0	0	0	0	0	5
d__Bacteria	k__norank_d__Bacteria	p__Chloroflexi	c__TK10	o__norank_c__TK10	f__norank_c__TK10	g__norank_c__TK10	s__bacterium_Ellin6543	OTU5116	5	6	7	33	7	16	5	3	4	12	22	30	0	5	7	1	0	4	167
d__Bacteria	k__norank_d__Bacteria	p__Proteobacteria	c__Gammaproteobacteria	o__Legionellales	f__Coxiellaceae	g__Aquicella	s__uncultured_gamma_proteobacterium_g__Aquicella	OTU762	2	0	2	0	0	0	0	0	0	0	0	0	2	0	1	0	0	0	7
d__Bacteria	k__norank_d__Bacteria	p__Chloroflexi	c__Ktedonobacteria	o__Ktedonobacterales	f__FCPS473	g__norank_f__FCPS473	s__uncultured_Ktedobacteria_bacterium_g__norank_f__FCPS473	OTU3107	6	2	6	6	9	5	2	0	0	4	4	5	2	6	4	2	3	0	66
d__Bacteria	k__norank_d__Bacteria	p__Chloroflexi	c__TK10	o__norank_c__TK10	f__norank_c__TK10	g__norank_c__TK10	s__uncultured_bacterium_SE7	OTU3248	2	9	0	19	8	7	3	2	1	12	12	5	0	0	3	0	1	2	86
d__Bacteria	k__norank_d__Bacteria	p__Gemmatimonadetes	c__Gemmatimonadetes	o__Gemmatimonadales	f__Gemmatimonadaceae	g__norank_f__Gemmatimonadaceae	s__Gemmatimonadetes_bacterium_LX87	OTU1862	0	0	0	0	0	0	0	0	1	0	0	0	0	0	0	0	0	1	2
d__Bacteria	k__norank_d__Bacteria	p__Proteobacteria	c__Betaproteobacteria	o__Neisseriales	f__Neisseriaceae	g__Silvimonas	s__Silvimonas_terrae	OTU2459	9	2	5	0	4	0	1	0	2	1	0	0	6	11	1	0	0	0	42
d__Bacteria	k__norank_d__Bacteria	p__Cyanobacteria	c__Cyanobacteria	o__unclassified_c__Cyanobacteria	f__unclassified_c__Cyanobacteria	g__unclassified_c__Cyanobacteria	s__unclassified_c__Cyanobacteria	OTU5780	0	0	1	0	0	0	0	1	0	2	0	0	0	1	2	1	0	1	9
d__Bacteria	k__norank_d__Bacteria	p__Proteobacteria	c__Deltaproteobacteria	o__Myxococcales	f__Polyangiaceae	g__unclassified_f__Polyangiaceae	s__unclassified_f__Polyangiaceae	OTU630	2	1	3	0	2	1	0	0	1	0	0	1	0	0	1	0	2	1	15
d__Bacteria	k__norank_d__Bacteria	p__Proteobacteria	c__Gammaproteobacteria	o__Xanthomonadales	f__Xanthomonadaceae	g__Stenotrophomonas	s__Stenotrophomonas_chelatiphaga	OTU5753	1	0	1	0	0	0	18	1	1	0	0	1	1	0	0	69	4	1	98
d__Bacteria	k__norank_d__Bacteria	p__Proteobacteria	c__Deltaproteobacteria	o__Bdellovibrionales	f__Bdellovibrionaceae	g__Bdellovibrio	s__unclassified_g__Bdellovibrio	OTU101	2	0	0	0	0	0	0	0	0	0	0	0	0	0	0	0	0	0	2
d__Bacteria	k__norank_d__Bacteria	p__Proteobacteria	c__Deltaproteobacteria	o__Oligoflexales	f__0319-6G20	g__norank_f__0319-6G20	s__uncultured_bacterium_g__norank_f__0319-6G20	OTU1268	0	6	1	3	3	4	0	0	0	0	0	0	0	0	3	0	0	0	20
d__Bacteria	k__norank_d__Bacteria	p__unclassified_k__norank_d__Bacteria	c__unclassified_k__norank_d__Bacteria	o__unclassified_k__norank_d__Bacteria	f__unclassified_k__norank_d__Bacteria	g__unclassified_k__norank_d__Bacteria	s__unclassified_k__norank_d__Bacteria	OTU4459	0	0	0	0	0	0	0	0	0	0	0	1	0	0	0	0	0	0	1
d__Bacteria	k__norank_d__Bacteria	p__Firmicutes	c__Bacilli	o__Bacillales	f__Paenibacillaceae	g__Paenibacillus	s__unclassified_g__Paenibacillus	OTU431	0	1	1	0	0	0	3	0	1	0	0	0	0	0	0	1	0	0	7
d__Bacteria	k__norank_d__Bacteria	p__Chloroflexi	c__Ktedonobacteria	o__Ktedonobacterales	f__unclassified_o__Ktedonobacterales	g__unclassified_o__Ktedonobacterales	s__unclassified_o__Ktedonobacterales	OTU2906	17	14	21	23	30	27	8	11	6	14	21	20	17	22	19	1	14	17	302
d__Bacteria	k__norank_d__Bacteria	p__Proteobacteria	c__Deltaproteobacteria	o__Myxococcales	f__Sandaracinaceae	g__norank_f__Sandaracinaceae	s__unclassified_g__norank_f__Sandaracinaceae	OTU1	0	0	0	0	0	1	0	0	0	0	0	0	0	0	0	0	0	0	1
d__Bacteria	k__norank_d__Bacteria	p__Firmicutes	c__Bacilli	o__Bacillales	f__Paenibacillaceae	g__Paenibacillus	s__unclassified_g__Paenibacillus	OTU435	0	7	0	0	0	0	0	2	3	0	0	2	0	0	0	0	0	3	17
d__Bacteria	k__norank_d__Bacteria	p__Proteobacteria	c__Betaproteobacteria	o__Burkholderiales	f__Burkholderiaceae	g__Limnobacter	s__Limnobacter_litoralis	OTU3263	161	25	118	5	16	14	1	0	0	11	13	2	33	33	36	0	1	0	469
d__Bacteria	k__norank_d__Bacteria	p__Proteobacteria	c__Gammaproteobacteria	o__Thiotrichales	f__unclassified_o__Thiotrichales	g__unclassified_o__Thiotrichales	s__unclassified_o__Thiotrichales	OTU2020	2	1	1	0	0	1	1	0	0	2	0	0	0	0	0	0	0	0	8
d__Bacteria	k__norank_d__Bacteria	p__Proteobacteria	c__Gammaproteobacteria	o__Legionellales	f__Coxiellaceae	g__Aquicella	s__uncultured_bacterium_g__Aquicella	OTU3031	0	0	0	4	2	2	0	0	0	2	3	4	3	1	0	0	0	0	21
d__Bacteria	k__norank_d__Bacteria	p__Firmicutes	c__Clostridia	o__Clostridiales	f__Clostridiaceae_1	g__Clostridium_sensu_stricto_8	s__unclassified_g__Clostridium_sensu_stricto_8	OTU319	5	10	7	0	4	3	0	0	4	2	4	1	4	0	2	0	1	2	49
d__Bacteria	k__norank_d__Bacteria	p__Acidobacteria	c__Acidobacteria	o__Acidobacteriales	f__Acidobacteriaceae__Subgroup_1_	g__norank_f__Acidobacteriaceae__Subgroup_1_	s__unclassified_g__norank_f__Acidobacteriaceae__Subgroup_1_	OTU1243	155	187	161	316	225	264	102	67	72	245	258	321	157	192	417	36	69	101	3345
d__Bacteria	k__norank_d__Bacteria	p__Actinobacteria	c__Actinobacteria	o__Acidimicrobiales	f__unclassified_o__Acidimicrobiales	g__unclassified_o__Acidimicrobiales	s__unclassified_o__Acidimicrobiales	OTU12	2	5	1	2	5	1	1	1	0	0	4	5	1	3	3	0	0	1	35
d__Bacteria	k__norank_d__Bacteria	p__Proteobacteria	c__Alphaproteobacteria	o__Sphingomonadales	f__Sphingomonadaceae	g__Sphingomonas	s__unclassified_g__Sphingomonas	OTU2336	1	7	23	2	1	1	16	2	9	7	3	1	1	6	10	2	6	9	107
d__Bacteria	k__norank_d__Bacteria	p__Proteobacteria	c__Gammaproteobacteria	o__Legionellales	f__Legionellaceae	g__Legionella	s__unclassified_g__Legionella	OTU917	2	0	0	1	0	1	1	0	0	0	0	0	0	0	0	0	0	0	5
d__Bacteria	k__norank_d__Bacteria	p__Proteobacteria	c__Alphaproteobacteria	o__unclassified_c__Alphaproteobacteria	f__unclassified_c__Alphaproteobacteria	g__unclassified_c__Alphaproteobacteria	s__unclassified_c__Alphaproteobacteria	OTU5932	0	0	0	0	0	1	0	0	0	0	3	0	0	0	0	0	3	0	7
d__Bacteria	k__norank_d__Bacteria	p__Chlamydiae	c__Chlamydiae	o__Chlamydiales	f__unclassified_o__Chlamydiales	g__unclassified_o__Chlamydiales	s__unclassified_o__Chlamydiales	OTU5122	0	0	0	0	0	0	0	0	0	0	0	0	0	3	0	0	0	0	3
d__Bacteria	k__norank_d__Bacteria	p__Chloroflexi	c__Ktedonobacteria	o__Ktedonobacterales	f__Thermosporotrichaceae	g__norank_f__Thermosporotrichaceae	s__unclassified_g__norank_f__Thermosporotrichaceae	OTU5370	1	0	0	1	0	2	0	0	0	0	0	0	0	0	3	0	0	0	7
[illegible]	[illegible]	[illegible]	[illegible]	[illegible]	[illegible]	[illegible]	[illegible]	[illegible]	[illegible]	[illegible]	[illegible]	[illegible]	[illegible]	[illegible]	[illegible]	[illegible]	[illegible]	[illegible]	[illegible]	[illegible]	[illegible]	[illegible]	[illegible]	[illegible]	[illegible]	[illegible]	[illegible]

d__Bacteria	k__norank_d__Bacteria	p__unclassified_k__norank_d__Bacteria	c__unclassified_k__norank_d__Bacteria	o__unclassified_k__norank_d__Bacteria	f__unclassified_k__norank_d__Bacteria	g__unclassified_k__norank_d__Bacteria	s__unclassified_k__norank_d__Bacteria	OTU1568	0	1	0	0	3	0	1	0	0	0	0	2	0	0	0	0	0	0	7
d__Bacteria	k__norank_d__Bacteria	p__unclassified_k__norank_d__Bacteria	c__unclassified_k__norank_d__Bacteria	o__unclassified_k__norank_d__Bacteria	f__unclassified_k__norank_d__Bacteria	g__unclassified_k__norank_d__Bacteria	s__unclassified_k__norank_d__Bacteria	OTU1560	0	0	0	0	2	0	0	0	0	0	0	0	0	0	0	0	0	0	2
d__Bacteria	k__norank_d__Bacteria	p__unclassified_k__norank_d__Bacteria	c__unclassified_k__norank_d__Bacteria	o__unclassified_k__norank_d__Bacteria	f__unclassified_k__norank_d__Bacteria	g__unclassified_k__norank_d__Bacteria	s__unclassified_k__norank_d__Bacteria	OTU1561	0	0	0	0	2	2	0	0	0	0	1	0	1	0	0	0	1	2	9
d__Bacteria	k__norank_d__Bacteria	p__Actinobacteria	c__Actinobacteria	o__Streptosporangiales	f__Thermomonosporaceae	g__Actinomadura	s__unclassified_g__Actinomadura	OTU4406	0	1	1	2	0	0	0	0	1	2	0	141	1	0	4	0	1	0	154
d__Bacteria	k__norank_d__Bacteria	p__Proteobacteria	c__Gammaproteobacteria	o__HTA4	f__norank_o__HTA4	g__norank_o__HTA4	s__uncultured_bacterium_g__norank_o__HTA4	OTU265	0	2	0	0	1	0	0	0	0	0	0	0	2	0	0	0	0	1	6
d__Bacteria	k__norank_d__Bacteria	p__Proteobacteria	c__Deltaproteobacteria	o__Bdellovibrionales	f__Bdellovibrionaceae	g__Bdellovibrio	s__uncultured_deep-sea_bacterium_g__Bdellovibrio	OTU4140	1	1	0	0	1	0	0	0	0	0	1	1	0	0	0	0	0	0	5
d__Bacteria	k__norank_d__Bacteria	p__Cyanobacteria	c__Cyanobacteria	o__norank_c__Cyanobacteria	f__norank_c__Cyanobacteria	g__norank_c__Cyanobacteria	s__unclassified_g__norank_c__Cyanobacteria	OTU2943	0	3	1	1	0	0	0	0	0	0	2	0	2	0	0	0	0	0	9
d__Bacteria	k__norank_d__Bacteria	p__Proteobacteria	c__Deltaproteobacteria	o__Bdellovibrionales	f__Bdellovibrionaceae	g__Bdellovibrio	s__Bdellovibrio_sp._JSF2	OTU794	0	0	0	0	0	1	0	0	0	0	0	0	0	0	0	0	0	0	1
d__Bacteria	k__norank_d__Bacteria	p__Chloroflexi	c__TK10	o__norank_c__TK10	f__norank_c__TK10	g__norank_c__TK10	s__uncultured_bacterium_SE7	OTU1174	0	0	0	8	1	0	0	0	0	6	0	0	0	0	1	0	0	0	16
d__Bacteria	k__norank_d__Bacteria	p__Proteobacteria	c__Alphaproteobacteria	o__Rhodospirillales	f__Rhodospirillaceae	g__unclassified_f__Rhodospirillaceae	s__unclassified_f__Rhodospirillaceae	OTU2192	1	6	1	0	0	0	0	0	4	0	0	0	2	0	1	2	0	3	20
d__Bacteria	k__norank_d__Bacteria	p__Proteobacteria	c__Gammaproteobacteria	o__Xanthomonadales	f__Xanthomonadaceae	g__Stenotrophomonas	s__unclassified_g__Stenotrophomonas	OTU2302	1	0	1	0	0	0	0	3	2	0	0	1	4	0	0	16	1	0	29
d__Bacteria	k__norank_d__Bacteria	p__unclassified_k__norank_d__Bacteria	c__unclassified_k__norank_d__Bacteria	o__unclassified_k__norank_d__Bacteria	f__unclassified_k__norank_d__Bacteria	g__unclassified_k__norank_d__Bacteria	s__unclassified_k__norank_d__Bacteria	OTU1205	0	0	0	1	2	2	0	0	0	0	0	0	0	0	0	0	0	0	5
d__Bacteria	k__norank_d__Bacteria	p__unclassified_k__norank_d__Bacteria	c__unclassified_k__norank_d__Bacteria	o__unclassified_k__norank_d__Bacteria	f__unclassified_k__norank_d__Bacteria	g__unclassified_k__norank_d__Bacteria	s__unclassified_k__norank_d__Bacteria	OTU1202	0	0	0	2	1	0	0	0	0	0	0	1	0	0	0	0	0	0	4
d__Bacteria	k__norank_d__Bacteria	p__Planctomycetes	c__Planctomycetacia	o__Planctomycetales	f__Planctomycetaceae	g__norank_f__Planctomycetaceae	s__unclassified_g__norank_f__Planctomycetaceae	OTU3727	1	5	0	1	5	8	0	0	0	0	1	5	3	4	0	0	0	2	35
d__Bacteria	k__norank_d__Bacteria	p__Actinobacteria	c__Actinobacteria	o__Acidimicrobiales	f__norank_o__Acidimicrobiales	g__norank_o__Acidimicrobiales	s__unclassified_g__norank_o__Acidimicrobiales	OTU1750	1	0	0	0	0	1	0	0	0	0	0	0	0	0	0	0	0	0	2
d__Bacteria	k__norank_d__Bacteria	p__unclassified_k__norank_d__Bacteria	c__unclassified_k__norank_d__Bacteria	o__unclassified_k__norank_d__Bacteria	f__unclassified_k__norank_d__Bacteria	g__unclassified_k__norank_d__Bacteria	s__unclassified_k__norank_d__Bacteria	OTU2572	1	1	2	5	7	4	4	1	6	3	5	1	4	3	0	0	1	0	48
d__Bacteria	k__norank_d__Bacteria	p__Chloroflexi	c__Ktedonobacteria	o__Ktedonobacterales	f__unclassified_o__Ktedonobacterales	g__unclassified_o__Ktedonobacterales	s__unclassified_o__Ktedonobacterales	OTU2946	4	6	3	3	4	1	3	0	1	7	12	9	3	2	8	0	0	1	67
d__Bacteria	k__norank_d__Bacteria	p__Actinobacteria	c__Actinobacteria	o__Acidimicrobiales	f__norank_o__Acidimicrobiales	g__norank_o__Acidimicrobiales	s__unclassified_g__norank_o__Acidimicrobiales	OTU1754	3	7	5	8	4	6	0	4	0	5	2	3	3	3	9	1	1	0	64
d__Bacteria	k__norank_d__Bacteria	p__Chloroflexi	c__Chloroflexia	o__Chloroflexales	f__Roseiflexaceae	g__Roseiflexus	s__uncultured_bacterium_g__Roseiflexus	OTU845	0	0	1	0	0	0	0	2	0	0	0	0	0	0	0	0	0	0	3
d__Bacteria	k__norank_d__Bacteria	p__Planctomycetes	c__Planctomycetacia	o__Planctomycetales	f__Planctomycetaceae	g__norank_f__Planctomycetaceae	s__Gemmata_sp._SD2-6	OTU1031	0	0	0	4	0	0	1	0	0	0	0	0	0	1	0	0	0	0	6
d__Bacteria	k__norank_d__Bacteria	p__Acidobacteria	c__Acidobacteria	o__Acidobacteriales	f__Acidobacteriaceae__Subgroup_1_	g__norank_f__Acidobacteriaceae__Subgroup_1_	s__bacterium_Ellin7529	OTU4638	0	0	0	0	0	0	0	0	0	0	0	0	3	0	0	0	0	0	3
d__Bacteria	k__norank_d__Bacteria	p__TM6__Dependentiae_	c__norank_p__TM6__Dependentiae_	o__norank_p__TM6__Dependentiae_	f__norank_p__TM6__Dependentiae_	g__norank_p__TM6__Dependentiae_	s__uncultured_bacterium_g__norank_p__TM6__Dependentiae_	OTU3376	1	1	3	6	1	0	0	0	0	6	0	0	3	2	0	0	2	0	25
d__Bacteria	k__norank_d__Bacteria	p__Chlamydiae	c__Chlamydiae	o__Chlamydiales	f__cvE6	g__norank_f__cvE6	s__uncultured_bacterium_g__norank_f__cvE6	OTU4228	0	0	0	0	0	0	0	0	0	0	5	0	0	0	0	0	0	0	5
d__Bacteria	k__norank_d__Bacteria	p__Verrucomicrobia	c__OPB35_soil_group	o__norank_c__OPB35_soil_group	f__norank_c__OPB35_soil_group	g__norank_c__OPB35_soil_group	s__uncultured_soil_bacterium_g__norank_c__OPB35_soil_group	OTU290	5	8	4	8	5	1	0	0	0	1	3	0	0	2	4	0	0	0	41
d__Bacteria	k__norank_d__Bacteria	p__Gemmatimonadetes	c__Gemmatimonadetes	o__Gemmatimonadales	f__Gemmatimonadaceae	g__norank_f__Gemmatimonadaceae	s__Gemmatimonadetes_bacterium_WY71	OTU3448	3	3	6	4	5	3	0	1	0	1	9	5	1	3	3	0	1	1	49
d__Bacteria	k__norank_d__Bacteria	p__Parcubacteria	c__Candidatus_Adlerbacteria	o__norank_c__Candidatus_Adlerbacteria	f__norank_c__Candidatus_Adlerbacteria	g__norank_c__Candidatus_Adlerbacteria	s__unclassified_g__norank_c__Candidatus_Adlerbacteria	OTU47	2	0	0	0	0	1	0	0	0	0	0	0	0	0	0	0	0	0	3
d__Bacteria	k__norank_d__Bacteria	p__Acidobacteria	c__Acidobacteria	o__Acidobacteriales	f__Acidobacteriaceae__Subgroup_1_	g__unclassified_f__Acidobacteriaceae__Subgroup_1_	s__unclassified_f__Acidobacteriaceae__Subgroup_1_	OTU4114	10	3	15	20	13	22	5	4	4	35	17	15	10	28	20	4	26	14	265
d__Bacteria	k__norank_d__Bacteria	p__Bacteroidetes	c__Sphingobacteriia	o__Sphingobacteriales	f__Chitinophagaceae	g__Taibaiella	s__unclassified_g__Taibaiella	OTU2362	0	0	0	0	0	0	0	0	7	2	0	0	0	0	0	0	0	0	9
d__Bacteria	k__norank_d__Bacteria	p__unclassified_k__norank_d__Bacteria	c__unclassified_k__norank_d__Bacteria	o__unclassified_k__norank_d__Bacteria	f__unclassified_k__norank_d__Bacteria	g__unclassified_k__norank_d__Bacteria	s__unclassified_k__norank_d__Bacteria	OTU2314	0	0	0	0	0	0	0	0	2	0	0	0	0	0	0	0	0	0	2
d__Bacteria	k__norank_d__Bacteria	p__Acidobacteria	c__Acidobacteria	o__Acidobacteriales	f__Acidobacteriaceae__Subgroup_1_	g__norank_f__Acidobacteriaceae__Subgroup_1_	s__unclassified_g__norank_f__Acidobacteriaceae__Subgroup_1_	OTU4493	0	1	0	0	0	0	0	0	0	0	0	1	0	0	0	1	0	0	3
d__Bacteria	k__norank_d__Bacteria	p__Proteobacteria	c__Alphaproteobacteria	o__Rickettsiales	f__Mitochondria	g__norank_f__Mitochondria	s__unclassified_g__norank_f__Mitochondria	OTU6023	0	0	0	0	0	0	0	0	0	0	0	1	0	0	0	0	0	1	2
d__Bacteria	k__norank_d__Bacteria	p__Planctomycetes	c__Planctomycetacia	o__Planctomycetales	f__Planctomycetaceae	g__norank_f__Planctomycetaceae	s__unclassified_g__norank_f__Planctomycetaceae	OTU1851	0	0	0	0	0	3	0	0	0	0	1	0	0	0	0	0	0	0	4
d__Bacteria	k__norank_d__Bacteria	p__Proteobacteria	c__Gammaproteobacteria	o__Xanthomonadales	f__Xanthomonadales_Incertae_Sedis	g__Acidibacter	s__uncultured_proteobacterium_g__Acidibacter	OTU2396	5	9	20	33	6	5	6	3	3	0	1	1	3	0	2	0	0	1	98
d__Bacteria	k__norank_d__Bacteria	p__Gemmatimonadetes	c__Gemmatimonadetes	o__Gemmatimonadales	f__Gemmatimonadaceae	g__Gemmatimonas	s__unclassified_g__Gemmatimonas	OTU3997	0	1	0	0	0	0	0	0	0	0	1	0	3	0	0	0	0	0	5
d__Bacteria	k__norank_d__Bacteria	p__Acidobacteria	c__Acidobacteria	o__norank_c__Acidobacteria	f__norank_c__Acidobacteria	g__norank_c__Acidobacteria	s__unclassified_g__norank_c__Acidobacteria	OTU2647	0	0	0	0	0	0	0	0	0	2	0	0	0	0	0	0	0	0	2
[illegible]	[illegible]	[illegible]	[illegible]	[illegible]	[illegible]	[illegible]	[illegible]	[illegible]	[illegible]	[illegible]	[illegible]	[illegible]	[illegible]	[illegible]	[illegible]	[illegible]	[illegible]	[illegible]	[illegible]	[illegible]	[illegible]	[illegible]	[illegible]	[illegible]	[illegible]	[illegible]	[illegible]

d__Bacteria	k__norank_d__Bacteria	p__Proteobacteria	c__Deltaproteobacteria	o__Oligoflexales	f__0319-6G20	g__norank_f__0319-6G20	s__unclassified_g__norank_f__0319-6G20	OTU3799	1	1	2	2	2	0	2	0	0	3	3	0	6	2	1	0	0	0	25
d__Bacteria	k__norank_d__Bacteria	p__unclassified_k__norank_d__Bacteria	c__unclassified_k__norank_d__Bacteria	o__unclassified_k__norank_d__Bacteria	f__unclassified_k__norank_d__Bacteria	g__unclassified_k__norank_d__Bacteria	s__unclassified_k__norank_d__Bacteria	OTU5159	0	0	0	0	0	0	0	0	0	0	0	0	0	2	0	0	0	0	2
d__Bacteria	k__norank_d__Bacteria	p__Proteobacteria	c__Gammaproteobacteria	o__Legionellales	f__Coxiellaceae	g__Aquicella	s__unclassified_g__Aquicella	OTU5795	0	0	0	0	0	0	0	0	0	0	0	0	0	0	1	1	0	0	2
d__Bacteria	k__norank_d__Bacteria	p__unclassified_k__norank_d__Bacteria	c__unclassified_k__norank_d__Bacteria	o__unclassified_k__norank_d__Bacteria	f__unclassified_k__norank_d__Bacteria	g__unclassified_k__norank_d__Bacteria	s__unclassified_k__norank_d__Bacteria	OTU218	0	2	0	0	0	0	0	0	0	0	0	0	0	0	0	0	0	0	2
d__Bacteria	k__norank_d__Bacteria	p__Planctomycetes	c__Phycisphaerae	o__Tepidisphaerales	f__Tepidisphaeraceae	g__norank_f__Tepidisphaeraceae	s__uncultured_Planctomycetaceae_bacterium_g__norank_f__Tepidisphaeraceae	OTU3130	4	14	8	9	7	5	1	5	1	15	9	2	9	8	9	0	2	0	108
d__Bacteria	k__norank_d__Bacteria	p__Actinobacteria	c__Actinobacteria	o__Acidimicrobiales	f__norank_o__Acidimicrobiales	g__norank_o__Acidimicrobiales	s__unclassified_g__norank_o__Acidimicrobiales	OTU1717	0	0	1	0	0	1	0	0	1	1	0	0	0	0	0	0	0	0	4
d__Bacteria	k__norank_d__Bacteria	p__Actinobacteria	c__Actinobacteria	o__Micromonosporales	f__Micromonosporaceae	g__Actinocatenispora	s__unclassified_g__Actinocatenispora	OTU4324	0	0	0	2	1	0	1	1	0	1	4	0	3	0	1	0	1	0	15
d__Bacteria	k__norank_d__Bacteria	p__Acidobacteria	c__Acidobacteria	o__Solibacterales	f__Solibacteraceae__Subgroup_3_	g__Bryobacter	s__unclassified_g__Bryobacter	OTU1970	0	0	0	0	0	1	1	0	0	0	0	0	2	0	0	0	0	1	5
d__Bacteria	k__norank_d__Bacteria	p__Chloroflexi	c__Ktedonobacteria	o__Ktedonobacterales	f__unclassified_o__Ktedonobacterales	g__unclassified_o__Ktedonobacterales	s__unclassified_o__Ktedonobacterales	OTU2980	1	0	4	2	11	3	1	1	2	1	4	3	8	3	1	0	1	0	46
d__Bacteria	k__norank_d__Bacteria	p__Chlamydiae	c__Chlamydiae	o__Chlamydiales	f__Parachlamydiaceae	g__unclassified_f__Parachlamydiaceae	s__unclassified_f__Parachlamydiaceae	OTU132	2	0	0	0	0	0	0	0	0	0	0	0	0	0	0	0	0	0	2
d__Bacteria	k__norank_d__Bacteria	p__Proteobacteria	c__Alphaproteobacteria	o__Rhodospirillales	f__Acetobacteraceae	g__unclassified_f__Acetobacteraceae	s__unclassified_f__Acetobacteraceae	OTU5262	1	0	2	4	3	7	1	6	0	2	1	2	1	1	2	0	0	2	35
d__Bacteria	k__norank_d__Bacteria	p__Proteobacteria	c__Betaproteobacteria	o__Burkholderiales	f__Comamonadaceae	g__Variovorax	s__uncultured_organism_g__Variovorax	OTU6043	49	14	19	0	0	0	6	2	14	0	0	0	14	4	7	6	8	12	155
d__Bacteria	k__norank_d__Bacteria	p__Gemmatimonadetes	c__Gemmatimonadetes	o__Gemmatimonadales	f__Gemmatimonadaceae	g__norank_f__Gemmatimonadaceae	s__unclassified_g__norank_f__Gemmatimonadaceae	OTU1280	6	14	10	3	0	0	0	0	0	0	2	0	0	3	1	0	0	0	39
d__Bacteria	k__norank_d__Bacteria	p__Chlamydiae	c__Chlamydiae	o__Chlamydiales	f__Parachlamydiaceae	g__unclassified_f__Parachlamydiaceae	s__unclassified_f__Parachlamydiaceae	OTU139	4	0	0	1	0	0	0	0	0	0	0	0	0	0	0	0	0	0	5
d__Bacteria	k__norank_d__Bacteria	p__Proteobacteria	c__Betaproteobacteria	o__Neisseriales	f__Neisseriaceae	g__Vogesella	s__unclassified_g__Vogesella	OTU3858	46	319	102	73	56	6	0	2	1	20	11	3	88	15	64	0	0	1	807
d__Bacteria	k__norank_d__Bacteria	p__Chloroflexi	c__Ktedonobacteria	o__Ktedonobacterales	f__1921-3	g__norank_f__1921-3	s__uncultured_bacterium_g__norank_f__1921-3	OTU3327	1	0	1	0	0	0	0	0	1	0	3	0	0	0	0	0	0	0	6
d__Bacteria	k__norank_d__Bacteria	p__Chloroflexi	c__KD4-96	o__norank_c__KD4-96	f__norank_c__KD4-96	g__norank_c__KD4-96	s__unclassified_g__norank_c__KD4-96	OTU1831	1	2	3	3	2	2	0	3	0	0	2	0	2	0	0	0	1	1	22
d__Bacteria	k__norank_d__Bacteria	p__Cyanobacteria	c__Cyanobacteria	o__norank_c__Cyanobacteria	f__norank_c__Cyanobacteria	g__norank_c__Cyanobacteria	s__unclassified_g__norank_c__Cyanobacteria	OTU1901	0	0	0	1	5	0	3	1	0	4	0	0	0	0	2	1	0	1	18
d__Bacteria	k__norank_d__Bacteria	p__Actinobacteria	c__Actinobacteria	o__Frankiales	f__Geodermatophilaceae	g__unclassified_f__Geodermatophilaceae	s__unclassified_f__Geodermatophilaceae	OTU2957	1	2	4	0	1	0	2	1	2	0	2	0	1	1	0	0	2	1	20
d__Bacteria	k__norank_d__Bacteria	p__Proteobacteria	c__Betaproteobacteria	o__unclassified_c__Betaproteobacteria	f__unclassified_c__Betaproteobacteria	g__unclassified_c__Betaproteobacteria	s__unclassified_c__Betaproteobacteria	OTU4783	0	1	0	0	0	0	0	0	0	0	0	0	0	1	0	0	0	0	2
d__Bacteria	k__norank_d__Bacteria	p__Chloroflexi	c__Anaerolineae	o__Anaerolineales	f__Anaerolineaceae	g__norank_f__Anaerolineaceae	s__uncultured_Gemmatimonadetes_bacterium_g__norank_f__Anaerolineaceae	OTU4359	1	1	0	0	0	0	0	0	0	0	4	0	0	2	0	0	0	0	8
d__Bacteria	k__norank_d__Bacteria	p__Proteobacteria	c__Gammaproteobacteria	o__Legionellales	f__Legionellaceae	g__Legionella	s__unclassified_g__Legionella	OTU533	0	2	0	0	0	0	0	0	0	0	0	0	0	0	0	0	0	0	2
d__Bacteria	k__norank_d__Bacteria	p__Acidobacteria	c__Acidobacteria	o__unclassified_c__Acidobacteria	f__unclassified_c__Acidobacteria	g__unclassified_c__Acidobacteria	s__unclassified_c__Acidobacteria	OTU716	0	0	2	0	0	0	0	0	0	0	0	0	0	0	0	0	0	0	2
d__Bacteria	k__norank_d__Bacteria	p__Actinobacteria	c__Actinobacteria	o__Pseudonocardiales	f__Pseudonocardiaceae	g__Goodfellowiella	s__Goodfellowiella_coeruleoviolacea	OTU1875	0	0	0	0	0	4	0	0	0	0	0	0	0	0	0	0	0	0	4
d__Bacteria	k__norank_d__Bacteria	p__Proteobacteria	c__Alphaproteobacteria	o__Rhodospirillales	f__DA111	g__norank_f__DA111	s__unclassified_g__norank_f__DA111	OTU3687	3	0	1	1	0	2	2	2	1	3	1	1	0	2	1	0	2	2	24
d__Bacteria	k__norank_d__Bacteria	p__Proteobacteria	c__Alphaproteobacteria	o__Rhodospirillales	f__DA111	g__norank_f__DA111	s__unclassified_g__norank_f__DA111	OTU3689	12	14	8	11	14	3	4	2	2	2	7	6	10	2	12	0	0	5	114
d__Bacteria	k__norank_d__Bacteria	p__Firmicutes	c__Clostridia	o__Clostridiales	f__Clostridiaceae_1	g__unclassified_f__Clostridiaceae_1	s__unclassified_f__Clostridiaceae_1	OTU5378	0	27	14	4	6	2	6	1	2	1	7	4	0	4	3	2	10	2	95
d__Bacteria	k__norank_d__Bacteria	p__Bacteroidetes	c__Cytophagia	o__Cytophagales	f__Cytophagaceae	g__Sporocytophaga	s__uncultured_bacterium_g__Sporocytophaga	OTU1990	13	1	1	0	0	0	38	6	1	0	0	0	0	2	0	0	14	0	76
d__Bacteria	k__norank_d__Bacteria	p__Acidobacteria	c__Acidobacteria	o__Solibacterales	f__Solibacteraceae__Subgroup_3_	g__AKIW659	s__uncultured_bacterium_g__AKIW659	OTU3363	0	0	0	0	3	0	0	0	0	0	4	0	0	0	0	0	0	0	7
d__Bacteria	k__norank_d__Bacteria	p__Proteobacteria	c__Gammaproteobacteria	o__Legionellales	f__Coxiellaceae	g__Aquicella	s__unclassified_g__Aquicella	OTU4227	0	2	2	1	0	0	1	1	1	0	6	0	0	1	3	0	0	1	19
d__Bacteria	k__norank_d__Bacteria	p__Chloroflexi	c__JG37-AG-4	o__norank_c__JG37-AG-4	f__norank_c__JG37-AG-4	g__norank_c__JG37-AG-4	s__unclassified_g__norank_c__JG37-AG-4	OTU1505	0	0	0	0	2	0	0	0	0	0	0	0	0	0	0	0	0	0	2
d__Bacteria	k__norank_d__Bacteria	p__Proteobacteria	c__Alphaproteobacteria	o__Rhodospirillales	f__DA111	g__norank_f__DA111	s__unclassified_g__norank_f__DA111	OTU2621	20	18	22	23	19	25	0	1	4	21	31	47	11	12	22	0	7	3	286
d__Bacteria	k__norank_d__Bacteria	p__Chloroflexi	c__TK10	o__norank_c__TK10	f__norank_c__TK10	g__norank_c__TK10	s__Chloroflexi_bacterium_JEA33	OTU3977	2	0	0	3	0	0	0	3	0	0	1	0	0	0	0	0	0	0	9
d__Bacteria	k__norank_d__Bacteria	p__TM6__Dependentiae_	c__norank_p__TM6__Dependentiae_	o__norank_p__TM6__Dependentiae_	f__norank_p__TM6__Dependentiae_	g__norank_p__TM6__Dependentiae_	s__uncultured_soil_bacterium_g__norank_p__TM6__Dependentiae_	OTU3291	0	3	0	0	0	3	0	0	1	1	6	1	0	2	0	0	0	3	20
d__Bacteria	k__norank_d__Bacteria	p__Planctomycetes	c__Planctomycetacia	o__Planctomycetales	f__Planctomycetaceae	g__norank_f__Planctomycetaceae	s__uncultured_Gemmata_sp._g__norank	OTU2789	0	0	2	0	0	1	0	0	0	3	0	1	0	0	0	0	1	0	8
[illegible]	[illegible]	[illegible]	[illegible]	[illegible]	[illegible]	[illegible]	[illegible]	[illegible]	[illegible]	[illegible]	[illegible]	[illegible]	[illegible]	[illegible]	[illegible]	[illegible]	[illegible]	[illegible]	[illegible]	[illegible]	[illegible]	[illegible]	[illegible]	[illegible]	[illegible]	[illegible]	[illegible]

d__Bacteria	k__norank_d__Bacteria	p__TM6__Dependentiae_	c__norank_p__TM6__Dependentiae_	o__norank_p__TM6__Dependentiae_	f__norank_p__TM6__Dependentiae_	g__norank_p__TM6__Dependentiae_	s__unclassified_g__norank_p__TM6__Dependentiae_	OTU5933	0	0	0	0	0	1	0	0	0	0	0	0	0	1	0	0	1	0	3
d__Bacteria	k__norank_d__Bacteria	p__Chlamydiae	c__Chlamydiae	o__Chlamydiales	f__cvE6	g__norank_f__cvE6	s__uncultured_bacterium_g__norank_f__cvE6	OTU4458	0	0	1	0	0	3	0	0	0	1	1	5	0	0	0	0	0	0	11
d__Bacteria	k__norank_d__Bacteria	p__Proteobacteria	c__Alphaproteobacteria	o__Rickettsiales	f__Mitochondria	g__norank_f__Mitochondria	s__Spongospora_subterranea	OTU2182	0	0	3	0	0	2	1	1	2	0	5	0	0	0	0	3	0	0	17
d__Bacteria	k__norank_d__Bacteria	p__Gemmatimonadetes	c__Gemmatimonadetes	o__Gemmatimonadales	f__Gemmatimonadaceae	g__unclassified_f__Gemmatimonadaceae	s__unclassified_f__Gemmatimonadaceae	OTU1411	1	0	0	0	4	1	0	0	0	0	0	4	0	0	0	0	0	0	10
d__Bacteria	k__norank_d__Bacteria	p__Armatimonadetes	c__Fimbriimonadia	o__Fimbriimonadales	f__Fimbriimonadaceae	g__norank_f__Fimbriimonadaceae	s__unclassified_g__norank_f__Fimbriimonadaceae	OTU5807	0	0	1	0	0	0	1	0	0	0	0	0	0	0	0	1	0	0	3
d__Bacteria	k__norank_d__Bacteria	p__Parcubacteria	c__unclassified_p__Parcubacteria	o__unclassified_p__Parcubacteria	f__unclassified_p__Parcubacteria	g__unclassified_p__Parcubacteria	s__unclassified_p__Parcubacteria	OTU4984	0	0	0	0	1	0	0	0	0	0	0	0	5	5	0	0	0	0	11
d__Bacteria	k__norank_d__Bacteria	p__Proteobacteria	c__Gammaproteobacteria	o__unclassified_c__Gammaproteobacteria	f__unclassified_c__Gammaproteobacteria	g__unclassified_c__Gammaproteobacteria	s__unclassified_c__Gammaproteobacteria	OTU5866	0	0	0	0	0	0	0	0	0	0	0	0	0	0	0	0	2	0	2
d__Bacteria	k__norank_d__Bacteria	p__Chloroflexi	c__Ktedonobacteria	o__B12-WMSP1	f__norank_o__B12-WMSP1	g__norank_o__B12-WMSP1	s__unclassified_g__norank_o__B12-WMSP1	OTU1673	0	0	0	0	2	0	0	0	0	0	0	0	0	0	0	0	0	0	2
d__Bacteria	k__norank_d__Bacteria	p__Armatimonadetes	c__Chthonomonadetes	o__Chthonomonadales	f__Chthonomonadaceae	g__Chthonomonas	s__unclassified_g__Chthonomonas	OTU2566	1	4	5	4	5	4	1	2	2	4	7	8	9	3	4	0	4	2	69
d__Bacteria	k__norank_d__Bacteria	p__Cyanobacteria	c__Cyanobacteria	o__SubsectionIII	f__FamilyI_o__SubsectionIII	g__Microcoleus	s__unclassified_g__Microcoleus	OTU225	0	6	0	0	1	0	0	0	0	0	0	0	0	0	0	0	0	0	7
d__Bacteria	k__norank_d__Bacteria	p__Proteobacteria	c__Gammaproteobacteria	o__Legionellales	f__Coxiellaceae	g__Coxiella	s__unclassified_g__Coxiella	OTU5567	0	1	0	2	1	0	0	0	0	0	0	0	0	0	1	0	0	0	5
d__Bacteria	k__norank_d__Bacteria	p__Proteobacteria	c__Gammaproteobacteria	o__Xanthomonadales	f__Xanthomonadaceae	g__Lysobacter	s__unclassified_g__Lysobacter	OTU2013	42	0	0	0	2	0	12	0	0	0	0	0	0	0	0	0	0	0	56
d__Bacteria	k__norank_d__Bacteria	p__unclassified_k__norank_d__Bacteria	c__unclassified_k__norank_d__Bacteria	o__unclassified_k__norank_d__Bacteria	f__unclassified_k__norank_d__Bacteria	g__unclassified_k__norank_d__Bacteria	s__unclassified_k__norank_d__Bacteria	OTU1285	0	0	0	3	0	0	0	0	0	0	0	0	0	0	0	0	0	0	3
d__Bacteria	k__norank_d__Bacteria	p__Chloroflexi	c__Ktedonobacteria	o__Thermogemmatisporales	f__1921-2	g__norank_f__1921-2	s__unclassified_g__norank_f__1921-2	OTU4036	7	1	10	6	11	6	3	2	5	10	10	19	7	16	8	0	2	3	126
d__Bacteria	k__norank_d__Bacteria	p__Proteobacteria	c__Alphaproteobacteria	o__Rhizobiales	f__Rhodobiaceae	g__norank_f__Rhodobiaceae	s__unclassified_g__norank_f__Rhodobiaceae	OTU2085	0	0	0	0	0	0	3	0	0	0	0	0	0	0	0	0	0	0	3
d__Bacteria	k__norank_d__Bacteria	p__Armatimonadetes	c__norank_p__Armatimonadetes	o__norank_p__Armatimonadetes	f__norank_p__Armatimonadetes	g__norank_p__Armatimonadetes	s__unclassified_g__norank_p__Armatimonadetes	OTU3283	6	0	0	2	9	7	5	1	0	7	12	7	1	0	0	0	1	1	59
d__Bacteria	k__norank_d__Bacteria	p__Bacteroidetes	c__Sphingobacteriia	o__Sphingobacteriales	f__Chitinophagaceae	g__unclassified_f__Chitinophagaceae	s__unclassified_f__Chitinophagaceae	OTU2552	1	0	0	0	0	1	0	0	5	0	0	0	0	0	0	0	0	1	8
d__Bacteria	k__norank_d__Bacteria	p__Firmicutes	c__Bacilli	o__Bacillales	f__Paenibacillaceae	g__Paenibacillus	s__unclassified_g__Paenibacillus	OTU5811	0	0	0	0	0	0	0	0	0	0	0	0	0	0	0	1	0	0	1
d__Bacteria	k__norank_d__Bacteria	p__Actinobacteria	c__Actinobacteria	o__Frankiales	f__Acidothermaceae	g__Acidothermus	s__unclassified_g__Acidothermus	OTU4933	1	0	1	4	3	7	0	0	0	4	3	9	1	4	5	0	0	0	42
d__Bacteria	k__norank_d__Bacteria	p__Actinobacteria	c__Actinobacteria	o__Acidimicrobiales	f__norank_o__Acidimicrobiales	g__norank_o__Acidimicrobiales	s__uncultured_Ferrimicrobium_sp._g__norank	OTU1440	5	10	2	13	6	4	6	3	2	6	7	4	4	13	5	0	5	5	100
d__Bacteria	k__norank_d__Bacteria	p__Actinobacteria	c__Actinobacteria	o__Propionibacteriales	f__Nocardioidaceae	g__Kribbella	s__uncultured_bacterium_g__Kribbella	OTU2609	2	2	11	1	0	0	3	5	2	0	0	3	0	5	1	0	0	4	39
d__Bacteria	k__norank_d__Bacteria	p__Proteobacteria	c__Alphaproteobacteria	o__Sphingomonadales	f__Sphingomonadaceae	g__Sphingomonas	s__unclassified_g__Sphingomonas	OTU1111	1	0	3	2	2	0	0	0	0	1	6	3	1	0	0	0	0	0	19
d__Bacteria	k__norank_d__Bacteria	p__Proteobacteria	c__Deltaproteobacteria	o__Oligoflexales	f__0319-6G20	g__norank_f__0319-6G20	s__unclassified_g__norank_f__0319-6G20	OTU781	0	0	2	0	0	0	0	0	0	0	0	0	1	0	0	0	0	0	3
d__Bacteria	k__norank_d__Bacteria	p__Acidobacteria	c__Acidobacteria	o__Acidobacteriales	f__Acidobacteriaceae__Subgroup_1_	g__norank_f__Acidobacteriaceae__Subgroup_1_	s__uncultured_Acidobacteriaceae_bacterium_g__norank_f__Acidobacteriaceae__Subgroup_1_	OTU6020	44	59	92	97	52	35	19	17	15	42	54	70	61	68	72	6	31	23	857
d__Bacteria	k__norank_d__Bacteria	p__unclassified_k__norank_d__Bacteria	c__unclassified_k__norank_d__Bacteria	o__unclassified_k__norank_d__Bacteria	f__unclassified_k__norank_d__Bacteria	g__unclassified_k__norank_d__Bacteria	s__unclassified_k__norank_d__Bacteria	OTU4884	0	0	0	0	0	0	0	0	0	0	0	0	0	2	0	0	0	0	2
d__Bacteria	k__norank_d__Bacteria	p__Proteobacteria	c__Gammaproteobacteria	o__Legionellales	f__Coxiellaceae	g__Aquicella	s__unclassified_g__Aquicella	OTU4915	0	1	1	0	0	0	0	0	0	0	0	0	0	1	0	0	0	0	3
d__Bacteria	k__norank_d__Bacteria	p__Parcubacteria	c__unclassified_p__Parcubacteria	o__unclassified_p__Parcubacteria	f__unclassified_p__Parcubacteria	g__unclassified_p__Parcubacteria	s__unclassified_p__Parcubacteria	OTU4959	6	3	0	4	0	1	0	0	0	2	0	0	0	2	0	0	0	0	18
d__Bacteria	k__norank_d__Bacteria	p__Proteobacteria	c__Alphaproteobacteria	o__Rhodospirillales	f__unclassified_o__Rhodospirillales	g__unclassified_o__Rhodospirillales	s__unclassified_o__Rhodospirillales	OTU1974	0	4	1	0	1	1	2	2	0	1	1	0	1	0	0	1	0	0	15
d__Bacteria	k__norank_d__Bacteria	p__Armatimonadetes	c__Chthonomonadetes	o__Chthonomonadales	f__norank_o__Chthonomonadales	g__norank_o__Chthonomonadales	s__unclassified_g__norank_o__Chthonomonadales	OTU4061	1	1	1	1	6	0	4	0	1	1	9	7	2	2	0	0	1	0	37
d__Bacteria	k__norank_d__Bacteria	p__Planctomycetes	c__Phycisphaerae	o__Tepidisphaerales	f__Tepidisphaeraceae	g__norank_f__Tepidisphaeraceae	s__unclassified_g__norank_f__Tepidisphaeraceae	OTU44	2	0	2	0	0	0	0	0	0	0	1	0	1	0	0	0	0	1	7
d__Bacteria	k__norank_d__Bacteria	p__Chloroflexi	c__Ktedonobacteria	o__B12-WMSP1	f__norank_o__B12-WMSP1	g__norank_o__B12-WMSP1	s__uncultured_bacterium_g__norank_o__B12-WMSP1	OTU865	0	0	1	0	0	0	0	0	0	0	1	0	0	0	0	0	0	0	2
d__Bacteria	k__norank_d__Bacteria	p__TM6__Dependentiae_	c__norank_p__TM6__Dependentiae_	o__norank_p__TM6__Dependentiae_	f__norank_p__TM6__Dependentiae_	g__norank_p__TM6__Dependentiae_	s__unclassified_g__norank_p__TM6__Dependentiae_	OTU3424	0	0	0	0	0	0	0	0	0	0	3	1	0	0	0	0	1	2	7
d__Bacteria	k__norank_d__Bacteria	p__Acidobacteria	c__Acidobacteria	o__Solibacterales	f__Solibacteraceae__Subgroup_3_	g__unclassified_f__Solibacteraceae__Subgroup_3_	s__unclassified_f__Solibacteraceae__Subgroup_3_	OTU1620	1	2	1	0	1	4	0	0	1	1	0	1	0	0	0	0	0	0	12
d__Bacteria	k__norank_d__Bacteria	p__Proteobacteria	c__Gammaproteobacteria	o__Xanthomonadales	f__Xanthomonadales_Incertae_Sedis	g__Steroidobacter	s__unclassified_g__Steroidobacter	OTU695	5	1	2	0	0	0	0	0	0	0	0	0	0	0	0	0	0	0	8
d__Bacteria	k__norank_d__Bacteria	p__Acidobacteria	c__Acidobacteria	o__Acidobacteriales	f__Acidobacteriaceae__Subgroup_1_	g__norank_f__Acidobacteriaceae__Subgroup_1_	s__unclassified_g__norank_f__Acidobacteriaceae__Subgroup_1_	OTU4678	0	0	0	0	0	0	0	0	0	0	0	0	3	0	0	0	0	0	3
[illegible]	[illegible]	[illegible]	[illegible]	[illegible]	[illegible]	[illegible]	[illegible]	[illegible]	[illegible]	[illegible]	[illegible]	[illegible]	[illegible]	[illegible]	[illegible]	[illegible]	[illegible]	[illegible]	[illegible]	[illegible]	[illegible]	[illegible]	[illegible]	[illegible]	[illegible]	[illegible]	[illegible]

d__Bacteria	k__norank_d__Bacteria	p__Acidobacteria	c__Acidobacteria	o__Blastocatellales	f__Blastocatellaceae__Subgroup_4_	g__norank_f__Blastocatellaceae__Subgroup_4_	s__unclassified_g__norank_f__Blastocatellaceae__Subgroup_4_	OTU1102	3	0	6	3	5	3	5	3	1	3	4	0	2	4	0	0	0	0	42
d__Bacteria	k__norank_d__Bacteria	p__Bacteroidetes	c__Sphingobacteriia	o__Sphingobacteriales	f__Chitinophagaceae	g__unclassified_f__Chitinophagaceae	s__unclassified_f__Chitinophagaceae	OTU3490	0	0	3	2	11	0	0	0	0	4	4	4	0	2	5	0	0	0	35
d__Bacteria	k__norank_d__Bacteria	p__Proteobacteria	c__Alphaproteobacteria	o__Sphingomonadales	f__Sphingomonadaceae	g__Sphingomonas	s__unclassified_g__Sphingomonas	OTU2427	21	33	68	0	4	10	81	25	37	7	1	4	70	32	24	29	26	68	540
d__Bacteria	k__norank_d__Bacteria	p__Chloroflexi	c__Ktedonobacteria	o__unclassified_c__Ktedonobacteria	f__unclassified_c__Ktedonobacteria	g__unclassified_c__Ktedonobacteria	s__unclassified_c__Ktedonobacteria	OTU135	6	0	0	0	0	0	0	0	0	0	0	0	0	0	0	0	0	0	6
d__Bacteria	k__norank_d__Bacteria	p__Planctomycetes	c__Phycisphaerae	o__Tepidisphaerales	f__Tepidisphaeraceae	g__norank_f__Tepidisphaeraceae	s__unclassified_g__norank_f__Tepidisphaeraceae	OTU5483	0	1	0	0	1	3	1	0	0	0	1	1	1	0	1	1	0	3	14
d__Bacteria	k__norank_d__Bacteria	p__Proteobacteria	c__Gammaproteobacteria	o__HTA4	f__norank_o__HTA4	g__norank_o__HTA4	s__uncultured_bacterium_g__norank_o__HTA4	OTU5727	1	1	2	0	3	0	4	1	0	4	0	4	10	0	2	4	1	1	38
d__Bacteria	k__norank_d__Bacteria	p__Nitrospirae	c__Nitrospira	o__norank_c__Nitrospira	f__norank_c__Nitrospira	g__Nitrospira	s__Nitrospira_japonica	OTU3928	231	268	209	281	281	224	53	45	75	301	328	194	270	231	293	6	64	25	3379
d__Bacteria	k__norank_d__Bacteria	p__Acidobacteria	c__Acidobacteria	o__Acidobacteriales	f__Acidobacteriaceae__Subgroup_1_	g__norank_f__Acidobacteriaceae__Subgroup_1_	s__unclassified_g__norank_f__Acidobacteriaceae__Subgroup_1_	OTU1929	21	33	29	14	35	14	9	29	9	19	32	23	31	9	33	1	6	8	355
d__Bacteria	k__norank_d__Bacteria	p__Acidobacteria	c__Acidobacteria	o__Solibacterales	f__Solibacteraceae__Subgroup_3_	g__Candidatus_Solibacter	s__unclassified_g__Candidatus_Solibacter	OTU4198	0	0	0	0	0	0	0	0	0	0	2	0	0	0	0	0	0	0	2
d__Bacteria	k__norank_d__Bacteria	p__Planctomycetes	c__Phycisphaerae	o__Tepidisphaerales	f__Tepidisphaeraceae	g__norank_f__Tepidisphaeraceae	s__uncultured_bacterium_g__norank_f__Tepidisphaeraceae	OTU3570	0	0	0	1	0	0	0	0	0	0	3	0	0	0	1	0	0	0	5
d__Bacteria	k__norank_d__Bacteria	p__Bacteroidetes	c__Sphingobacteriia	o__Sphingobacteriales	f__Chitinophagaceae	g__Chitinophaga	s__bacterium_14C13	OTU245	0	43	1	0	0	0	0	1	0	0	0	0	0	0	0	0	0	0	45
d__Bacteria	k__norank_d__Bacteria	p__Acidobacteria	c__Acidobacteria	o__norank_c__Acidobacteria	f__norank_c__Acidobacteria	g__norank_c__Acidobacteria	s__unclassified_g__norank_c__Acidobacteria	OTU2926	22	11	20	31	17	17	4	3	2	27	56	33	9	24	35	1	10	4	326
d__Bacteria	k__norank_d__Bacteria	p__Bacteroidetes	c__Sphingobacteriia	o__Sphingobacteriales	f__Chitinophagaceae	g__norank_f__Chitinophagaceae	s__unclassified_g__norank_f__Chitinophagaceae	OTU1402	0	0	0	0	1	0	0	0	0	0	0	0	8	0	0	0	0	0	9
d__Bacteria	k__norank_d__Bacteria	p__Firmicutes	c__Bacilli	o__Bacillales	f__Paenibacillaceae	g__Paenibacillus	s__Paenibacillus_telluris	OTU5854	54	0	11	1	1	0	5	24	6	0	2	0	1	0	1	0	85	9	200
d__Bacteria	k__norank_d__Bacteria	p__Chlamydiae	c__Chlamydiae	o__Chlamydiales	f__Parachlamydiaceae	g__unclassified_f__Parachlamydiaceae	s__unclassified_f__Parachlamydiaceae	OTU4245	0	0	1	3	1	0	1	4	2	0	4	0	3	0	0	0	0	0	19
d__Bacteria	k__norank_d__Bacteria	p__Chloroflexi	c__S085	o__norank_c__S085	f__norank_c__S085	g__norank_c__S085	s__uncultured_bacterium_SG4	OTU1076	0	1	0	4	3	4	0	2	0	0	0	0	0	0	2	0	1	0	17
d__Bacteria	k__norank_d__Bacteria	p__Elusimicrobia	c__Elusimicrobia	o__Lineage_IV	f__norank_o__Lineage_IV	g__norank_o__Lineage_IV	s__unclassified_g__norank_o__Lineage_IV	OTU1379	1	0	0	0	1	0	0	0	0	0	0	0	1	0	0	0	0	0	3
d__Bacteria	k__norank_d__Bacteria	p__Chloroflexi	c__JG37-AG-4	o__norank_c__JG37-AG-4	f__norank_c__JG37-AG-4	g__norank_c__JG37-AG-4	s__uncultured_bacterium_g__norank_c__JG37-AG-4	OTU4603	86	94	90	166	195	132	56	29	39	214	151	209	77	108	138	25	88	68	1965
d__Bacteria	k__norank_d__Bacteria	p__Acidobacteria	c__Acidobacteria	o__Acidobacteriales	f__Acidobacteriaceae__Subgroup_1_	g__unclassified_f__Acidobacteriaceae__Subgroup_1_	s__unclassified_f__Acidobacteriaceae__Subgroup_1_	OTU4907	0	0	0	0	0	0	0	0	0	1	0	0	0	1	0	0	0	0	2
d__Bacteria	k__norank_d__Bacteria	p__Elusimicrobia	c__Elusimicrobia	o__Lineage_IIa	f__norank_o__Lineage_IIa	g__norank_o__Lineage_IIa	s__uncultured_bacterium_g__norank_o__Lineage_IIa	OTU3384	6	4	6	13	9	12	0	0	0	13	13	8	9	10	15	0	0	0	118
d__Bacteria	k__norank_d__Bacteria	p__Proteobacteria	c__Gammaproteobacteria	o__unclassified_c__Gammaproteobacteria	f__unclassified_c__Gammaproteobacteria	g__unclassified_c__Gammaproteobacteria	s__unclassified_c__Gammaproteobacteria	OTU1413	0	0	1	0	2	0	0	0	0	0	0	0	0	0	1	0	0	0	4
d__Bacteria	k__norank_d__Bacteria	p__Chloroflexi	c__Ktedonobacteria	o__Ktedonobacterales	f__unclassified_o__Ktedonobacterales	g__unclassified_o__Ktedonobacterales	s__unclassified_o__Ktedonobacterales	OTU3508	0	0	1	1	1	0	1	0	1	2	1	1	0	0	0	1	1	0	11
d__Bacteria	k__norank_d__Bacteria	p__Chloroflexi	c__Ktedonobacteria	o__Ktedonobacterales	f__unclassified_o__Ktedonobacterales	g__unclassified_o__Ktedonobacterales	s__unclassified_o__Ktedonobacterales	OTU3502	5	6	7	2	7	17	6	1	2	4	17	9	8	6	8	1	6	5	117
d__Bacteria	k__norank_d__Bacteria	p__Acidobacteria	c__Acidobacteria	o__Acidobacteriales	f__Acidobacteriaceae__Subgroup_1_	g__norank_f__Acidobacteriaceae__Subgroup_1_	s__unclassified_g__norank_f__Acidobacteriaceae__Subgroup_1_	OTU5498	9	6	8	27	5	11	0	1	4	15	17	15	9	8	30	0	0	0	165
d__Bacteria	k__norank_d__Bacteria	p__TM6__Dependentiae_	c__norank_p__TM6__Dependentiae_	o__norank_p__TM6__Dependentiae_	f__norank_p__TM6__Dependentiae_	g__norank_p__TM6__Dependentiae_	s__unclassified_g__norank_p__TM6__Dependentiae_	OTU1828	0	0	0	0	0	2	0	0	0	0	0	0	0	0	0	0	0	0	2
d__Bacteria	k__norank_d__Bacteria	p__Actinobacteria	c__Actinobacteria	o__Acidimicrobiales	f__Iamiaceae	g__Iamia	s__uncultured_bacterium_g__Iamia	OTU2134	3	3	1	5	1	3	0	4	0	8	7	1	0	9	1	0	1	0	47
d__Bacteria	k__norank_d__Bacteria	p__Bacteroidetes	c__Sphingobacteriia	o__Sphingobacteriales	f__CWT_CU03-E12	g__norank_f__CWT_CU03-E12	s__unclassified_g__norank_f__CWT_CU03-E12	OTU5264	1	2	1	19	3	7	0	0	0	1	9	5	7	1	2	0	0	0	58
d__Bacteria	k__norank_d__Bacteria	p__Gemmatimonadetes	c__Gemmatimonadetes	o__Gemmatimonadales	f__Gemmatimonadaceae	g__norank_f__Gemmatimonadaceae	s__uncultured_bacterium_g__norank_f__Gemmatimonadaceae	OTU5285	5	17	11	3	0	0	0	0	0	3	0	0	5	7	1	0	0	0	52
d__Bacteria	k__norank_d__Bacteria	p__unclassified_k__norank_d__Bacteria	c__unclassified_k__norank_d__Bacteria	o__unclassified_k__norank_d__Bacteria	f__unclassified_k__norank_d__Bacteria	g__unclassified_k__norank_d__Bacteria	s__unclassified_k__norank_d__Bacteria	OTU3968	0	1	0	1	0	0	0	0	0	0	1	0	0	0	0	0	0	0	3
d__Bacteria	k__norank_d__Bacteria	p__unclassified_k__norank_d__Bacteria	c__unclassified_k__norank_d__Bacteria	o__unclassified_k__norank_d__Bacteria	f__unclassified_k__norank_d__Bacteria	g__unclassified_k__norank_d__Bacteria	s__unclassified_k__norank_d__Bacteria	OTU3966	0	0	0	0	0	1	0	0	0	0	2	1	0	0	0	0	0	0	4
d__Bacteria	k__norank_d__Bacteria	p__unclassified_k__norank_d__Bacteria	c__unclassified_k__norank_d__Bacteria	o__unclassified_k__norank_d__Bacteria	f__unclassified_k__norank_d__Bacteria	g__unclassified_k__norank_d__Bacteria	s__unclassified_k__norank_d__Bacteria	OTU3967	0	0	0	0	0	0	0	0	0	0	2	0	0	0	0	0	0	0	2
d__Bacteria	k__norank_d__Bacteria	p__Acidobacteria	c__Acidobacteria	o__Acidobacteriales	f__Acidobacteriaceae__Subgroup_1_	g__Candidatus_Koribacter	s__uncultured_Acidobacteria_bacterium_g__Candidatus_Koribacter	OTU2084	3	10	4	4	0	2	7	7	1	2	6	4	2	3	2	1	3	0	61
d__Bacteria	k__norank_d__Bacteria	p__Proteobacteria	c__Deltaproteobacteria	o__Oligoflexales	f__0319-6G20	g__norank_f__0319-6G20	s__bacterium_enrichment_culture_clone_auto112_4W	OTU473	2	6	0	2	4	3	0	0	0	1	2	0	0	2	0	0	0	0	22
d__Bacteria	k__norank_d__Bacteria	p__Bacteroidetes	c__Sphingobacteriia	o__Sphingobacteriales	f__Chitinophagaceae	g__Flavitalea	s__unclassified_g__Flavitalea	OTU141	10	0	9	0	0	0	1	0	0	1	0	0	0	0	1	0	1	0	23
d__Bacteria	k__norank_d__Bacteria	p__Firmicutes	c__Bacilli	o__Bacillales	f__Alicyclobacillaceae	g__unclassified_f__Alicyclobacillaceae	s__unclassified_f__Alicyclobacillaceae	OTU1246	0	0	0	1	0	0	0	0	0	0	0	0	1	0	1	0	0	0	3
[illegible]																											

d__Bacteria	k__norank_d__Bacteria	p__Cyanobacteria	c__Cyanobacteria	o__norank_c__Cyanobacteria	[illegible]
d__Bacteria	k__norank_d__Bacteria	p__Planctomycetes	c__Planctomycetacia	o__Planctomycetales	[illegible]
d__Bacteria	k__norank_d__Bacteria	p__Chloroflexi	c__Ktedonobacteria	o__Ktedonobacterales	[illegible]
d__Bacteria	k__norank_d__Bacteria	p__Latescibacteria	c__norank_p__Latescibacteria	o__norank_p__Latescibacteria	[illegible]
d__Bacteria	k__norank_d__Bacteria	p__Chloroflexi	c__Ktedonobacteria	o__JG30-KF-AS9	[illegible]
d__Bacteria	k__norank_d__Bacteria	p__Proteobacteria	c__Alphaproteobacteria	o__Rhodospirillales	[illegible]
d__Bacteria	k__norank_d__Bacteria	p__Firmicutes	c__Bacilli	o__Bacillales	[illegible]
d__Bacteria	k__norank_d__Bacteria	p__Proteobacteria	c__Alphaproteobacteria	o__Rickettsiales	[illegible]
d__Bacteria	k__norank_d__Bacteria	p__Gemmatimonadetes	c__Gemmatimonadetes	o__Gemmatimonadales	[illegible]
d__Bacteria	k__norank_d__Bacteria	p__Acidobacteria	c__Acidobacteria	o__Acidobacteriales	[illegible]
d__Bacteria	k__norank_d__Bacteria	p__Proteobacteria	c__Gammaproteobacteria	o__Xanthomonadales	[illegible]
d__Bacteria	k__norank_d__Bacteria	p__Planctomycetes	c__Planctomycetacia	o__Planctomycetales	[illegible]
d__Bacteria	k__norank_d__Bacteria	p__Chloroflexi	c__Ktedonobacteria	o__Thermogemmatisporales	[illegible]
d__Bacteria	k__norank_d__Bacteria	p__Chloroflexi	c__Ktedonobacteria	o__Thermogemmatisporales	[illegible]
d__Bacteria	k__norank_d__Bacteria	p__Proteobacteria	c__Gammaproteobacteria	o__unclassified_c__Gammaproteobacteria	[illegible]
d__Bacteria	k__norank_d__Bacteria	p__Proteobacteria	c__Gammaproteobacteria	o__unclassified_c__Gammaproteobacteria	[illegible]
d__Bacteria	k__norank_d__Bacteria	p__Proteobacteria	c__Deltaproteobacteria	o__Oligoflexales	[illegible]
d__Bacteria	k__norank_d__Bacteria	p__Proteobacteria	c__Alphaproteobacteria	o__Rhodospirillales	[illegible]
d__Bacteria	k__norank_d__Bacteria	p__Proteobacteria	c__Gammaproteobacteria	o__Legionellales	[illegible]
d__Bacteria	k__norank_d__Bacteria	p__Verrucomicrobia	c__Spartobacteria	o__Chthoniobacterales	[illegible]
d__Bacteria	k__norank_d__Bacteria	p__Cyanobacteria	c__Cyanobacteria	o__SubsectionIII	[illegible]
d__Bacteria	k__norank_d__Bacteria	p__Proteobacteria	c__Betaproteobacteria	o__TRA3-20	[illegible]
d__Bacteria	k__norank_d__Bacteria	p__Firmicutes	c__Clostridia	o__Clostridiales	[illegible]
d__Bacteria	k__norank_d__Bacteria	p__Acidobacteria	c__Acidobacteria	o__Acidobacteriales	[illegible]
d__Bacteria	k__norank_d__Bacteria	p__Firmicutes	c__Clostridia	o__Clostridiales	[illegible]
d__Bacteria	k__norank_d__Bacteria	p__Chloroflexi	c__Ktedonobacteria	o__unclassified_c__Ktedonobacteria	[illegible]
d__Bacteria	k__norank_d__Bacteria	p__Chlamydiae	c__Chlamydiae	o__Chlamydiales	[illegible]
d__Bacteria	k__norank_d__Bacteria	p__Planctomycetes	c__Planctomycetacia	o__Planctomycetales	[illegible]
d__Bacteria	k__norank_d__Bacteria	p__Chloroflexi	c__Ktedonobacteria	o__Ktedonobacterales	[illegible]
d__Bacteria	k__norank_d__Bacteria	p__Chloroflexi	c__Ktedonobacteria	o__C0119	[illegible]
d__Bacteria	k__norank_d__Bacteria	p__Chloroflexi	c__Ktedonobacteria	o__Ktedonobacterales	[illegible]
d__Bacteria	k__norank_d__Bacteria	p__Proteobacteria	c__Gammaproteobacteria	o__Legionellales	[illegible]
d__Bacteria	k__norank_d__Bacteria	p__Proteobacteria	c__Gammaproteobacteria	o__Legionellales	[illegible]
d__Bacteria	k__norank_d__Bacteria	p__Proteobacteria	c__Gammaproteobacteria	o__Legionellales	[illegible]
[illegible]	[illegible]	[illegible]	[illegible]	[illegible]	[illegible]

d__Bacteria	k__norank_d__Bacteria	p__Proteobacteria	c__Gammaproteobacteria	o__Thiotrichales	f__CHAB-XI-27	g__norank_f__CHAB-XI-27	s__uncultured_bacterium_g__norank_f__CHAB-XI-27	OTU4305	0	0	0	0	0	0	0	0	0	0	1	0	1	0	0	0	0	0	2
d__Bacteria	k__norank_d__Bacteria	p__Proteobacteria	c__Deltaproteobacteria	o__Myxococcales	f__unclassified_o__Myxococcales	g__unclassified_o__Myxococcales	s__unclassified_o__Myxococcales	OTU4752	0	0	0	0	0	0	0	0	0	0	0	0	0	1	0	0	0	0	1
d__Bacteria	k__norank_d__Bacteria	p__Bacteroidetes	c__Bacteroidia	o__Bacteroidales	f__Bacteroidaceae	g__Bacteroides	s__unclassified_g__Bacteroides	OTU3701	1	2	0	0	0	1	0	5	1	1	3	0	3	0	1	0	1	3	22
d__Bacteria	k__norank_d__Bacteria	p__Chloroflexi	c__Ktedonobacteria	o__Ktedonobacterales	f__unclassified_o__Ktedonobacterales	g__unclassified_o__Ktedonobacterales	s__unclassified_o__Ktedonobacterales	OTU3008	5	0	1	3	4	6	0	1	0	2	8	1	1	2	0	0	0	0	34
d__Bacteria	k__norank_d__Bacteria	p__Proteobacteria	c__Gammaproteobacteria	o__Xanthomonadales	f__Xanthomonadaceae	g__Panacagrimonas	s__uncultured_bacterium_g__Panacagrimonas	OTU4970	0	0	0	0	0	0	0	0	0	0	0	0	0	2	0	0	0	0	2
d__Bacteria	k__norank_d__Bacteria	p__Proteobacteria	c__Gammaproteobacteria	o__Enterobacteriales	f__Enterobacteriaceae	g__unclassified_f__Enterobacteriaceae	s__unclassified_f__Enterobacteriaceae	OTU5787	0	0	0	0	0	0	0	0	0	0	0	0	0	0	0	2	0	0	2
d__Bacteria	k__norank_d__Bacteria	p__unclassified_k__norank_d__Bacteria	c__unclassified_k__norank_d__Bacteria	o__unclassified_k__norank_d__Bacteria	f__unclassified_k__norank_d__Bacteria	g__unclassified_k__norank_d__Bacteria	s__unclassified_k__norank_d__Bacteria	OTU136	2	0	1	0	0	0	0	0	0	1	0	0	0	0	2	0	0	0	6
d__Bacteria	k__norank_d__Bacteria	p__unclassified_k__norank_d__Bacteria	c__unclassified_k__norank_d__Bacteria	o__unclassified_k__norank_d__Bacteria	f__unclassified_k__norank_d__Bacteria	g__unclassified_k__norank_d__Bacteria	s__unclassified_k__norank_d__Bacteria	OTU134	2	0	0	0	0	0	0	0	0	0	0	0	0	0	0	0	0	0	2
d__Bacteria	k__norank_d__Bacteria	p__Acidobacteria	c__Acidobacteria	o__Solibacterales	f__Solibacteraceae__Subgroup_3_	g__Candidatus_Solibacter	s__unclassified_g__Candidatus_Solibacter	OTU4266	105	124	120	162	172	157	30	30	31	145	221	209	84	127	120	7	38	41	1923
d__Bacteria	k__norank_d__Bacteria	p__Proteobacteria	c__Alphaproteobacteria	o__Rhizobiales	f__Hyphomicrobiaceae	g__Rhodomicrobium	s__unclassified_g__Rhodomicrobium	OTU2986	5	9	9	11	1	0	3	2	3	5	10	2	4	3	4	0	3	2	76
d__Bacteria	k__norank_d__Bacteria	p__unclassified_k__norank_d__Bacteria	c__unclassified_k__norank_d__Bacteria	o__unclassified_k__norank_d__Bacteria	f__unclassified_k__norank_d__Bacteria	g__unclassified_k__norank_d__Bacteria	s__unclassified_k__norank_d__Bacteria	OTU138	2	0	0	0	0	0	0	0	0	0	0	0	0	0	0	0	0	0	2
d__Bacteria	k__norank_d__Bacteria	p__Proteobacteria	c__Gammaproteobacteria	o__Oceanospirillales	f__SAR86_clade	g__norank_f__SAR86_clade	s__unclassified_g__norank_f__SAR86_clade	OTU1030	0	0	0	2	0	0	0	0	0	0	0	0	0	0	0	0	0	0	2
d__Bacteria	k__norank_d__Bacteria	p__Proteobacteria	c__Deltaproteobacteria	o__Oligoflexales	f__0319-6G20	g__norank_f__0319-6G20	s__unclassified_g__norank_f__0319-6G20	OTU5248	0	2	1	0	5	0	0	0	0	0	0	0	5	0	1	0	0	0	14
d__Bacteria	k__norank_d__Bacteria	p__TM6__Dependentiae_	c__norank_p__TM6__Dependentiae_	o__norank_p__TM6__Dependentiae_	f__norank_p__TM6__Dependentiae_	g__norank_p__TM6__Dependentiae_	s__uncultured_bacterium_g__norank_p__TM6__Dependentiae_	OTU4550	0	0	0	0	0	0	0	0	0	0	0	0	15	0	0	3	0	0	18
d__Bacteria	k__norank_d__Bacteria	p__Proteobacteria	c__Gammaproteobacteria	o__Legionellales	f__Coxiellaceae	g__norank_f__Coxiellaceae	s__uncultured_Coxiellaceae_bacterium_g__norank	OTU2623	0	0	0	0	0	0	0	0	2	0	0	0	0	0	0	0	0	0	2
d__Bacteria	k__norank_d__Bacteria	p__Proteobacteria	c__Gammaproteobacteria	o__Xanthomonadales	f__norank_o__Xanthomonadales	g__norank_o__Xanthomonadales	s__unclassified_g__norank_o__Xanthomonadales	OTU1050	0	0	0	2	0	0	0	0	0	0	0	0	0	0	0	0	0	0	2
d__Bacteria	k__norank_d__Bacteria	p__Proteobacteria	c__Deltaproteobacteria	o__Myxococcales	f__norank_o__Myxococcales	g__norank_o__Myxococcales	s__uncultured_bacterium_g__norank_o__Myxococcales	OTU1051	0	0	0	1	1	0	0	0	0	0	1	3	0	0	0	0	0	0	6
d__Bacteria	k__norank_d__Bacteria	p__Elusimicrobia	c__Elusimicrobia	o__Lineage_IV	f__norank_o__Lineage_IV	g__norank_o__Lineage_IV	s__unclassified_g__norank_o__Lineage_IV	OTU4787	2	2	0	1	0	1	0	0	0	2	1	1	3	2	0	0	0	0	15
d__Bacteria	k__norank_d__Bacteria	p__Chloroflexi	c__P2-11E	o__norank_c__P2-11E	f__norank_c__P2-11E	g__norank_c__P2-11E	s__uncultured_bacterium_g__norank_c__P2-11E	OTU3556	0	0	0	0	0	0	0	1	0	0	1	2	0	2	0	0	0	0	6
d__Bacteria	k__norank_d__Bacteria	p__Acidobacteria	c__Acidobacteria	o__norank_c__Acidobacteria	f__norank_c__Acidobacteria	g__norank_c__Acidobacteria	s__uncultured_Acidobacteriales_bacterium_g__norank_c__Acidobacteria	OTU5346	1	6	7	2	1	0	1	1	0	1	3	2	3	4	4	1	2	3	42
d__Bacteria	k__norank_d__Bacteria	p__Chloroflexi	c__Ktedonobacteria	o__C0119	f__norank_o__C0119	g__norank_o__C0119	s__uncultured_bacterium_g__norank_o__C0119	OTU3978	1	2	1	0	0	3	4	0	0	1	1	1	0	0	0	0	0	0	14
d__Bacteria	k__norank_d__Bacteria	p__Acidobacteria	c__Acidobacteria	o__norank_c__Acidobacteria	f__norank_c__Acidobacteria	g__norank_c__Acidobacteria	s__uncultured_Acidobacteriales_bacterium_g__norank_c__Acidobacteria	OTU5342	0	1	4	3	5	1	2	4	1	1	1	2	0	6	1	0	1	0	33
d__Bacteria	k__norank_d__Bacteria	p__Proteobacteria	c__Gammaproteobacteria	o__unclassified_c__Gammaproteobacteria	f__unclassified_c__Gammaproteobacteria	g__unclassified_c__Gammaproteobacteria	s__unclassified_c__Gammaproteobacteria	OTU1363	3	3	0	2	0	0	0	0	0	1	2	0	0	0	1	0	0	3	15
d__Bacteria	k__norank_d__Bacteria	p__Proteobacteria	c__Alphaproteobacteria	o__Rhodospirillales	f__Acetobacteraceae	g__unclassified_f__Acetobacteraceae	s__unclassified_f__Acetobacteraceae	OTU3739	13	25	24	100	55	30	7	8	8	64	55	64	28	38	29	0	8	8	564
d__Bacteria	k__norank_d__Bacteria	p__Proteobacteria	c__Gammaproteobacteria	o__unclassified_c__Gammaproteobacteria	f__unclassified_c__Gammaproteobacteria	g__unclassified_c__Gammaproteobacteria	s__unclassified_c__Gammaproteobacteria	OTU1496	0	1	0	0	1	0	0	0	0	0	0	0	0	0	0	0	0	0	2
d__Bacteria	k__norank_d__Bacteria	p__Proteobacteria	c__Alphaproteobacteria	o__Sphingomonadales	f__Sphingomonadaceae	g__Sphingomonas	s__uncultured_Sphingomonadaceae_bacterium_g__Sphingomonas	OTU5096	41	29	57	29	29	32	35	5	36	34	31	23	54	110	43	13	64	47	712
d__Bacteria	k__norank_d__Bacteria	p__Planctomycetes	c__Planctomycetacia	o__Planctomycetales	f__Planctomycetaceae	g__norank_f__Planctomycetaceae	s__unclassified_g__norank_f__Planctomycetaceae	OTU2898	0	1	0	4	11	7	0	0	2	7	12	15	0	2	1	0	1	2	65
d__Bacteria	k__norank_d__Bacteria	p__Acidobacteria	c__Acidobacteria	o__Acidobacteriales	f__Acidobacteriaceae__Subgroup_1_	g__unclassified_f__Acidobacteriaceae__Subgroup_1_	s__unclassified_f__Acidobacteriaceae__Subgroup_1_	OTU1769	5	6	10	2	13	8	2	2	1	5	3	3	15	5	11	0	7	9	107
d__Bacteria	k__norank_d__Bacteria	p__Elusimicrobia	c__Elusimicrobia	o__Lineage_IIb	f__norank_o__Lineage_IIb	g__norank_o__Lineage_IIb	s__uncultured_bacterium_g__norank_o__Lineage_IIb	OTU4833	3	1	0	0	2	1	0	0	0	0	0	0	0	2	0	0	0	0	9
d__Bacteria	k__norank_d__Bacteria	p__Bacteroidetes	c__Sphingobacteriia	o__Sphingobacteriales	f__Chitinophagaceae	g__norank_f__Chitinophagaceae	s__unclassified_g__norank_f__Chitinophagaceae	OTU5723	0	1	0	2	0	2	0	0	0	0	0	1	0	0	0	1	0	2	9
d__Bacteria	k__norank_d__Bacteria	p__unclassified_k__norank_d__Bacteria	c__unclassified_k__norank_d__Bacteria	o__unclassified_k__norank_d__Bacteria	f__unclassified_k__norank_d__Bacteria	g__unclassified_k__norank_d__Bacteria	s__unclassified_k__norank_d__Bacteria	OTU1656	0	1	3	2	1	0	2	5	0	0	0	3	3	1	0	0	1	0	22
d__Bacteria	k__norank_d__Bacteria	p__Proteobacteria	c__Alphaproteobacteria	o__Rickettsiales	f__Mitochondria	g__norank_f__Mitochondria	s__unclassified_g__norank_f__Mitochondria	OTU215	8	139	88	29	34	7	11	14	4	8	3	3	88	114	40	2	8	2	602
d__Bacteria	k__norank_d__Bacteria	p__Firmicutes	c__Clostridia	o__Clostridiales	f__Peptostreptococcaceae	g__Sporacetigenium	s__uncultured_Firmicutes_bacterium_g__Sporacetigenium	OTU125	1	0	0	0	0	0	0	0	0	0	0	0	0	0	0	0	0	0	1
d__Bacteria	k__norank_d__Bacteria	p__Bacteroidetes	c__Cytophagia	o__Cytophagales	f__Cytophagaceae	g__norank_f__Cytophagaceae	s__uncultured_Cytophagaceae_bacterium_g__norank	OTU194	6	6	0	0	0	0	0	0	0	0	0	0	1	0	0	0	0	0	13
d__Bacteria	k__norank_d__Bacteria	p__Bacteroidetes	c__Sphingobacteriia	o__Sphingobacteriales	f__Sphingobacteriaceae	g__unclassified_f__Sphingobacteriaceae	s__unclassified_f__Sphingobacteriaceae	OTU5830	2	6	4	0	0	0	0	0	0	0	0	0	2	1	1	0	0	0	16
[illegible]																										

d__Bacteria	k__norank_d__Bacteria	p__Proteobacteria	c__Deltaproteobacteria	o__Myxococcales	f__Polyangiaceae	g__Polyangium	s__uncultured_bacterium_g__Polyangium	OTU318	2	3	10	0	2	3	2	2	1	0	0	0	4	0	0	0	0	1	30
d__Bacteria	k__norank_d__Bacteria	p__Armatimonadetes	c__Armatimonadia	o__Armatimonadales	f__norank_o__Armatimonadales	g__norank_o__Armatimonadales	s__unclassified_g__norank_o__Armatimonadales	OTU1520	0	0	0	0	3	0	0	0	0	0	0	0	0	0	0	0	0	0	3
d__Bacteria	k__norank_d__Bacteria	p__Chloroflexi	c__Ktedonobacteria	o__B12-WMSP1	f__norank_o__B12-WMSP1	g__norank_o__B12-WMSP1	s__uncultured_bacterium_g__norank_o__B12-WMSP1	OTU1553	1	1	0	0	2	2	0	2	1	1	1	1	2	0	2	1	0	0	17
d__Bacteria	k__norank_d__Bacteria	p__Chloroflexi	c__Ktedonobacteria	o__Ktedonobacterales	f__unclassified_o__Ktedonobacterales	g__unclassified_o__Ktedonobacterales	s__unclassified_o__Ktedonobacterales	OTU5221	0	3	0	2	3	1	2	0	0	2	0	1	0	0	2	1	2	0	19
d__Bacteria	k__norank_d__Bacteria	p__unclassified_k__norank_d__Bacteria	c__unclassified_k__norank_d__Bacteria	o__unclassified_k__norank_d__Bacteria	f__unclassified_k__norank_d__Bacteria	g__unclassified_k__norank_d__Bacteria	s__unclassified_k__norank_d__Bacteria	OTU2649	0	1	0	0	0	0	0	0	0	2	0	0	0	0	0	0	0	0	3
d__Bacteria	k__norank_d__Bacteria	p__Proteobacteria	c__Alphaproteobacteria	o__Rickettsiales	f__norank_o__Rickettsiales	g__norank_o__Rickettsiales	s__uncultured_Acanthamoeba	OTU1416	0	0	0	0	2	0	0	0	0	0	0	0	0	1	0	0	0	0	3
d__Bacteria	k__norank_d__Bacteria	p__unclassified_k__norank_d__Bacteria	c__unclassified_k__norank_d__Bacteria	o__unclassified_k__norank_d__Bacteria	f__unclassified_k__norank_d__Bacteria	g__unclassified_k__norank_d__Bacteria	s__unclassified_k__norank_d__Bacteria	OTU2138	0	0	0	0	0	0	0	3	0	0	0	0	2	0	0	0	0	0	5
d__Bacteria	k__norank_d__Bacteria	p__unclassified_k__norank_d__Bacteria	c__unclassified_k__norank_d__Bacteria	o__unclassified_k__norank_d__Bacteria	f__unclassified_k__norank_d__Bacteria	g__unclassified_k__norank_d__Bacteria	s__unclassified_k__norank_d__Bacteria	OTU2136	0	0	0	0	0	0	0	2	0	0	0	0	0	0	0	0	0	0	2
d__Bacteria	k__norank_d__Bacteria	p__Proteobacteria	c__Gammaproteobacteria	o__Legionellales	f__Coxiellaceae	g__Aquicella	s__bacterium_enrichment_culture_clone_B61_2011_	OTU3742	0	0	0	0	0	2	0	0	0	0	2	1	1	1	2	0	1	0	10
d__Bacteria	k__norank_d__Bacteria	p__unclassified_k__norank_d__Bacteria	c__unclassified_k__norank_d__Bacteria	o__unclassified_k__norank_d__Bacteria	f__unclassified_k__norank_d__Bacteria	g__unclassified_k__norank_d__Bacteria	s__unclassified_k__norank_d__Bacteria	OTU2644	0	0	0	0	0	0	0	0	0	1	0	0	0	0	0	0	0	0	1
d__Bacteria	k__norank_d__Bacteria	p__Proteobacteria	c__Alphaproteobacteria	o__Rhodospirillales	f__DA111	g__norank_f__DA111	s__unclassified_g__norank_f__DA111	OTU918	0	0	0	1	0	0	0	0	0	0	0	0	0	0	0	0	1	0	2
d__Bacteria	k__norank_d__Bacteria	p__unclassified_k__norank_d__Bacteria	c__unclassified_k__norank_d__Bacteria	o__unclassified_k__norank_d__Bacteria	f__unclassified_k__norank_d__Bacteria	g__unclassified_k__norank_d__Bacteria	s__unclassified_k__norank_d__Bacteria	OTU2646	3	8	1	2	1	3	0	0	0	4	3	12	2	2	3	0	0	0	44
d__Bacteria	k__norank_d__Bacteria	p__Proteobacteria	c__Alphaproteobacteria	o__Rhodospirillales	f__Acetobacteraceae	g__unclassified_f__Acetobacteraceae	s__unclassified_f__Acetobacteraceae	OTU5608	2	1	0	0	0	0	0	2	0	0	0	2	1	0	0	0	2	0	10
d__Bacteria	k__norank_d__Bacteria	p__Proteobacteria	c__Deltaproteobacteria	o__Bdellovibrionales	f__Bdellovibrionaceae	g__Bdellovibrio	s__unclassified_g__Bdellovibrio	OTU2335	0	2	0	0	0	0	0	0	1	0	0	0	0	1	0	0	0	0	4
d__Bacteria	k__norank_d__Bacteria	p__Bacteroidetes	c__Sphingobacteriia	o__Sphingobacteriales	f__Chitinophagaceae	g__norank_f__Chitinophagaceae	s__unclassified_g__norank_f__Chitinophagaceae	OTU3311	3	5	14	0	13	33	4	2	0	4	16	4	15	2	10	0	0	3	128
d__Bacteria	k__norank_d__Bacteria	p__Chlamydiae	c__Chlamydiae	o__Chlamydiales	f__cvE6	g__norank_f__cvE6	s__uncultured_bacterium_g__norank_f__cvE6	OTU4271	6	2	3	21	19	5	4	2	4	10	19	47	5	10	29	0	5	6	197
d__Bacteria	k__norank_d__Bacteria	p__unclassified_k__norank_d__Bacteria	c__unclassified_k__norank_d__Bacteria	o__unclassified_k__norank_d__Bacteria	f__unclassified_k__norank_d__Bacteria	g__unclassified_k__norank_d__Bacteria	s__unclassified_k__norank_d__Bacteria	OTU4998	0	0	1	0	0	0	0	0	0	0	0	0	0	1	0	0	0	0	2
d__Bacteria	k__norank_d__Bacteria	p__Proteobacteria	c__Gammaproteobacteria	o__unclassified_c__Gammaproteobacteria	f__unclassified_c__Gammaproteobacteria	g__unclassified_c__Gammaproteobacteria	s__unclassified_c__Gammaproteobacteria	OTU749	0	0	2	0	0	0	0	0	0	0	0	0	0	0	0	0	0	0	2
d__Bacteria	k__norank_d__Bacteria	p__Proteobacteria	c__Gammaproteobacteria	o__Legionellales	f__Coxiellaceae	g__norank_f__Coxiellaceae	s__unclassified_g__norank_f__Coxiellaceae	OTU4985	2	0	3	2	3	4	0	0	0	1	1	0	3	4	0	2	1	0	26
d__Bacteria	k__norank_d__Bacteria	p__Verrucomicrobia	c__OPB35_soil_group	o__norank_c__OPB35_soil_group	f__norank_c__OPB35_soil_group	g__norank_c__OPB35_soil_group	s__uncultured_Verrucomicrobia_subdivision_3_bacterium	OTU1201	52	62	61	61	31	39	6	4	4	24	156	19	37	63	41	5	2	7	674
d__Bacteria	k__norank_d__Bacteria	p__Proteobacteria	c__Betaproteobacteria	o__Burkholderiales	f__Oxalobacteraceae	g__Paucimonas	s__unclassified_g__Paucimonas	OTU2540	10	58	17	5	2	0	3	13	1	1	1	3	2	6	1	0	7	1	131
d__Bacteria	k__norank_d__Bacteria	p__Firmicutes	c__Bacilli	o__Bacillales	f__Paenibacillaceae	g__Paenibacillus	s__unclassified_g__Paenibacillus	OTU5099	0	0	0	0	0	1	0	0	0	1	0	0	0	4	0	0	0	0	6
d__Bacteria	k__norank_d__Bacteria	p__Chloroflexi	c__Ktedonobacteria	o__Ktedonobacterales	f__unclassified_o__Ktedonobacterales	g__unclassified_o__Ktedonobacterales	s__unclassified_o__Ktedonobacterales	OTU2252	17	12	25	11	16	31	7	2	5	12	14	16	11	17	22	0	8	4	230
d__Bacteria	k__norank_d__Bacteria	p__Proteobacteria	c__Deltaproteobacteria	o__Bdellovibrionales	f__Bdellovibrionaceae	g__Bdellovibrio	s__unclassified_g__Bdellovibrio	OTU4485	0	0	0	0	0	0	2	0	0	0	0	2	0	0	0	0	0	0	4
d__Bacteria	k__norank_d__Bacteria	p__Proteobacteria	c__Deltaproteobacteria	o__Oligoflexales	f__0319-6G20	g__norank_f__0319-6G20	s__unclassified_g__norank_f__0319-6G20	OTU1472	0	0	0	0	2	0	0	0	0	0	0	0	0	0	0	0	0	0	2
d__Bacteria	k__norank_d__Bacteria	p__Proteobacteria	c__Gammaproteobacteria	o__Xanthomonadales	f__Nevskiaceae	g__Nevskia	s__unclassified_g__Nevskia	OTU2909	0	3	1	1	0	0	0	0	0	5	1	0	2	0	0	0	0	0	13
d__Bacteria	k__norank_d__Bacteria	p__Chloroflexi	c__Ktedonobacteria	o__Ktedonobacterales	f__1921-3	g__norank_f__1921-3	s__uncultured_bacterium_g__norank_f__1921-3	OTU4258	0	2	2	2	1	2	0	0	0	0	4	0	0	0	1	0	0	0	14
d__Bacteria	k__norank_d__Bacteria	p__Armatimonadetes	c__Chthonomonadetes	o__Chthonomonadales	f__Chthonomonadaceae	g__Chthonomonas	s__uncultured_bacterium_g__Chthonomonas	OTU1303	0	0	0	1	0	0	0	0	0	0	0	0	0	0	3	0	0	0	4
d__Bacteria	k__norank_d__Bacteria	p__Armatimonadetes	c__Chthonomonadetes	o__Chthonomonadales	f__Chthonomonadaceae	g__Chthonomonas	s__uncultured_bacterium_g__Chthonomonas	OTU1300	0	0	0	2	1	0	0	0	0	0	1	2	0	0	0	0	0	0	6
d__Bacteria	k__norank_d__Bacteria	p__Firmicutes	c__Erysipelotrichia	o__Erysipelotrichales	f__Erysipelotrichaceae	g__unclassified_f__Erysipelotrichaceae	s__unclassified_f__Erysipelotrichaceae	OTU2807	0	0	0	0	0	0	0	0	0	1	0	0	0	0	0	0	0	0	1
d__Bacteria	k__norank_d__Bacteria	p__Firmicutes	c__Clostridia	o__Clostridiales	f__Clostridiaceae_1	g__Oxobacter	s__uncultured_bacterium_g__Oxobacter	OTU1895	0	0	0	0	0	0	2	0	0	1	0	1	0	0	1	0	1	0	6
d__Bacteria	k__norank_d__Bacteria	p__Latescibacteria	c__norank_p__Latescibacteria	o__norank_p__Latescibacteria	f__norank_p__Latescibacteria	g__norank_p__Latescibacteria	s__unclassified_g__norank_p__Latescibacteria	OTU1000	0	0	0	2	0	0	0	0	0	0	0	0	0	0	0	0	0	0	2
d__Bacteria	k__norank_d__Bacteria	p__Firmicutes	c__Bacilli	o__Bacillales	f__unclassified_o__Bacillales	g__unclassified_o__Bacillales	s__unclassified_o__Bacillales	OTU2075	0	0	0	0	0	0	2	0	0	0	0	0	0	0	0	0	0	0	2
d__Bacteria	k__norank_d__Bacteria	p__Bacteroidetes	c__Sphingobacteriia	o__Sphingobacteriales	f__Chitinophagaceae	g__norank_f__Chitinophagaceae	s__unclassified_g__norank_f__Chitinophagaceae	OTU3222	3	1	3	16	17	12	2	0	0	2	8	0	2	1	3	0	0	0	70
d__Bacteria	k__norank_d__Bacteria	p__Acidobacteria	c__Acidobacteria	o__Blastocatellales	f__Blastocatellaceae__Subgroup_4_	g__11-24	s__unclassified_g__11-24	OTU1544	0	0	0	0	3	0	0	0	0	0	0	0	0	0	0	0	0	0	3
d__Bacteria	k__norank_d__Bacteria	p__Proteobacteria	c__Betaproteobacteria	o__unclassified_c__Betaproteobacteria	f__unclassified_c__Betaproteobacteria	g__unclassified_c__Betaproteobacteria	s__unclassified_c__Betaproteobacteria	OTU4733	0	0	0	0	0	0	0	0	0	0	0	0	2	0	0	0	0	0	2
d__Bacteria	k__norank_d__Bacteria	p__Actinobacteria	c__Actinobacteria	o__Pseudonocardiales	f__Pseudonocardiaceae	g__unclassified_f__Pseudonocardiaceae	s__unclassified_f__Pseudonocardiaceae	OTU1180	0	0	0	6	0	0	0	0	0	1	0	0	0	0	0	0	0	0	7
d__Bacteria	k__norank_d__Bacteria	p__Proteobacteria	c__Deltaproteobacteria	o__Myxococcales	f__Haliangiaceae	g__Haliangium	s__unclassified_g__Haliangium	OTU3306	36	23	20	40	45	57	13	6	8	24	12	27	14	37	14	1	5	4	386
d__Bacteria	k__norank_d__Bacteria	p__unclassified_k__norank_d__Bacteria	c__unclassified_k__norank_d__Bacteria	o__unclassified_k__norank_d__Bacteria	f__unclassified_k__norank_d__Bacteria	g__unclassified_k__norank_d__Bacteria	s__unclassified_k__norank_d__Bacteria	OTU5642	0	0	0	0	0	2	0	0	0	0	0	0	0	0	2	0	0	0	4
d__Bacteria	k__norank_d__Bacteria	p__Bacteroidetes	c__Sphingobacteriia	o__Sphingobacteriales	f__Sphingobacteriaceae	g__Mucilaginibacter	s__unclassified_g__Mucilaginibacter	OTU593	2	11	23	0	0	0	0	2	4	0	0	0	32	7	6	0	0	1	88
d__Bacteria	k__norank_d__Bacteria	p__Planctomycetes	c__Planctomycetacia	o__Planctomycetales	f__Planctomycetaceae	g__unclassified_f__Planctomycetaceae	s__unclassified_f__Planctomycetaceae	OTU4745	0	0	0	0	0	0	0	0	0	0	0	0	2	0	0	0	0	0	2
d__Bacteria	k__norank_d__Bacteria	p__Bacteroidetes	c__Sphingobacteriia	o__Sphingobacteriales	f__Chitinophagaceae	g__unclassified_f__Chitinophagaceae	s__unclassified_f__Chitinophagaceae	OTU2463	4	0	1	5	0	0	2	1	5	0	2	0	0	1	0	1	2	2	26
d__Bacteria	k__norank_d__Bacteria	p__Firmicutes	c__Clostridia	o__Clostridiales	f__Clostridiaceae_1	g__Clostridium_sensu_stricto_1	s__unclassified_g__Clostridium_sensu_stricto_1	OTU3213	14	97	41	4	8	8	21	4	5	4	20	25	3	13	2	4	10	6	289
d__Bacteria	k__norank_d__Bacteria	p__Proteobacteria	c__Alphaproteobacteria	o__Rhodospirillales	f__Rhodospirillaceae	g__Azospirillum	s__unclassified_g__Azospirillum	OTU2045	1	3	0	0	0	0	1	0	0	0	0	0	0	0	0	0	0	0	5

d__Bacteria	k__norank_d__Bacteria	p__Firmicutes	c__Bacilli	o__Bacillales	f__Paenibacillaceae	g__Paenibacillus	s__unclassified_g__Paenibacillus	OTU402	0	7	0	0	0	0	0	0	0	0	0	0	0	0	0	0	1	0	8
d__Bacteria	k__norank_d__Bacteria	p__unclassified_k__norank_d__Bacteria	c__unclassified_k__norank_d__Bacteria	o__unclassified_k__norank_d__Bacteria	f__unclassified_k__norank_d__Bacteria	g__unclassified_k__norank_d__Bacteria	s__unclassified_k__norank_d__Bacteria	OTU4812	1	0	1	0	0	0	0	0	0	0	0	0	0	2	0	0	0	0	4
d__Bacteria	k__norank_d__Bacteria	p__Actinobacteria	c__Actinobacteria	o__Solirubrobacterales	f__Elev-16S-1332	g__norank_f__Elev-16S-1332	s__uncultured_bacterium_g__norank_f__Elev-16S-1332	OTU1386	0	1	2	2	3	0	0	1	0	0	1	0	0	0	0	0	0	0	10
d__Bacteria	k__norank_d__Bacteria	p__Actinobacteria	c__Actinobacteria	o__Gaiellales	f__norank_o__Gaiellales	g__norank_o__Gaiellales	s__unclassified_g__norank_o__Gaiellales	OTU1324	0	0	0	2	0	0	0	0	0	0	2	0	0	0	0	0	0	0	4
d__Bacteria	k__norank_d__Bacteria	p__Chloroflexi	c__Thermomicrobia	o__JG30-KF-CM45	f__norank_o__JG30-KF-CM45	g__norank_o__JG30-KF-CM45	s__uncultured_Chloroflexi_bacterium_g__norank_o__JG30-KF-CM45	OTU122	2	0	0	0	0	0	0	0	0	0	0	0	0	0	0	0	0	0	2
d__Bacteria	k__norank_d__Bacteria	p__Planctomycetes	c__Planctomycetacia	o__Planctomycetales	f__Planctomycetaceae	g__unclassified_f__Planctomycetaceae	s__unclassified_f__Planctomycetaceae	OTU5075	0	0	0	0	0	0	0	0	0	1	0	0	0	2	0	0	0	0	3
d__Bacteria	k__norank_d__Bacteria	p__Proteobacteria	c__Alphaproteobacteria	o__Rhodospirillales	f__Rhodospirillaceae	g__unclassified_f__Rhodospirillaceae	s__unclassified_f__Rhodospirillaceae	OTU3054	5	7	6	9	5	22	9	18	7	7	16	14	4	10	4	3	7	17	170
d__Bacteria	k__norank_d__Bacteria	p__Chlamydiae	c__Chlamydiae	o__Chlamydiales	f__cvE6	g__norank_f__cvE6	s__uncultured_bacterium_g__norank_f__cvE6	OTU4723	0	0	0	0	0	0	1	0	0	0	0	0	3	0	0	0	0	0	4
d__Bacteria	k__norank_d__Bacteria	p__Verrucomicrobia	c__S-BQ2-57_soil_group	o__norank_c__S-BQ2-57_soil_group	f__norank_c__S-BQ2-57_soil_group	g__norank_c__S-BQ2-57_soil_group	s__uncultured_Verrucomicrobia_bacterium_g__norank_c__S-BQ2-57_soil_group	OTU5274	3	2	1	9	4	3	0	1	0	4	0	0	4	2	1	0	0	0	34
d__Bacteria	k__norank_d__Bacteria	p__Proteobacteria	c__Deltaproteobacteria	o__Desulfovibrionales	f__Desulfovibrionaceae	g__Desulfovibrio	s__unclassified_g__Desulfovibrio	OTU2831	0	1	0	0	0	0	0	0	1	1	0	0	0	0	0	0	0	1	4
d__Bacteria	k__norank_d__Bacteria	p__unclassified_k__norank_d__Bacteria	c__unclassified_k__norank_d__Bacteria	o__unclassified_k__norank_d__Bacteria	f__unclassified_k__norank_d__Bacteria	g__unclassified_k__norank_d__Bacteria	s__unclassified_k__norank_d__Bacteria	OTU2305	0	0	0	0	0	0	0	0	2	0	0	0	0	0	0	0	0	0	2
d__Bacteria	k__norank_d__Bacteria	p__Proteobacteria	c__Gammaproteobacteria	o__Xanthomonadales	f__norank_o__Xanthomonadales	g__norank_o__Xanthomonadales	s__uncultured_gamma_proteobacterium_g__norank_o__Xanthomonadales	OTU1959	0	0	0	0	0	0	4	0	1	0	0	0	1	0	0	0	0	0	6
d__Bacteria	k__norank_d__Bacteria	p__Bacteroidetes	c__Cytophagia	o__Cytophagales	f__Cytophagaceae	g__norank_f__Cytophagaceae	s__unclassified_g__norank_f__Cytophagaceae	OTU3504	36	141	64	9	22	56	0	0	0	43	8	1	63	16	2	0	0	0	461
d__Bacteria	k__norank_d__Bacteria	p__Cyanobacteria	c__Cyanobacteria	o__norank_c__Cyanobacteria	f__norank_c__Cyanobacteria	g__norank_c__Cyanobacteria	s__unclassified_g__norank_c__Cyanobacteria	OTU5650	0	0	0	0	0	0	0	0	0	0	0	0	0	0	4	0	0	0	4
d__Bacteria	k__norank_d__Bacteria	p__Saccharibacteria	c__norank_p__Saccharibacteria	o__norank_p__Saccharibacteria	f__norank_p__Saccharibacteria	g__norank_p__Saccharibacteria	s__uncultured_bacterium_g__norank_p__Saccharibacteria	OTU1808	1	0	1	0	0	1	0	0	1	0	0	0	0	2	0	0	0	0	6
d__Bacteria	k__norank_d__Bacteria	p__Chloroflexi	c__Ktedonobacteria	o__Thermogemmatisporales	f__1921-2	g__norank_f__1921-2	s__uncultured_bacterium_g__norank_f__1921-2	OTU3665	14	0	0	1	0	0	3	0	0	0	2	0	0	0	0	0	0	1	21
d__Bacteria	k__norank_d__Bacteria	p__Actinobacteria	c__Actinobacteria	o__Micrococcales	f__Micrococcaceae	g__unclassified_f__Micrococcaceae	s__unclassified_f__Micrococcaceae	OTU5979	0	0	0	0	0	0	0	1	0	0	0	0	0	0	0	0	0	3	4
d__Bacteria	k__norank_d__Bacteria	p__Planctomycetes	c__BD7-11	o__norank_c__BD7-11	f__norank_c__BD7-11	g__norank_c__BD7-11	s__uncultured_bacterium_g__norank_c__BD7-11	OTU1712	0	0	0	0	0	1	0	0	0	0	0	0	0	0	0	0	0	0	1
d__Bacteria	k__norank_d__Bacteria	p__TM6__Dependentiae_	c__norank_p__TM6__Dependentiae_	o__norank_p__TM6__Dependentiae_	f__norank_p__TM6__Dependentiae_	g__norank_p__TM6__Dependentiae_	s__uncultured_bacterium_g__norank_p__TM6__Dependentiae_	OTU5683	0	0	0	0	0	0	0	0	0	1	0	0	0	0	1	0	0	0	2
d__Bacteria	k__norank_d__Bacteria	p__Proteobacteria	c__Gammaproteobacteria	o__Pseudomonadales	f__Pseudomonadaceae	g__Pseudomonas	s__unclassified_g__Pseudomonas	OTU1952	0	0	0	0	0	0	9	2	1	0	0	0	0	0	0	2	0	0	14
d__Bacteria	k__norank_d__Bacteria	p__unclassified_k__norank_d__Bacteria	c__unclassified_k__norank_d__Bacteria	o__unclassified_k__norank_d__Bacteria	f__unclassified_k__norank_d__Bacteria	g__unclassified_k__norank_d__Bacteria	s__unclassified_k__norank_d__Bacteria	OTU54	1	0	0	0	0	0	0	0	0	0	0	0	0	0	0	0	0	0	1
d__Bacteria	k__norank_d__Bacteria	p__Proteobacteria	c__Gammaproteobacteria	o__HTA4	f__norank_o__HTA4	g__norank_o__HTA4	s__uncultured_bacterium_g__norank_o__HTA4	OTU3243	1	0	1	0	0	0	0	0	2	0	3	2	1	0	3	0	0	3	16
d__Bacteria	k__norank_d__Bacteria	p__Chloroflexi	c__Thermomicrobia	o__JG30-KF-CM45	f__norank_o__JG30-KF-CM45	g__norank_o__JG30-KF-CM45	s__bacterium_Ellin6537	OTU964	2	5	0	1	2	1	1	0	0	0	3	2	2	0	0	0	4	1	24
d__Bacteria	k__norank_d__Bacteria	p__Proteobacteria	c__Alphaproteobacteria	o__Rhizobiales	f__Methylobacteriaceae	g__Methylobacterium	s__unclassified_g__Methylobacterium	OTU2094	0	0	0	0	0	0	0	2	0	0	1	0	0	0	0	0	0	0	3
d__Bacteria	k__norank_d__Bacteria	p__Firmicutes	c__Bacilli	o__Bacillales	f__Thermoactinomycetaceae	g__Thermoflavimicrobium	s__unclassified_g__Thermoflavimicrobium	OTU4285	0	0	0	0	0	0	0	0	0	0	2	0	0	0	0	0	0	0	2
d__Bacteria	k__norank_d__Bacteria	p__Acidobacteria	c__Acidobacteria	o__Acidobacteriales	f__Acidobacteriaceae__Subgroup_1_	g__norank_f__Acidobacteriaceae__Subgroup_1_	s__unclassified_g__norank_f__Acidobacteriaceae__Subgroup_1_	OTU1278	145	146	267	397	40	66	69	49	73	91	140	101	105	182	129	18	63	68	2149
d__Bacteria	k__norank_d__Bacteria	p__Proteobacteria	c__Alphaproteobacteria	o__Rhizobiales	f__Rhizobiales_Incertae_Sedis	g__Nordella	s__Nordella_oligomobilis	OTU2291	2	22	8	5	0	0	11	17	7	1	2	0	10	5	4	3	37	4	138
d__Bacteria	k__norank_d__Bacteria	p__Chloroflexi	c__SBR2076	o__norank_c__SBR2076	f__norank_c__SBR2076	g__norank_c__SBR2076	s__unclassified_g__norank_c__SBR2076	OTU3475	0	0	0	1	0	1	0	0	0	0	0	0	0	1	2	0	1	0	6
d__Bacteria	k__norank_d__Bacteria	p__Bacteroidetes	c__Sphingobacteriia	o__Sphingobacteriales	f__env.OPS_17	g__norank_f__env.OPS_17	s__uncultured_bacterium_g__norank_f__env.OPS_17	OTU1233	7	2	2	0	0	0	0	0	0	0	0	0	0	0	0	0	0	0	11
d__Bacteria	k__norank_d__Bacteria	p__Chloroflexi	c__TK10	o__norank_c__TK10	f__norank_c__TK10	g__norank_c__TK10	s__uncultured_Chloroflexus_sp._g__norank_c__TK10	OTU400	1	2	6	1	0	5	0	2	0	0	0	1	0	0	0	0	0	0	18
d__Bacteria	k__norank_d__Bacteria	p__Proteobacteria	c__Gammaproteobacteria	o__Legionellales	f__Coxiellaceae	g__Aquicella	s__unclassified_g__Aquicella	OTU5760	0	0	0	0	0	0	0	0	0	0	0	0	2	0	1	0	5	1	9
d__Bacteria	k__norank_d__Bacteria	p__Bacteroidetes	c__Bacteroidia	o__Bacteroidales	f__Porphyromonadaceae	g__Dysgonomonas	s__unclassified_g__Dysgonomonas	OTU111	3	0	0	0	0	0	0	0	0	0	0	0	0	0	0	0	0	0	3
d__Bacteria	k__norank_d__Bacteria	p__Bacteroidetes	c__Sphingobacteriia	o__Sphingobacteriales	f__Chitinophagaceae	g__Chitinophaga	s__Chitinophaga_arvensicola	OTU1975	6	0	0	0	0	0	3	0	0	0	0	0	0	0	0	0	0	0	9
d__Bacteria	k__norank_d__Bacteria	p__Acidobacteria	c__Acidobacteria	o__Blastocatellales	f__Blastocatellaceae__Subgroup_4_	g__11-24	s__unclassified_g__11-24	OTU4701	0	2	0	4	1	0	0	0	0	2	0	0	2	1	1	0	1	0	14
d__Bacteria	k__norank_d__Bacteria	p__Chloroflexi	c__Ktedonobacteria	o__C0119	f__norank_o__C0119	g__norank_o__C0119	s__uncultured_Chloroflexi_bacterium_g__norank_o__C0119	OTU2098	2	1	1	0	0	0	0	1	0	0	0	0	0	0	0	0	0	0	5
[illegible]																											

d__Bacteria	k__norank_d__Bacteria	p__Proteobacteria	c__Deltaproteobacteria	o__Myxococcales	f__Haliangiaceae	g__Haliangium	s__unclassified_g__Haliangium	OTU2945	6	5	5	7	2	0	0	0	0	2	0	2	3	3	2	0	0	4	41
d__Bacteria	k__norank_d__Bacteria	p__Verrucomicrobia	c__OPB35_soil_group	o__norank_c__OPB35_soil_group	f__norank_c__OPB35_soil_group	g__norank_c__OPB35_soil_group	s__unclassified_g__norank_c__OPB35_soil_group	OTU3328	51	31	41	54	68	46	13	10	16	79	100	64	58	43	50	3	16	17	760
d__Bacteria	k__norank_d__Bacteria	p__Acidobacteria	c__Acidobacteria	o__unclassified_c__Acidobacteria	f__unclassified_c__Acidobacteria	g__unclassified_c__Acidobacteria	s__unclassified_c__Acidobacteria	OTU255	0	2	0	0	0	0	0	0	0	0	0	0	0	0	0	0	0	0	2
d__Bacteria	k__norank_d__Bacteria	p__TM6__Dependentiae_	c__norank_p__TM6__Dependentiae_	o__norank_p__TM6__Dependentiae_	f__norank_p__TM6__Dependentiae_	g__norank_p__TM6__Dependentiae_	s__unclassified_g__norank_p__TM6__Dependentiae_	OTU66	2	0	0	0	0	0	0	0	0	0	0	0	0	0	0	0	0	0	2
d__Bacteria	k__norank_d__Bacteria	p__Proteobacteria	c__Deltaproteobacteria	o__Myxococcales	f__Phaselicystidaceae	g__Phaselicystis	s__uncultured_delta_proteobacterium_g__Phaselicystis	OTU922	1	5	0	2	4	1	0	0	0	1	0	0	2	0	0	0	0	0	16
d__Bacteria	k__norank_d__Bacteria	p__Proteobacteria	c__Deltaproteobacteria	o__Myxococcales	f__KD3-10	g__norank_f__KD3-10	s__uncultured_bacterium_g__norank_f__KD3-10	OTU5861	3	0	2	2	1	1	0	0	0	0	0	0	0	0	1	0	2	0	12
d__Bacteria	k__norank_d__Bacteria	p__Verrucomicrobia	c__OPB35_soil_group	o__norank_c__OPB35_soil_group	f__norank_c__OPB35_soil_group	g__norank_c__OPB35_soil_group	s__unclassified_g__norank_c__OPB35_soil_group	OTU3456	3	17	29	2	42	8	0	0	0	4	6	1	25	3	36	0	0	0	176
d__Bacteria	k__norank_d__Bacteria	p__Acidobacteria	c__Acidobacteria	o__norank_c__Acidobacteria	f__norank_c__Acidobacteria	g__norank_c__Acidobacteria	s__unclassified_g__norank_c__Acidobacteria	OTU2167	0	2	0	1	0	0	0	2	0	0	0	0	0	0	0	0	0	0	5
d__Bacteria	k__norank_d__Bacteria	p__Planctomycetes	c__Planctomycetacia	o__Planctomycetales	f__Planctomycetaceae	g__Pirellula	s__unclassified_g__Pirellula	OTU192	4	4	0	1	2	1	1	0	0	0	0	0	0	0	0	0	0	0	13
d__Bacteria	k__norank_d__Bacteria	p__Actinobacteria	c__Actinobacteria	o__Pseudonocardiales	f__Pseudonocardiaceae	g__unclassified_f__Pseudonocardiaceae	s__unclassified_f__Pseudonocardiaceae	OTU2987	10	5	9	26	18	15	6	0	1	24	18	56	5	24	11	0	6	1	235
d__Bacteria	k__norank_d__Bacteria	p__Proteobacteria	c__Gammaproteobacteria	o__Legionellales	f__Coxiellaceae	g__Aquicella	s__unclassified_g__Aquicella	OTU5011	0	0	0	0	0	0	0	0	0	0	0	0	0	2	0	0	0	0	2
d__Bacteria	k__norank_d__Bacteria	p__Parcubacteria	c__norank_p__Parcubacteria	o__norank_p__Parcubacteria	f__norank_p__Parcubacteria	g__norank_p__Parcubacteria	s__uncultured_bacterium_g__norank_p__Parcubacteria	OTU4836	1	1	0	0	2	1	0	0	0	1	0	0	0	1	0	0	0	0	7
d__Bacteria	k__norank_d__Bacteria	p__Armatimonadetes	c__Armatimonadia	o__Armatimonadales	f__norank_o__Armatimonadales	g__norank_o__Armatimonadales	s__unclassified_g__norank_o__Armatimonadales	OTU5539	2	0	6	2	7	5	3	0	1	6	10	4	3	2	2	0	0	0	53
d__Bacteria	k__norank_d__Bacteria	p__Parcubacteria	c__norank_p__Parcubacteria	o__norank_p__Parcubacteria	f__norank_p__Parcubacteria	g__norank_p__Parcubacteria	s__unclassified_g__norank_p__Parcubacteria	OTU5585	2	0	2	3	5	2	0	0	0	12	4	7	5	0	9	0	0	0	51
d__Bacteria	k__norank_d__Bacteria	p__Chlamydiae	c__Chlamydiae	o__Chlamydiales	f__cvE6	g__norank_f__cvE6	s__uncultured_bacterium_g__norank_f__cvE6	OTU3815	0	0	0	0	0	0	0	0	0	1	1	0	0	0	0	0	0	0	2
d__Bacteria	k__norank_d__Bacteria	p__Chloroflexi	c__Ktedonobacteria	o__Ktedonobacterales	f__Thermosporotrichaceae	g__Thermosporothrix	s__uncultured_bacterium_g__Thermosporothrix	OTU668	2	1	9	6	1	5	2	1	0	1	3	4	3	2	1	0	1	0	42
d__Bacteria	k__norank_d__Bacteria	p__Proteobacteria	c__Deltaproteobacteria	o__Oligoflexales	f__Oligoflexaceae	g__norank_f__Oligoflexaceae	s__uncultured_soil_bacterium_g__norank_f__Oligoflexaceae	OTU3865	0	0	0	0	3	2	0	0	0	0	7	3	0	0	0	0	0	0	15
d__Bacteria	k__norank_d__Bacteria	p__Proteobacteria	c__Alphaproteobacteria	o__Rhodospirillales	f__DA111	g__norank_f__DA111	s__uncultured_bacterium_g__norank_f__DA111	OTU937	0	2	0	2	0	0	0	0	0	2	1	0	1	0	0	0	0	0	8
d__Bacteria	k__norank_d__Bacteria	p__Proteobacteria	c__Deltaproteobacteria	o__Bdellovibrionales	f__Bdellovibrionaceae	g__Bdellovibrio	s__unclassified_g__Bdellovibrio	OTU2588	0	0	0	0	0	0	0	0	4	0	0	0	0	0	0	0	0	0	4
d__Bacteria	k__norank_d__Bacteria	p__Proteobacteria	c__Gammaproteobacteria	o__Xanthomonadales	f__Xanthomonadales_Incertae_Sedis	g__Steroidobacter	s__unclassified_g__Steroidobacter	OTU5377	1	1	3	2	1	0	0	0	0	0	0	0	1	0	3	0	0	0	12
d__Bacteria	k__norank_d__Bacteria	p__Planctomycetes	c__Planctomycetacia	o__Planctomycetales	f__Planctomycetaceae	g__unclassified_f__Planctomycetaceae	s__unclassified_f__Planctomycetaceae	OTU2886	0	1	0	1	1	0	0	0	0	4	0	2	1	0	0	0	0	0	10
d__Bacteria	k__norank_d__Bacteria	p__Chloroflexi	c__JG37-AG-4	o__norank_c__JG37-AG-4	f__norank_c__JG37-AG-4	g__norank_c__JG37-AG-4	s__uncultured_bacterium_g__norank_c__JG37-AG-4	OTU1798	15	4	12	16	26	20	1	4	6	32	40	76	6	14	10	2	8	8	300
d__Bacteria	k__norank_d__Bacteria	p__Acidobacteria	c__Acidobacteria	o__Solibacterales	f__Solibacteraceae__Subgroup_3_	g__Bryobacter	s__uncultured_Acidobacteria_bacterium_g__Bryobacter	OTU778	1	0	3	0	0	2	2	0	1	1	3	1	0	1	2	0	0	0	17
d__Bacteria	k__norank_d__Bacteria	p__Gemmatimonadetes	c__Gemmatimonadetes	o__Gemmatimonadales	f__Gemmatimonadaceae	g__norank_f__Gemmatimonadaceae	s__unclassified_g__norank_f__Gemmatimonadaceae	OTU1714	0	1	3	0	2	7	2	0	0	4	7	4	9	1	2	0	0	0	42
d__Bacteria	k__norank_d__Bacteria	p__Actinobacteria	c__Actinobacteria	o__Frankiales	f__Acidothermaceae	g__Acidothermus	s__uncultured_actinobacterium_g__Acidothermus	OTU1442	0	0	1	0	4	0	0	0	0	0	3	1	0	0	0	0	1	0	10
d__Bacteria	k__norank_d__Bacteria	p__Cyanobacteria	c__Cyanobacteria	o__norank_c__Cyanobacteria	f__norank_c__Cyanobacteria	g__norank_c__Cyanobacteria	s__unclassified_g__norank_c__Cyanobacteria	OTU130	4	0	0	0	0	0	0	0	0	0	0	0	0	0	0	0	0	0	4
d__Bacteria	k__norank_d__Bacteria	p__unclassified_k__norank_d__Bacteria	c__unclassified_k__norank_d__Bacteria	o__unclassified_k__norank_d__Bacteria	f__unclassified_k__norank_d__Bacteria	g__unclassified_k__norank_d__Bacteria	s__unclassified_k__norank_d__Bacteria	OTU1759	0	1	0	0	0	1	0	0	0	0	0	0	0	0	0	0	0	0	2
d__Bacteria	k__norank_d__Bacteria	p__unclassified_k__norank_d__Bacteria	c__unclassified_k__norank_d__Bacteria	o__unclassified_k__norank_d__Bacteria	f__unclassified_k__norank_d__Bacteria	g__unclassified_k__norank_d__Bacteria	s__unclassified_k__norank_d__Bacteria	OTU1751	0	0	0	0	0	2	0	0	0	0	4	1	0	0	0	0	0	0	7
d__Bacteria	k__norank_d__Bacteria	p__Acidobacteria	c__Acidobacteria	o__Solibacterales	f__Solibacteraceae__Subgroup_3_	g__Candidatus_Solibacter	s__unclassified_g__Candidatus_Solibacter	OTU3917	214	121	186	350	376	370	43	12	24	383	498	560	166	238	358	7	36	31	3973
d__Bacteria	k__norank_d__Bacteria	p__Verrucomicrobia	c__Verrucomicrobia_Incertae_Sedis	o__Unknown_Order_c__Verrucomicrobia_Incertae_Sedis	f__Unknown_Family_o__Unknown_Order_c__Verrucomicrobia_Incertae_Sedis	g__Candidatus_Methylacidiphilum	s__uncultured_bacterium_g__Candidatus_Methylacidiphilum	OTU60	2	0	0	0	0	0	0	0	0	0	0	0	0	0	0	0	0	0	2
d__Bacteria	k__norank_d__Bacteria	p__Verrucomicrobia	c__Spartobacteria	o__Chthoniobacterales	f__unclassified_o__Chthoniobacterales	g__unclassified_o__Chthoniobacterales	s__unclassified_o__Chthoniobacterales	OTU4540	3	1	0	0	0	1	0	4	3	2	0	1	0	1	1	0	0	2	19
d__Bacteria	k__norank_d__Bacteria	p__Firmicutes	c__Bacilli	o__Bacillales	f__Paenibacillaceae	g__Paenibacillus	s__unclassified_g__Paenibacillus	OTU2232	0	0	0	0	0	0	1	2	12	0	3	0	0	0	0	4	5	12	39
d__Bacteria	k__norank_d__Bacteria	p__unclassified_k__norank_d__Bacteria	c__unclassified_k__norank_d__Bacteria	o__unclassified_k__norank_d__Bacteria	f__unclassified_k__norank_d__Bacteria	g__unclassified_k__norank_d__Bacteria	s__unclassified_k__norank_d__Bacteria	OTU5260	0	0	5	2	0	3	0	0	0	0	0	2	2	0	1	0	0	2	17
d__Bacteria	k__norank_d__Bacteria	p__Firmicutes	c__Bacilli	o__Bacillales	f__Paenibacillaceae	g__Paenibacillus	s__unclassified_g__Paenibacillus	OTU2234	0	0	0	0	0	0	0	3	24	0	0	0	0	0	0	0	2	1	30
d__Bacteria	k__norank_d__Bacteria	p__Chloroflexi	c__Ktedonobacteria	o__JG30-KF-AS9	f__norank_o__JG30-KF-AS9	g__norank_o__JG30-KF-AS9	s__uncultured_bacterium_g__norank_o__JG30-KF-AS9	OTU3675	3	3	6	9	6	10	4	1	1	6	16	12	2	5	3	4	3	8	102
[illegible]																											

d__Bacteria	k__norank_d__Bacteria	p__Chlamydiae	c__Chlamydiae	o__Chlamydiales	f__Simkaniaceae	g__unclassified_f__Simkaniaceae	s__unclassified_f__Simkaniaceae	OTU1844	0	0	1	1	0	2	0	0	0	0	0	0	0	0	1	0	0	0	5
d__Bacteria	k__norank_d__Bacteria	p__Chloroflexi	c__unclassified_p__Chloroflexi	o__unclassified_p__Chloroflexi	f__unclassified_p__Chloroflexi	g__unclassified_p__Chloroflexi	s__unclassified_p__Chloroflexi	OTU5018	0	0	0	0	0	0	0	0	0	0	0	0	0	3	0	0	0	0	3
d__Bacteria	k__norank_d__Bacteria	p__Proteobacteria	c__Alphaproteobacteria	o__Sphingomonadales	f__Sphingomonadaceae	g__Novosphingobium	s__Novosphingobium_rosa	OTU495	1	7	0	0	0	0	0	0	0	1	0	0	2	0	0	0	0	0	11
d__Bacteria	k__norank_d__Bacteria	p__Acidobacteria	c__Acidobacteria	o__norank_c__Acidobacteria	f__norank_c__Acidobacteria	g__norank_c__Acidobacteria	s__unclassified_g__norank_c__Acidobacteria	OTU4195	0	0	0	0	0	0	0	0	0	0	3	0	0	0	0	0	0	0	3
d__Bacteria	k__norank_d__Bacteria	p__unclassified_k__norank_d__Bacteria	c__unclassified_k__norank_d__Bacteria	o__unclassified_k__norank_d__Bacteria	f__unclassified_k__norank_d__Bacteria	g__unclassified_k__norank_d__Bacteria	s__unclassified_k__norank_d__Bacteria	OTU4893	0	1	2	0	0	1	0	0	0	1	0	0	0	1	0	0	0	0	6
d__Bacteria	k__norank_d__Bacteria	p__Microgenomates	c__norank_p__Microgenomates	o__norank_p__Microgenomates	f__norank_p__Microgenomates	g__norank_p__Microgenomates	s__uncultured_candidate_division_WS6_bacterium_g__norank	OTU1730	0	0	0	1	0	2	0	0	0	0	0	0	0	0	0	0	0	0	3
d__Bacteria	k__norank_d__Bacteria	p__Proteobacteria	c__Alphaproteobacteria	o__Rhodospirillales	f__Rhodospirillaceae	g__Inquilinus	s__uncultured_alpha_proteobacterium_g__Inquilinus	OTU4008	16	26	11	10	7	6	7	8	12	9	12	13	10	14	22	5	6	4	198
d__Bacteria	k__norank_d__Bacteria	p__Proteobacteria	c__Deltaproteobacteria	o__Oligoflexales	f__Oligoflexaceae	g__norank_f__Oligoflexaceae	s__unclassified_g__norank_f__Oligoflexaceae	OTU1200	1	2	0	2	1	0	0	0	0	0	0	1	0	0	0	0	0	0	7
d__Bacteria	k__norank_d__Bacteria	p__Proteobacteria	c__Gammaproteobacteria	o__HTA4	f__norank_o__HTA4	g__norank_o__HTA4	s__unclassified_g__norank_o__HTA4	OTU1642	0	0	0	0	1	0	1	0	2	5	1	0	1	0	0	0	0	4	15
d__Bacteria	k__norank_d__Bacteria	p__Proteobacteria	c__Gammaproteobacteria	o__unclassified_c__Gammaproteobacteria	f__unclassified_c__Gammaproteobacteria	g__unclassified_c__Gammaproteobacteria	s__unclassified_c__Gammaproteobacteria	OTU2226	1	0	2	1	0	1	0	0	1	0	0	0	0	0	0	0	0	0	6
d__Bacteria	k__norank_d__Bacteria	p__Chlamydiae	c__Chlamydiae	o__Chlamydiales	f__unclassified_o__Chlamydiales	g__unclassified_o__Chlamydiales	s__unclassified_o__Chlamydiales	OTU1562	0	2	0	0	4	2	0	0	0	0	0	0	0	0	0	0	0	0	8
d__Bacteria	k__norank_d__Bacteria	p__Armatimonadetes	c__Fimbriimonadia	o__Fimbriimonadales	f__Fimbriimonadaceae	g__norank_f__Fimbriimonadaceae	s__uncultured_bacterium_g__norank_f__Fimbriimonadaceae	OTU908	2	1	0	5	1	1	0	0	0	3	3	0	0	0	0	0	2	1	19
d__Bacteria	k__norank_d__Bacteria	p__TM6__Dependentiae_	c__norank_p__TM6__Dependentiae_	o__norank_p__TM6__Dependentiae_	f__norank_p__TM6__Dependentiae_	g__norank_p__TM6__Dependentiae_	s__uncultured_bacterium_g__norank_p__TM6__Dependentiae_	OTU4007	0	0	0	0	0	0	0	0	0	0	2	0	0	0	0	0	0	0	2
d__Bacteria	k__norank_d__Bacteria	p__Parcubacteria	c__unclassified_p__Parcubacteria	o__unclassified_p__Parcubacteria	f__unclassified_p__Parcubacteria	g__unclassified_p__Parcubacteria	s__unclassified_p__Parcubacteria	OTU751	0	0	1	0	0	0	0	0	0	0	0	0	0	0	0	0	0	0	1
d__Bacteria	k__norank_d__Bacteria	p__Firmicutes	c__Bacilli	o__Bacillales	f__Paenibacillaceae	g__Paenibacillus	s__unclassified_g__Paenibacillus	OTU488	0	3	0	0	0	0	0	0	0	0	0	0	0	0	0	0	0	0	3
d__Bacteria	k__norank_d__Bacteria	p__Acidobacteria	c__Acidobacteria	o__Solibacterales	f__Solibacteraceae__Subgroup_3_	g__Candidatus_Solibacter	s__unclassified_g__Candidatus_Solibacter	OTU4449	4	5	2	6	11	3	3	4	0	5	5	15	7	5	8	0	0	0	83
d__Bacteria	k__norank_d__Bacteria	p__Firmicutes	c__Bacilli	o__Bacillales	f__Paenibacillaceae	g__Paenibacillus	s__unclassified_g__Paenibacillus	OTU5860	3	1	1	0	4	0	27	54	43	0	0	1	1	0	0	1	84	33	253
d__Bacteria	k__norank_d__Bacteria	p__unclassified_k__norank_d__Bacteria	c__unclassified_k__norank_d__Bacteria	o__unclassified_k__norank_d__Bacteria	f__unclassified_k__norank_d__Bacteria	g__unclassified_k__norank_d__Bacteria	s__unclassified_k__norank_d__Bacteria	OTU3387	3	5	5	4	5	7	1	2	0	15	15	7	2	9	4	0	2	1	87
d__Bacteria	k__norank_d__Bacteria	p__Proteobacteria	c__Gammaproteobacteria	o__Legionellales	f__Legionellaceae	g__Legionella	s__unclassified_g__Legionella	OTU4901	0	0	0	0	0	0	0	0	0	0	0	0	0	2	0	0	0	0	2
d__Bacteria	k__norank_d__Bacteria	p__unclassified_k__norank_d__Bacteria	c__unclassified_k__norank_d__Bacteria	o__unclassified_k__norank_d__Bacteria	f__unclassified_k__norank_d__Bacteria	g__unclassified_k__norank_d__Bacteria	s__unclassified_k__norank_d__Bacteria	OTU3656	5	1	0	1	3	4	1	3	0	3	8	0	0	3	0	0	4	2	38
d__Bacteria	k__norank_d__Bacteria	p__Chloroflexi	c__Ktedonobacteria	o__Ktedonobacterales	f__JG30a-KF-32	g__norank_f__JG30a-KF-32	s__uncultured_bacterium_g__norank_f__JG30a-KF-32	OTU71	3	0	0	0	0	0	0	0	0	0	0	0	0	0	2	0	0	0	5
d__Bacteria	k__norank_d__Bacteria	p__Actinobacteria	c__Actinobacteria	o__Acidimicrobiales	f__norank_o__Acidimicrobiales	g__norank_o__Acidimicrobiales	s__bacterium_enrichment_culture_clone_auto73_4W	OTU5147	3	3	3	1	0	1	3	1	1	1	4	0	3	2	6	0	6	0	38
d__Bacteria	k__norank_d__Bacteria	p__Acidobacteria	c__Acidobacteria	o__norank_c__Acidobacteria	f__norank_c__Acidobacteria	g__norank_c__Acidobacteria	s__uncultured_forest_soil_bacterium_g__norank_c__Acidobacteria	OTU2287	5	11	5	12	5	3	3	4	2	5	6	9	6	4	6	2	2	3	93
d__Bacteria	k__norank_d__Bacteria	p__Actinobacteria	c__Actinobacteria	o__Acidimicrobiales	f__unclassified_o__Acidimicrobiales	g__unclassified_o__Acidimicrobiales	s__unclassified_o__Acidimicrobiales	OTU1850	0	0	1	0	0	3	0	0	0	0	0	0	0	2	0	0	1	0	7
d__Bacteria	k__norank_d__Bacteria	p__unclassified_k__norank_d__Bacteria	c__unclassified_k__norank_d__Bacteria	o__unclassified_k__norank_d__Bacteria	f__unclassified_k__norank_d__Bacteria	g__unclassified_k__norank_d__Bacteria	s__unclassified_k__norank_d__Bacteria	OTU2384	1	0	0	1	2	0	0	0	2	9	0	0	0	0	0	0	0	0	15
d__Bacteria	k__norank_d__Bacteria	p__Actinobacteria	c__Actinobacteria	o__Solirubrobacterales	f__YNPFFP1	g__norank_f__YNPFFP1	s__uncultured_bacterium_g__norank_f__YNPFFP1	OTU143	9	5	6	9	7	13	2	1	0	18	18	12	3	5	12	2	6	5	133
d__Bacteria	k__norank_d__Bacteria	p__Proteobacteria	c__Alphaproteobacteria	o__Rhizobiales	f__Xanthobacteraceae	g__norank_f__Xanthobacteraceae	s__unclassified_g__norank_f__Xanthobacteraceae	OTU195	0	2	0	0	0	0	0	0	0	0	0	0	0	0	0	0	0	0	2
d__Bacteria	k__norank_d__Bacteria	p__Proteobacteria	c__Alphaproteobacteria	o__Rhizobiales	f__alphaI_cluster	g__norank_f__alphaI_cluster	s__uncultured_bacterium_g__norank_f__alphaI_cluster	OTU2601	5	4	6	1	5	0	4	5	8	3	3	1	4	2	4	1	5	7	68
d__Bacteria	k__norank_d__Bacteria	p__Bacteroidetes	c__Sphingobacteriia	o__Sphingobacteriales	f__unclassified_o__Sphingobacteriales	g__unclassified_o__Sphingobacteriales	s__unclassified_o__Sphingobacteriales	OTU726	0	0	3	0	0	0	0	0	0	0	0	0	1	0	0	0	0	0	4
d__Bacteria	k__norank_d__Bacteria	p__Bacteroidetes	c__Sphingobacteriia	o__Sphingobacteriales	f__Chitinophagaceae	g__norank_f__Chitinophagaceae	s__unclassified_g__norank_f__Chitinophagaceae	OTU2575	8	11	13	28	39	32	3	1	2	28	8	50	6	29	14	1	3	1	277
d__Bacteria	k__norank_d__Bacteria	p__unclassified_k__norank_d__Bacteria	c__unclassified_k__norank_d__Bacteria	o__unclassified_k__norank_d__Bacteria	f__unclassified_k__norank_d__Bacteria	g__unclassified_k__norank_d__Bacteria	s__unclassified_k__norank_d__Bacteria	OTU3017	1	2	0	0	0	31	0	0	0	1	3	0	6	1	0	0	0	0	45
d__Bacteria	k__norank_d__Bacteria	p__unclassified_k__norank_d__Bacteria	c__unclassified_k__norank_d__Bacteria	o__unclassified_k__norank_d__Bacteria	f__unclassified_k__norank_d__Bacteria	g__unclassified_k__norank_d__Bacteria	s__unclassified_k__norank_d__Bacteria	OTU3032	0	0	0	0	0	0	0	0	0	0	2	0	0	0	0	0	0	0	2
d__Bacteria	k__norank_d__Bacteria	p__unclassified_k__norank_d__Bacteria	c__unclassified_k__norank_d__Bacteria	o__unclassified_k__norank_d__Bacteria	f__unclassified_k__norank_d__Bacteria	g__unclassified_k__norank_d__Bacteria	s__unclassified_k__norank_d__Bacteria	OTU3035	0	1	1	0	2	0	0	0	0	0	1	0	0	0	0	0	0	0	5
d__Bacteria	k__norank_d__Bacteria	p__Bacteroidetes	c__Cytophagia	o__Cytophagales	f__Cytophagaceae	g__norank_f__Cytophagaceae	s__unclassified_g__norank_f__Cytophagaceae	OTU221	0	10	0	0	0	0	0	0	0	0	0	0	4	0	2	0	0	0	16
d__Bacteria	k__norank_d__Bacteria	p__Proteobacteria	c__Deltaproteobacteria	o__Oligoflexales	f__Oligoflexaceae	g__norank_f__Oligoflexaceae	s__unclassified_g__norank_f__Oligoflexaceae	OTU2736	0	0	0	0	0	0	0	0	0	1	0	0	0	0	0	0	0	0	1
d__Bacteria	k__norank_d__Bacteria	p__Proteobacteria	c__Betaproteobacteria	o__Burkholderiales	f__Alcaligenaceae	g__norank_f__Alcaligenaceae	s__uncultured_Bordetella_sp._g__norank	OTU2928	591	117	1306	57	13	84	55	6	29	145	36	115	1887	3050	949	70	1342	461	10313
d__Bacteria	k__norank_d__Bacteria	p__Proteobacteria	c__Gammaproteobacteria	o__Legionellales	f__Coxiellaceae	g__Aquicella	s__unclassified_g__Aquicella	OTU5054	0	0	2	0	0	0	0	0	0	0	0	0	1	4	0	0	0	0	7
d__Bacteria	k__norank_d__Bacteria	p__Bacteroidetes	c__Sphingobacteriia	o__Sphingobacteriales	f__unclassified_o__Sphingobacteriales	g__unclassified_o__Sphingobacteriales	s__unclassified_o__Sphingobacteriales	OTU146	4	0	0	0	0	0	0	0	0	0	0	0	0	0	0	0	0	0	4
d__Bacteria	k__norank_d__Bacteria	p__Actinobacteria	c__Actinobacteria	o__Catenulisporales	f__Actinospicaceae	g__Actinospica	s__Actinospica_acidiphila	OTU3531	2	0	4	2	3	0	3	0	0	1	2	1	1	8	3	0	9	1	40
d__Bacteria	k__norank_d__Bacteria	p__Verrucomicrobia	c__OPB35_soil_group	o__norank_c__OPB35_soil_group	f__norank_c__OPB35_soil_group	g__norank_c__OPB35_soil_group	s__unclassified_g__norank_c__OPB35_soil_group	OTU3361	8	3	10	9	7	6	2	0	1	6	11	8	14	4	9	0	2	4	104
d__Bacteria	k__norank_d__Bacteria	p__Planctomycetes	c__Planctomycetacia	o__Planctomycetales	f__Planctomycetaceae	g__Pirellula	s__uncultured_soil_bacterium_g__Pirellula	OTU4311	1	4	3	5	0	0	1	2	0	0	4	0	0	6	3	0	3	0	32
d__Bacteria	k__norank_d__Bacteria	p__Acidobacteria	c__Acidobacteria	o__Acidobacteriales	f__Acidobacteriaceae__Subgroup_1_	g__Candidatus_Koribacter	s__uncultured_bacterium_g__Candidatus_Koribacter	OTU259	14	25	10	15	3	3	1	2	6	2	7	10	1	6	4	3	0	3	115
d__Bacteria	k__norank_d__Bacteria	p__Actinobacteria	c__Actinobacteria	o__Acidimicrobiales	f__norank_o__Acidimicrobiales	g__norank_o__Acidimicrobiales	s__uncultured_bacterium_g__norank_o__Acidimicrobiales	OTU4614	0	1	0	0	0	0	0	0	0	0	0	1	1	0	0	0	0	0	3
[illegible]																											

d__Bacteria	k__norank_d__Bacteria	p__Bacteroidetes	c__Cytophagia	o__Cytophagales	f__Cytophagaceae	g__Cytophaga	s__Hypsibius_dujardini_g__Cytophaga	OTU1240	8	3	1	5	2	1	0	2	0	5	0	0	1	0	0	0	0	0	28
d__Bacteria	k__norank_d__Bacteria	p__Proteobacteria	c__Gammaproteobacteria	o__Legionellales	f__Coxiellaceae	g__Aquicella	s__unclassified_g__Aquicella	OTU5092	0	4	0	1	0	0	0	2	0	0	0	0	2	0	1	0	5	6	21
d__Bacteria	k__norank_d__Bacteria	p__Acidobacteria	c__Acidobacteria	o__Acidobacteriales	f__Acidobacteriaceae__Subgroup_1_	g__Candidatus_Koribacter	s__unclassified_g__Candidatus_Koribacter	OTU3305	70	69	49	126	64	93	36	37	28	56	37	54	65	53	93	8	20	33	991
d__Bacteria	k__norank_d__Bacteria	p__Actinobacteria	c__Actinobacteria	o__Micromonosporales	f__Micromonosporaceae	g__Luedemannella	s__uncultured_bacterium_g__Luedemannella	OTU304	10	14	12	14	12	6	9	9	5	3	9	4	6	8	5	0	4	2	132
d__Bacteria	k__norank_d__Bacteria	p__Proteobacteria	c__Gammaproteobacteria	o__Legionellales	f__Coxiellaceae	g__Aquicella	s__uncultured_bacterium_g__Aquicella	OTU2489	0	0	0	1	2	0	0	0	3	3	2	0	0	2	0	0	0	0	13
d__Bacteria	k__norank_d__Bacteria	p__Verrucomicrobia	c__Spartobacteria	o__Chthoniobacterales	f__Chthoniobacteraceae	g__Chthoniobacter	s__bacterium_Ellin506	OTU6009	0	0	0	0	0	0	0	0	1	0	0	0	0	0	0	0	0	2	3
d__Bacteria	k__norank_d__Bacteria	p__Actinobacteria	c__Actinobacteria	o__Gaiellales	f__Gaiellaceae	g__Gaiella	s__unclassified_g__Gaiella	OTU4159	1	0	0	0	0	1	0	0	0	0	2	0	0	0	0	1	0	0	5
d__Bacteria	k__norank_d__Bacteria	p__Proteobacteria	c__Betaproteobacteria	o__Neisseriales	f__Neisseriaceae	g__Chitinibacter	s__unclassified_g__Chitinibacter	OTU118	2	0	0	0	0	0	0	0	0	0	0	0	0	0	0	0	0	0	2
d__Bacteria	k__norank_d__Bacteria	p__Proteobacteria	c__Alphaproteobacteria	o__unclassified_c__Alphaproteobacteria	f__unclassified_c__Alphaproteobacteria	g__unclassified_c__Alphaproteobacteria	s__unclassified_c__Alphaproteobacteria	OTU1441	0	0	0	0	3	0	0	0	0	0	0	1	0	0	0	0	0	0	4
d__Bacteria	k__norank_d__Bacteria	p__Planctomycetes	c__Planctomycetacia	o__Planctomycetales	f__Planctomycetaceae	g__Isosphaera	s__unclassified_g__Isosphaera	OTU2236	0	1	1	0	0	0	0	1	1	0	0	0	0	2	0	0	0	1	7
d__Bacteria	k__norank_d__Bacteria	p__Planctomycetes	c__Planctomycetacia	o__Planctomycetales	f__Planctomycetaceae	g__Gemmata	s__unclassified_g__Gemmata	OTU1219	0	0	0	1	1	2	0	0	0	0	0	1	0	0	0	0	0	0	5
d__Bacteria	k__norank_d__Bacteria	p__Proteobacteria	c__Gammaproteobacteria	o__unclassified_c__Gammaproteobacteria	f__unclassified_c__Gammaproteobacteria	g__unclassified_c__Gammaproteobacteria	s__unclassified_c__Gammaproteobacteria	OTU1420	0	1	0	0	1	0	0	0	0	0	0	0	0	0	0	0	0	0	2
d__Bacteria	k__norank_d__Bacteria	p__Firmicutes	c__Bacilli	o__Bacillales	f__Alicyclobacillaceae	g__Tumebacillus	s__unclassified_g__Tumebacillus	OTU4145	0	0	0	0	1	0	0	0	0	0	2	3	1	2	0	0	0	0	9
d__Bacteria	k__norank_d__Bacteria	p__Acidobacteria	c__Acidobacteria	o__norank_c__Acidobacteria	f__norank_c__Acidobacteria	g__norank_c__Acidobacteria	s__uncultured_Truepera_sp._g__norank	OTU856	0	0	2	0	0	0	0	0	0	0	0	0	0	0	0	0	0	0	2
d__Bacteria	k__norank_d__Bacteria	p__Acidobacteria	c__Acidobacteria	o__Acidobacteriales	f__Acidobacteriaceae__Subgroup_1_	g__Granulicella	s__unclassified_g__Granulicella	OTU3404	28	8	61	56	19	9	5	3	11	12	29	19	55	90	61	2	25	30	523
d__Bacteria	k__norank_d__Bacteria	p__Firmicutes	c__Clostridia	o__Clostridiales	f__Ruminococcaceae	g__unclassified_f__Ruminococcaceae	s__unclassified_f__Ruminococcaceae	OTU2023	2	1	2	0	0	1	4	0	0	0	0	0	0	1	0	0	2	0	13
d__Bacteria	k__norank_d__Bacteria	p__unclassified_k__norank_d__Bacteria	c__unclassified_k__norank_d__Bacteria	o__unclassified_k__norank_d__Bacteria	f__unclassified_k__norank_d__Bacteria	g__unclassified_k__norank_d__Bacteria	s__unclassified_k__norank_d__Bacteria	OTU1313	0	0	0	2	1	0	1	1	1	2	1	4	0	0	3	0	0	0	16
d__Bacteria	k__norank_d__Bacteria	p__Chloroflexi	c__Anaerolineae	o__Anaerolineales	f__Anaerolineaceae	g__norank_f__Anaerolineaceae	s__unclassified_g__norank_f__Anaerolineaceae	OTU3273	3	3	4	9	1	3	3	1	1	10	15	7	4	5	3	1	3	1	77
d__Bacteria	k__norank_d__Bacteria	p__Proteobacteria	c__Alphaproteobacteria	o__Rhodospirillales	f__Acetobacteraceae	g__norank_f__Acetobacteraceae	s__unclassified_g__norank_f__Acetobacteraceae	OTU832	0	0	1	0	0	0	0	0	0	0	0	0	0	0	0	0	0	0	1
d__Bacteria	k__norank_d__Bacteria	p__Chloroflexi	c__Ktedonobacteria	o__JG30-KF-AS9	f__norank_o__JG30-KF-AS9	g__norank_o__JG30-KF-AS9	s__uncultured_bacterium_g__norank_o__JG30-KF-AS9	OTU3777	0	1	3	0	0	0	0	0	1	0	3	1	1	3	4	0	0	0	17
d__Bacteria	k__norank_d__Bacteria	p__Bacteroidetes	c__Sphingobacteriia	o__Sphingobacteriales	f__norank_o__Sphingobacteriales	g__norank_o__Sphingobacteriales	s__unclassified_g__norank_o__Sphingobacteriales	OTU854	0	0	2	0	0	0	0	0	0	0	0	0	0	0	0	0	0	0	2
d__Bacteria	k__norank_d__Bacteria	p__Proteobacteria	c__Gammaproteobacteria	o__Legionellales	f__Coxiellaceae	g__Aquicella	s__unclassified_g__Aquicella	OTU5238	2	2	0	0	0	7	0	1	1	0	0	0	5	0	4	0	0	0	22
d__Bacteria	k__norank_d__Bacteria	p__Actinobacteria	c__Actinobacteria	o__Solirubrobacterales	f__Solirubrobacteraceae	g__Solirubrobacter	s__uncultured_bacterium_g__Solirubrobacter	OTU5056	3	1	2	2	0	0	0	2	0	0	0	2	0	1	0	2	0	0	15
d__Bacteria	k__norank_d__Bacteria	p__Proteobacteria	c__Gammaproteobacteria	o__Xanthomonadales	f__Xanthomonadaceae	g__Rhodanobacter	s__unclassified_g__Rhodanobacter	OTU5526	0	0	0	0	0	0	0	0	0	0	0	0	0	0	2	0	0	0	2
d__Bacteria	k__norank_d__Bacteria	p__Actinobacteria	c__Actinobacteria	o__Propionibacteriales	f__Nocardioidaceae	g__Nocardioides	s__unclassified_g__Nocardioides	OTU4987	0	0	1	0	1	1	1	0	1	1	2	0	1	2	0	0	0	0	11
d__Bacteria	k__norank_d__Bacteria	p__Planctomycetes	c__Planctomycetacia	o__Planctomycetales	f__Planctomycetaceae	g__norank_f__Planctomycetaceae	s__unclassified_g__norank_f__Planctomycetaceae	OTU1881	0	0	0	0	0	1	0	0	0	0	0	0	0	0	0	0	0	0	1
d__Bacteria	k__norank_d__Bacteria	p__Bacteroidetes	c__Sphingobacteriia	o__Sphingobacteriales	f__Chitinophagaceae	g__unclassified_f__Chitinophagaceae	s__unclassified_f__Chitinophagaceae	OTU1251	0	0	0	2	0	0	0	0	0	0	0	0	0	0	0	0	0	0	2
d__Bacteria	k__norank_d__Bacteria	p__Firmicutes	c__Erysipelotrichia	o__Erysipelotrichales	f__Erysipelotrichaceae	g__norank_f__Erysipelotrichaceae	s__uncultured_bacterium_g__norank_f__Erysipelotrichaceae	OTU5561	0	0	4	0	0	0	0	0	0	0	0	0	0	0	1	0	0	0	5
d__Bacteria	k__norank_d__Bacteria	p__Bacteroidetes	c__Sphingobacteriia	o__Sphingobacteriales	f__Chitinophagaceae	g__norank_f__Chitinophagaceae	s__Flavihumibacter_sp._MC113	OTU2445	28	128	65	37	175	12	38	43	28	14	1	4	23	159	13	0	11	15	794
d__Bacteria	k__norank_d__Bacteria	p__Gemmatimonadetes	c__Gemmatimonadetes	o__norank_c__Gemmatimonadetes	f__norank_c__Gemmatimonadetes	g__norank_c__Gemmatimonadetes	s__uncultured_soil_bacterium_g__norank_c__Gemmatimonadetes	OTU1931	0	0	0	0	0	0	2	0	0	0	0	0	0	0	0	0	0	0	2
d__Bacteria	k__norank_d__Bacteria	p__Proteobacteria	c__Alphaproteobacteria	o__Rhodospirillales	f__Rhodospirillaceae	g__norank_f__Rhodospirillaceae	s__uncultured_eubacterium_WD229	OTU3357	60	67	50	32	27	13	3	0	1	37	11	14	62	96	76	0	4	1	554
d__Bacteria	k__norank_d__Bacteria	p__Firmicutes	c__Bacilli	o__Bacillales	f__Paenibacillaceae	g__Paenibacillus	s__Paenibacillus_odorifer	OTU6054	1	1	1	2	0	1	793	238	582	2	1	2	0	3	3	102	522	488	2742
d__Bacteria	k__norank_d__Bacteria	p__Acidobacteria	c__Acidobacteria	o__norank_c__Acidobacteria	f__norank_c__Acidobacteria	g__norank_c__Acidobacteria	s__uncultured_Acidobacteriales_bacterium_g__norank_c__Acidobacteria	OTU3988	13	18	13	49	24	19	11	9	9	38	34	36	12	9	16	2	8	4	324
d__Bacteria	k__norank_d__Bacteria	p__Actinobacteria	c__Actinobacteria	o__Frankiales	f__Geodermatophilaceae	g__Geodermatophilus	s__unclassified_g__Geodermatophilus	OTU2626	14	10	11	10	28	11	2	9	8	23	19	27	5	14	10	1	23	5	230
d__Bacteria	k__norank_d__Bacteria	p__unclassified_k__norank_d__Bacteria	c__unclassified_k__norank_d__Bacteria	o__unclassified_k__norank_d__Bacteria	f__unclassified_k__norank_d__Bacteria	g__unclassified_k__norank_d__Bacteria	s__unclassified_k__norank_d__Bacteria	OTU2200	0	0	0	0	0	0	0	0	3	0	0	0	0	0	0	0	0	0	3
[illegible]	[illegible]	[illegible]	[illegible]	[illegible]	[illegible]	[illegible]	[illegible]	[illegible]	[illegible]	[illegible]	[illegible]	[illegible]	[illegible]	[illegible]	[illegible]	[illegible]	[illegible]	[illegible]	[illegible]	[illegible]	[illegible]	[illegible]	[illegible]	[illegible]	[illegible]	[illegible]	[illegible]

d__Bacteria	k__norank_d__Bacteria	p__Planctomycetes	c__Planctomycetacia	o__Planctomycetales	f__Planctomycetaceae	g__norank_f__Planctomycetaceae	s__unclassified_g__norank_f__Planctomycetaceae	OTU3994	0	0	0	0	0	0	0	0	0	1	1	0	0	0	0	0	0	0	2
d__Bacteria	k__norank_d__Bacteria	p__Bacteroidetes	c__Cytophagia	o__Cytophagales	f__Cytophagaceae	g__unclassified_f__Cytophagaceae	s__unclassified_f__Cytophagaceae	OTU700	0	0	2	0	0	0	0	0	0	0	0	0	0	0	0	0	0	0	2
d__Bacteria	k__norank_d__Bacteria	p__Proteobacteria	c__Alphaproteobacteria	o__Rhodospirillales	f__unclassified_o__Rhodospirillales	g__unclassified_o__Rhodospirillales	s__unclassified_o__Rhodospirillales	OTU5359	0	0	0	0	0	0	0	0	0	0	0	0	0	0	2	0	0	0	2
d__Bacteria	k__norank_d__Bacteria	p__Chlamydiae	c__Chlamydiae	o__Chlamydiales	f__unclassified_o__Chlamydiales	g__unclassified_o__Chlamydiales	s__unclassified_o__Chlamydiales	OTU5430	0	0	0	2	1	0	0	0	0	1	0	0	0	0	1	0	2	0	7
d__Bacteria	k__norank_d__Bacteria	p__Acidobacteria	c__Acidobacteria	o__norank_c__Acidobacteria	f__norank_c__Acidobacteria	g__norank_c__Acidobacteria	s__bacterium_Ellin7509	OTU2520	58	53	63	75	62	53	18	15	32	59	79	43	52	59	60	7	17	22	827
d__Bacteria	k__norank_d__Bacteria	p__Bacteroidetes	c__Flavobacteriia	o__Flavobacteriales	f__Flavobacteriaceae	g__Flavobacterium	s__unclassified_g__Flavobacterium	OTU621	0	0	6	0	0	0	0	0	0	0	0	0	0	0	0	0	0	0	6
d__Bacteria	k__norank_d__Bacteria	p__Planctomycetes	c__Planctomycetacia	o__Planctomycetales	f__Planctomycetaceae	g__norank_f__Planctomycetaceae	s__unclassified_g__norank_f__Planctomycetaceae	OTU980	0	0	0	2	0	0	2	0	0	0	0	1	0	0	0	0	0	0	5
d__Bacteria	k__norank_d__Bacteria	p__Proteobacteria	c__Gammaproteobacteria	o__Xanthomonadales	f__norank_o__Xanthomonadales	g__norank_o__Xanthomonadales	s__uncultured_bacterium_g__norank_o__Xanthomonadales	OTU5493	1	6	2	4	4	1	1	0	2	1	9	3	2	3	9	1	2	3	54
d__Bacteria	k__norank_d__Bacteria	p__Proteobacteria	c__Gammaproteobacteria	o__Legionellales	f__Coxiellaceae	g__Aquicella	s__uncultured_bacterium_KF-JG30-B15	OTU5697	0	0	0	0	0	0	0	1	0	0	0	0	0	1	1	0	0	0	3
d__Bacteria	k__norank_d__Bacteria	p__Chloroflexi	c__Ktedonobacteria	o__Ktedonobacterales	f__Thermosporotrichaceae	g__Thermosporothrix	s__uncultured_bacterium_g__Thermosporothrix	OTU4633	0	0	0	0	0	0	0	0	0	0	0	0	2	0	0	0	0	0	2
d__Bacteria	k__norank_d__Bacteria	p__Proteobacteria	c__Alphaproteobacteria	o__Rhizobiales	f__Rhizobiaceae	g__Rhizobium	s__unclassified_g__Rhizobium	OTU4736	0	0	0	0	0	0	0	0	0	0	0	0	2	0	0	0	0	0	2
d__Bacteria	k__norank_d__Bacteria	p__Armatimonadetes	c__Fimbriimonadia	o__Fimbriimonadales	f__Fimbriimonadaceae	g__norank_f__Fimbriimonadaceae	s__unclassified_g__norank_f__Fimbriimonadaceae	OTU3807	3	4	4	5	9	3	1	1	0	4	11	2	2	3	3	0	0	2	57
d__Bacteria	k__norank_d__Bacteria	p__Chloroflexi	c__Anaerolineae	o__Anaerolineales	f__Anaerolineaceae	g__norank_f__Anaerolineaceae	s__unclassified_g__norank_f__Anaerolineaceae	OTU3234	21	7	21	32	11	6	12	4	4	30	61	18	23	17	24	3	8	5	307
d__Bacteria	k__norank_d__Bacteria	p__Proteobacteria	c__Alphaproteobacteria	o__Rickettsiales	f__SM2D12	g__norank_f__SM2D12	s__unclassified_g__norank_f__SM2D12	OTU4903	2	1	0	3	1	6	0	0	0	0	0	1	0	1	2	0	2	0	19
d__Bacteria	k__norank_d__Bacteria	p__Proteobacteria	c__Alphaproteobacteria	o__unclassified_c__Alphaproteobacteria	f__unclassified_c__Alphaproteobacteria	g__unclassified_c__Alphaproteobacteria	s__unclassified_c__Alphaproteobacteria	OTU144	1	0	0	0	0	1	0	0	0	0	0	0	0	0	0	0	0	0	2
d__Bacteria	k__norank_d__Bacteria	p__Proteobacteria	c__Gammaproteobacteria	o__HTA4	f__norank_o__HTA4	g__norank_o__HTA4	s__uncultured_soil_bacterium_g__norank_o__HTA4	OTU3028	3	2	5	8	3	0	2	1	3	28	6	5	7	2	5	5	3	3	91
d__Bacteria	k__norank_d__Bacteria	p__Bacteroidetes	c__Sphingobacteriia	o__Sphingobacteriales	f__Chitinophagaceae	g__Niastella	s__unclassified_g__Niastella	OTU4615	0	44	6	3	0	0	4	19	0	0	0	1	18	1	0	0	0	1	97
d__Bacteria	k__norank_d__Bacteria	p__Chloroflexi	c__Ktedonobacteria	o__Ktedonobacterales	f__unclassified_o__Ktedonobacterales	g__unclassified_o__Ktedonobacterales	s__unclassified_o__Ktedonobacterales	OTU2130	0	0	0	0	0	0	0	2	0	0	0	0	0	0	0	0	0	0	2
d__Bacteria	k__norank_d__Bacteria	p__Proteobacteria	c__Deltaproteobacteria	o__Myxococcales	f__Polyangiaceae	g__Sorangium	s__uncultured_bacterium_g__Sorangium	OTU1933	0	0	0	0	0	1	1	0	1	2	0	0	0	0	1	0	0	0	6
d__Bacteria	k__norank_d__Bacteria	p__Parcubacteria	c__norank_p__Parcubacteria	o__norank_p__Parcubacteria	f__norank_p__Parcubacteria	g__norank_p__Parcubacteria	s__uncultured_bacterium_g__norank_p__Parcubacteria	OTU3969	0	0	0	0	0	0	0	0	0	0	1	0	0	0	0	0	0	0	1
d__Bacteria	k__norank_d__Bacteria	p__Gemmatimonadetes	c__Gemmatimonadetes	o__Gemmatimonadales	f__Gemmatimonadaceae	g__Gemmatimonas	s__unclassified_g__Gemmatimonas	OTU1127	0	0	0	1	0	0	0	0	0	0	0	0	2	0	0	0	0	0	3
d__Bacteria	k__norank_d__Bacteria	p__Firmicutes	c__Clostridia	o__Clostridiales	f__Clostridiaceae_1	g__Clostridium_sensu_stricto_6	s__Clostridium_bornimense	OTU157	1	0	0	0	0	0	1	0	0	0	0	0	0	0	0	0	0	0	2
d__Bacteria	k__norank_d__Bacteria	p__Proteobacteria	c__unclassified_p__Proteobacteria	o__unclassified_p__Proteobacteria	f__unclassified_p__Proteobacteria	g__unclassified_p__Proteobacteria	s__unclassified_p__Proteobacteria	OTU2247	0	0	0	0	0	0	0	0	2	0	0	0	0	0	0	0	0	0	2
d__Bacteria	k__norank_d__Bacteria	p__Actinobacteria	c__Actinobacteria	o__Frankiales	f__Frankiaceae	g__Jatrophihabitans	s__unclassified_g__Jatrophihabitans	OTU2539	7	7	3	8	2	5	4	8	2	6	3	2	5	6	2	1	2	4	77
d__Bacteria	k__norank_d__Bacteria	p__Proteobacteria	c__Deltaproteobacteria	o__Bdellovibrionales	f__Bdellovibrionaceae	g__Bdellovibrio	s__unclassified_g__Bdellovibrio	OTU971	3	0	2	2	2	0	0	0	0	0	0	0	0	0	0	0	0	0	9
d__Bacteria	k__norank_d__Bacteria	p__Ignavibacteriae	c__Ignavibacteria	o__Ignavibacteriales	f__BSV26	g__norank_f__BSV26	s__uncultured_bacterium_g__norank_f__BSV26	OTU3548	0	0	3	3	0	0	0	0	0	0	1	0	0	0	0	0	0	0	7
d__Bacteria	k__norank_d__Bacteria	p__Proteobacteria	c__Alphaproteobacteria	o__Rhodospirillales	f__DA111	g__norank_f__DA111	s__unclassified_g__norank_f__DA111	OTU3236	0	0	1	10	2	3	0	0	0	4	4	0	0	1	1	0	0	0	26
d__Bacteria	k__norank_d__Bacteria	p__Proteobacteria	c__Gammaproteobacteria	o__unclassified_c__Gammaproteobacteria	f__unclassified_c__Gammaproteobacteria	g__unclassified_c__Gammaproteobacteria	s__unclassified_c__Gammaproteobacteria	OTU5501	0	1	0	0	0	1	0	0	0	0	0	0	0	0	1	0	0	0	3
d__Bacteria	k__norank_d__Bacteria	p__Planctomycetes	c__Planctomycetacia	o__Planctomycetales	f__Planctomycetaceae	g__norank_f__Planctomycetaceae	s__unclassified_g__norank_f__Planctomycetaceae	OTU5742	0	0	0	1	0	0	0	0	1	0	0	0	0	0	0	0	0	2	4
d__Bacteria	k__norank_d__Bacteria	p__Planctomycetes	c__Planctomycetacia	o__Planctomycetales	f__Planctomycetaceae	g__norank_f__Planctomycetaceae	s__unclassified_g__norank_f__Planctomycetaceae	OTU1043	0	0	0	4	0	0	0	0	0	0	0	0	0	0	0	0	0	0	4
d__Bacteria	k__norank_d__Bacteria	p__Planctomycetes	c__Planctomycetacia	o__Planctomycetales	f__Planctomycetaceae	g__norank_f__Planctomycetaceae	s__unclassified_g__norank_f__Planctomycetaceae	OTU1045	1	2	0	1	0	0	0	0	0	0	0	0	0	0	0	0	0	0	4
d__Bacteria	k__norank_d__Bacteria	p__Firmicutes	c__Bacilli	o__Bacillales	f__Paenibacillaceae	g__Paenibacillus	s__unclassified_g__Paenibacillus	OTU4765	2	0	4	0	3	0	0	0	0	1	0	0	2	4	2	0	0	0	18
d__Bacteria	k__norank_d__Bacteria	p__Chloroflexi	c__Anaerolineae	o__Anaerolineales	f__Anaerolineaceae	g__norank_f__Anaerolineaceae	s__uncultured_Gemmatimonadetes_bacterium_g__norank_f__Anaerolineaceae	OTU1605	0	0	1	0	1	0	0	0	0	0	0	0	0	0	1	0	0	0	3
d__Bacteria	k__norank_d__Bacteria	p__Proteobacteria	c__Gammaproteobacteria	o__Xanthomonadales	f__unclassified_o__Xanthomonadales	g__unclassified_o__Xanthomonadales	s__unclassified_o__Xanthomonadales	OTU646	2	0	7	3	3	3	0	0	0	6	3	4	3	2	0	0	0	0	36
d__Bacteria	k__norank_d__Bacteria	p__Proteobacteria	c__Alphaproteobacteria	o__Rhodospirillales	f__Rhodospirillaceae	g__norank_f__Rhodospirillaceae	s__uncultured_Rhodospirillaceae_bacterium_g__norank_f__Rhodospirillaceae	OTU3463	7	3	6	18	12	18	4	3	3	12	8	14	5	5	8	1	2	5	134
d__Bacteria	k__norank_d__Bacteria	p__Chloroflexi	c__Ktedonobacteria	o__B12-WMSP1	f__norank_o__B12-WMSP1	g__norank_o__B12-WMSP1	s__unclassified_g__norank_o__B12-WMSP1	OTU2047	0	0	0	6	6	2	2	0	1	7	4	4	2	0	0	1	2	3	40
d__Bacteria	k__norank_d__Bacteria	p__unclassified_k__norank_d__Bacteria	c__unclassified_k__norank_d__Bacteria	o__unclassified_k__norank_d__Bacteria	f__unclassified_k__norank_d__Bacteria	g__unclassified_k__norank_d__Bacteria	s__unclassified_k__norank_d__Bacteria	OTU1716	0	0	3	1	0	2	1	0	0	0	0	1	0	0	1	0	0	0	9
d__Bacteria	k__norank_d__Bacteria	p__Proteobacteria	c__Alphaproteobacteria	o__Rickettsiales	f__Mitochondria	g__norank_f__Mitochondria	s__unclassified_g__norank_f__Mitochondria	OTU2766	0	1	2	5	2	1	0	0	0	2	0	2	0	0	1	0	0	0	16
d__Bacteria	k__norank_d__Bacteria	p__Chlamydiae	c__Chlamydiae	o__Chlamydiales	f__Simkaniaceae	g__norank_f__Simkaniaceae	s__unclassified_g__norank_f__Simkaniaceae	OTU5810	1	0	0	0	0	0	0	0	0	0	0	0	0	0	0	1	0	0	2
d__Bacteria	k__norank_d__Bacteria	p__unclassified_k__norank_d__Bacteria	c__unclassified_k__norank_d__Bacteria	o__unclassified_k__norank_d__Bacteria	f__unclassified_k__norank_d__Bacteria	g__unclassified_k__norank_d__Bacteria	s__unclassified_k__norank_d__Bacteria	OTU5429	0	0	0	0	3	2	4	0	0	5	0	4	2	1	4	0	0	1	26
d__Bacteria	k__norank_d__Bacteria	p__unclassified_k__norank_d__Bacteria	c__unclassified_k__norank_d__Bacteria	o__unclassified_k__norank_d__Bacteria	f__unclassified_k__norank_d__Bacteria	g__unclassified_k__norank_d__Bacteria	s__unclassified_k__norank_d__Bacteria	OTU5428	1	0	0	0	1	0	0	0	0	0	0	0	0	0	1	0	0	0	3
d__Bacteria	k__norank_d__Bacteria	p__Chloroflexi	c__Ktedonobacteria	o__JG30-KF-AS9	f__norank_o__JG30-KF-AS9	g__norank_o__JG30-KF-AS9	s__uncultured_bacterium_g__norank_o__JG30-KF-AS9	OTU4238	39	39	64	57	33	25	18	20	30	26	50	25	20	44	20	13	15	23	561
d__Bacteria	k__norank_d__Bacteria	p__Firmicutes	c__Bacilli	o__Bacillales	f__Bacillaceae	g__Falsibacillus	s__Bacillus_sp._mixed_culture_J1-55	OTU5756	0	0	0	0	0	0	54	90	85	0	0	0	0	0	0	59	168	77	533
d__Bacteria	k__norank_d__Bacteria	p__Bacteroidetes	c__Sphingobacteriia	o__Sphingobacteriales	f__Sphingobacteriaceae	g__Sphingobacterium	s__Sphingobacterium_bambusae	OTU5938	0	0	0	0	0	0	0	0	0	0	0	0	0	0	0	0	11	0	11
d__Bacteria	k__norank_d__Bacteria	p__unclassified_k__norank_d__Bacteria	c__unclassified_k__norank_d__Bacteria	o__unclassified_k__norank_d__Bacteria	f__unclassified_k__norank_d__Bacteria	g__unclassified_k__norank_d__Bacteria	s__unclassified_k__norank_d__Bacteria	OTU1863	0	0	0	0	0	2	0	0	0	0	0	0	0	0	0	0	0	0	2
d__Bacteria	k__norank_d__Bacteria	p__TM6__Dependentiae_	c__norank_p__TM6__Dependentiae_	o__norank_p__TM6__Dependentiae_	f__norank_p__TM6__Dependentiae_	g__norank_p__TM6__Dependentiae_	s__unclassified_g__norank_p__TM6__Dependentiae_	OTU5179	0	0	0	0	1	0	0	0	2	0	0	0	0	0	3	0	0	0	6
d__Bacteria	k__norank_d__Bacteria	p__Firmicutes	c__Bacilli	o__Bacillales	f__Bacillaceae	g__Bacillus	s__unclassified_g__Bacillus	OTU759	0	3	3	3	0	1	4	7	11	2	0	1	3	0	4	2	20	15	79
d__Bacteria	k__norank_d__Bacteria	p__Proteobacteria	c__Alphaproteobacteria	o__Rhodospirillales	f__Rhodospirillaceae	g__unclassified_f__Rhodospirillaceae	s__unclassified_f__Rhodospirillaceae	OTU3140	0	0	1	0	2	0	0	0	0	0	2	2	0	0	0	0	0	1	8
d__Bacteria	k__norank_d__Bacteria	p__Chloroflexi	c__JG37-AG-4	o__norank_c__JG37-AG-4	f__norank_c__JG37-AG-4	g__norank_c__JG37-AG-4	s__unclassified_g__norank_c__JG37-AG-4	OTU4646	0	0	0	0	5	1	0	0	0	0	0	0	1	0	0	0	0	0	7
d__Bacteria	k__norank_d__Bacteria	p__unclassified_k__norank_d__Bacteria	c__unclassified_k__norank_d__Bacteria	o__unclassified_k__norank_d__Bacteria	f__unclassified_k__norank_d__Bacteria	g__unclassified_k__norank_d__Bacteria	s__unclassified_k__norank_d__Bacteria	OTU3441	0	6	0	4	10	5	1	0	2	6	6	7	2	1	1	0	1	2	54
d__Bacteria	k__norank_d__Bacteria	p__unclassified_k__norank_d__Bacteria	c__unclassified_k__norank_d__Bacteria	o__unclassified_k__norank_d__Bacteria	f__unclassified_k__norank_d__Bacteria	g__unclassified_k__norank_d__Bacteria	s__unclassified_k__norank_d__Bacteria	OTU3442	2	0	0	3	7	2	2	1	0	4	2	3	2	0	0	0	0	1	29
d__Bacteria	k__norank_d__Bacteria	p__unclassified_k__norank_d__Bacteria	c__unclassified_k__norank_d__Bacteria	o__unclassified_k__norank_d__Bacteria	f__unclassified_k__norank_d__Bacteria	g__unclassified_k__norank_d__Bacteria	s__unclassified_k__norank_d__Bacteria	OTU3113	1	0	1	0	7	4	1	1	2	0	2	1	2	0	0	0	0	1	23
d__Bacteria	k__norank_d__Bacteria	p__unclassified_k__norank_d__Bacteria	c__unclassified_k__norank_d__Bacteria	o__unclassified_k__norank_d__Bacteria	f__unclassified_k__norank_d__Bacteria	g__unclassified_k__norank_d__Bacteria	s__unclassified_k__norank_d__Bacteria	OTU3447	5	11	6	8	8	5	0	0	0	6	4	6	4	4	5	1	0	0	73
d__Bacteria	k__norank_d__Bacteria	p__Proteobacteria	c__Gammaproteobacteria	o__unclassified_c__Gammaproteobacteria	f__unclassified_c__Gammaproteobacteria	g__unclassified_c__Gammaproteobacteria	s__unclassified_c__Gammaproteobacteria	OTU3229	1	8	2	13	7	19	0	0	0	6	2	1	1	0	0	0	0	0	60
d__Bacteria	k__norank_d__Bacteria	p__Chloroflexi	c__Ktedonobacteria	o__Thermogemmatisporales	f__1921-2	g__norank_f__1921-2	s__uncultured_bacterium_g__norank_f__1921-2	OTU4852	0	0	2	1	1	0	2	0	0	0	2	3	0	8	0	0	0	0	19
d__Bacteria	k__norank_d__Bacteria	p__Verrucomicrobia	c__OPB35_soil_group	o__norank_c__OPB35_soil_group	f__norank_c__OPB35_soil_group	g__norank_c__OPB35_soil_group	s__uncultured_Verrucomicrobia_bacterium_g__norank_c__OPB35_soil_group	OTU281	0	3	0	0	0	0	0	0	0	0	0	0	0	0	0	0	0	0	3
d__Bacteria	k__norank_d__Bacteria	p__Chloroflexi	c__Caldilineae	o__Caldilineales	f__Caldilineaceae	g__norank_f__Caldilineaceae	s__unclassified_g__norank_f__Caldilineaceae	OTU1502	1	0	1	1	2	1	0	0	0	1	2	0	0	0	0	0	0	2	11
d__Bacteria	k__norank_d__Bacteria	p__Armatimonadetes	c__Chthonomonadetes	o__Chthonomonadales	f__Chthonomonadaceae	g__Chthonomonas	s__uncultured_Armatimonadetes_bacterium_g__Chthonomonas	OTU2376	3	4	4	5	1	1	0	1	2	2	4	6	3	1	3	1	1	4	46
d__Bacteria	k__norank_d__Bacteria	p__Proteobacteria	c__Alphaproteobacteria	o__Rhizobiales	f__Rhizobiales_Incertae_Sedis	g__Phreatobacter	s__unclassified_g__Phreatobacter	OTU1930	0	0	0	0	0	0	4	0	0	0	0	0	0	0	0	0	0	0	4
d__Bacteria	k__norank_d__Bacteria	p__Chloroflexi	c__Ktedonobacteria	o__Ktedonobacterales	f__FCPS473	g__norank_f__FCPS473	s__uncultured_Ktedonobacter_sp._g__norank_f__FCPS473	OTU5624	0	0	0	0	1	2	0	0	0	0	1	0	2	0	2	2	0	0	10
d__Bacteria	k__norank_d__Bacteria	p__Actinobacteria	c__Actinobacteria	o__Micrococcales	f__unclassified_o__Micrococcales	g__unclassified_o__Micrococcales	s__unclassified_o__Micrococcales	OTU1943	0	0	0	0	0	0	1	0	0	0	0	0	0	0	0	0	0	0	1
d__Bacteria	k__norank_d__Bacteria	p__Gemmatimonadetes	c__Gemmatimonadetes	o__Gemmatimonadales	f__Gemmatimonadaceae	g__unclassified_f__Gemmatimonadaceae	s__unclassified_f__Gemmatimonadaceae	OTU4824	45	4	26	1	2	0	1	0	0	0	0	0	1	6	0	0	1	0	87
d__Bacteria	k__norank_d__Bacteria	p__Verrucomicrobia	c__OPB35_soil_group	o__norank_c__OPB35_soil_group	f__norank_c__OPB35_soil_group	g__norank_c__OPB35_soil_group	s__uncultured_bacterium_g__norank_c__OPB35_soil_group	OTU3976	21	16	12	21	16	11	3	0	1	6	12	10	28	11	19	0	5	0	192
d__Bacteria	k__norank_d__Bacteria	p__Saccharibacteria	c__norank_p__Saccharibacteria	o__norank_p__Saccharibacteria	f__norank_p__Saccharibacteria	g__norank_p__Saccharibacteria	s__unclassified_g__norank_p__Saccharibacteria	OTU2892	0	0	0	1	0	0	0	0	0	2	0	0	0	0	0	0	0	0	3
d__Bacteria	k__norank_d__Bacteria	p__Acidobacteria	c__Acidobacteria	o__norank_c__Acidobacteria	f__norank_c__Acidobacteria	g__norank_c__Acidobacteria	s__uncultured_forest_soil_bacterium_g__norank_c__Acidobacteria	OTU4251	1	0	4	4	1	3	0	1	0	1	1	2	0	1	4	0	0	0	23
d__Bacteria	k__norank_d__Bacteria	p__Chloroflexi	c__Ktedonobacteria	o__Ktedonobacterales	f__HSB_OF53-F07	g__norank_f__HSB_OF53-F07	s__unclassified_g__norank_f__HSB_OF53-F07	OTU4098	0	0	1	3	0	0	0	3	0	1	1	1	0	1	0	2	0	0	13
d__Bacteria	k__norank_d__Bacteria	p__TM6__Dependentiae_	c__norank_p__TM6__Dependentiae_	o__norank_p__TM6__Dependentiae_	f__norank_p__TM6__Dependentiae_	g__norank_p__TM6__Dependentiae_	s__unclassified_g__norank_p__TM6__Dependentiae_	OTU1279	0	0	0	3	0	0	0	0	0	0	0	0	0	0	0	0	0	0	3
d__Bacteria	k__norank_d__Bacteria	p__Bacteroidetes	c__Sphingobacteriia	o__Sphingobacteriales	f__Chitinophagaceae	g__norank_f__Chitinophagaceae	s__uncultured_bacterium_g__norank_f__Chitinophagaceae	OTU2337	4	19	42	12	15	8	0	7	3	13	0	15	5	13	19	0	0	1	176
d__Bacteria	k__norank_d__Bacteria	p__Verrucomicrobia	c__Opitutae	o__Opitutales	f__Opitutaceae	g__Opitutus	s__Hypsibius_dujardini_g__Opitutus	OTU5222	35	152	42	9	134	28	0	2	0	7	1	1	28	12	16	0	0	0	467
d__Bacteria	k__norank_d__Bacteria	p__Proteobacteria	c__Deltaproteobacteria	o__Desulfurellales	f__Desulfurellaceae	g__H16	s__uncultured_bacterium_g__H16	OTU3513	1	4	0	3	0	0	0	0	0	2	3	1	0	1	0	0	0	0	15
d__Bacteria	k__norank_d__Bacteria	p__Bacteroidetes	c__Sphingobacteriia	o__Sphingobacteriales	f__norank_o__Sphingobacteriales	g__norank_o__Sphingobacteriales	s__uncultured_bacterium_g__norank_o__Sphingobacteriales	OTU3470	3	3	1	4	2	3	0	0	0	5	2	6	0	0	0	0	0	0	29
d__Bacteria	k__norank_d__Bacteria	p__Proteobacteria	c__Alphaproteobacteria	o__Rhodospirillales	f__DA111	g__norank_f__DA111	s__unclassified_g__norank_f__DA111	OTU2675	1	0	0	0	1	2	1	0	0	3	0	1	0	1	1	2	0	0	13
d__Bacteria	k__norank_d__Bacteria	p__unclassified_k__norank_d__Bacteria	c__unclassified_k__norank_d__Bacteria	o__unclassified_k__norank_d__Bacteria	f__unclassified_k__norank_d__Bacteria	g__unclassified_k__norank_d__Bacteria	s__unclassified_k__norank_d__Bacteria	OTU4465	11	42	12	1	1	1	8	6	0	1	2	2	15	24	3	0	2	8	139
d__Bacteria	k__norank_d__Bacteria	p__Proteobacteria	c__Deltaproteobacteria	o__Myxococcales	f__Polyangiaceae	g__Labilithrix	s__Labilithrix_luteola	OTU5833	0	0	0	0	0	1	0	1	0	0	0	1	0	0	0	0	4	0	7
d__Bacteria	k__norank_d__Bacteria	p__Proteobacteria	c__Betaproteobacteria	o__Burkholderiales	f__Comamonadaceae	g__unclassified_f__Comamonadaceae	s__unclassified_f__Comamonadaceae	OTU830	0	0	3	0	0	0	0	0	0	0	0	0	1	0	1	0	0	1	6
d__Bacteria	k__norank_d__Bacteria	p__Proteobacteria	c__Gammaproteobacteria	o__Legionellales	f__Coxiellaceae	g__Aquicella	s__uncultured_Aquicella_sp.	OTU5206	1	3	2	6	1	1	6	4	4	11	3	3	2	6	8	4	4	0	69
d__Bacteria	k__norank_d__Bacteria	p__unclassified_k__norank_d__Bacteria	c__unclassified_k__norank_d__Bacteria	o__unclassified_k__norank_d__Bacteria	f__unclassified_k__norank_d__Bacteria	g__unclassified_k__norank_d__Bacteria	s__unclassified_k__norank_d__Bacteria	OTU671	1	0	2	0	0	0	0	0	0	0	0	0	0	0	0	0	0	0	3
d__Bacteria	k__norank_d__Bacteria	p__Firmicutes	c__Clostridia	o__Clostridiales	f__Ruminococcaceae	g__unclassified_f__Ruminococcaceae	s__unclassified_f__Ruminococcaceae	OTU4509	0	0	0	0	0	0	0	0	0	0	0	2	0	0	0	0	0	0	2
d__Bacteria	k__norank_d__Bacteria	p__Proteobacteria	c__Alphaproteobacteria	o__unclassified_c__Alphaproteobacteria	f__unclassified_c__Alphaproteobacteria	g__unclassified_c__Alphaproteobacteria	s__unclassified_c__Alphaproteobacteria	OTU5817	0	0	0	0	0	0	0	0	0	0	0	0	0	0	0	2	0	0	2
d__Bacteria	k__norank_d__Bacteria	p__Proteobacteria	c__Alphaproteobacteria	o__Rhodospirillales	f__Rhodospirillaceae	g__Azospirillum	s__Azospirillum_zeae	OTU5745	0	1	0	0	0	0	1	0	0	0	0	0	0	0	0	0	0	0	2
d__Bacteria	k__norank_d__Bacteria	p__unclassified_k__norank_d__Bacteria	c__unclassified_k__norank_d__Bacteria	o__unclassified_k__norank_d__Bacteria	f__unclassified_k__norank_d__Bacteria	g__unclassified_k__norank_d__Bacteria	s__unclassified_k__norank_d__Bacteria	OTU5847	2	0	10	0	2	2	0	2	3	2	0	0	0	1	1	1	9	3	38
d__Bacteria	k__norank_d__Bacteria	p__Acidobacteria	c__Acidobacteria	o__Solibacterales	f__Solibacteraceae__Subgroup_3_	g__Bryobacter	s__unclassified_g__Bryobacter	OTU4155	99	61	89	95	94	60	45	16	26	98	133	151	62	134	116	7	25	32	1343
d__Bacteria	k__norank_d__Bacteria	p__unclassified_k__norank_d__Bacteria	c__unclassified_k__norank_d__Bacteria	o__unclassified_k__norank_d__Bacteria	f__unclassified_k__norank_d__Bacteria	g__unclassified_k__norank_d__Bacteria	s__unclassified_k__norank_d__Bacteria	OTU5842	0	0	0	0	0	0	0	0	0	0	0	0	0	0	0	0	1	1	2
d__Bacteria	k__norank_d__Bacteria	p__Verrucomicrobia	c__OPB35_soil_group	o__norank_c__OPB35_soil_group	f__norank_c__OPB35_soil_group	g__norank_c__OPB35_soil_group	s__uncultured_bacterium_g__norank_c__OPB35_soil_group	OTU4192	27	2	4	13	4	3	0	0	0	5	9	4	2	3	20	0	0	0	96
d__Bacteria	k__norank_d__Bacteria	p__Acidobacteria	c__Acidobacteria	o__Acidobacteriales	f__Acidobacteriaceae__Subgroup_1_	g__norank_f__Acidobacteriaceae__Subgroup_1_	s__uncultured_Acidobacteriales_bacterium_g__norank_f__Acidobacteriaceae__Subgroup_1_	OTU2716	0	2	4	3	0	1	0	1	1	3	1	1	2	3	6	0	1	1	30
d__Bacteria	k__norank_d__Bacteria	p__Proteobacteria	c__Gammaproteobacteria	o__HTA4	f__norank_o__HTA4	g__norank_o__HTA4	s__uncultured_bacterium_g__norank_o__HTA4	OTU3652	1	1	0	1	0	1	1	0	0	0	2	0	1	3	1	0	0	0	12
d__Bacteria	k__norank_d__Bacteria	p__Proteobacteria	c__Alphaproteobacteria	o__Rhodospirillales	f__DA111	g__norank_f__DA111	s__uncultured_bacterium_g__norank_f__DA111	OTU3161	7	1	4	11	6	10	0	0	1	12	10	10	2	5	9	0	2	0	90
d__Bacteria	k__norank_d__Bacteria	p__Actinobacteria	c__Actinobacteria	o__Acidimicrobiales	f__norank_o__Acidimicrobiales	g__norank_o__Acidimicrobiales	s__bacterium_enrichment_culture_clone_auto73_4W	OTU3558	0	0	0	0	0	0	0	0	0	0	2	0	0	0	0	0	0	0	2
d__Bacteria	k__norank_d__Bacteria	p__Acidobacteria	c__Acidobacteria	o__Acidobacteriales	f__Acidobacteriaceae__Subgroup_1_	g__norank_f__Acidobacteriaceae__Subgroup_1_	s__unclassified_g__norank_f__Acidobacteriaceae__Subgroup_1_	OTU4100	261	173	251	293	241	327	83	41	104	323	330	537	272	335	419	32	170	82	4274
d__Bacteria	k__norank_d__Bacteria	p__TM6__Dependentiae_	c__norank_p__TM6__Dependentiae_	o__norank_p__TM6__Dependentiae_	f__norank_p__TM6__Dependentiae_	g__norank_p__TM6__Dependentiae_	s__unclassified_g__norank_p__TM6__Dependentiae_	OTU927	0	0	1	3	0	0	0	0	0	0	0	0	0	0	0	0	0	0	4
d__Bacteria	k__norank_d__Bacteria	p__Proteobacteria	c__Gammaproteobacteria	o__Legionellales	f__Coxiellaceae	g__norank_f__Coxiellaceae	s__unclassified_g__norank_f__Coxiellaceae	OTU2185	0	0	0	0	0	0	0	0	2	0	0	0	0	1	0	0	0	0	3
d__Bacteria	k__norank_d__Bacteria	p__Firmicutes	c__Bacilli	o__Bacillales	f__unclassified_o__Bacillales	g__unclassified_o__Bacillales	s__unclassified_o__Bacillales	OTU2155	0	0	0	0	0	0	0	2	0	0	0	0	0	0	0	0	0	0	2
d__Bacteria	k__norank_d__Bacteria	p__Proteobacteria	c__Alphaproteobacteria	o__Sphingomonadales	f__Sphingomonadaceae	g__Sphingomonas	s__unclassified_g__Sphingomonas	OTU65	1	0	0	0	0	0	0	0	0	0	0	0	0	0	0	0	0	0	1
d__Bacteria	k__norank_d__Bacteria	p__Parcubacteria	c__norank_p__Parcubacteria	o__norank_p__Parcubacteria	f__norank_p__Parcubacteria	g__norank_p__Parcubacteria	s__uncultured_bacterium_g__norank_p__Parcubacteria	OTU1609	1	4	2	7	3	0	0	0	0	0	0	0	1	1	1	0	1	0	21
d__Bacteria	k__norank_d__Bacteria	p__Proteobacteria	c__Alphaproteobacteria	o__Rickettsiales	f__unclassified_o__Rickettsiales	g__unclassified_o__Rickettsiales	s__unclassified_o__Rickettsiales	OTU687	18	6	29	0	0	2	3	0	1	2	3	0	13	5	13	0	1	0	96
d__Bacteria	k__norank_d__Bacteria	p__TM6__Dependentiae_	c__norank_p__TM6__Dependentiae_	o__norank_p__TM6__Dependentiae_	f__norank_p__TM6__Dependentiae_	g__norank_p__TM6__Dependentiae_	s__uncultured_bacterium_g__norank_p__TM6__Dependentiae_	OTU2976	0	0	1	5	1	3	0	0	0	2	4	0	2	0	4	0	1	1	24
d__Bacteria	k__norank_d__Bacteria	p__Planctomycetes	c__Planctomycetacia	o__Planctomycetales	f__Planctomycetaceae	g__norank_f__Planctomycetaceae	s__unclassified_g__norank_f__Planctomycetaceae	OTU1906	0	0	0	0	0	0	1	0	0	0	0	0	0	0	0	0	0	0	1
d__Bacteria	k__norank_d__Bacteria	p__Acidobacteria	c__Acidobacteria	o__norank_c__Acidobacteria	f__norank_c__Acidobacteria	g__norank_c__Acidobacteria	s__unclassified_g__norank_c__Acidobacteria	OTU472	3	2	4	2	0	0	0	0	0	0	2	1	1	0	0	0	0	0	15
d__Bacteria	k__norank_d__Bacteria	p__Armatimonadetes	c__Chthonomonadetes	o__Chthonomonadales	f__Chthonomonadaceae	g__Chthonomonas	s__unclassified_g__Chthonomonas	OTU4164	0	0	0	0	0	1	3	0	0	2	0	2	0	0	0	0	0	1	9
d__Bacteria	k__norank_d__Bacteria	p__Proteobacteria	c__Gammaproteobacteria	o__Legionellales	f__Coxiellaceae	g__Aquicella	s__unclassified_g__Aquicella	OTU5135	0	0	0	0	0	0	0	1	0	1	0	2	12	12	0	4	6	8	46
d__Bacteria	k__norank_d__Bacteria	p__Proteobacteria	c__Deltaproteobacteria	o__Myxococcales	f__Archangiaceae	g__Anaeromyxobacter	s__unclassified_g__Anaeromyxobacter	OTU5253	3	18	3	4	0	1	8	7	1	2	4	9	1	3	2	0	1	1	68
d__Bacteria	k__norank_d__Bacteria	p__Planctomycetes	c__Planctomycetacia	o__Planctomycetales	f__Planctomycetaceae	g__norank_f__Planctomycetaceae	s__uncultured_Gemmata_sp._g__norank	OTU3837	1	1	0	0	0	1	1	2	0	0	2	0	0	0	0	0	0	0	8
d__Bacteria	k__norank_d__Bacteria	p__Saccharibacteria	c__norank_p__Saccharibacteria	o__norank_p__Saccharibacteria	f__norank_p__Saccharibacteria	g__norank_p__Saccharibacteria	s__unclassified_g__norank_p__Saccharibacteria	OTU1236	0	0	0	2	0	0	0	0	0	0	0	0	0	0	0	0	0	0	2
d__Bacteria	k__norank_d__Bacteria	p__Acidobacteria	c__Acidobacteria	o__norank_c__Acidobacteria	f__norank_c__Acidobacteria	g__norank_c__Acidobacteria	s__unclassified_g__norank_c__Acidobacteria	OTU5544	15	2	4	6	3	11	0	0	0	20	12	4	7	2	5	0	0	0	91
d__Bacteria	k__norank_d__Bacteria	p__Chlamydiae	c__Chlamydiae	o__Chlamydiales	f__Parachlamydiaceae	g__unclassified_f__Parachlamydiaceae	s__unclassified_f__Parachlamydiaceae	OTU1694	0	0	0	0	0	4	0	0	0	0	0	0	0	0	0	0	0	0	4
d__Bacteria	k__norank_d__Bacteria	p__Parcubacteria	c__norank_p__Parcubacteria	o__norank_p__Parcubacteria	f__norank_p__Parcubacteria	g__norank_p__Parcubacteria	s__uncultured_bacterium_g__norank_p__Parcubacteria	OTU688	0	0	5	0	0	0	0	0	0	0	0	0	0	0	0	0	0	0	5
d__Bacteria	k__norank_d__Bacteria	p__Chloroflexi	c__Ktedonobacteria	o__Ktedonobacterales	f__unclassified_o__Ktedonobacterales	g__unclassified_o__Ktedonobacterales	s__unclassified_o__Ktedonobacterales	OTU3949	461	244	463	689	752	813	98	74	168	656	851	795	430	496	587	42	233	158	8010
d__Bacteria	k__norank_d__Bacteria	p__Parcubacteria	c__norank_p__Parcubacteria	o__norank_p__Parcubacteria	f__norank_p__Parcubacteria	g__norank_p__Parcubacteria	s__uncultured_bacterium_g__norank_p__Parcubacteria	OTU682	2	0	2	0	0	0	0	0	0	0	0	0	0	0	0	0	0	0	4
d__Bacteria	k__norank_d__Bacteria	p__Armatimonadetes	c__Chthonomonadetes	o__Chthonomonadales	f__Chthonomonadaceae	g__Chthonomonas	s__unclassified_g__Chthonomonas	OTU3921	0	0	0	0	2	0	0	0	0	0	1	0	0	0	0	0	0	0	3
d__Bacteria	k__norank_d__Bacteria	p__Proteobacteria	c__Deltaproteobacteria	o__Myxococcales	f__unclassified_o__Myxococcales	g__unclassified_o__Myxococcales	s__unclassified_o__Myxococcales	OTU6015	0	0	0	0	0	0	0	0	0	0	0	0	0	0	0	0	0	2	2
d__Bacteria	k__norank_d__Bacteria	p__Chlorobi	c__Chlorobia	o__Chlorobiales	f__OPB56	g__norank_f__OPB56	s__unclassified_g__norank_f__OPB56	OTU2024	0	0	0	0	0	0	2	0	0	0	0	0	0	0	0	0	0	0	2
d__Bacteria	k__norank_d__Bacteria	p__Acidobacteria	c__Acidobacteria	o__Subgroup_7	f__norank_o__Subgroup_7	g__norank_o__Subgroup_7	s__uncultured_bacterium_g__norank_o__Subgroup_7	OTU2352	5	3	6	12	2	0	9	8	7	3	3	1	2	5	11	0	0	6	83
d__Bacteria	k__norank_d__Bacteria	p__Chloroflexi	c__Ktedonobacteria	o__B12-WMSP1	f__norank_o__B12-WMSP1	g__norank_o__B12-WMSP1	s__uncultured_bacterium_g__norank_o__B12-WMSP1	OTU1475	0	0	0	0	2	0	0	0	0	0	0	0	0	0	0	0	0	0	2
d__Bacteria	k__norank_d__Bacteria	p__Proteobacteria	c__Betaproteobacteria	o__Nitrosomonadales	f__Nitrosomonadaceae	g__norank_f__Nitrosomonadaceae	s__unclassified_g__norank_f__Nitrosomonadaceae	OTU1617	1	2	7	4	3	4	1	4	2	2	3	4	0	1	2	0	2	1	43
d__Bacteria	k__norank_d__Bacteria	p__Proteobacteria	c__unclassified_p__Proteobacteria	o__unclassified_p__Proteobacteria	f__unclassified_p__Proteobacteria	g__unclassified_p__Proteobacteria	s__unclassified_p__Proteobacteria	OTU2651	0	0	0	0	2	0	0	0	0	4	0	0	2	0	0	0	0	0	8
d__Bacteria	k__norank_d__Bacteria	p__Actinobacteria	c__Actinobacteria	o__Solirubrobacterales	f__unclassified_o__Solirubrobacterales	g__unclassified_o__Solirubrobacterales	s__unclassified_o__Solirubrobacterales	OTU1372	0	0	0	1	0	0	0	0	0	2	0	0	0	0	1	1	0	0	5
d__Bacteria	k__norank_d__Bacteria	p__Proteobacteria	c__Gammaproteobacteria	o__Legionellales	f__Legionellaceae	g__Legionella	s__unclassified_g__Legionella	OTU2399	0	0	2	6	1	3	2	0	1	2	0	0	1	0	0	0	0	1	19
d__Bacteria	k__norank_d__Bacteria	p__Chlamydiae	c__Chlamydiae	o__Chlamydiales	f__Simkaniaceae	g__unclassified_f__Simkaniaceae	s__unclassified_f__Simkaniaceae	OTU962	0	0	2	2	0	0	0	0	0	1	0	2	0	0	1	0	0	0	8
d__Bacteria	k__norank_d__Bacteria	p__Actinobacteria	c__Actinobacteria	o__Acidimicrobiales	f__unclassified_o__Acidimicrobiales	g__unclassified_o__Acidimicrobiales	s__unclassified_o__Acidimicrobiales	OTU2873	0	0	0	1	0	0	0	0	0	2	0	0	0	0	0	0	0	0	3
d__Bacteria	k__norank_d__Bacteria	p__Actinobacteria	c__Actinobacteria	o__Micromonosporales	f__Micromonosporaceae	g__norank_f__Micromonosporaceae	s__unclassified_g__norank_f__Micromonosporaceae	OTU5678	0	0	0	0	0	0	0	0	0	0	0	0	0	1	3	0	0	0	4
d__Bacteria	k__norank_d__Bacteria	p__Proteobacteria	c__Alphaproteobacteria	o__Rhodospirillales	f__unclassified_o__Rhodospirillales	g__unclassified_o__Rhodospirillales	s__unclassified_o__Rhodospirillales	OTU1062	0	2	5	2	0	0	0	0	0	0	0	0	1	0	0	0	0	1	11
d__Bacteria	k__norank_d__Bacteria	p__Elusimicrobia	c__Elusimicrobia	o__Lineage_IV	f__norank_o__Lineage_IV	g__norank_o__Lineage_IV	s__uncultured_bacterium_g__norank_o__Lineage_IV	OTU4422	0	0	0	1	0	1	0	0	0	2	0	1	0	0	0	0	0	0	5
d__Bacteria	k__norank_d__Bacteria	p__Firmicutes	c__Clostridia	o__Halanaerobiales	f__ODP1230B8.23	g__norank_f__ODP1230B8.23	s__unclassified_g__norank_f__ODP1230B8.23	OTU376	0	5	2	6	13	3	4	1	2	15	11	19	4	11	7	2	7	5	117
d__Bacteria	k__norank_d__Bacteria	p__Chloroflexi	c__SBR2076	o__norank_c__SBR2076	f__norank_c__SBR2076	g__norank_c__SBR2076	s__uncultured_eubacterium_WCHB1-50	OTU3081	2	2	2	5	4	1	0	2	2	2	5	2	1	4	4	1	6	0	45
d__Bacteria	k__norank_d__Bacteria	p__Proteobacteria	c__Gammaproteobacteria	o__unclassified_c__Gammaproteobacteria	f__unclassified_c__Gammaproteobacteria	g__unclassified_c__Gammaproteobacteria	s__unclassified_c__Gammaproteobacteria	OTU5779	0	0	0	0	0	0	0	0	0	0	0	0	0	0	0	3	0	0	3
d__Bacteria	k__norank_d__Bacteria	p__Gemmatimonadetes	c__Gemmatimonadetes	o__Gemmatimonadales	f__Gemmatimonadaceae	g__norank_f__Gemmatimonadaceae	s__uncultured_Gemmatimonadetes_bacterium_g__norank_f__Gemmatimonadaceae	OTU2223	4	0	1	8	2	0	0	3	2	1	1	0	2	0	0	0	0	0	24
d__Bacteria	k__norank_d__Bacteria	p__Cyanobacteria	c__Cyanobacteria	o__Vampirovibrionales	f__norank_o__Vampirovibrionales	g__norank_o__Vampirovibrionales	s__uncultured_bacterium_g__norank_o__Vampirovibrionales	OTU2467	0	0	0	2	1	0	0	0	4	0	1	1	0	1	0	0	2	1	13
d__Bacteria	k__norank_d__Bacteria	p__Chloroflexi	c__Ktedonobacteria	o__Ktedonobacterales	f__1921-3	g__norank_f__1921-3	s__uncultured_bacterium_g__norank_f__1921-3	OTU3909	27	24	36	31	47	40	10	3	12	19	26	29	12	19	18	0	16	7	376
d__Bacteria	k__norank_d__Bacteria	p__Planctomycetes	c__Phycisphaerae	o__Tepidisphaerales	f__Tepidisphaeraceae	g__norank_f__Tepidisphaeraceae	s__uncultured_soil_bacterium_g__norank_f__Tepidisphaeraceae	OTU3118	3	4	1	1	0	0	0	2	2	0	5	0	0	3	1	0	0	0	22
d__Bacteria	k__norank_d__Bacteria	p__Firmicutes	c__Clostridia	o__Clostridiales	f__Ruminococcaceae	g__Ruminiclostridium_1	s__unclassified_g__Ruminiclostridium_1	OTU4523	0	0	0	0	0	0	0	0	0	0	0	2	0	0	0	0	0	0	2
d__Bacteria	k__norank_d__Bacteria	p__Firmicutes	c__Clostridia	o__Halanaerobiales	f__ODP1230B8.23	g__norank_f__ODP1230B8.23	s__uncultured_forest_soil_bacterium_g__norank_f__ODP1230B8.23	OTU1887	12	11	19	19	25	65	12	9	9	41	36	56	25	36	31	4	17	20	447
d__Bacteria	k__norank_d__Bacteria	p__Saccharibacteria	c__norank_p__Saccharibacteria	o__norank_p__Saccharibacteria	f__norank_p__Saccharibacteria	g__norank_p__Saccharibacteria	s__bacterium_LWQ8	OTU5620	0	0	0	0	1	0	0	0	0	1	0	0	0	3	1	0	0	0	6
d__Bacteria	k__norank_d__Bacteria	p__Proteobacteria	c__Gammaproteobacteria	o__Pseudomonadales	f__Moraxellaceae	g__Perlucidibaca	s__unclassified_g__Perlucidibaca	OTU3722	6	37	5	11	122	21	0	0	3	38	14	31	15	22	68	0	0	3	396
d__Bacteria	k__norank_d__Bacteria	p__Proteobacteria	c__Alphaproteobacteria	o__Rhizobiales	f__A0839	g__norank_f__A0839	s__uncultured_bacterium_g__norank_f__A0839	OTU100	5	0	1	0	0	0	0	0	0	0	0	0	0	0	0	0	0	0	6
d__Bacteria	k__norank_d__Bacteria	p__Actinobacteria	c__Actinobacteria	o__Streptosporangiales	f__Thermomonosporaceae	g__Actinoallomurus	s__unclassified_g__Actinoallomurus	OTU840	0	0	2	0	0	0	0	0	0	0	0	0	0	0	0	0	0	0	2
d__Bacteria	k__norank_d__Bacteria	p__Proteobacteria	c__Betaproteobacteria	o__Nitrosomonadales	f__Nitrosomonadaceae	g__Nitrosospira	s__unclassified_g__Nitrosospira	OTU113	2	2	0	0	0	1	0	0	0	0	0	0	0	0	0	0	0	0	5
d__Bacteria	k__norank_d__Bacteria	p__Chlamydiae	c__Chlamydiae	o__Chlamydiales	f__cvE6	g__norank_f__cvE6	s__unclassified_g__norank_f__cvE6	OTU5703	0	0	0	0	0	0	0	0	0	0	0	0	0	0	1	0	0	0	1
d__Unclassified	k__unclassified_d__Unclassified	p__unclassified_d__Unclassified	c__unclassified_d__Unclassified	o__unclassified_d__Unclassified	f__unclassified_d__Unclassified	g__unclassified_d__Unclassified	s__unclassified_d__Unclassified	OTU2866	0	0	1	0	0	0	0	0	0	2	0	0	0	0	0	0	0	0	3
d__Unclassified	k__unclassified_d__Unclassified	p__unclassified_d__Unclassified	c__unclassified_d__Unclassified	o__unclassified_d__Unclassified	f__unclassified_d__Unclassified	g__unclassified_d__Unclassified	s__unclassified_d__Unclassified	OTU706	0	0	3	0	0	0	1	2	0	0	1	0	0	2	0	0	4	0	13
d__Unclassified	k__unclassified_d__Unclassified	p__unclassified_d__Unclassified	c__unclassified_d__Unclassified	o__unclassified_d__Unclassified	f__unclassified_d__Unclassified	g__unclassified_d__Unclassified	s__unclassified_d__Unclassified	OTU2738	0	0	0	0	0	0	0	0	0	23	0	0	0	0	0	0	0	0	23

Table S3-Statistical analyses of bacterial community

Group vs. Group	Adonis		ANOSIM	
	R^2	***P*- value**	**Statistic**	***P*-value**
BX_14FSRH vs BX_14FBRH	0.4734	0.1	0.7407	0.112
BX_14FSRH vs BX_14FWRH	0.6462	0.1	1	0.103
BX_14FBRH vs BX_14FWRH	0.6927	0.1	1	0.088
BD_14FSRH vs BD_14FBRH	0.4422	0.1	0.7778	0.093
BD_14FSRH vs BD_14FWRH	0.7995	0.1	1	0.109
BD_14FBRH vs BD_14FWRH	0.7165	0.1	1	0.101

Table S4 Relative abundance of COG function classification

	BX_14FWR	BX_14FWR	BX_14FWR	BX_14FBRH	BX_14FBRH	BX_14FBRH	BX_14FSR	BX_14FSRH	BX_14FSRH	BD_14FBRI	BD_14FBRI	BD_14FBRI	BD_14FSRH	BD_14FSRI	BD_14FSRI	BD_14FWF	BD_14FWR	BD_14FWF	Description
COG1348	906	1227	1810	2875	1287	1470	2322	1486	2098	2235	1897	2679	2120	3235	1631	1197	931	1051	"The key enzymatic reactions in nitrogen fixation are catalyzed by the nitrogenase complex, which has 2 components the iron protein and the molybdenum-iron protein (By similarity)"
COG5420	132	201	95	133	139	117	152	129	116	234	149	231	119	137	138	251	142	162	nitrogen fixation
COG5554	349	652	1614	163	133	114	226	170	147	273	270	248	121	168	141	471	350	446	nitrogen fixation protein
COG5456	364	599	436	346	379	315	497	420	434	643	498	585	284	339	430	480	316	382	nitrogen fixation protein FixH
COG4656	712	878	1551	1236	1110	1277	1084	1077	1076	1348	1347	1368	1359	1349	1257	741	669	787	Required for nitrogen fixation. May be part of a membrane complex functioning as an intermediate in the electron transport to nitrogenase (By similarity)
COG3337	158	107	39	303	363	387	231	251	293	230	261	313	364	361	399	112	122	93	-
COG4338	261	285	88	635	377	425	1141	558	600	1026	675	1028	402	596	584	534	222	262	-
COG5166	0	0	0	0	0	0	0	0	0	0	0	0	0	0	0	0	0	0	-
COG5415	0	0	0	0	0	0	0	0	0	0	0	0	0	0	0	0	0	0	-
COG5487	502	1049	2806	126	209	152	142	154	183	386	191	322	165	245	235	2011	1647	1689	-
COG3280	5207	5853	4898	7778	9283	8681	7871	9098	8704	6584	7405	8189	9053	7758	9581	5541	5824	5152	"(1,4)-alpha-D-glucan 1-alpha-D-glucosylmutase activity"
COG4084	0	0	0	0	0	0	0	0	0	0	0	0	0	0	0	0	0	0	"(NiFe)-hydrogenase-3-type complex Eha, EhaM"
COG4037	0	0	0	0	0	0	0	0	0	0	0	0	0	0	0	0	0	0	"(NiFe)-hydrogenase-3-type complex Eha, membrane protein EhaF"
COG0561	17996	18504	17904	12772	12707	12679	12817	12470	13050	12310	11040	13441	13083	12822	13694	13901	14302	16000	",hydrolase"
COG1575	3556	3815	2021	7667	6365	6925	3955	6024	5331	4418	7171	4213	7085	7225	6003	2534	2659	3168	"1,4-dihydroxy-2-naphthoate octaprenyltransferase"
COG2871	383	450	119	662	812	791	667	693	690	894	793	986	894	769	832	690	366	324	"2 iron, 2 sulfur cluster binding"
COG1021	2101	3119	4071	370	451	470	271	246	317	296	292	327	459	467	458	1712	1466	2402	"2,3-dihydroxybenzoate-AMP ligase"
COG3484	798	1009	1663	1819	890	996	2087	1148	1505	1970	1756	2076	1334	1761	1232	1070	884	938	"20S proteasome, A and B subunits"
COG0567	7476	7599	5839	9030	11020	10500	10266	11419	10634	9297	9763	10697	10052	8741	11403	7323	6975	6515	"2-oxoglutarate dehydrogenase, E1"
COG1218	1582	2246	3519	3249	1657	1690	3346	2035	2773	2912	2132	3578	2527	3461	1921	3143	2590	2485	"3'(2'),5'bisphosphate nucleotidase"
COG1145	22	64	20	28	10	21	70	38	37	137	98	94	12	28	31	94	38	32	"4Fe-4S ferredoxin, iron-sulfur binding"
COG1719	644	421	188	11852	9344	10276	6279	9926	7935	5690	11848	5660	10261	10368	8457	606	528	358	"4-vinyl reductase, 4VR"
COG2503	848	1325	3156	509	394	422	1184	717	788	828	762	1022	419	543	530	2182	1986	1818	"5'-nucleotidase, lipoprotein e(p4) family"
COG3443	250	432	1414	7	3	3	16	9	12	31	15	24	4	4	3	273	246	314	"ABC superfamily ATP binding cassette transporter, binding protein"
COG1101	1273	651	282	183	120	115	1313	532	875	818	698	751	159	273	188	495	694	615	"ABC transporter, (ATP-binding protein)"
COG1126	22749	30540	54992	6231	5548	5629	12765	7934	11977	10502	9800	11008	5466	6199	5901	30449	27083	30481	"ABC transporter, (ATP-binding protein)"
COG4598	1616	3257	8680	169	109	110	1145	566	639	861	716	592	108	144	148	5690	5476	4911	"ABC transporter, (ATP-binding protein)"
COG4136	20	21	3	6	5	4	13	9	8	22	6	14	4	6	9	22	16	12	"ABC transporter, ATP-binding protein"
COG0725	9516	11358	12685	16405	16267	16296	14913	17017	16112	12929	17020	14707	16565	15619	16278	9637	8742	9431	"ABC transporter, periplasmic molybdate-binding protein"
COG4647	39	49	26	25	12	23	50	29	39	71	43	56	11	19	26	39	37	30	"acetone carboxylase, gamma subunit"
COG0511	9136	9006	9638	16849	19897	18958	17579	20039	18579	15678	16774	18322	18644	16292	20669	10365	9944	8033	"Acetyl-CoA carboxylase, biotin carboxyl carrier protein"
COG3818	351	269	135	367	248	218	588	457	640	504	368	698	192	257	300	309	177	194	"Acetyltransferase, GNAT Family"
COG3027	4837	4612	5222	7377	8948	8345	8643	9641	8664	7694	8129	8632	8084	6749	9176	6652	6158	5207	"Activator of cell division through the inhibition of FtsZ GTPase activity, therefore promoting FtsZ assembly into bundles of protofilaments necessary for the formation of the division Z ring. It is recruited early at mid-cell but it is not essential for cell division (By similarity)"
COG5581	2286	1858	3126	976	700	819	2223	1379	1550	1945	1935	1717	701	928	806	2956	2749	2646	"Acts as a flagellar brake, regulating swimming and swarming in a bis-(3'-5') cyclic diguanylic acid (c-di-GMP)- dependent manner. Increasing levels of c-di-GMP lead to decreased motility (By similarity)"
COG0465	11575	10230	6794	16796	15588	15619	17944	16070	16568	15680	15035	18435	16721	17198	16579	10154	10626	9226	"Acts as a processive, ATP-dependent zinc metallopeptidase for both cytoplasmic and membrane proteins. Plays a role in the quality control of integral membrane proteins (By similarity)"
COG2026	2256	2215	2218	8804	6617	7312	6245	7323	6513	5254	8440	5946	8073	8678	6258	3145	3698	2505	"Addiction module toxin, RelE StbE family"
COG3041	567	711	1516	1327	599	758	1028	746	1037	839	774	1054	1012	1432	728	546	540	595	"Addiction module toxin, RelE StbE family"
COG4115	1119	1540	3024	1825	808	984	2672	1345	1532	1550	1495	2166	1261	1955	947	939	1108	946	"Addiction module toxin, Txe YoeB family"
COG5580	28	29	5	41	12	6	537	149	116	187	216	257	23	53	27	29	36	20	"AHA1, activator of heat shock 90kDa protein ATPase homolog"
COG1529	19021	19394	16077	80867	81440	83852	50908	81319	67292	50929	85638	52047	80433	73280	76579	24010	21020	18010	"Aldehyde oxidase and xanthine dehydrogenase, molybdopterin binding"
COG2049	4250	5471	7434	1288	1289	1362	2693	1891	2106	2325	2093	2459	1229	1627	1457	6180	5276	5993	"Allophanate hydrolase, subunit 1"
COG0366	39947	43489	26045	46862	55640	51745	45855	53773	50138	41853	43727	48531	52758	45799	56825	34157	33838	34405	"alpha amylase, catalytic"
COG2945	5737	6188	3947	7828	9626	8941	8770	10129	9304	7801	8608	9293	8807	7271	9741	6180	5440	5255	"alpha, beta"
COG3537	8314	7441	2771	19063	24762	22554	22366	25250	21951	17459	19796	21742	22287	18107	25112	7002	8184	4288	"Alpha-1,2-mannosidase"
[illegible]	[illegible]	[illegible]	[illegible]	[illegible]	[illegible]	[illegible]	[illegible]	[illegible]	[illegible]	[illegible]	[illegible]	[illegible]	[illegible]	[illegible]	[illegible]	[illegible]	[illegible]	[illegible]	[illegible]

COG1798	123	73	20	5112	4486	4890	1841	4544	3356	2200	5446	1879	4697	4422	3922	93	77	37	"S-adenosyl-L-methionine-dependent methyltransferase that catalyzes the trimethylation of the amino group of the modified target histidine residue in translation el[illegible]
COG4583	2479	3606	3068	453	444	356	1453	823	1139	1107	1070	1045	361	386	414	3537	2836	3346	"Sarcosine oxidase, gamma subunit"
COG5028	183	185	177	113	77	67	225	186	276	129	124	254	62	88	86	75	57	43	"SEC24 family, member"
COG5613	251	98	19	68	71	82	359	223	583	67	151	293	71	70	120	31	28	36	"secretion system, effector"
COG2930	7079	5475	1567	18435	23671	21814	19328	23745	20524	15464	17835	19122	21317	16779	23931	5456	5782	3344	"SH3 domain containing, Ysc84-like 1 (S. cerevisiae)"
COG3026	752	1148	2826	305	142	161	1207	575	917	868	997	834	169	252	227	2047	1645	1711	"sigma E regulatory protein, MucB RseB"
COG0616	8229	7901	8195	13471	13956	13615	14011	14208	13603	13213	12638	15232	13788	13109	14633	9729	9432	8171	"Signal peptide peptidase, SppA"
COG1676	248	148	42	10226	8973	9781	3688	9090	6717	4403	10898	3764	9394	8843	7844	186	155	74	"sites to release the intron. The products are an intron and two tRNA half-molecules bearing 2',3' cyclic phosphate and 5'-OH termini. Recognizes a pseudosymmetric s[illegible]
COG4973	2180	2293	3466	1921	1253	1419	3865	2445	2938	2986	3135	3516	1376	1916	1671	3145	2448	2551	"Site-specific tyrosine recombinase, which acts by catalyzing the cutting and rejoining of the recombining DNA molecules. The XerC-XerD complex is essential to conve[illegible]
COG4974	65578	70228	44551	97236	110405	109637	99174	110707	102810	88830	95844	103475	106540	97423	114815	59195	58231	55394	"Site-specific tyrosine recombinase, which acts by catalyzing the cutting and rejoining of the recombining DNA molecules. The XerC-XerD complex is essential to conve[illegible]
COG1412	123	73	20	5112	4486	4890	1841	4544	3356	2200	5446	1879	4697	4422	3922	93	77	37	"small subunit (SSU) processome component, homolog"
COG4146	1578	1257	559	1251	1423	1284	3371	1871	1793	2784	2205	2868	1335	1976	1646	1253	1629	800	"solute carrier family 5 (sodium glucose cotransporter), member"
COG4509	7	2	5	7	6	5	4	4	3	6	7	9	7	5	3	11	6	14	"sortase, SrtB family"
COG2103	6040	5210	2743	12212	15452	13549	12744	14776	13137	10974	11623	13076	14273	11596	15373	4875	5475	3580	"Specifically catalyzes the cleavage of the D-lactyl ether substituent of MurNAc 6-phosphate, producing GlcNAc 6- phosphate and D-lactate"
COG2520	299	197	67	10321	9098	9913	3748	9180	6791	4509	10980	3860	9520	8975	7990	257	196	130	"Specifically methylates the N1 position of guanosine-37 in various cytoplasmic and mitochondrial tRNAs. Methylation is not dependent on the nature of the nucleosid[illegible]
COG1873	1897	802	290	5276	4641	5054	2227	4781	3644	3042	5708	2131	4830	4571	4075	769	1029	857	"sporulation protein, YlmC YmxH family"
COG1528	1995	3301	3190	423	243	238	1286	477	514	797	509	1005	255	464	260	1644	2030	2236	"Stores iron in a soluble, non-toxic, readily available form. Important for iron homeostasis (By similarity)"
COG3729	232	388	1400	21	28	23	59	36	38	100	40	60	16	28	39	244	233	329	"Stress-induced protein, KGG, repeat protein"
COG1815	5118	4405	3970	7930	9601	9095	8503	9916	8953	7981	8424	8826	8983	7496	9874	5418	5112	4309	"Structural component of flagellum, the bacterial motility apparatus. Part of the rod structure of flagellar basal body (By similarity)"
COG0258	6462	6061	3735	13477	14813	14572	10134	14395	12125	9864	13281	10829	14178	12399	14350	5004	4951	4904	"Structure-specific nuclease with 5'-flap endonuclease and 5'-3' exonuclease activities involved in DNA replication and repair. During DNA replication, cleaves the 5'-ov[illegible]
COG2009	6951	6772	5363	12735	13763	13522	10646	14568	12721	9897	13792	10886	13047	11381	13385	6510	6012	5846	"succinate dehydrogenase, cytochrome"
COG0822	8627	8371	5826	18614	19526	20005	15250	19276	17264	14089	18147	16025	19134	17487	19557	8303	7937	7500	"SUF system FeS assembly protein, NifU family"
COG4150	249	434	1415	6	2	3	21	11	13	36	33	34	4	6	1	272	245	308	"Sulfate ABC transporter, periplasmic sulfate-binding protein"
COG4032	403	229	89	233	245	218	724	467	790	323	416	553	252	323	291	247	144	140	"Sulfopyruvate decarboxylase, subunit alpha"
COG5259	0	0	0	0	0	0	0	0	0	0	0	0	0	0	0	0	0	0	"SWI SNF related, matrix associated, actin dependent regulator of chromatin, subfamily c, member"
COG5531	719	537	200	813	817	963	1360	1058	1371	1309	1357	1425	817	900	1125	702	559	507	"SWI SNF related, matrix associated, actin dependent regulator of chromatin, subfamily d, member"
COG4651	1492	1914	2147	2414	2315	2510	2824	2365	2423	2749	2748	2810	2301	2415	2630	1526	1342	1237	"system, protein"
COG5162	0	0	0	0	0	0	0	0	0	0	0	0	0	0	0	0	0	0	"TAF10 RNA polymerase II, TATA box binding protein (TBP)-associated factor"
COG5624	0	0	0	0	0	0	0	0	0	0	0	0	0	0	0	0	0	0	"TAF12 RNA polymerase II, TATA box binding protein (TBP)-associated factor"
COG4521	616	1083	2920	109	89	90	383	162	131	465	381	249	81	88	68	2005	1732	1719	"Taurine ABC transporter, periplasmic"
COG4626	776	1110	2953	323	388	388	386	419	437	549	475	577	326	371	473	883	691	900	"Terminase, large subunit"
COG5484	306	479	1473	18	12	9	49	30	23	96	58	59	11	12	12	332	388	499	"terminase, small subunit"
COG0201	7914	8091	6094	15946	16452	16513	13427	16757	15094	12622	16111	14121	16249	15055	16445	7752	7407	6822	"The central subunit of the protein translocation channel SecYEG. Consists of two halves formed by TMs 1-5 and 6-10. These two domains form a lateral gate at the fro[illegible]
COG5240	18	24	4	27	35	32	22	25	36	42	39	45	35	40	38	26	12	101	"The coatomer is a cytosolic protein complex that binds to dilysine motifs and reversibly associates with Golgi non- clathrin-coated vesicles, which further mediate bio[illegible]
COG5096	8	4	3	16	16	12	10	8	8	14	6	18	10	18	14	8	7	14	"The coatomer is a cytosolic protein complex that binds to dilysine motifs and reversibly associates with Golgi non- clathrin-coated vesicles, which further mediate bio[illegible]
COG0213	5888	5704	3770	7273	9217	8702	7021	8935	7898	7002	7321	7666	8419	7201	9610	4422	4317	4385	"The enzymes which catalyze the reversible phosphorolysis of pyrimidine nucleosides are involved in the degradation of these compounds and in their utilization as ca[illegible]
COG2041	17008	17433	9931	31699	35581	34812	30065	35424	32013	26583	31709	30500	34078	30269	36128	15229	14221	13647	"The exact function is not known. Can catalyze the reduction of a variety of substrates like dimethyl sulfoxide, trimethylamine N-oxide, phenylmethyl sulfoxide and L-m[illegible]
COG0091	7839	7957	5939	15935	16437	16502	13385	16732	15066	12607	16090	14095	16241	15038	16433	7701	7358	6788	"The globular domain of the protein is located near the polypeptide exit tunnel on the outside of the subunit, while an extended beta-hairpin is found that lines the wa[illegible]
COG3857	3804	2602	956	7028	9017	8454	7050	8994	7967	6420	6854	7657	8323	6876	9233	2603	3076	2192	"The heterodimer acts as both an ATP-dependent DNA helicase and an ATP-dependent, dual-direction single-stranded exonuclease. Recognizes the chi site generating [illegible]
COG1074	5796	5326	4758	9727	11556	11210	10313	11754	10786	9818	10086	11092	10754	9288	12049	6250	6127	4947	"The heterodimer acts as both an ATP-dependent DNA helicase and an ATP-dependent, dual-direction single-stranded exonuclease. Recognizes the chi site generating [illegible]
COG4447	14420	13449	6831	32256	40030	38397	30127	38352	33063	27200	30214	32144	36998	30801	40699	14884	14552	11012	"The ortholog in A.thaliana is involved in photosystem II (PSII) assembly, but knockout of the corresponding gene in Synechoccus PCC 7002 has no effect on PSII activit[illegible]
COG0638	3911	5081	2500	26641	23669	25782	10134	23642	17870	11771	28100	10477	24600	23133	20886	3204	2790	3909	"The proteasome is a multicatalytic proteinase complex which is characterized by its ability to cleave peptides with Arg, Phe, Tyr, Leu, and Glu adjacent to the leaving g[illegible]
COG0005	10032	10068	6428	17394	20683	19774	16294	19667	17657	15243	16012	17933	19537	16665	21309	10981	10530	9154	"The purine nucleoside phosphorylases catalyze the phosphorolytic breakdown of the N-glycosidic bond in the beta- (deoxy)ribonucleoside molecules, with the format[illegible]
COG2255	7766	7899	5925	10838	11955	11628	11556	12206	11722	10427	10663	12227	11555	10631	12524	7634	7308	6770	"The RuvA-RuvB complex in the presence of ATP renatures cruciform structure in supercoiled DNA with palindromic sequence, indicating that it may promote strand e[illegible]
COG0632	7783	8016	6071	10825	11949	11612	11579	12201	11718	10418	10652	12229	11544	10615	12511	7652	7323	6775	"The RuvA-RuvB complex in the presence of ATP renatures cruciform structure in supercoiled DNA with palindromic sequence, indicating that it may promote strand e[illegible]
COG0178	17819	18681	12140	26455	31865	30515	27601	31357	28932	24388	26241	28928	29691	25558	32867	17359	16948	15407	"The UvrABC repair system catalyzes the recognition and processing of DNA lesions. UvrA is an ATPase and a DNA-binding protein. A damage recognition complex com[illegible]
COG2143	555	748	167	938	934	1040	2810	1516	1454	1607	1722	1983	921	963	1220	863	825	428	"thioredoxin, soxw"
COG0756	6331	7086	5416	8470	10259	9641	9560	10657	9871	8559	9165	10069	9477	8253	10555	6852	6153	5981	"This enzyme is involved in nucleotide metabolism it produces dUMP, the immediate precursor of thymidine nucleotides and it decreases the intracellular concentrati[illegible]
COG0094	7881	7966	5940	15938	16440	16503	13390	16734	15068	12611	16098	14104	16242	15046	16434	7716	7374	6808	"This is 1 of the proteins that binds and probably mediates the attachment of the 5S RNA into the large ribosomal subunit, where it forms part of the central protubera[illegible]
COG0256	7878	7965	5939	15936	16438	16502	13388	16732	15068	12607	16096	14096	16242	15040	16434	7713	7372	6799	"This is one of the proteins that binds and probably mediates the attachment of the 5S RNA into the large ribosomal subunit, where it forms part of the central protub[illegible]
COG0097	7877	7964	5939	15936	16437	16502	13388	16732	15068	12606	16096	14095	16242	15040	16434	7713	7371	6799	"This protein binds to the 23S rRNA, and is important in its secondary structure. It is located near the subunit interface in the base of the L7 L12 stalk, and near the tRN[illegible]
COG0323	9533	9301	6122	16671	19664	18652	17020	19682	17956	15372	16422	17951	18429	15904	20237	9291	9035	7667	"This protein is involved in the repair of mismatches in DNA. It is required for dam-dependent methyl-directed DNA mismatch repair. May act as a molecular matchma[illegible]
COG0102	7924	8016	5942	16125	16611	16710	13552	16921	15220	12745	16271	14275	16408	15212	16607	7779	7437	6835	"This protein is one of the early assembly proteins of the 50S ribosomal subunit, although it is not seen to bind rRNA by itself. It is important during the early stages of [illegible]
COG0776	19994	19920	17688	33475	38944	36674	37990	41043	38179	33239	35428	39061	36078	31481	40575	22500	20802	17902	"This protein is one of the two subunits of integration host factor, a specific DNA-binding protein that functions in genetic recombination as well as in transcriptional a[illegible]
COG3060	299	404	1404	68	23	16	145	124	192	45	55	156	13	21	14	235	209	296	"This regulatory protein, when combined with SAM (S- adenosylmethionine) represses the expression of the methionine regulon and of enzymes involved in SAM synt[illegible]
COG0315	7011	7720	7054	15196	15723	15796	12324	15814	14190	11543	15288	12808	15654	14147	15681	8519	8531	7589	"Together with MoaA, is involved in the conversion of 5'- GTP to cyclic pyranopterin monophosphate (cPMP or molybdopterin precursor Z) (By similarity)"
COG0063	8693	10048	7097	16083	16580	16678	13595	16747	15056	12352	16256	14016	16373	15186	16553	8756	7967	8049	"Together with NAD(P)HX epimerase, which catalyzes the epimerization of the S- and R-forms, the enzyme allows the repair of both epimers of NAD(P)HX, a damaged [illegible]
COG3943	852	670	223	1888	1872	2071	2245	1851	1890	2057	2054	2296	1847	1963	2114	704	559	508	"Toxin-antitoxin system, toxin component, Fic family"
COG4679	3396	2676	788	9416	10774	10565	8888	10949	9434	7217	8528	9111	10444	9157	10867	2859	3033	1848	"Toxin-antitoxin system, toxin component, RelE family"
COG4680	819	1593	2865	1181	518	512	1817	755	1023	1319	1249	1462	1145	1548	578	2315	2779	1871	"Toxin-antitoxin system, toxin component, RelE family"
COG1675	145	92	22	5157	4572	4935	1902	4607	3450	2319	5515	2032	4765	4569	4026	135	125	77	"Transcription factor that plays a role in the activation of archaeal genes transcribed by RNA polymerase. Facilitates transcription initiation by enhancing TATA-box rec[illegible]
COG1733	25532	25971	23622	38911	43729	41847	39165	43242	39168	34575	41636	39055	41273	37489	42809	24780	25325	21099	"transcription, DNA-dependent"
COG0819	3495	4245	4516	2158	1865	1873	1884	1340	1769	1823	1540	1923	2133	2441	1731	3903	3557	4225	"Transcriptional activator, tena"
COG2336	2540	2043	701	6478	8148	7620	7002	8448	7167	5406	6317	6855	7309	5966	8289	1951	2060	1244	"transcriptional regulator antitoxin, MazE"
COG4977	30370	34432	45405	38069	45517	42605	52574	48775	46935	42991	44662	49116	41903	38396	47621	46067	42091	36736	"Transcriptional regulator, ARAC family"
COG0640	47832	48511	32348	65736	75455	72755	65671	72704	69251	59275	64411	68347	71560	64491	76787	42719	42740	39797	"Transcriptional regulator, arsr family"
COG4860	0	0	0	1	0	2	0	0	0	0	0	0	0	1	2	0	0	0	"Transcriptional regulator, arsR family"
COG1959	14829	14405	12891	19258	19846	20430	21904	21012	21103	20066	19803	22792	19598	19938	21485	13215	13670	12178	"Transcriptional regulator, BadM Rrf2 family"
COG3905	2552	2637	2062	6261	8008	7298	6181	7730	6540	5316	5862	6304	7317	5630	7954	3545	3359	2546	"transcriptional regulator, copG family"
COG0664	43576	46116	36315	94721	103625	97690	107059	107833	98419	89181	96944	104867	99975	92245	106649	51354	52578	36154	"transcriptional regulator, crp fnr family"
COG4463	909	325	72	24	24	28	33	20	35	353	33	41	18	21	23	324	559	551	"transcriptional regulator, ctsr"
COG1167	23588	26304	36741	17743	19983	19942	28198	22929	25284	22423	23988	24208	18594	17428	20939	31502	29581	28090	"Transcriptional regulator, gntR family"
COG1725	10128	7739	4499	9238	10573	9996	9346	10132	9676	8913	8348	9729	9862	8860	10647	6471	7360	6868	"Transcriptional regulator, GntR family"
COG2771	6361	10183	10575	2356	2011	2063	5287	3345	4185	4577	4140	4433	1991	2248	2377	8743	6868	8443	"Transcriptional regulator, luxr family"
COG2005	1965	2295	3314	6681	5972	6399	5488	6803	6233	4890	8265	4854	6233	5757	5593	3313	2713	2553	"Transcriptional regulator, ModE family"
COG3682	11733	11212	4472	26240	32083	31024	25119	30870	27161	21960	24223	26502	29859	25390	32832	10119	10702	7639	"transcriptional repressor, copy family"
COG3504	3771	3096	1162	7036	8867	8056	8542	9637	9233	7201	7998	8570	7846	6327	8992	3111	2801	2002	"transfer protein, trbG"
COG5386	152	132	44	5	8	6	5	3	6	9	11	11	3	4	5	118	199	258	"Transfers its hemin to hemin-free IsdC (apo-IsdC) directly probably through the activation of the holo-IsdA-apo-IsdC complex and driven by the higher affinity of apo-I[illegible]
COG1663	4443	4574	4236	8835	10745	10216	9728	11020	9989	8665	9388	10192	9894	8474	11116	5530	5153	3961	"Transfers the gamma-phosphate of ATP to the 4'-position of a tetraacyldisaccharide 1-phosphate intermediate (termed DS-1- P) to form tetraacyldisaccharide 1,4'-bis[illegible]
COG4370	92	72	36	934	102	169	865	201	566	425	293	739	542	1027	156	54	113	64	"Transfers the gamma-phosphate of ATP to the 4'-position of a tetraacyldisaccharide 1-phosphate intermediate (termed DS-1- P) to form tetraacyldisaccharide 1,4'-bis[illegible]
COG3672	1044	1769	2153	622	634	526	876	679	671	1334	943	1161	507	636	709	2768	2714	1969	"Transglutaminase family protein cysteine peptidase, btlcp"
[illegible]																			

COG2110	4136	3225	1061	15805	15568	15678	12072	15471	13862	10613	14581	12758	15854	15101	15436	3333	3316	2619	appr-1-p processing domain protein
COG0794	6862	7309	5471	14105	15394	15250	11933	15721	13591	11655	15074	12298	14967	12968	15264	7216	6768	6230	Arabinose 5-phosphate isomerase
COG1672	723	701	1492	5727	5330	5690	2532	5246	4141	2873	6155	2786	5317	5162	4761	574	529	544	Archaeal ATPase
COG1602	123	73	20	5112	4486	4890	1841	4544	3356	2200	5447	1884	4697	4422	3922	93	77	37	Archaeal protein of unknown function (DUF650)
COG3138	1524	2538	5780	543	398	425	1583	889	891	1508	1178	981	423	464	469	5560	5423	4754	arginine
COG2235	559	853	1502	447	514	500	912	499	584	774	641	817	484	541	524	1837	1591	1493	Arginine dihydrolase
COG4161	203	370	1395	0	1	0	2	3	1	9	4	3	0	5	0	223	205	292	arginine-importing ATPase activity
COG0165	9109	8723	7417	15914	16440	16529	13486	16670	14983	12865	16043	14031	16253	15076	16442	8245	8772	7546	argininosuccinate lyase
COG0018	8943	8386	6058	16758	16660	16748	13774	16932	15648	13212	16403	14580	16838	16036	16645	8106	8045	7411	arginyL-tRNA synthetase
COG5369	0	0	0	0	0	0	0	0	0	0	0	0	0	0	0	0	0	0	Armadillo repeat containing 8
COG1393	6744	6354	9029	4594	3331	3589	8029	5208	6700	7320	6216	7300	3653	4648	4009	7920	7533	7040	arsenate reductase (glutaredoxin) activity
COG1055	6926	5471	4336	15391	16107	16219	12514	16150	14182	12345	15635	13068	16044	14722	16124	6639	6539	5554	arsenical pump membrane protein
COG0798	4021	3772	2194	9043	10176	9566	10011	10378	9997	8295	8980	9910	9918	8849	10415	3495	3930	2389	arsenical-resistance protein
COG1777	1715	2395	1235	555	564	623	601	453	514	687	631	529	619	713	699	1450	1208	2007	arsR family transcriptional regulator
COG4321	1086	1671	1992	796	788	738	1393	1064	1302	1567	1173	1556	647	723	873	2609	1974	1941	arylsulfate sulfotransferase-like protein
COG2411	564	520	1425	5178	4557	4960	2153	4721	3865	2252	5573	2120	4752	4460	3965	329	299	348	ASCH
COG4405	1955	844	309	339	331	333	474	276	290	1138	484	418	376	389	379	858	1067	963	asch domain protein
COG5137	3	1	0	2	2	1	2	2	0	4	2	9	0	6	0	3	2	10	ASF1 anti-silencing function 1 homolog
COG2982	7858	8301	8795	14220	17856	16485	15449	18552	17226	13789	15231	16427	16171	13448	18165	10795	10421	7964	asmA family
COG3473	2571	3245	2855	5777	5189	5568	3331	5470	4903	3120	6441	3086	5354	5072	4586	1978	1527	2321	Asp Glu hydantoin racemase
COG1446	3845	3489	2801	8415	9524	8882	9148	9741	9498	7492	8036	9306	9143	8278	9845	3222	3425	2227	asparaginase
COG0367	11182	10439	8392	26790	26499	27413	20574	26562	23774	19655	26085	21957	26860	25360	26199	10586	10407	9223	asparagine synthetase
COG2502	438	345	96	1781	1950	2338	1178	1859	1515	962	1316	1292	1900	1732	1989	375	408	251	asparagine synthetase a
COG0017	4542	3837	3020	22823	22041	22628	13122	22201	17917	13318	23135	13559	22339	20692	20603	3353	3709	2531	asparaginyl-tRNA synthetase
COG1027	4939	5733	5098	7845	9206	8724	7059	8845	7937	6391	7114	7658	8871	7465	9348	5511	5220	5065	Aspartate ammonia-lyase
COG1608	136	82	24	5143	4530	4936	1858	4564	3380	2228	5462	1903	4728	4454	3963	106	92	54	Aspartate glutamate uridylate kinase
COG2054	77	94	27	162	104	136	250	160	143	340	265	252	113	158	163	141	79	91	Aspartate glutamate uridylate kinase
COG1794	3197	2736	3515	3026	3011	3500	3556	3326	3439	3374	3411	3315	2962	2896	3195	3665	4122	3313	aspartate racemase
COG0540	8118	8080	6000	15935	16462	16494	13801	16783	15486	12528	16170	14096	16236	15030	16406	7745	7427	6810	aspartate transcarbamylase
COG0527	11818	11466	9685	27487	29312	29006	22267	29539	25501	21264	28185	23152	28552	25802	28903	11161	11045	9532	Aspartokinase
COG3577	1052	1691	2019	2018	1054	1139	2276	1353	1736	2337	1918	2389	1434	1910	1392	2836	2231	2103	Aspartyl protease
COG0173	7443	7598	5667	10819	11951	11621	11512	12183	11700	10407	10651	12198	11545	10615	12516	7510	7205	6668	aspartyl-trna synthetase
COG3807	827	924	604	890	720	701	1338	1193	1072	1491	1354	1623	606	858	798	931	730	667	Aspartyl-tRNA synthetase
COG1706	4097	4000	3859	7646	9311	8753	8410	9802	8798	7555	8261	8697	8640	7187	9549	5032	4505	3665	Assembles around the rod to form the L-ring and probably protects the motor basal body from shearing forces [illegible]
COG2063	4087	4011	3857	7601	9263	8675	8408	9767	8784	7515	8246	8688	8571	7143	9472	5032	4487	3738	Assembles around the rod to form the L-ring and probably protects the motor basal body from shearing forces [illegible]
COG0858	7769	7903	5926	10867	12014	11675	11582	12232	11749	10473	10685	12280	11613	10700	12587	7640	7307	6785	Associates with free 30S ribosomal subunits (but not with 30S subunits that are part of 70S ribosomes or polysomes) [illegible]
COG0264	7755	7892	5919	10824	11953	11614	11547	12189	11712	10406	10650	12217	11545	10620	12516	7621	7295	6763	Associates with the EF-Tu.GDP complex and induces the exchange of GDP to GTP. It remains bound to the aminoacyl [illegible]
COG2102	2913	2427	960	16849	17472	17661	9682	17298	13685	9789	17150	10429	17052	15110	16281	2251	2683	1517	ATP- binding
COG1423	183	162	36	316	362	356	194	376	257	222	232	457	464	398	284	156	164	147	ATP dependent DNA ligase
COG2229	1564	1155	471	3471	3101	3251	2670	2293	3005	2505	2395	3317	3211	3639	3210	1252	1152	1344	atp gtp-binding protein
COG3869	1291	715	203	929	1098	1278	827	967	942	1290	891	1138	1200	1059	1330	733	996	949	ATP guanido phosphotransferase
COG3312	330	458	1483	134	108	80	636	318	290	268	361	284	91	95	119	298	300	358	atp synthase
COG5336	2575	2985	1470	634	672	659	848	752	784	1300	776	1002	536	620	718	1912	1744	2404	atp synthase
COG5387	420	566	313	482	485	447	604	556	544	778	587	740	378	433	537	549	376	403	ATP synthase mitochondrial F1 complex assembly factor
COG2811	0	0	0	0	0	0	0	0	0	0	0	0	0	0	0	0	0	0	ATP synthase subunit h
COG1373	1685	1884	764	4105	4080	4354	4715	3930	3689	4206	4005	4530	4061	4273	4542	1844	1671	1071	ATPase (AAA
COG2607	1384	1617	1888	2070	1024	1135	3106	1866	2398	2453	2360	2885	1408	2108	1203	2765	2230	2137	ATPase (AAA
COG1483	341	358	108	705	882	939	494	607	624	644	573	751	981	779	932	346	294	412	ATPase (AAA superfamily)-like protein
COG1269	1747	1393	2970	5875	5258	5692	2621	5214	4124	3220	6232	2723	5513	5153	4639	1108	1175	1310	ATPase 116 kDa subunit
COG0714	27668	29126	19179	42077	41658	43902	35996	39914	38536	36182	40985	39657	42628	41199	43365	27466	25952	26542	ATPase activity
COG1122	6571	5083	2248	25700	21015	23008	12163	20987	17886	13116	24817	12467	23184	23315	18939	3800	4282	4593	ATPase activity
COG3911	329	889	544	272	279	267	1012	548	612	667	528	748	259	373	351	549	725	243	atpase-like protein
COG0489	22063	21957	13114	43180	44865	44800	37441	44714	40475	34146	41126	39009	44524	41047	45192	20619	20227	18214	ATP-binding protein
COG3950	734	737	1516	1986	786	789	1849	1014	1742	1279	1249	1842	1526	2407	851	572	679	513	Atp-binding protein
COG4170	203	370	1395	1	1	0	2	3	1	9	4	3	0	5	0	223	205	294	atp-binding protein
COG4930	83	126	31	244	285	274	175	204	185	214	250	251	413	247	240	100	89	101	atp-dependent
COG2170	7385	9922	5201	3450	3003	3041	4399	2890	3827	3128	3264	4214	3220	3966	3031	5889	5034	7507	ATP-dependent carboxylate-amine ligase (By similarity)
COG0542	22809	22570	19406	35440	39172	38539	35792	38605	37183	32370	33472	38005	38372	35432	40870	23835	22902	20625	ATP-dependent CLP protease ATP-binding subunit
COG0514	11196	12597	9570	14774	15438	15439	15627	15561	15257	13626	14009	16185	15374	15093	16227	12103	11619	10897	atp-dependent dna helicase
COG1200	7712	7868	5924	10494	11580	11107	11226	11730	11310	9976	10261	11775	11132	10082	11938	7622	7254	6742	ATP-dependent DNA helicase recG
COG3593	4770	5447	6294	8949	10504	9936	8909	10380	9817	8188	8781	9933	10193	8916	10797	6888	6722	5388	ATP-dependent endonuclease of the OLD
COG1067	1012	1409	2950	1112	921	1111	947	1020	896	1222	1278	1273	919	991	1084	2480	2162	2120	atp-dependent protease
COG1219	8072	8157	5989	11684	12869	12679	12301	13074	12582	11320	11604	13215	12415	11649	13568	7905	7538	7173	ATP-dependent specificity component of the Clp protease. It directs the protease to specific substrates. Can perform [illegible]
COG3919	771	700	328	1222	665	588	1041	673	1166	910	763	1254	1145	1495	651	554	664	730	ATP-grasp
COG2232	125	102	31	162	106	137	254	159	157	338	272	259	113	160	165	149	98	84	ATP-grasp domain
COG1938	3231	4641	2466	5204	4610	5011	2077	4676	3639	2327	5591	2075	4781	4550	4056	2449	2030	3387	ATP-grasp superfamily
COG3199	150	122	46	176	152	190	586	325	335	322	405	350	154	169	213	136	63	73	ATP-NAD AcoX kinase
COG5571	756	1433	4199	260	308	323	239	220	249	350	229	327	278	148	378	2199	1943	2040	Autotransporter beta-domain
COG0679	8603	9664	9543	5237	4283	4739	7427	5304	6745	7062	6412	7600	4645	5379	5091	8116	7481	8616	Auxin Efflux Carrier
COG1296	5419	5129	4818	1560	1545	1541	3445	2134	2952	3016	2661	2772	1367	1488	1613	4902	4428	4977	AzlC Family
COG3382	2688	3112	2784	5637	5112	5563	2184	4914	3789	2947	5831	2329	5294	4984	4529	1853	1807	2595	B3 4 domain protein
COG3108	4066	3594	2914	6660	8534	7832	6720	8506	7119	6547	6844	7143	7629	6230	8580	3324	3305	2416	Bacterial protein of unknown function (DUF882)
COG4312	4839	4463	3100	8462	10722	10165	8306	10137	9093	7658	8110	8912	9670	7895	10982	5257	4999	3969	Bacterial protein of unknown function (DUF899)
COG4483	748	211	34	19	18	20	24	16	27	328	19	21	13	13	16	203	384	334	Bacterial protein of unknown function (DUF910)
COG5661	98	213	184	38	21	35	83	56	38	113	50	54	22	21	25	116	86	62	Bacterial protein of unknown function (DUF922)
COG5664	88	224	194	8	1	4	56	23	21	54	25	46	5	3	2	108	88	41	Bacterial protein of unknown function (DUF922)
COG3575	1065	554	201	183	219	188	218	200	211	620	204	303	162	200	226	602	764	731	Bacterial protein of unknown function (DUF925)
COG3753	1289	1328	1920	974	922	847	2142	1382	1819	1471	1542	1811	782	780	1090	1249	970	969	Bacterial protein of unknown function (DUF937)
COG5403	215	346	523	204	227	209	240	239	213	328	292	332	162	181	231	390	293	173	Bacterial protein of unknown function (DUF937)
COG4472	911	326	75	30	29	33	35	24	40	357	42	48	25	28	27	332	561	553	Bacterial protein of unknown function (DUF965)
COG4652	2	1	0	2	1	1	1	0	4	6	1	6	1	1	2	7	1	2	bacteriocin-associated integral membrane protein
COG2906	1208	1083	1732	798	658	667	1989	1296	1810	1306	1380	1699	578	628	726	1011	771	854	Bacterioferritin-associated ferredoxin
COG3413	248	148	42	10227	8974	9783	3688	9090	6717	4404	10896	3764	9396	8845	7842	186	155	74	Bacterioopsin activator HTH domain protein
COG3566	48	61	24	24	17	14	244	112	156	62	123	75	21	19	17	37	29	19	bacteriophage protein
COG4834	19	41	20	8	10	10	18	17	13	32	14	28	13	12	10	26	27	17	Bacteriophage protein
COG2971	6708	6758	4388	8513	10856	10139	10005	10521	9484	8301	8877	9654	9947	8447	10915	5394	5783	4926	BadF BadG BcrA BcrD
COG0330	18247	17977	17232	33277	32616	32810	33625	34936	34363	29721	33343	34579	33089	32181	33749	21830	20768	18190	Band 7 protein
COG2268	3039	2468	1258	5524	5085	5838	4991	4830	4870	4503	4420	5341	5344	5805	5612	2313	2529	2002	Band 7 protein
COG1261	2057	2333	3375	1872	1598	1744	3123	2435	2675	2880	2850	3122	1723	1929	1898	3374	2711	2658	basal body p-ring
COG3948	783	1430	4276	101	162	96	328	110	116	447	257	283	68	69	107	2168	1821	1974	Baseplate assembly protein
COG4384	194	566	1380	56	17	25	77	48	32	199	169	71	23	48	23	1585	1320	1287	baseplate assembly protein
COG4540	577	1066	2893	137	202	140	342	141	134	471	276	319	100	97	147	1951	1617	1694	Baseplate Assembly protein
COG3299	671	861	1471	266	240	271	758	506	808	504	513	632	260	292	319	1786	1570	1592	baseplate J family protein
COG1744	5961	6261	4540	13122	11646	12691	7597	11764	9461	9099	14711	7651	12069	11728	10848	6052	5374	5893	basic membrane
COG2992	32	41	22	70	62	77	101	110	73	102	74	119	72	69	80	44	42	33	BAX protein
COG5374	0	0	0	0	0	0	0	0	0	0	0	0	0	0	0	0	0	0	b-cell receptor-associated protein
COG3135	2943	4440	5814	353	296	261	1408	717	1135	1171	1098	1140	268	347	355	5206	5032	5057	benzoate transporter activity
COG4880	997	406	150	364	400	413	364	263	291	720	408	353	468	479	454	457	635	738	beta propeller domain protein
COG5029	54	28	14	66	63	93	37	62	57	61	61	60	66	58	70	32	34	31	beta SUBUNIT
COG1874	14246	13545	6969	22734	29115	27487	23901	28302	24839	20494	22882	24236	26542	22760	30035	11029	11378	9527	beta-galactosidase
COG2723	13411	16367	17378	11134	13519	13224	11732	12781	11866	10946	11062	12444	12389	11539	14195	10929	9995	12462	beta-glucosidase
COG3555	1401	1815	3068	840	432	432	2178	1170	1796	1531	1175	1957	386	562	555	3798	3829	3151	beta-hydroxylase
COG2335	3745	5031	2623	6611	4652	4982	10213	5819	6325	6650	6764	8267	5916	7720	5154	4185	4008	3096	Beta-Ig-H3 fasciclin
COG1680	45651	44521	28995	77483	95216	92042	76885	90094	81316	70156	74512	81850	86957	74902	97781	43839	44547	36072	Beta-lactamase
COG2367	7606	6007	2660	15085	18407	17046	15430	17679	15830	13114	14153	15780	16870	14228	18574	5898	6328	4198	Beta-lactamase
COG2602	425	355	161	272	327	219	1015	482	449	769	817	808	255	400	316	590	496	281	Beta-lactamase
COG0491	50470	50574	36267	75061	84266	82961	72825	83407	76863	69427	76675	76490	80534	73572	86156	48056	47002	43976	Beta-lactamase domain protein
COG1237	290	254	74	871	784	941	1112	799	737	979	923	1066	791	1003	921	315	267	226	beta-lactamase domain protein
COG1782	557	414	112	6893	6440	7226	3009	6397	4856	3153	6751	3147	6595	6155	5912	461	474	269	beta-lactamase domain protein
COG2015	572	677	1536	522	548	582	751	570	512	731	608	694	445	534	573	607	474	592	Beta-lactamase domain protein
COG1907	201	167	47	5273	4590	5025	2091	4703	3499	2539	5710	2131	4809	4578	4084	234	157	128	beta-ribofuranosylaminobenzene 5-phosphate synthase
COG0376	6058	6610	5378	7692	9589	9128	7720	9581	8418	7687	7955	8580	8734	7299	10042	6417	6138	5639	Bifunctional enzyme with both catalase and broad- spectrum peroxidase activity (By similarity)
COG0138	7747	7904	5919	10833	11983	11668	11452	12040	11531	10268	10517	11975	11560	10623	12511	7621	7301	6765	bifunctional purine biosynthesis protein PURH
COG0385	7322	8224	8097	9982	11401	10986	10634	11351	10876	10015	9932	11050	10999	9921	11904	8892	8357	8091	bile acid
COG0395	68915	81163	52232	34081	35072	36058	30353	30359	33064	27771	34327	31291	34971	35507	33604	54679	49168	60996	Binding-protein-dependent transport systems inner membrane component
COG0600	31462	38141	39921	25531	25091	25160	28663	26723	29045	25244	28727	27616	24852	24894	25594	34216	29412	34063	Binding-protein-dependent transport systems inner membrane component
COG0601	40326	47829	58544	34458	34769	35750	29940	33008	35116	28481	34209	31450	34400	33495	34344	35100	31322	37417	Binding-protein-dependent transport systems inner membrane component
COG1173	38126	44944	55839	34081	34334	35344	29317	32602	34351	28166	33818	30988	33995	33110	33997	33568	30116	35464	Binding-protein-dependent transport systems inner membrane component
COG1175	62736	76145	52170	33505	34379	35366	29988	29940	32177	27612	33776	30688	34271	34853	32916	50057	44416	56367	binding-protein-dependent transport systems inner membrane Component
COG1177	11229	15801	26135	12244	11032	11957	12690	12728	12579	11300	14213	11525	11262	11572	11022	19469	17055	16945	Binding-protein-dependent transport systems inner membrane component
COG4132	790	1382	3122	114	136	141	388	197	272	312	248	252	97	130	115	1997	1785	1861	Binding-protein-dependent transport systems inner membrane component
COG4209	7637	6088	1779	568	696	691	596	485	706	806	800	704	554	729	695	5394	5703	5704	Binding-protein-dependent transport systems inner membrane component
COG4590	464	1045	2792	198	285	255	131	185	171	306	172	254	403	212	222	1884	1599	1671	Binding-protein-dependent transport systems inner membrane component
COG4662	330	331	111	778	817	947	763	754	675	812	835	914	810	805	905	399	311	261	binding-protein-dependent transport systems inner membrane Component
COG4986	2412	1902	588	10108	10930	10358	7928	11270	9157	6931	10631	7760	10327	8854	10377	1967	1967	1262	Binding-protein-dependent transport systems inner membrane component
COG0197	7877	7964	5939	15936	16437	16502	13388	16732	15068	12606	16096	14095	16242	15040	16434	7713	7371	6799	Binds 23S rRNA and is also seen to make contacts with the A and possibly P site tRNAs (By similarity)
COG0268	7658	7780	5881	10546	11672	11304	11396	12023	11508	10184	10448	11982	11312	10348	12224	7524	7171	6489	Binds directly to 16S ribosomal RNA (By similarity)
COG0292	7754	7892	5919	10824	11950	11611	11547	12189	11712	10402	10649	12215	11544	10618	12512	7620	7294	6762	Binds directly to 23S ribosomal RNA and is necessary for the in vitro assembly process of the 50S ribosomal subunit. [illegible]
COG0691	7765	7898	5923	10836	11971	11632	11556	12202	11738	10426	10666	12248	11564	10643	12550	7632	7301	6774	Binds specifically to the SsrA RNA (tmRNA) and is required for stable association of SsrA with ribosomes (By similarity)
COG0093	7877	7962	5939	15934	16438	16501	13388	16731	15068	12602	16092	14093	16241	15040	16434	7708	7371	6797	Binds to 23S rRNA. Forms part of two intersubunit bridges in the 70S ribosome (By similarity)
COG0234	8549	9049	6672	12157	13473	13142	12823	13428	12983	11868	11849	13764	13212	12182	14063	8630	8039	7490	Binds to cpn60 in the presence of Mg-ATP and suppresses the ATPase activity of the latter (By similarity)
COG0200	7877	7964	5939	15935	16437	16502	13388	16732	15068	12606	16096	14095	16242	15040	16434	7713	7371	6799	Binds to the 23S rRNA (By similarity)
COG0359	7755	7892	5919	10824	11951	11612	11547	12189	11712	10406	10649	12217	11545	10618	12512	7620	7295	6762	Binds to the 23S rRNA (By similarity)
COG2126	123	73	20	5112	4486	4890	1841	4544	3356	2200	5446	1879	4697	4422	3921	93	77	37	Binds to the 23S rRNA (By similarity)
COG1976	123	73	20	5112	4486	4890	1841	4544	3356	2200	5446	1879	4697	4422	3922	93	77	37	Binds to the 60S ribosomal subunit and prevents its association with the 40S ribosomal subunit to form the 80S [illegible]
COG0360	7755	7892	5919	10824	11951	11612	11547	12189	11712	10406	10649	12217	11545	10618	12512	7620	7295	6762	Binds together with S18 to 16S ribosomal RNA (By similarity)
COG3088	3938	3801	3823	7275	9095	8454	7565	9359	8515	6859	7563	8344	8153	6750	9223	4722	4270	3504	biogenesis protein
COG0848	18674	18703	16289	39844	44794	43756	43256	46787	43486	39601	41308	45947	43068	39301	47891	25943	24296	19539	Biopolymer transport protein exbD tolR
COG3206	15699	16232	13857	32487	37360	35385	33470	38114	34079	29023	31060	34921	35458	31348	38794	16697	15826	13128	biosynthesis protein
COG3207	4	5	2	16	11	21	14	18	15	34	28	24	24	27	39	6	5	9	biosynthesis protein
COG3754	715	986	2895	283	215	272	331	419	350	444	326	551	197	372	288	691	661	813	biosynthesis protein
COG3944	2333	1419	704	1229	1436	1509	943	1012	1125	1156	987	1177	1335	1275	1383	1259	1580	1272	biosynthesis protein
COG5653	1664	2255	2334	1794	1990	1912	1671	1821	1729	2423	1939	2430	1877	1936	1999	3202	3240	2779	biosynthesis protein CelD
COG0340	8066	8206	6001	16469	17079	17174	13763	17249	15487	13134	16548	14654	17014	15737	17063	7960	7573	7003	Biotin- acetyl-CoA-carboxylase ligase
COG4285	200	164	79	304	336	379	277	269	283	247	251	333	334	200	453	224	265	215	Biotin apoprotein ligase
COG1654	0	0	0	0	0	0	0	0	0	0	0	0	0	0	0	0	0	0	biotin-[acetyl-CoA-carboxylase] ligase activity
COG1268	6388	5433	2414	7659	8908	8313	7352	8547	8182	6512	6738	7634	8508	7308	8982	3981	4424	4320	bioY protein
COG3794	5544	3928	1345	45135	42130	44440	21904	42083	33458	23285	46448	22722	43086	40262	38452	3930	3937	2770	Blue (Type 1) copper domain protein
COG4454	1117	1428	1924	1715	831	944	1966	1086	1707	1475	1422	1976	1343	1936	936	2360	1986	1792	blue (type 1) copper domain protein
COG4692	2457	3576	2864	2404	2654	2973	2840	2745	2418	2194	2405	2762	2596	2687	2896	2223	2240	2515	BNR Asp-box repeat protein
COG5007	1193	1389	2979	770	495	549	1852	1225	1534	1386	1569	1623	513	735	628	2387	1958	1931	BolA family
COG0271	2268	2723	3750	2672	1606	1609	4173	2810	3430	3622	3166	4245	1866	2627	1888	3730	2931	2885	Bola-like protein
COG1687	78	32	28	22	24	21	27	29	40	20	46	39	19	28	18	31	25	19	Branched-chain amino acid
COG0115	17281	16975	15522	22457	22356	22820	22365	23682	23833	21215	23628	23511	22377	22117	23154	15908	15273	14755	brancheD-chain amino acid aminotransferase
COG4392	2487	1473	1698	326	217	198	1754	774	1166	1705	1318	1008	219	276	249	2626	2640	2440	Branched-chain amino acid transport
COG4541	140	303	212	41	22	31	128	73	65	176	130	139	27	40	46	220	132	71	Branched-chain amino acid transport
COG1114	1233	1242	2925	58	57	66	78	70	78	539	128	132	68	64	80	2090	2187	2352	branched-chain amino acid transport system II carrier protein
COG3426	820	298	63	248	244	301	190	185	217	628	229	317	233	286	312	318	463	502	Branched-chain carboxylic acid kinase
COG5132	0	0	0	0	0	0	0	0	0	0	0	0	0	0	0	0	0	0	BUD31 homolog
COG4023	123	73	20	5112	4486	4890	1841	4544	3356	2200	5446	1879	4697	4422	3922	93	77	37	but it may be involved in stabilization of the trimeric complex (By similarity)
COG1288	438	535	1491	85	74	59	290	130	128	298	251	202	72	93	107	440	476	591	c4-dicarboxylate anaerobic carrier
COG3069	163	91	48	81	55	58	656	343	201	214	217	321	80	75	82	94	65	55	C4-dicarboxylate transporter
COG1275	1020	1242	1660	936	1048	1069	1052	928	1076	1117	1001	1314	1201	948	1131	2308	2022	1972	C4-dicarboxylate transporter malic acid transport protein
COG1266	6180	5647	3509	13551	14168	13903	10133	13878	12076	9432	12875	10549	13934	12749	13674	4474	4505	4407	caax amino terminal protease family protein
COG4300	948	355	103	991	196	253	905	271	656	752	346	802	587	1054	207	315	572	445	cadmium resistance
COG1768	69	59	25	130	168	206	93	104	115	142	102	149	154	167	176	71	64	76	Calcineurin-like phosphoesterase
COG4875	264	638	1444	132	117	118	120	109	108	338	183	239	107	134	133	1691	1383	1354	Calcium calmodulin dependent protein kinase ii association-domain protein
COG0387	2750	2737	4104	4081	3390	3811	3922	3314	3462	3192	2984	3821	3640	3983	3549	3488	3607	2985	Calcium Proton
COG5126	340	377	275	1012	434	457	658	475	840	596	506	788	790	1175	463	288	276	234	Calcium-binding protein
COG5244	3	2	1	0	0	1	0	0	0	0	0	1	0	0	0	5	4	0	CAP-GLY domain containing linker protein
COG4464	3308	2493	640	6333	8361	7685	6203	7958	6768	5765	6152	6489	7609	6089	8448	2442	2777	1802	Capsular polysaccharide biosynthesis protein
COG4421	533	882	1466	611	365	386	1536	646	621	1176	752	1135	361	659	542	1921	1617	1603	Capsular Polysaccharide Biosynthesis Protein-Like Protein
COG3562	981	486	110	313	274	289	615	458	487	884	579	567	233	266	349	505	584	534	capsule polysaccharide
COG3563	3163	2803	1203	225	163	188	845	477	882	1034	506	599	161	180	205	1716	1799	2382	capsule polysaccharide
COG2843	3835	3697	1576	3443	2909	3089	2822	2338	2793	2994	2566	3386	3113	3848	3054	2786	2723	3362	Capsule synthesis protein
COG0549	581	890	1514	5597	5102	5481	2123	4899	3754	2682	5783	2369	5459	4982	4470	1908	1616	1564	carbamate kinase
COG0458	10815	10137	6501	21057	23354	22523	18430	23110	20621	17737	21279	19503	22288	19975	23520	9780	9674	8359	carbamoyl-phosphate synthetase ammonia chain
COG0505	8607	8170	5996	10828	11974	11614	11623	12067	11554	10696	10672	12017	11547	10629	12490	7948	7779	7143	carbamoyl-phosphate synthetase glutamine chain
COG2222	3733	3900	4193	6824	8834	8056	7079	8626	7683	6273	6966	7326	7838	6490	8891	4435	4228	3333	carbohydrate binding
COG1070	17152	18638	15697	24714	28174	27308	22713	26960	24250	20768	24618	23290	26740	24043	27989	16293	15514	15315	Carbohydrate kinase
COG1649	4311	3622	2842	10181	8419	7649	10224	8501	9334	8058	7761	10029	9737	10773	8851	3296	4114	2546	carbohydrate metabolic process
COG3833	3064	3255	2769	409	495	485	580	381	512	861	540	571	374	492	454	2004	1990	2773	carbohydrate transport
COG3659	4948	5537	9392	7960	9898	9438	8468	10219	9237	7531	8060	9081	8864	7258	10419	7102	7283	6054	Carbohydrate-selective porin OprB
COG1966	5088	5062	4779	4251	4353	5076	3771	4394	3960	4258	3820	4443	4258	4258	4688	4963	4942	5265	carbon starvation protein
COG0288	13619	13339	11629	24083	28356	26693	25086	28302	25925	22016	23289	25726	26756	23191	29341	13886	14298	11560	carbonic anhydrase
COG3338	584	434	211	1154	369	384	1131	655	904	1068	1008	1175	787	1379	448	640	571	431	Carbonic anhydrase
COG4770	16750	19520	15081	22041	22908	23589	20252	23336	22203	19890	23248	21590	22340	21940	23460	17944	15989	17514	carboxylase
COG4799	13178	13597	8320	22087	25687	24894	19263	24988	22183	19524	23059	20717	23978	21048	25603	13479	12449	11979	carboxylase
COG2272	11233	12703	7057	17210	21478	20817	16729	21014	18319	14429	16421	17074	19592	16514	21844	9099	8572	9025	Carboxylesterase
COG1647	5209	6752	5474	2514	2154	2363	3010	2000	2287	3115	2494	3053	2063	2281	2350	6642	5862	7424	carboxylesterase (ec 3.1.1.1)
COG0163	5172	5318	8069	8900	9978	9420	10135	10414	9856	8245	8888	9957	9525	8451	10250	5910	5633	4922	carboxy-lyase activity
COG1686	10790	8928	11371	6263	6432	6647	8721	6844	7689	9058	7975	8744	6056	5947	6994	10324	10035	9719	Carboxypeptidase
COG1876	3955	3399	1539	2016	1088	1251	1963	894	1408	1839	1028	1841	1528	2093	1164	2372	2485	3197	Carboxypeptidase
COG2317	2478	1429	1681	1302	1561	1635	1257	1518	1501	2196	1484	1860	1430	1647	1675	1288	1617	1684	Carboxypeptidase
COG2939	5692	4855	1584	13183	16897	15530	13911	16994	14752	11630	12994	14030	15282	11835	16962	4928	4813	3273	carboxy-peptidase
COG2324	1130	529	166	1521	801	844	1513	774	1185	1464	945	1607	1227	1676	1098	584	835	672	Carotene biosynthesis associated membrane protein
COG0236	19112	19162	15339	28163	32879	31401	28820	32763	30587	26589	27556	31260	31004	27601	33957	21133	20442	17920	Carrier of the growing fatty acid chain in fatty acid biosynthesis (By similarity)
COG3202	816	1022	2925	1424	682	762	1222	1147	1637	1327	1127	1795	1173	1626	901	781	718	971	carrier protein
COG5041	0	0	0	0	0	0	0	0	0	0	0	0	0	0	0	0	0	0	casein kinase ii
COG0753	11570	12996	13634	9511	11432	10880	11880	12173	11805	10837	10867	11973	10473	9076	11724	14581	13933	13876	Catalase
COG3546	5572	3378	1111	7715	9052	8348	7355	8582	7793	6833	6713	7555	8568	7390	8934	3230	3798	2770	Catalase
COG0274	7519	6920	4496	14133	14771	14795	10669	14617	12755	10443	13628	11166	14502	13312	14689	5095	5561	5066	Catalyzes a reversible aldol reaction between acetaldehyde and D-glyceraldehyde 3-phosphate to generate 2-deoxy- [illegible]
COG0757	5445	6502	6853	3986	3085	3339	5793	3975	4640	5051	4513	5507	3429	4429	3536	7122	6328	6597	Catalyzes a trans-dehydration via an enolate intermediate (By similarity)
COG2933	549	1035	2815	139	73	96	132	112	106	397	291	237	120	287	156	1945	1647	1696	Catalyzes the 2'-O-methylation at nucleotide C2498 in 23S rRNA (By similarity)
COG0313	8366	8480	6289	11112	11979	11601	13496	13069	12839	11572	11995	13618	11490	11089	12651	8274	7967	6965	Catalyzes the 2'-O-methylation of the ribose of cytidine 1402 (C1402) in 16S rRNA (By similarity)
COG1154	7903	7908	5675	10932	12074	11799	11410	11978	11576	10216	10473	11978	11656	10601	12650	7670	7985	6918	Catalyzes the acyloin condensation reaction between C atoms 2 and 3 of pyruvate and glyceraldehyde 3-phosphate [illegible]
COG0769	11239	11232	7814	13296	12996	12716	13950	13274	13952	12425	12389	14643	13496	13600	13628	9942	9763	9617	Catalyzes the addition of meso-diaminopimelic acid to the nucleotide precursor UDP-N-acetylmuramoyl-L-alanyl-D- [illegible]
COG1759	246	146	40	10222	8972	9776	3682	9086	6712	4400	10892	3758	9392	8840	7844	186	154	74	Catalyzes the ATP- and formate-dependent formylation of 5-aminoimidazole-4-carboxamide-1-beta-d-ribofuranosyl [illegible]
COG0504	9399	9977	10354	18402	19092	19673	15356	19411	17459	14444	18257	16376	18833	17631	19327	10358	9705	9087	Catalyzes the ATP-dependent amination of UTP to CTP with either L-glutamine or ammonia as the source of nitrogen [illegible]
COG0603	6487	5146	4197	15009	15455	15473	11986	15711	13940	11823	15010	12693	15255	13961	15373	6136	6756	5232	Catalyzes the ATP-dependent conversion of 7-carboxy-7- deazaguanine (CDG) to 7-cyano-7-deazaguanine (preQ(0)) [illegible]
COG0083	6933	6247	3987	13721	14398	14270	10137	13923	12137	9752	12983	10624	14235	12859	14134	4854	5280	4721	Catalyzes the ATP-dependent phosphorylation of L-homoserine to L-homoserine phosphate (By similarity)
COG0013	10818	10024	6633	21726	24154	23497	18726	24087	21226	17574	21554	19718	23163	20327	24058	9664	9655	8379	Catalyzes the attachment of alanine to tRNA(Ala) in a two-step reaction alanine is first activated by ATP to form Ala- [illegible]
COG0008	16644	17239	14639	26945	29117	28896	26068	30267	27650	24766	28486	27630	28019	25739	29899	18147	16794	15776	Catalyzes the attachment of glutamate to tRNA(Glu) in a two-step reaction glutamate is first activated by ATP to [illegible]
COG0423	2892	3620	1973	7174	6866	7453	3984	6463	5389	4241	7421	4312	6958	6802	6532	2418	2341	2613	Catalyzes the attachment of glycine to tRNA(Gly) (By similarity)
COG2024	0	0	0	0	0	0	0	0	0	0	0	0	0	0	0	0	0	0	Catalyzes the attachment of O-phosphoserine (Sep) to tRNA(Cys) (By similarity)
COG0162	8711	8385	6430	15955	16456	16520	13451	16766	15105	12973	16129	14167	16257	15058	16456	7994	7990	7279	Catalyzes the attachment of tyrosine to tRNA(Tyr) in a two-step reaction tyrosine is first activated by ATP to form Tyr [illegible]
COG1166	986	1376	2944	2179	1327	1618	2058	1518	1994	1976	1577	2440	1780	2430	1868	2347	2064	2124	Catalyzes the biosynthesis of agmatine from arginine (By similarity)
COG0854	2554	3059	3872	3881	2864	3093	5558	3832	4373	4684	4412	5389	3321	4103	3443	4073	3601	3112	Catalyzes the complicated ring closure reaction between the two acyclic compounds 1-deoxy-D-xylulose-5-phosphate [illegible]
COG0329	31381	38380	34298	36364	41768	40849	38068	42364	39646	32998	38091	38162	39586	34941	42620	32227	28976	31086	Catalyzes the condensation of (S)-aspartate-beta- semialdehyde (S)-ASA and pyruvate to 4-hydroxy- tetrahydrodipic [illegible]
COG0040	7768	7895	5892	15793	16307	16407	13149	16452	14763	12360	15858	13730	16137	14938	16291	7636	7310	6751	Catalyzes the condensation of ATP and 5-phosphoribose 1- diphosphate to form N'-(5'-phosphoribosyl)-ATP (PR-ATP) [illegible]
COG0379	7322	7610	5708	10600	11788	11461	10910	11698	10868	9899	10210	11439	11342	10404	12296	7249	7171	6653	Catalyzes the condensation of iminoaspartate with dihydroxyacetone phosphate to form quinolinate (By similarity)
COG0020	10359	10998	7513	18165	18948	19390	14898	18915	17011	13883	17724	15787	18655	17293	18933	9632	9017	9012	Catalyzes the condensation of isopentenyl diphosphate (IPP) with allylic pyrophosphates generating different type of [illegible]
COG0414	8575	8209	6031	10767	11899	11561	11390	12001	11461	10509	10483	11911	11536	10673	12495	7811	7625	7063	Catalyzes the condensation of pantoate with beta-alanine in an ATP-dependent reaction via a pantoyl-adenylate inte [illegible]
COG0119	19844	19486	15380	41389	41430	42184	34501	41916	38936	32685	42829	35182	41834	39455	41121	20367	18838	17884	Catalyzes the condensation of the acetyl group of acetyl-CoA with 3-methyl-2-oxobutanoate (2-oxoisovalerate) to [illegible]
COG0304	19865	23119	23691	29629	33103	33062	31230	32407	31307	29119	29494	34056	32356	30056	35105	26242	24643	21975	Catalyzes the condensation reaction of fatty acid synthesis by the addition to an acyl acceptor of two carbons from [illegible]
COG0332	11656	12684	11007	16351	17795	17939	18695	18267	17656	16984	16947	19348	17300	16814	19109	14288	13819	11993	Catalyzes the condensation reaction of fatty acid synthesis by the addition to an acyl acceptor of two carbons from [illegible]
COG0289	7780	7921	5945	10841	11820	11518	11682	12287	11788	10444	10759	12263	11390	10505	12360	7635	7303	6743	Catalyzes the conversion of 4-hydroxy- tetrahydrodipicolinate (HTPA) to tetrahydrodipicolinate (By similarity)
COG4689	267	251	154	209	242	203	620	386	390	350	352	415	190	206	203	292	195	190	Catalyzes the conversion of acetoacetate to acetone and carbon dioxide (By similarity)
COG0502	5089	5753	4945	5248	4440	4817	6115	4781	5410	5736	5174	6478	4895	5359	4904	5770	5281	5549	Catalyzes the conversion of dethiobiotin (DTB) to biotin by the insertion of a sulfur atom into dethiobiotin via a radic [illegible]
COG0167	11571	11129	8188	18253	20795	19899	18339	20608	19113	16728	17332	19141	19900	17876	21390	11764	11511	10104	Catalyzes the conversion of dihydroorotate to orotate
COG1109	20109	20401	13841	39334	41300	40923	33964	41681	37912	30896	38632	35257	40547	37070	41364	18801	18050	16724	Catalyzes the conversion of glucosamine-6-phosphate to glucosamine-1-phosphate (By similarity)
COG2160	4466	4836	3459	6840	8973	8323	6906	8599	7493	6081	6820	7295	8135	6900	9128	3655	3747	3382	Catalyzes the conversion of L-arabinose to L-ribulose (By similarity)
COG0041	7862	7965	5965	10849	11951	11611	11640	12060	11535	10384	10746	12003	11534	10640	12476	7748	7403	6821	Catalyzes the conversion of N5-carboxyaminoimidazole ribonucleotide (N5-CAIR) to 4-carboxy-5-aminoimidazole ribo [illegible]
COG2171	6762	6998	5619	7754	9483	8756	9106	10088	9318	8287	8587	9403	8583	7405	9726	6717	6298	5879	Catalyzes the conversion of the cyclic tetrahydrodipicolinate (THDP) into the acyclic N-succinyl-L-2- amino-6-oxopime [illegible]
COG0035	5051	5644	5263	8628	7357	7989	6579	7724	7406	6457	9039	6582	7959	8341	7256	5380	4961	5239	Catalyzes the conversion of uracil and 5-phospho-alpha- D-ribose 1-diphosphate (PRPP) to UMP and diphosphate (By [illegible]
COG1339	123	73	20	5112	4487	4891	1841	4544	3357	2206	5448	1881	4698	4422	3922	93	80	37	Catalyzes the CTP-dependent phosphorylation of riboflavin (vitamin B2) to form flavin mononucleotide (FMN) (By si [illegible]
COG3363	92	72	12	250	245	281	122	140	164	156	167	173	201	233	251	69	63	64	Catalyzes the cyclization of 5-formylamidoimidazole-4- carboxamide ribonucleotide to IMP (By similarity)
COG0043	5216	5307	8098	14028	14496	14357	11949	14981	13228	10451	14347	11808	14245	12907	14201	5943	5659	4908	Catalyzes the decarboxylation of 3-octaprenyl-4-hydroxy benzoate to 2-octaprenylphenol (By similarity)
COG0407	8150	8195	6015	12790	13639	13834	13045	13938	13336	12801	12567	14507	13444	13345	15242	8058	7571	6979	Catalyzes the decarboxylation of four acetate groups of uroporphyrinogen-III to yield coproporphyrinogen-III (By sim [illegible]
COG0284	7823	7918	5651	15877	16417	16473	13246	16526	14835	12421	15934	13800	16205	15003	16376	7680	7225	6775	Catalyzes the decarboxylation of orotidine 5'- monophosphate (OMP) to uridine 5'-monophosphate (UMP) (By simila [illegible]
COG1312	1494	1938	2255	2603	2777	3208	3416	3160	2760	2657	2897	3084	2739	2951	3070	1512	1667	1010	Catalyzes the dehydration of D-mannonate (By similarity)
COG1352	10107	9237	8289	15284	14929	14896	17962	16053	15700	16939	16109	17671	15705	15739	16337	12796	12280	11163	catalyzes the demethylation of specific methylglutamate residues introduced into the chemoreceptors (methyl-accep [illegible]
COG1968	11065	10783	7199	16184	16734	16852	13980	16992	15407	13423	16410	14314	16561	15361	16700	9446	9372	9397	Catalyzes the dephosphorylation of undecaprenyl diphosphate (UPP). Confers resistance to bacitracin (By similarity)
COG0276	7521	7579	5819	10834	10952	10558	11887	11780	11671	10127	10297	12156	10950	10768	11493	7259	6966	6405	Catalyzes the ferrous insertion into protoporphyrin IX (By similarity)
COG1206	3705	2818	947	7740	8794	8239	7171	8543	7758	6689	6760	7636	8312	7291	9017	2782	2990	2283	Catalyzes the folate-dependent formation of 5-methyl- uridine at position 54 (M-5-U54) in all tRNAs (By similarity)
COG2429	0	0	0	0	0	0	0	0	0	0	1	0	0	0	1	0	0	0	Catalyzes the formation of 2-amino-5-formylamino-6- ribofuranosylamino-4(3H)-pyrimidinone ribonucleotide mono [illegible]
COG1211	7575	7472	5566	10756	11841	11631	11163	11763	11323	10080	10269	11667	11528	10669	12417	7321	6901	6676	Catalyzes the formation of 4-diphosphocytidyl-2-C- methyl-D-erythritol from CTP and 2-C-methyl-D-erythritol 4- pho [illegible]
COG2265	11193	12321	11509	11854	13109	12879	12219	12799	12484	11549	11412	12987	12463	11621	13708	11651	11087	11120	catalyzes the formation of 5-methyl-uridine at position
COG1351	3210	2634	912	13008	14349	14264	9100	13762	11369	8622	12557	9842	13910	11936	13894	2605	2591	1831	Catalyzes the formation of dTMP and tetrahydrofolate from dUMP and methylenetetrahydrofolate (By similarity)
COG0220	5216	5862	5252	4269	3404	3609	6340	4290	4886	5450	4841	6069	3763	4521	3836	5703	5348	5458	Catalyzes the formation of N(7)-methylguanine at position 46 (m7G46) in tRNA (By similarity)
COG0192	8243	8294	6030	12632	13936	13973	12737	14094	13256	11422	11992	13534	13472	12356	14536	8051	7724	7021	Catalyzes the formation of S-adenosylmethionine from methionine and ATP
COG1812	242	256	70	5385	4856	5252	2034	4786	3586	2477	5653	2188	5368	4720	4226	232	222	194	Catalyzes the formation of S-adenosylmethionine from methionine and ATP (By similarity)
COG3252	84	97	30	176	118	148	258	168	151	350	269	261	122	170	178	146	84	95	Catalyzes the hydrolysis of methenyl-H(4)MPT( ) to 5- formyl-H(4)MPT (By similarity)
COG0588	3758	4548	5027	1782	1675	1695	3307	2399	2674	2479	2573	3211	1697	1860	1773	3096	2701	3423	Catalyzes the interconversion of 2-phosphoglycerate and 3-phosphoglycerate (By similarity)
COG0696	3609	3588	2538	8945	10123	9674	8687	9910	9071	7749	8189	9318	9682	8731	10548	4616	4624	3481	Catalyzes the interconversion of 2-phosphoglycerate and 3-phosphoglycerate (By similarity)
COG3635	563	567	145	6268	5804	6361	2645	5591	4265	3152	6356	2999	6262	5601	5227	576	538	460	Catalyzes the interconversion of 2-phosphoglycerate and 3-phosphoglycerate (By similarity)
COG1869	668	1104	2852	115	122	121	267	149	125	419	331	217	146	222	157	2053	1762	1763	Catalyzes the interconversion of beta-pyran and beta- furan forms of D-ribose (By similarity)
COG0787	8715	8975	6538	11035	12076	11747	12202	12415	12267	10801	11012	12595	11698	10814	12614	8164	7937	7166	Catalyzes the interconversion of L-alanine and D- alanine. May also act on other amino acids (By similarity)
COG0182	4781	4117	3770	13866	14427	14414	9894	14217	12150	9794	13228	10691	14316	12882	14146	4916	4783	3983	Catalyzes the interconversion of methylthioribose-1- phosphate (MTR-1-P) into methylthioribulose-1-phosphate (MT [illegible]
COG2518	5097	5427	5287	7101	7113	7567	9409	8027	8426	7786	8221	9456	6843	7171	7498	6189	5965	4621	Catalyzes the methyl esterification of L-isoaspartyl residues in peptides and proteins that result from spontaneous de [illegible]
COG1995	4699	6317	6872	4423	3467	3696	7141	4792	5608	5381	5369	6549	4010	4791	4086	5875	5140	5231	Catalyzes the NAD(P)-dependent oxidation of 4- (phosphohydroxy)-L-threonine (HTP) into 2-amino-3-oxo-4- (phosph [illegible]
COG0743	7700	7477	5576	10622	11727	11425	11249	11814	11322	10028	10332	11713	11375	10389	12263	7308	7659	6725	Catalyzes the NADP-dependent rearrangement and reduction of 1-deoxy-D-xylulose-5-phosphate (DXP) to 2-C-meth [illegible]
COG0014	5333	5970	5104	5425	3725	4120	6672	4438	5436	5709	5020	6612	4640	5806	4341	5720	5222	5594	Catalyzes the NADPH dependent reduction of L-gamma- glutamyl 5-phosphate into L-glutamate 5-semialdehyde and [illegible]
COG0136	11362	10997	9302	21917	24390	23723	19081	24366	21572	18077	21912	20107	23344	20593	24439	11661	11260	10022	Catalyzes the NADPH-dependent formation of L-aspartate- semialdehyde (L-ASA) by the reductive dephosphorylation [illegible]
COG0780	5019	3510	1114	9144	10205	9888	8417	10025	9013	8301	8245	9242	9659	8714	10655	3581	3912	3022	Catalyzes the NADPH-dependent reduction of 7-cyano-7- deazaguanine (preQ0) to 7-aminomethyl-7-deazaguanine [illegible]
COG2904	1199	1626	4336	357	227	201	1398	731	1139	830	966	969	238	268	283	2396	1997	2069	Catalyzes the NADPH-dependent reduction of 7-cyano-7- deazaguanine (preQ0) to 7-aminomethyl-7-deazaguanine [illegible]
COG0373	7452	7408	5587	15378	15917	15995	12581	15976	14333	11652	15343	13053	15833	14576	15881	7168	6988	6505	Catalyzes the NADPH-dependent reduction of glutamyl- tRNA(Glu) to glutamate 1-semialdehyde (GSA) (By similarity)
COG1893	5734	6302	11301	6453	5858	6671	7482	6767	7165	7083	6921	7431	6197	6693	6652	7867	7615	7085	Catalyzes the NADPH-dependent reduction of ketopantoate into pantoic acid (By similarity)
COG0259	4891	6456	6931	3894	3291	3454	5463	3750	4589	4577	4262	5340	3566	4309	3795	7096	6708	6288	Catalyzes the oxidation of either pyridoxine 5'- phosphate (PNP) or pyridoxamine 5'-phosphate (PMP) into pyridoxal [illegible]
COG1612	7218	7137	4119	14650	15114	15050	12536	15693	14053	11651	15067	12915	14831	13749	15052	7072	6626	6182	Catalyzes the oxidation of the C8 methyl side group on heme O porphyrin ring into a formyl group (By similarity)
COG3483	3313	3843	2792	774	819	718	2010	1237	1766	1931	1558	1757	720	868	869	3910	3404	3812	Catalyzes the oxidative cleavage of the L-tryptophan (L- Trp) pyrrole ring (By similarity)
COG4857	1388	957	1613	129	154	133	145	123	140	478	130	191	111	116	132	726	837	867	Catalyzes the phosphorylation of methylthioribose into methylthioribose-1-phosphate (By similarity)
COG0061	8381	8353	6129	17262	17015	17100	14750	17329	16051	13443	16820	15349	17182	16365	17125	8277	7908	7235	Catalyzes the phosphorylation of NAD to NADP. Utilizes ATP and other nucleoside triphosphates as well as inorganic [illegible]
COG0237	7603	7754	5853	10624	11690	11349	11357	12015	11509	10262	10519	11983	11315	10520	12211	7439	7069	6576	Catalyzes the phosphorylation of the 3'-hydroxyl group of dephosphocoenzyme A to form coenzyme A (By similarity)
COG1947	8303	7701	5616	10632	11734	11437	11272	11824	11339	10354	10331	11708	11378	10392	12270	7519	7303	6976	Catalyzes the phosphorylation of the position 2 hydroxy group of 4-diphosphocytidyl-2C-methyl-D-erythritol (By simi [illegible]
COG0853	4478	5099	4750	3799	3030	3165	4682	3125	3981	4036	3534	4527	3510	3941	3312	4921	4657	4948	Catalyzes the pyruvoyl-dependent decarboxylation of aspartate to produce beta-alanine (By similarity)
COG1513	661	968	1602	287	229	186	790	367	340	947	908	613	210	279	236	2255	1775	1636	Catalyzes the reaction of cyanate with bicarbonate to produce ammonia and carbon dioxide (By similarity)
COG1151	467	652	1467	1443	784	924	864	783	1036	994	801	1148	1408	1613	883	555	522	537	Catalyzes the reduction of hydroxylamine to form NH(3) and H(2)O (By similarity)
COG1057	7785	7934	5890	10784	11925	11599	11404	12011	11504	10252	10512	11960	11537	10605	12496	7631	7367	6905	Catalyzes the reversible adenylation of nicotinate mononucleotide (NaMN) to nicotinic acid adenine dinucleotide (Na [illegible]
COG0148	8822	9106	9163	21447	21389	21863	15854	21606	18698	15202	21962	16345	21346	19750	20854	8640	8384	7741	Catalyzes the reversible conversion of 2- phosphoglycerate into phosphoenolpyruvate. It is essential for the degrada [illegible]
COG1932	4735	5423	5162	2306	2067	2138	3931	2778	3171	3665	3381	3799	1950	2432	2355	5134	4634	5027	Catalyzes the reversible conversion of 3- phosphohydroxypyruvate to phosphoserine and of 3-hydroxy-2-oxo-4- pho [illegible]
COG3622	9182	12313	10555	8589	10959	10151	10006	10840	9729	9177	9459	10274	9992	8441	11192	10878	10231	10659	Catalyzes the reversible isomerization between hydroxypyruvate and 2-hydroxy-3-oxopropanoate (also termed tartr [illegible]
COG0363	13599	15498	9171	18131	22020	20744	18771	21180	19354	16811	17379	20219	20924	18244	22670	12560	12574	12019	Catalyzes the reversible isomerization-deamination of glucosamine 6-phosphate (GlcN6P) to form fructose 6-phosph [illegible]
COG0039	8569	7743	4539	16298	16798	17050	13811	17307	15541	13435	16659	14655	16619	15596	17024	6582	6756	6372	Catalyzes the reversible oxidation of malate to oxaloacetate (By similarity)
COG1502	16365	16823	20257	18305	19009	19661	23623	21778	22529	20320	20522	23562	18874	18017	20745	21003	19987	18565	Catalyzes the reversible phosphatidyl group transfer from one phosphatidylglycerol molecule to another to form car [illegible]
COG1927	0	0	0	0	0	0	0	0	0	0	0	0	0	0	0	0	0	0	Catalyzes the reversible reduction of methenyl-H(4)MPT( ) to methylene-H(4)MPT (By similarity)
COG4074	0	0	0	0	0	0	0	0	0	0	0	0	0	0	0	0	0	0	Catalyzes the reversible reduction of methenyl- H(4)MPT( ) to methylene-H(4)MPT (By similarity)
COG0563	14932	15852	11325	18655	20928	20144	19645	21230	19790	17287	17921	20223	20067	18229	21692	12967	13409	12481	Catalyzes the reversible transfer of the terminal phosphate group between ATP and AMP. Plays an important role in [illegible]
COG0855	7298	7453	5696	9837	10639	10155	11800	11395	10961	9904	9962	11658	10174	9561	11149	7394	6990	6395	Catalyzes the reversible transfer of the terminal phosphate of ATP to form a long-chain polyphosphate (polyP) (By si [illegible]
COG4106	6316	7472	7493	9321	11449	10959	9741	11020	9744	8757	9368	10535	10398	9202	11740	5683	5543	5231	Catalyzes the S-adenosylmethionine monomethyl esterification of trans-aconitate (By similarity)
COG0141	8272	8595	7708	16160	16716	16864	13527	16826	15114	12728	16146	14176	16536	15190	16778	8170	7998	7322	Catalyzes the sequential NAD-dependent oxidations of L- histidinol to L-histidinaldehyde and then to L-histidine (By s [illegible]
COG0703	8324	8746	7403	12559	14037	14017	12507	13957	13080	11308	11824	13423	13721	12395	14570	8349	7899	7347	Catalyzes the specific phosphorylation of the 3-hydroxyl group of shikimic acid using ATP as a cosubstrate (By similar [illegible]
COG0529	3954	3825	4891	5937	5612	6261	5723	5565	5423	5552	5277	6209	6160	6262	5970	5891	6194	5352	Catalyzes the synthesis of activated sulfate (By similarity)
COG0042	10531	11233	12079	12929	13187	13125	14614	14385	14627	13781	13518	15324	13195	12914	14398	12642	11842	11168	Catalyzes the synthesis of dihydrouridine a modified base found in the D-loop of most tRNAs (By similarity)
COG0518	6230	7510	7383	12933	11459	12598	10387	12014	11077	9605	12623	11022	11941	12360	11709	6994	6302	6493	Catalyzes the synthesis of GMP from XMP (By similarity)
COG0519	7851	7957	5933	15924	16455	16521	13280	16574	14880	12494	16004	13869	16277	15146	16475	7695	7359	6781	Catalyzes the synthesis of GMP from XMP (By similarity)
COG1165	3002	3354	1863	2019	1258	1527	1796	1205	1647	1874	1372	1934	1821	2444	1609	1989	2129	2538	Catalyzes the thiamine diphosphate-dependent decarboxylation of 2-oxoglutarate and the subsequent addition of th [illegible]
COG2037	86	100	31	178	133	170	259	181	155	360	275	269	145	189	198	151	87	97	Catalyzes the transfer of a formyl group from 5-formyl tetrahydromethanopterin (5-formyl-H(4)MPT) to methanofur [illegible]
COG0620	8238	8922	10081	19780	20454	20634	14389	20337	17158	13394	20553	14550	19686	17565	19404	8414	8617	7542	Catalyzes the transfer of a methyl group from 5- methyltetrahydrofolate to homocysteine resulting in methionine for [illegible]
COG0263	4558	5763	5048	4556	3702	4051	5766	4264	4904	5054	4695	5935	4158	4859	4253	5520	4796	5268	Catalyzes the transfer of a phosphate group to glutamate to form glutamate 5-phosphate which rapidly cyclizes to 5- [illegible]
COG0321	6778	7443	5822	14780	15253	15080	12534	15711	14124	11420	15111	13060	15138	13931	15075	7190	6682	6156	Catalyzes the transfer of endogenously produced octanoic acid from octanoyl-acyl-carrier-protein onto the lipoyl do [illegible]
COG2603	790	965	1503	451	374	349	1211	792	698	1129	1250	1131	325	620	418	2175	1820	1672	Catalyzes the transfer of selenium from selenophosphate for conversion of 2-thiouridine to 2-selenouridine at the w [illegible]
COG0153	5260	5698	3813	8153	9736	9141	7818	9273	8500	6887	7616	8330	9252	8342	9900	4285	4350	3772	Catalyzes the transfer of the gamma-phosphate of ATP to D-galactose to form alpha-D-galactose-1-phosphate (Gal-1 [illegible]
COG1424	102	95	11	433	417	484	285	325	313	301	332	354	362	399	420	115	93	65	Catalyzes the transformation of pimelate into pimeloyl- CoA with concomitant hydrolysis of ATP to AMP (By similarit [illegible]
COG4870	361	269	93	362	440	399	672	524	467	906	716	727	431	692	497	319	302	217	cathepsin
COG0053	12637	13048	8333	18688	21313	20250	18912	20562	19281	16655	17411	19742	20521	18324	21867	11689	11641	10765	cation diffusion facilitator family transporter
COG1230	10007	11111	9466	11963	12769	12411	13335	13425	13250	11897	12156	14023	12221	11435	13417	9970	9988	9596	cation diffusion facilitator family transporter
COG3965	252	618	1400	205	11	36	556	115	131	324	133	402	28	110	45	1631	1352	1301	cation diffusion facilitator family transporter
COG3703	1572	1891	3227	6358	5048	5398	4085	5726	5498	3852	7130	3793	5633	5812	4581	2748	2192	2088	cation transport
COG4572	1100	653	1454	946	52	92	979	294	717	709	310	838	496	968	102	456	659	639	Cation transport regulator
COG4756	874	289	82	96	72	70	241	109	92	470	220	120	69	77	86	340	495	389	cation transporter
COG2905	8114	8620	5826	34951	31126	34046	19516	32066	25905	21430	36844	20565	32368	31821	29092	10089	9155	8887	cbs domain and cyclic nucleotide-regulated nucleotidyltransferase
COG1253	21830	24565	14037	22227	25255	24414	22194	24029	22864	21072	20872	24120	24231	22061	26155	18695	17732	19379	CBS Domain protein
COG4846	947	355	81	70	73	100	98	103	141	382	90	108	75	87	133	334	583	590	ccdC protein
COG5175	0	0	0	0	0	0	0	0	0	0	0	0	0	0	0	0	0	0	CCR4-NOT transcription complex subunit 4
COG2134	217	385	1411	31	19	28	49	30	38	86	53	90	21	38	37	253	217	314	cdp-diacylglycerol pyrophosphatase
COG0558	18509	18449	11275	31841	36672	35800	27633	35425	31639	25080	30821	29930	34432	30191	36995	17187	16289	15924	cdp-diacylglycerol-glycerol-3-phosphate 3-phosphatidyltransferase
COG1183	5968	6046	4769	9426	11307	10783	10627	11771	10788	10303	10506	11443	10444	9216	11741	8062	7506	5994	Cdp-diacylglycerol--serine o-phosphatidyltransferase
COG1887	2025	1571	1817	926	874	932	932	965	1055	1485	904	1388	717	1040	889	2356	2201	2149	CDP-glycerol poly(glycerophosphate) glycerophosphotransferase
COG5035	0	0	0	0	0	0	0	0	0	0	0	0	0	0	0	0	0	0	Cell cycle control protein
COG2884	5333	5526	5111	2484	2164	2238	3729	2621	3037	3792	2807	3784	2007	2500	2350	5178	4921	5296	Cell division ATP-binding protein ftsE
COG5019	0	0	0	0	0	0	0	0	0	0	0	0	0	0	0	0	0	0	Cell division control protein
COG5105	0	0	0	0	0	0	0	0	0	0	0	0	0	0	0	0	0	0	cell division cycle 25 homolog
COG0850	2340	2086	3319	1733	755	876	2699	1476	2169	2155	1785	2470	1169	1826	921	3024	2751	2677	Cell division inhibitor that blocks the formation of polar Z ring septums. Rapidly oscillates between the poles of the c [illegible]
COG0772	17813	16519	11950	22289	24628	23942	23731	24724	23921	21957	21944	24890	23787	22060	25748	16097	16802	14917	Cell Division Protein
COG3087	1644	1861	3077	879	673	710	2476	1601	2241	1672	1974	2153	671	917	805	2583	2262	2063	Cell Division Protein
COG1674	14184	14588	9397	13140	14991	14747	13732	14534	14420	13338	13033	14864	14102	13280	15652	11944	11347	12114	cell division protein FtsK
COG2001	7469	7634	5750	9698	11641	11275	10393	11639	10796	9613	10058	10993	10790	9389	12079	7313	7049	6548	Cell division protein mraZ
COG3064	4918	6739	6754	3420	3132	3186	3177	2774	3729	3182	3042	4006	3544	3716	3425	4353	3604	5586	Cell envelope integrity inner membrane protein TolA
COG2184	524	1067	2837	137	120	169	194	174	224	339	209	246	117	152	227	1900	1603	1673	cell filamentation protein
COG5309	633	1356	2865	392	328	282	496	404	515	816	489	709	275	342	361	3518	2804	2772	cell wall
COG2247	3385	1125	290	234	279	288	243	285	448	1496	237	419	164	324	243	1057	1721	1423	Cell wall binding repeat 2-containing protein
COG0773	11441	11611	9771	18157	20897	19989	19934	21555	20244	17483	18611	20619	19747	17545	21808	12334	11830	10179	cell wall formation (By similarity)
COG0812	7761	7874	5917	10806	11946	11595	11533	12179	11696	10389	10648	12183	11532	10605	12496	7606	7284	6757	cell wall formation (By similarity)
COG1181	10010	10420	9572	18665	18402	18634	16115	18727	17367	15577	18855	17371	18622	18266	18677	11113	10965	10004	cell wall formation (By similarity)
COG0766	10303	10562	7145	10987	12146	11761	11958	12426	12078	10919	10948	12497	11656	10773	12654	9324	8973	9113	Cell wall formation. Adds enolpyruvyl to UDP-N- acetylglucosamine (By similarity)
COG0771	7777	7889	5915	10791	11896	11547	11520	12148	11672	10355	10629	12160	11487	10573	12443	7618	7295	6755	Cell wall formation. Catalyzes the addition of glutamate to the nucleotide precursor UDP-N-acetylmuramoyl-L-alanin [illegible]
COG0707	9666	9623	6632	13128	14370	14225	13982	14209	13849	12615	12753	14905	13886	13066	15335	9277	8917	8058	Cell wall formation. Catalyzes the transfer of a GlcNAc subunit on undecaprenyl-pyrophosphoryl-MurNAc-pentapept [illegible]
COG3773	4488	2895	2254	861	734	716	1272	1024	1159	2791	1104	1506	593	715	832	3732	4137	4011	Cell wall hydrolase
COG4758	1764	592	119	179	234	217	135	159	199	735	167	182	160	171	173	574	991	923	Cell wall-active antibiotics response protein
COG5297	1067	749	374	1376	1774	1728	921	1146	1553	1084	1061	1513	1445	1686	1679	708	571	703	Cellobiohydrolase
COG1321	9787	11073	6470	24446	24919	25777	14024	24019	19418	14696	24813	14953	24830	22299	23437	7556	7351	8222	cellular manganese ion homeostasis
COG3510	284	765	1562	149	141	118	271	164	170	411	349	318	126	237	170	1716	1434	1392	cephalosporin hydroxylase
COG5058	0	0	0	0	0	0	0	0	0	0	0	0	0	0	0	0	0	0	ceramide synthase
COG5561	51	56	4	214	193	228	178	208	172	177	194	200	201	188	198	72	73	39	CGGC domain
COG5607	4364	5022	3345	7716	8832	8342	8085	8876	8009	6544	7142	7914	8493	7523	9094	4996	4685	4502	chad domain containing protein
COG1272	6077	6433	5218	6092	7692	6853	6678	7865	7122	6666	6712	7352	6875	5781	8035	6216	5722	5734	Channel protein (Hemolysin III family
COG1970	6232	7053	5680	8255	9894	9281	9682	10102	9500	8227	8701	9655	8984	7636	10238	6943	6261	5931	Channel that opens in response to stretch forces in the membrane lipid bilayer. May participate in the regulation of [illegible]
COG0580	11296	14798	15628	17759	18834	19105	15095	18536	16475	13527	17311	15380	18658	16951	18786	14116	12827	13415	Channel that permits osmotically driven movement of water in both directions. It is involved in the osmoregulation [illegible]
COG3121	2195	3970	10109	371	286	230	1589	909	1646	1441	1223	1426	234	289	308	8662	8023	7238	Chaperone
COG1371	507	466	127	6046	5606	6068	2532	5377	4188	2933	6202	2843	5873	5421	4963	470	444	457	Chaperone or modulator of proteins involved in DNA or RNA processing (By similarity)
COG0298	3063	2769	2106	12904	13556	13517	8970	13434	11274	8846	12854	9612	13279	12045	13236	2796	2656	2009	chaperone-mediated protein complex assembly
COG5491	369	219	60	15333	13458	14664	5523	13629	10068	6600	16339	5637	14088	13260	11767	279	231	111	Charged multivesicular body protein
COG5278	1077	543	170	1179	579	645	782	539	878	952	608	908	963	1238	678	593	816	630	CHASE3 domain
COG0643	9034	9433	10771	18068	14379	14690	17867	15862	17216	16330	15789	18664	16914	18489	15712	14955	13988	12671	CheA signal transduction histidine kinase
COG1776	2243	1011	292	1394	837	909	1073	719	1081	1555	946	1044	1489	1738	893	1054	1491	1530	CheC --inhibitor of MCP methylation
COG2882	2100	1773	3013	803	607	642	1857	1238	1570	1687	1555	1690	747	796	678	2705	2514	2510	chemotaxis
COG1406	3342	2367	637	6732	8748	8234	6063	8372	7030	6067	6331	6711	8238	6442	8869	2338	2673	1882	chemotaxis protein
COG2469	0	0	0	1	0	2	0	0	0	0	0	0	1	2	0	0	0	0	Chemotaxis signal transduction system protein F from archaea
COG0835	14708	14924	17695	23402	22721	22073	25173	24151	24053	24371	23597	26160	23772	22945	24170	23629	21940	20087	chew protein
COG3325	6412	4978	2683	11549	15454	13742	11771	14710	12817	10906	11709	12501	13621	10936	15574	4827	5316	3691	chitinase
COG3469	2882	2493	1976	5872	7868	7098	5534	7480	6345	4932	5684	5846	6958	5390	7694	3576	4116	2651	chitinase
COG3979	1238	1068	1656	384	465	432	514	373	464	446	540	532	428	486	455	894	875	1029	chitinase
COG4845	227	431	330	373	470	466	316	367	442	415	368	546	369	518	525	351	404	141	chloramphenicol
COG3896	146	223	192	32	34	40	76	53	63	61	47	90	45	44	54	129	113	78	Chloramphenicol phosphotransferase family protein
COG0038	9118	9435	8079	19227	21638	20825	21109	22060	20858	17547	18553	21402	20845	18685	22790	8173	8587	6174	chloride channel
COG3253	5612	5121	2170	7338	9502	8924	6546	8691	7684	5918	6568	7070	8572	6712	9476	3916	4083	3917	chlorite dismutase
COG5263	20	15	10	13	74	51	17	18	22	206	42	81	31	22	20	91	25	18	choline binding protein
COG3049	497	416	435	409	467	472	1097	645	888	716	600	933	638	484	492	334	427	256	Choloylglycine hydrolase
COG1605	7786	9422	6446	9238	11361	10782	10148	11374	10586	9062	9592	10673	10409	8854	11685	9344	8215	8579	Chorismate mutase
COG4401	1170	528	169	1339	562	685	1031	457	861	987	563	1015	1033	1475	662	520	756	821	chorismate mutase
COG2059	8445	6226	3685	8917	9953	9000	12695	10714	11054	11346	10994	11654	9536	8965	10383	7519	8299	6044	Chromate
COG4275	872	1357	1808	735	743	724	1080	766	801	1432	1111	1237	727	737	878	2508	2075	1971	Chromate resistance
COG3332	1072	1189	1731	677	562	603	1552	1020	1455	1392	1212	1372	565	631	654	2311	1751	1780	Chromosome 22 open reading frame 25
COG2106	123	73	20	5111	4486	4888	1841	4543	3356	2200	5446	1879	4696	4420	3922	93	77	37	chromosome 9 open reading frame 114
COG1256	5208	4642	4180	8091	9783	9257	8661	10083	9098	8183	8566	9049	9138	7662	10055	5642	5219	4433	ciliary or bacterial-type flagellar motility
COG0467	4381	4303	2833	21312	20212	20680	13714	20003	17372	13195	20216	14561	21207	20240	19690	5518	4487	4105	Circadian clock protein KaiC
COG3493	280	405	1407	43	21	16	454	212	213	114	181	161	26	24	26	253	230	323	citrate carrier protein
COG2301	8010	10339	7558	3727	3596	3740	5707	4137	5360	5039	5297	5431	3392	3728	3907	7973	6403	8751	citrate lyase
COG0372	14023	13771	9938	25374	25322	26175	21952	26676	24759	19826	27061	21534	25146	23550	25096	13407	12331	11997	citrate synthase
COG2851	2293	3869	5675	139	119	76	704	337	425	731	469	531	146	238	156	4628	3983	4587	Citrate transporter
COG0137	7224	7920	6054	16052	16623	16732	13380	16677	15034	12270	16097	14037	16454	15251	16637	7579	7086	6541	Citrulline--aspartate ligase
COG3700	0	0	0	0	0	0	0	0	0	0	0	0	0	0	0	0	0	0	class B acid phosphatase
COG0235	18878	23145	23620	19063	22483	21778	20685	22990	21661	18357	19758	20914	21208	18789	23106	20974	19199	20820	Class II aldolase adducin
COG1991	0	0	0	0	0	0	0	0	0	0	0	0	0	0	0	0	0	0	Class III signal peptide
COG1236	3448	4099	3207	15415	13511	14478	11340	14384	12649	10789	16689	11411	14383	14396	13030	5085	4886	3900	cleavage and polyadenylation
COG5161	180	552	1351	0	0	0	3	2	4	134	32	30	0	0	0	1572	1302	1260	cleavage and polyadenylation
COG1637	4593	4463	3184	9415	10601	9956	6199	10247	8335	6329	9758	6424	10033	8295	9922	3124	3186	3286	Cleaves both 3' and 5' ssDNA extremities of branched DNA structures (By similarity)
COG0740	13651	13529	9750	16343	15624	15524	17025	15610	16398	15420	14761	17799	16468	16934	16553	13350	12635	12179	Cleaves peptides in various proteins in a process that requires ATP hydrolysis. Has a chymotrypsin-like activity. Plays [illegible]
COG2195	4547	4620	5788	7285	9355	8654	6909	8689	7456	6695	6945	7501	8653	7083	9428	4091	4452	3490	Cleaves the N-terminal amino acid of tripeptides (By similarity)
COG1989	6992	6925	5188	10158	11208	10989	10210	11212	10760	9280	9591	10742	10833	9809	11723	6865	6475	6399	Cleaves type-4 fimbrial leader sequence and methylates the N-terminal (generally Phe) residue (By similarity)
COG4670	1710	1842	3319	398	291	289	1428	694	644	1605	1262	952	303	399	347	2803	2409	2330	CoA transferase having broad substrate specificity for short-chain acyl-CoA thioesters with the activity decreasing wh [illegible]
COG1042	6972	7631	4710	11562	10585	11483	9669	10996	11943	7523	11733	9308	11264	10701	10060	5439	4487	5681	CoA-binding domain protein
COG1832	6186	6230	3695	13275	14757	14537	9930	14173	11878	9924	13644	10517	14084	12218	14450	5182	4988	4735	CoA-binding domain protein
COG1924	426	468	115	1504	1473	1760	1022	1255	1166	1274	1191	1418	1583	1431	1629	512	432	474	coA-substrate-specific enzyme activase
COG3580	168	218	40	505	578	602	379	474	429	490	443	577	708	538	587	235	178	257	coA-substrate-specific enzyme activase
COG3581	190	235	49	570	629	676	426	541	480	533	489	622	765	585	632	259	197	274	coA-substrate-specific enzyme activase
COG2073	741	1147	1708	1265	344	428	1758	765	1152	1391	1149	1482	785	1341	529	2292	1849	1768	cobalamin (vitamin B12) biosynthesis CbiG protein
COG2138	3823	3755	1730	3862	2435	2722	4205	2554	3279	3534	3041	4022	3361	4165	2735	2804	2767	3349	cobalamin (vitamin b12) biosynthesis cbix protein
COG5012	2148	1037	272	1718	1725	2025	1387	1592	1511	2602	1613	1955	1700	1967	2158	1112	1333	1278	cobalamin b12-binding domain protein
COG0523	10106	11113	14066	9383	5934	6604	12812	7517	9689	10810	8942	11589	7729	9796	7299	13590	12202	12179	cobalamin synthesis protein
COG4822	28	23	9	98	75	107	68	93	77	104	74	84	92	69	76	40	29	26	cobalt chelatase
COG0619	9065	9600	4823	16241	13187	14373	8566	12429	11821	8605	14405	9033	14936	15415	12150	5894	5808	7490	Cobalt transport protein
COG3442	1894	2554	1364	1429	927	1052	774	580	1063	684	718	969	1351	1712	983	1458	1251	1925	CobB/CobQ-like glutamine amidotransferase domain
COG1148	382	379	109	1330	1172	1540	967	1223	1048	1115	1101	1138	1286	1231	1320	388	418	431	CoB--CoM heterodisulfide reductase activity
COG1035	357	246	107	6406	4989	5479	2938	4997	4212	2896	6014	2939	5745	5846	4548	265	296	202	Coenzyme F420 hydrogenase
COG4696	319	515	430	55	37	23	105	47	87	173	95	138	58	104	46	340	430	106	cof family
COG5234	0	0	0	0	0	0	0	0	0	0	0	0	0	0	0	0	0	0	Cofactor D
COG5134	0	0	0	0	0	0	0	0	0	0	0	0	0	0	0	0	0	0	Coiled-coil domain-containing protein
COG1278	32809	34132	28701	37848	47318	43939	41860	48368	44362	37522	38986	43228	42939	35747	47813	32460	31017	29732	Cold shock protein
COG3326	1431	1516	2047	238	179	228	502	290	308	1159	743	578	242	412	295	2376	2467	2080	Cold-shock dna-binding domain protein
COG1286	5071	4658	4028	8103	9910	9254	8708	10276	9394	8390	8633	9469	9061	7629	10122	5585	5182	4359	Colicin v production protein
COG4537	1630	1052	250	2773	3191	3830	2414	3142	2814	3050	2796	3073	3150	3354	3788	972	1096	1006	comG operon protein 3
COG4940	752	232	55	12	12	12	19	12	19	326	17	18	8	8	12	205	442	443	comg operon protein 6
COG1058	5409	4649	3029	14953	15582	15565	12329	15552	13812	11616	15019	12640	15558	14162	15593	4539	4661	3629	competence damage-inducible protein
COG1546	5773	6434	5717	6098	6100	6794	6921	6375	6385	6443	6163	7239	5996	6211	6710	6069	5727	5741	competence damage-inducible protein
COG1040	7720	7884	5911	10769	11926	11602	11410	11977	11465	10223	10499	11930	11513	10572	12473	7603	7282	6750	Competence protein
COG1555	9361	8905	5738	15735	18787	17490	16195	18378	16790	13698	14753	16581	17723	15056	18809	8565	8394	7380	Competence protein
COG4098	895	321	71	16	17	17	25	16	26	337	31	29	11	16	17	313	555	545	Competence protein
COG4469	902	343	77	327	386	388	336	288	307	642	376	386	302	347	384	310	535	609	Competence protein
COG4903	777	262	84	17	19	17	22	14	21	331	19	22	11	11	16	224	531	604	Competence transcription factor
COG5156	0	0	0	0	0	0	0	0	0	0	0	0	0	0	0	0	0	0	complex subunit 10
COG0777	7876	8638	8482	10451	11546	11151	11882	12047	11498	10701	10905	12082	11105	10236	12103	9535	8894	8405	Component of the acetyl coenzyme A carboxylase (ACC) complex. Biotin carboxylase (BC) catalyzes the carboxylation [illegible]
COG5031	255	271	98	1304	224	333	1635	537	878	1016	623	1403	738	1319	412	264	280	329	Component of the coenzyme Q biosynthetic pathway. May play a role in organizing a multi-subunit COQ enzyme com [illegible]
COG0155	8594	9017	7517	17306	17231	17341	15289	17787	16630	14013	17280	15825	17364	16830	17567	9981	10101	8782	Component of the sulfite reductase complex that catalyzes the 6-electron reduction of sulfite to sulfide. This is one o [illegible]
COG0369	5305	5661	7117	8078	9123	8306	10168	9927	10206	8631	8985	10605	8585	8112	9812	7502	6793	5780	Component of the sulfite reductase complex that catalyzes the 6-electron reduction of sulfite to sulfide. This is one o [illegible]
COG0352	12138	11517	8199	23320	25632	25064	21946	26149	23458	19945	23797	22470	24691	21915	25790	12593	12620	10339	Condenses 4-methyl-5-(beta-hydroxyethyl)thiazole monophosphate (THZ-P) and 2-methyl-4-amino-5-hydroxymethyl [illegible]
COG3701	3053	2567	907	6408	8317	7485	7220	8441	7509	5892	6606	7087	7371	5899	8325	2520	2482	1605	Conjugal transfer protein
COG4959	1860	1285	527	1217	1288	1266	1962	1456	1764	1834	1562	1703	1129	1092	1269	1231	1141	996	Conjugal transfer protein
COG5268	800	707	363	488	421	361	1664	950	1324	917	1054	1092	342	380	458	653	489	422	Conjugal transfer protein
COG4962	14082	16080	8740	15068	19066	17768	16342	19015	17595	13762	15556	16322	17370	14146	19193	12568	12096	12501	conjugation
COG2948	4016	3344	1228	7374	9137	8427	9036	9939	9533	7574	8321	8966	8157	6700	9449	3327	2996	2312	conjugation trbi family protein
COG2343	6572	7560	3667	8781	8963	8461	9362	9291	9060	7124	7893	8974	9063	8512	9230	5316	4751	5283	conserved domain protein
COG4443	21	27	26	3	4	3	3	3	4	11	4	12	3	4	3	19	70	128	conserved domain protein
COG4295	338	282	371	548	610	652	400	401	484	408	394	558	529	461	701	300	440	298	conserved hypothetical protein
COG3242	700	780	1547	698	478	550	1034	912	859	1026	1069	1264	480	682	593	812	554	669	conserved inner membrane protein
COG1432	2834	3227	2585	6131	3868	4244	6530	4259	5544	5362	4785	6792	5007	6489	4488	3905	3523	3199	conserved protein
COG1434	8276	8468	7490	13546	13475	13403	13994	14016	14005	11804	12075	14415	13921	13755	14223	8143	7818	7365	conserved protein
COG1479	854	848	397	2053	1376	1408	2379	1350	1703	1786	1678	2382	1741	2061	1481	835	884	691	conserved protein
COG1683	633	925	1614	257	261	260	715	448	821	439	430	534	232	227	234	1794	1536	1537	conserved protein
COG1721	18199	20005	11518	24872	26737	27487	21475	24615	23382	21644	23799	24160	26587	23848	27751	17680	16870	17597	Conserved protein
COG1801	10537	10399	8119	27420	29570	29354	22598	29786	25897	20878	28426	23070	28548	25663	29431	11793	11226	9722	conserved protein
COG1912	2968	2697	979	13230	13971	13728	9520	13488	11384	8777	12534	9963	13846	12329	13381	2684	2881	1678	conserved protein
COG2013	2602	2206	1108	10057	8459	9292	5913	8357	7710	5886	9319	6227	9412	9812	8152	2384	2444	1911	conserved protein
COG2135	7609	8408	6310	8854	10685	10001	10126	10951	10319	9301	9462	10554	9690	8574	10966	9100	7429	7518	conserved protein
COG2155	974	442	130	149	169	132	155	149	151	494	163	199	138	134	185	496	628	600	Conserved Protein
COG2307	5811	6723	5197	9554	10787	10399	10128	10714	9906	8802	9184	10230	10433	9010	11311	6520	5998	5957	Conserved Protein
COG2308	6544	7936	6930	10459	11666	11297	11216	11670	11007	10324	10529	11682	11272	10134	12450	9030	7838	7659	conserved protein
COG2454	261	225	354	339	446	440	329	345	352	332	326	460	410	371	548	233	398	209	Conserved Protein
COG2928	1650	1420	846	2783	2030	2209	4261	2968	3850	3424	3536	4059	2583	3120	2507	1481	1276	886	conserved protein
COG3068	203	370	1395	0	1	0	2	3	1	9	4	3	0	5	0	223	205	292	conserved protein
COG3084	203	366	1389	0	1	0	1	1	0	7	3	2	0	1	0	216	202	292	conserved protein
COG3159	1237	1427	2957	828	524	602	1996	1313	1627	1591	1665	1717	516	743	661	2431	1976	2007	Conserved Protein
COG3272	573	1162	2818	523	653	668	380	474	440	554	447	565	689	525	538	2009	1730	1771	conserved protein
COG3339	3653	4275	3460	2584	1741	1757	3304	1804	2593	3098	2326	3377	2223	2640	1950	4205	3895	4444	conserved protein
COG3365	0	0	0	0	0	0	0	0	0	0	0	0	0	0	0	0	0	0	conserved protein
COG3535	1307	1862	2305	686	769	803	604	582	611	740	553	759	689	661	695	2402	2065	2069	conserved protein
COG3538	2528	2080	848	5893	7777	7089	5993	7646	6432	4937	5752	6082	6988	5395	7775	2096	2350	1309	conserved protein
COG3592	94	329	334	52	15	25	157	53	75	205	150	132	68	93	45	53	298	38	conserved protein
COG4033	0	0	0	0	0	0	0	0	0	0	0	0	0	0	0	0	0	0	Conserved Protein
COG4044	0	0	0	1	0	0	0	0	0	2	0	0	0	0	2	0	0	0	conserved protein
COG4048	0	0	0	0	0	0	0	0	0	0	0	0	0	0	0	0	0	0	conserved protein
COG4077	0	0	0	0	0	0	0	0	0	0	0	0	0	0	0	0	0	0	conserved protein
COG4081	0	0	0	0	0	0	0	0	0	0	0	0	0	0	0	0	0	0	Conserved Protein
COG4289	721	784	491	371	472	352	1052	593	636	846	700	940	394	708	478	518	561	338	conserved protein
COG4575	2872	4481	11542	1672	1382	1759	3610	2681	2813	3169	3246	3188	1372	2014	1965	6916	5750	5920	Conserved Protein
COG4804	1526	2180	3458	2440	1867	1900	3202	2023	2503	2743	2419	3354	2385	3023	2288	2966	3015	2396	Conserved Protein
COG4837	754	233	57	13	14	14	19	12	18	327	17	17	8	9	12	209	449	456	conserved protein
COG4866	8	4	4	7	7	13	6	9	11	36	14	28	8	9	9	27	8	4	conserved protein
COG5282	3634	4939	2629	863	1055	1129	670	659	904	579	664	813	970	973	1044	2687	2288	3683	Conserved Protein
COG5383	605	1172	3014	71	41	39	273	102	109	349	255	206	45	68	45	2026	1719	1707	Conserved Protein
COG5530	247	352	259	312	362	370	306	274	292	260	228	362	328	208	425	305	319	272	conserved protein UCP01500
COG4394	783	889	1490	449	226	263	1469	822	1051	1042	1262	1138	244	455	286	2022	1639	1533	conserved protein UCP015557
COG3876	3775	3154	1012	7881	10009	9486	7426	9363	8131	7171	7499	8086	9010	7732	10132	2929	3428	1986	conserved protein UCP016719
COG4079	0	0	0	0	0	0	0	0	0	0	0	0	0	0	0	0	0	0	conserved protein UCP019262
COG4018	0	0	0	0	0	0	0	0	0	0	0	0	0	0	0	0	0	0	conserved protein UCP019375
COG3784	532	662	1491	388	291	315	593	497	559	721	461	658	283	444	394	493	446	564	conserved protein UCP025560
COG4334	256	266	218	304	363	373	274	280	334	242	279	348	358	378	396	162	192	125	conserved protein UCP028498
COG5345	375	807	1572	132	82	82	240	204	267	279	162	288	69	73	78	1706	1404	1340	conserved protein UCP029693
COG4817	1011	373	91	100	163	132	78	90	124	379	98	138	85	106	105	362	596	579	conserved protein UCP029876
COG3749	1220	1431	1856	769	570	557	1992	1148	1468	1755	1720	1614	504	645	703	2492	2708	1956	conserved protein ucp030820
COG5458	419	586	320	473	476	438	595	538	524	781	576	734	368	417	525	560	387	408	conserved protein UCP032025
COG4898	1317	866	657	612	660	692	1314	901	896	1433	1007	1255	579	822	850	888	981	714	conserved protein UCP033199
COG4198	1491	1351	695	8223	8080	8796	4282	7314	6191	4891	8120	5208	8280	7800	7989	1635	1558	1374	conserved protein UCP033563
COG5400	409	571	320	427	420	382	563	489	489	716	539	678	319	371	473	540	378	386	conserved protein UCP033924
COG4010	0	0	0	0	0	0	0	0	0	0	0	0	0	0	0	0	0	0	conserved protein UCP037052
COG4345	0	0	0	0	0	0	0	0	0	0	0	0	0	0	0	0	0	0	conserved protein UCP037214
COG4719	9	23	9	10	6	2	18	22	12	52	27	21	3	15	5	22	24	16	conserved repeat domain protein
COG0761	8053	7807	5757	11047	12225	11889	12033	12352	11877	10459	10797	12316	11802	10705	12816	7734	8023	7072	Converts 1-hydroxy-2-methyl-2-(E)-butenyl 4-diphosphate into isopentenyl diphosphate (IPP) and dimethylallyl diph [illegible]
COG1270	2339	2142	2049	2639	1691	1882	3622	2136	2509	3405	3145	3172	2273	2724	2012	3396	2962	2790	Converts cobyric acid to cobinamide by the addition of aminopropanol on the F carboxylic group (By similarity)
COG0109	7948	8334	7033	20139	20017	20358	14626	20571	17766	14280	20818	15199	19955	18582	19435	9128	8472	8019	Converts heme B (protoheme IX) to heme O by substitution of the vinyl group on carbon 2 of heme B porphyrin ring [illegible]
COG3010	253	262	200	891	60	90	868	186	544	395	279	719	499	969	111	183	228	146	Converts N-acetylmannosamine-6-phosphate (ManNAc-6-P) to N-acetylglucosamine-6-phosphate (GlcNAc-6-P) (By si [illegible]
COG1103	0	0	0	0	0	0	0	0	0	0	0	0	0	0	0	0	0	0	Converts O-phospho-L-seryl-tRNA(Cys) (Sep-tRNA(Cys)) to L-cysteinyl-tRNA(Cys) (Cys-tRNA(Cys)) (By similarity)
COG1921	6265	5186	4157	12820	16755	15509	11887	15959	13700	11014	12270	12760	15234	12073	16945	4834	5118	3726	Converts seryl-tRNA(Sec) to selenocysteinyl-tRNA(Sec) required for selenoprotein biosynthesis (By similarity)
COG1441	0	0	0	8	8	12	10	14	11	9	14	12	20	9	24	0	0	0	Converts SHCHC to OSB (By similarity)
COG2407	1843	1135	279	3087	3470	3993	2814	3198	2851	2639	2768	2955	3298	3411	3610	1168	1425	1016	Converts the aldose L-fucose into the corresponding ketose L-fuculose (By similarity)
COG5159	2	5	2	2	4	10	1	2	3	5	2	6	4	4	9	4	1	4	cop9 signalosome complex subunit
COG3609	3671	4495	4782	10565	11409	10805	9044	12066	10106	8203	11556	8837	10749	9403	11097	4826	4198	3415	CopG Arc MetJ family
COG4710	283	192	85	115	87	100	507	272	245	274	318	273	100	118	140	235	216	156	CopG domain protein DNA-binding domain protein
COG3142	4094	4795	3562	4373	6112	4916	5568	6210	5562	4613	4907	5459	5287	4034	6042	3195	3308	3059	copper homeostasis protein cutc
COG3241	804	1198	1530	816	732	890	1674	1157	1530	1351	1224	1627	812	1029	1084	2002	1876	1574	copper ion binding
COG1276	2840	2471	3406	2280	2540	2630	2374	2200	2280	2355	2168	2571	2308	2264	2578	3401	3988	3261	copper resistance
COG3667	2728	2436	2034	4826	6390	5309	5589	6735	6257	4969	5252	5963	5508	4293	6362	3403	3189	2510	copper resistance
COG2372	4920	6549	5716	1511	1620	1683	2076	1616	1810	1554	1561	1988	1462	1547	1701	5175	5254	6034	Copper resistance protein
COG0635	11003	11626	11165	14434	14732	14681	16696	15601	15420	15279	14733	16990	14645	14415	16024	13413	12586	11556	Coproporphyrinogen III oxidase
COG0816	7671	7734	5903	11241	11460	11137	12028	11911	11856	10415	10542	12434	11400	11136	12147	7473	7200	6631	Could be a nuclease that resolves Holliday junction intermediates in genetic recombination (By similarity)
COG1273	6393	7248	4896	7653	9678	9055	8527	9659	8539	7738	8127	8637	8779	7356	10052	6613	6404	5807	Could be involved in DNA repair (By similarity)
COG0759	7304	7133	4442	10199	11251	10820	10795	11326	10795	9428	9801	11079	10891	9833	11633	5810	5730	5307	Could be involved in insertion of integral membrane proteins into the membrane (By similarity)
COG2088	1823	740	152	572	673	702	369	448	461	1094	439	576	760	601	635	722	1081	1126	Could be involved in septation (By similarity)
COG3045	1015	1568	3124	620	551	530	1329	968	828	1285	1314	1267	469	655	601	2587	2033	2033	Crea protein
COG1857	271	275	76	5509	4957	5374	2141	4923	3684	2525	5759	2303	5276	4818	4337	274	304	307	CRISPR-associated autoregulator DevR family
COG2254	123	73	20	5111	4486	4888	1841	4543	3356	2200	5445	1879	4696	4420	3921	93	77	37	CRISPR-associated HD domain protein
COG1353	931	744	219	8376	6447	7029	4133	6284	5651	4035	7187	4632	7532	8059	5941	746	818	581	crispr-associated protein
COG1517	156	112	36	5186	4576	4998	1893	4589	3416	2266	5500	1952	4766	4509	4002	120	116	79	CRISPR-Associated Protein
COG3649	276	270	85	632	747	788	521	529	552	494	564	661	879	714	774	244	252	228	CRISPR-Associated Protein
COG4006	147	100	32	5166	4560	4965	1888	4596	3410	2263	5490	1945	4762	4484	4040	124	116	82	CRISPR-associated protein APE2256 family
COG1468	697	592	202	7389	5903	6443	4124	5835	4960	3617	6747	4042	6663	6715	5370	593	659	568	crispr-associated protein cas4
COG1688	176	128	26	5316	4676	5110	2014	4740	3518	2356	5627	2074	4874	4608	4112	166	150	81	crispr-associated protein cas5
COG1769	163	112	41	326	393	416	255	272	317	271	284	349	400	406	438	119	126	101	CRISPR-associated protein Cmr3
COG1421	163	216	49	470	556	582	346	421	384	377	362	480	646	480	488	192	195	180	CRISPR-associated protein Csm2 family
COG1337	1036	770	221	18424	14987	16375	8081	15197	12166	8353	17863	8361	16516	16575	13323	808	846	564	Crispr-associated ramp protein
COG3237	3553	3989	6436	3568	3632	4008	4449	4406	4291	4123	3945	4805	3643	3820	3879	4345	4002	3886	Csbd family
COG5647	0	0	0	0	0	0	0	0	0	0	0	0	0	0	0	0	0	0	cullin 1
COG1917	23427	24125	18753	52069	60918	59372	45820	59424	52716	40656	51716	47708	57546	49019	61116	22924	22625	18495	Cupin 2 Conserved Barrel Domain Protein
COG3837	1341	1629	1911	2310	1376	1589	3061	1940	2319	2691	2342	2840	1750	2341	1778	2736	2343	2335	Cupin 2 Conserved Barrel Domain Protein
COG4101	580	800	642	576	691	646	984	730	766	631	658	827	587	630	655	576	464	393	Cupin 2 Conserved Barrel Domain Protein
COG2850	1730	1787	3180	1023	680	690	2954	1629	2188	2211	2383	2191	705	889	847	2890	2342	2431	Cupin 4 family protein
COG3542	1083	1469	1913	585	598	487	1223	786	834	1490	1158	1209	558	832	728	2418	2045	2006	Cupin superfamily (DUF985)
COG1462	1476	2020	3076	2690	2648	3079	3474	3196	2968	2880	3178	3108	2552	2552	3008	2845	2539	2305	Curli production assembly transport component CsgG
COG2807	9964	14741	19390	1640	1691	1728	2542	1792	2386	2997	2238	2522	1621	1599	1908	13623	12672	15213	cyanate catabolic process
COG4242	3743	3614	1896	4795	3278	3837	4690	3541	3909	3614	3308	4469	4125	5089	3712	2641	2938	2975	Cyanophycinase

COG2867	3468	4430	4548	1715	1478	1523	3243	2408	2814	2778	2788	3199	1327	1472	1693	4499	3665	4380	Cyclase dehydrase
COG4276	5140	5087	2413	8078	9650	9118	7542	9163	8355	6881	7314	8062	9299	8145	9964	3958	4393	3625	Cyclase dehydrase
COG5637	9295	12261	6652	9621	10994	10595	9934	10508	9913	8138	8701	10203	10396	9370	11416	8988	8179	9968	Cyclase dehydrase
COG1878	10638	10833	7092	21079	22463	22800	14010	21107	18597	14380	21053	15281	21909	19173	21495	9422	9571	9235	Cyclase family
COG3300	2130	1612	490	4242	5979	4839	4675	5811	5317	4066	4567	4800	5120	3701	5801	1668	1580	974	cyclic nucleotide biosynthetic process
COG4615	1168	1683	4323	962	712	904	2060	1247	1155	1453	1398	1528	735	1010	979	2533	2181	2205	Cyclic peptide transporter
COG2230	12855	15202	11737	18619	19501	19844	19283	20420	18284	17435	20482	19065	18779	17504	20027	14035	13450	13725	cyclopropane-fatty-acyl-phospholipid synthase
COG3790	205	367	1420	45	3	14	46	22	12	42	114	18	8	34	9	222	226	293	cyd operon protein YbgE
COG4890	629	684	1604	209	114	89	847	459	941	260	390	561	92	118	130	352	298	365	Cyd operon protein YbgT
COG0626	21088	21027	15422	24480	30357	28482	26641	30286	27605	25741	25837	27839	27631	23767	31183	19190	18783	17514	cystathionine
COG0031	22552	22815	18198	40377	42623	42084	34270	42183	38205	32866	39618	36297	41666	38377	42765	23357	22285	20833	cysteine biosynthetic process from serine
COG0520	13802	15416	12707	25532	25514	26496	20953	23992	23030	20297	22966	23220	25873	25326	26336	15712	15430	14659	cysteine desulfurase
COG1104	15559	15641	11345	26922	27354	26765	25178	27515	25709	22546	25664	25565	27762	25849	27673	15065	14960	13791	cysteine desulfurase
COG5553	1705	1595	2935	1358	448	448	3048	1381	3122	1387	1535	2395	835	1304	531	2320	1830	1843	cysteine dioxygenase type I
COG4988	3896	4471	5967	685	705	748	1002	725	1182	1066	675	1002	658	678	721	2646	2575	3532	cysteine transport
COG2158	5	4	3	13	10	10	20	24	25	22	19	26	11	15	18	4	9	7	Cysteine-rich small domain protein
COG0215	10222	10854	7394	17356	18148	18253	14504	17840	16315	13601	17255	15349	17817	16528	18000	9718	8959	8981	Cysteinyl-tRNA synthetase
COG0295	7440	6815	4309	13196	14637	14495	10078	14324	12107	10272	13316	10619	13929	12266	14404	5408	5636	5272	cytidine deaminase
COG0283	7662	7846	5883	10028	11863	11494	11087	12031	11224	10004	10075	11733	11005	9663	12364	7529	7211	6745	Cytidine monophosphate kinase
COG1102	857	642	201	7576	7202	8076	3507	6967	5462	3736	7305	3857	7330	6901	6722	708	721	487	Cytidine monophosphate kinase
COG1849	0	0	0	1	0	2	0	0	0	0	0	0	1	2	0	0	0	0	Cytidyltransferase-related domain protein
COG0615	210	144	45	5410	4774	5220	2022	4737	3579	2539	5740	2179	4980	4854	4268	166	133	99	cytidylyltransferase
COG1083	2550	3339	2552	672	576	677	697	810	721	1239	716	920	571	758	614	3268	2936	3395	cytidylyltransferase
COG4750	2	1	0	1	3	4	3	1	1	10	2	3	2	1	1	4	1	0	cytidylyltransferase choline kinase
COG3005	1339	1274	1643	2032	1981	2263	2298	2470	2506	2332	2387	3073	2085	2312	2348	1352	1149	1316	Cytochrome
COG3245	1030	1604	2972	670	428	449	1770	997	1078	1713	1603	1400	453	560	575	3895	3126	2980	Cytochrome
COG3658	739	539	188	830	594	654	1810	1225	1656	1562	1632	1593	632	849	817	681	388	459	Cytochrome
COG4117	4305	4816	2079	6947	8669	7996	7940	8804	7596	6407	7040	7768	8023	6454	8776	3751	3501	3439	Cytochrome
COG3783	203	366	1420	4	4	2	2	7	2	14	4	2	1	12	1	217	225	293	Cytochrome b(562)
COG4892	775	265	37	86	35	25	603	181	159	650	294	331	72	117	60	297	495	329	Cytochrome b5
COG5274	0	1	0	0	0	0	0	0	0	1	0	0	0	0	0	0	2	0	cytochrome b5
COG3038	3469	5687	13424	1765	1282	1264	4336	2789	3256	3919	3781	3770	1176	1461	1574	9163	7417	7691	cytochrome b561
COG0755	8816	8429	6062	11035	12254	11872	11997	12548	12044	11274	11172	12748	12087	11062	12781	8181	8072	7657	cytochrome c
COG2010	26638	28353	35807	42288	50265	47306	46074	51492	47560	40157	42349	48217	46450	40153	51850	31262	30501	25677	cytochrome c
COG3258	3221	3065	2161	5615	7423	6296	7294	7632	7085	6387	6809	7253	6551	5201	7648	4507	3946	3378	cytochrome c
COG4137	3850	3540	3580	7574	9264	8855	8113	9688	8743	7097	8015	8450	8482	7130	9731	4612	4316	3514	cytochrome C assembly protein
COG0785	9902	9697	6828	14097	14474	14348	11068	14274	12516	10490	13525	11052	14172	12990	14056	8151	8028	8592	Cytochrome c biogenesis protein
COG1333	3647	3522	1706	2313	1466	1615	3077	1741	2508	2407	2152	2648	2106	2479	1646	2357	2333	2869	Cytochrome c biogenesis protein
COG2993	2538	3244	3777	4526	4701	5386	4803	5069	5026	5157	5101	5430	4739	4829	5495	5291	4505	4166	Cytochrome C oxidase
COG4736	990	1853	3374	281	296	234	872	509	810	903	717	790	216	250	302	3633	3020	2841	cytochrome C oxidase
COG3175	1041	1426	1857	935	731	726	1758	1350	1298	1975	1792	1944	641	907	850	2639	2071	1980	Cytochrome C oxidase assembly protein
COG0843	15864	15473	10198	26496	28569	28322	25014	29583	27721	22068	26539	25824	27746	25361	28913	15024	14834	13372	Cytochrome C oxidase subunit I
COG5473	552	862	527	612	675	619	873	752	736	1369	1134	1222	535	660	761	937	544	551	Cytochrome C oxidase subunit I
COG5605	318	816	1554	180	158	110	743	298	292	703	452	589	187	315	208	1825	1564	1399	Cytochrome C oxidase subunit IV
COG1858	6668	6851	4082	14430	16976	17145	16410	17282	15266	14587	15013	17251	16354	14489	18064	8326	8060	6002	cytochrome C peroxidase
COG2857	1560	1651	1923	1310	992	1012	2744	2082	2441	2334	2325	2780	893	1154	1135	2765	2155	2077	cytochrome c1
COG1138	4143	4076	4008	7499	9330	8618	8571	9838	8927	7533	8110	9106	8409	7147	9516	4951	4535	3616	cytochrome c-type biogenesis protein
COG4235	11285	10352	5669	26721	33452	30761	25981	32768	28557	22656	25229	27441	30735	24583	33520	10777	10862	7617	cytochrome c-type biogenesis protein
COG3105	387	923	2771	7	4	4	12	13	11	150	52	44	4	14	5	1798	1516	1572	Cytochrome d ubiquinol oxidase subunit III
COG3125	2465	2218	3347	564	474	393	1985	1067	1726	1569	1254	1470	415	470	537	3009	2819	2818	cytochrome o ubiquinol oxidase (subunit iv)
COG3197	1378	2121	2425	1174	1154	1044	2533	1634	2013	2337	2125	2398	1191	1249	1232	2911	2464	2106	cytochrome oxidase maturation protein cbb3-type
COG2124	18534	16338	10542	31212	32255	31968	28348	28779	30539	26356	25908	31503	32522	32804	33175	16704	14772	14505	Cytochrome p450
COG1346	2223	2721	5877	695	622	645	1359	844	858	1896	1412	1222	565	702	721	4719	4175	4395	cytolysis
COG4111	632	859	600	361	457	339	787	582	925	639	611	867	290	366	443	473	585	306	cytoplasmic membrane protein
COG2879	2468	3599	4173	468	292	339	943	682	671	929	809	1045	266	464	326	3478	2900	3616	cytoplasmic protein
COG2960	1550	1897	3251	1130	894	886	2228	1628	1924	1755	1743	2073	781	991	1001	2772	2237	2246	cytoplasmic protein
COG3139	387	920	2797	6	5	2	7	9	7	147	43	36	1	13	1	1795	1536	1572	cytoplasmic protein
COG3151	487	965	2799	103	38	36	187	148	232	241	147	272	36	57	47	1832	1530	1592	cytoplasmic protein
COG3530	1219	1804	3263	836	730	714	1940	1059	1443	1443	1464	1605	896	694	808	2677	2419	2062	cytoplasmic protein
COG4460	441	970	2789	37	23	31	260	122	175	228	187	126	35	39	48	1828	1543	1596	cytoplasmic protein
COG5426	1667	1945	2171	2549	3118	3313	2144	2477	2449	2249	2176	2893	3162	2391	3416	1743	1667	1687	cytoplasmic protein
COG5475	244	407	1409	19	7	6	237	100	148	57	118	62	9	9	7	246	226	305	cytoplasmic protein
COG4352	0	0	0	0	0	0	0	0	0	0	0	0	0	0	0	0	0	0	cytoplasmic translation
COG0270	8618	9564	6898	14070	11338	11088	13408	12098	14391	10742	11299	13606	13551	14920	12020	6939	6819	6904	Cytosine-specific methyltransferase
COG1426	8244	8206	7454	10931	11160	10915	11343	11616	11560	10116	10243	11664	11147	10930	11774	7281	7462	7090	Cytoskeletal protein that is involved in cell-shape control through regulation of the length of the long axis (By similarity)
COG4283	964	407	379	261	318	331	215	224	266	491	238	292	317	314	330	371	720	546	Cytosolic protein
COG4913	1956	2574	1367	455	432	446	724	488	629	485	511	699	392	301	502	1572	1324	1970	Cytosolic protein
COG1461	2808	2864	1344	1026	1236	1308	666	732	884	967	729	849	1182	1095	1181	1803	1840	2517	dak2 domain fusion protein ylov
COG2027	5960	6752	6803	10582	10123	9740	11380	10712	10857	9265	9604	11252	10846	10890	10641	7658	7345	6692	D-alanyl-D-alanine carboxypeptidase
COG3966	563	394	175	536	637	653	750	569	960	375	448	777	569	295	765	411	540	528	D-alanyl-lipoteichoic acid biosynthesis protein DltD
COG2362	1562	1090	1624	1186	1351	1437	1256	1079	1133	1276	1256	1161	1193	1131	1291	903	996	1037	d-aminopeptidase
COG3266	413	539	1446	284	306	349	302	356	392	317	313	379	290	272	398	364	325	427	DamX-related protein
COG5067	192	165	117	4	2	0	31	6	10	6	4	22	0	3	0	47	46	60	DBF4 homolog (S. cerevisiae)
COG2131	1395	1161	504	2005	1337	1569	1928	1419	1702	2327	1802	2163	1963	2456	1717	1190	1331	1048	dCMP deaminase activity
COG3233	684	1000	1573	682	691	746	725	580	624	822	671	774	598	525	751	2123	1841	1730	deacetylase
COG3653	5238	4917	3998	10628	12893	12695	10485	12583	11800	9349	10162	11121	11779	9908	12954	5818	5228	4390	Deacylase
COG1205	3608	3519	1575	9109	7377	8076	4845	6958	6607	5343	7983	5597	8506	9079	7201	2491	2578	3064	DEAD DEAH box helicase
COG1202	127	83	22	5114	4490	4898	1843	4546	3359	2206	5449	1888	4701	4425	3930	104	79	42	Dead deah box helicase domain protein
COG0402	15234	17317	24015	30575	29860	30787	24401	30548	26843	23346	32910	23993	29849	28304	28926	20161	18011	17754	deaminase
COG0590	17296	19161	13394	19747	22068	21158	21084	22546	21608	18828	19264	22220	21388	19397	22714	16723	15353	15912	deaminase
COG3654	3586	2994	1146	8177	9642	8992	7144	8954	7971	6379	7184	7703	9242	7872	9534	2964	2970	2056	Death-On-Curing Family
COG0076	10061	11200	7640	14741	18809	17430	14085	17541	15432	12645	13954	15013	16952	13954	18833	8389	7977	7721	decarboxylase
COG1611	12911	13887	9750	25623	27372	26960	24721	27981	25811	21219	25732	24903	26828	24204	27715	14036	13675	12046	decarboxylase
COG1982	4625	4648	5626	1803	829	990	2751	1370	2163	2521	1948	2229	1363	1872	1081	4490	4249	5069	decarboxylase
COG1883	254	263	57	962	1016	1211	751	958	886	1069	930	1059	1185	1287	1214	249	213	212	decarboxylase beta subunit
COG5551	190	110	43	5986	4514	4962	2664	4707	3873	2577	5691	2565	5180	5375	4015	131	158	67	defense response to virus
COG0399	19943	22261	17670	43775	49182	48130	40097	48521	43767	36905	42464	42593	47389	42681	50243	23524	23003	18739	DegT DnrJ EryC1 StrS aminotransferase
COG1307	5562	3907	1640	1753	1986	2196	1190	1272	1569	2464	1323	1618	1829	1778	2040	2813	3435	4095	degv family
COG2721	2734	3140	4876	3796	4178	4403	5534	4584	4621	4217	4547	4897	3920	3880	4383	4158	3659	3361	Dehydratase
COG4706	581	840	1500	603	442	521	1000	647	732	1038	1154	933	432	511	525	2021	1575	1501	Dehydratase
COG0111	24368	28125	31141	30344	33717	33785	29462	33662	31807	27856	31664	30978	32403	30572	34472	28332	26243	25636	Dehydrogenase
COG0300	36138	38409	39367	54513	57661	56966	59581	59397	57607	51625	55589	59344	56761	54688	60007	41434	39725	35126	Dehydrogenase
COG0371	1347	1826	5701	6171	4789	5226	2842	4864	4077	2817	5872	2820	5360	5587	4228	1262	1220	1460	Dehydrogenase
COG0508	24420	26475	19706	31059	31582	32966	27343	32236	30188	27042	32363	28744	31347	29787	31861	24540	22782	23995	Dehydrogenase
COG0677	6000	4887	3336	14689	16045	15948	12044	16023	14399	11430	14958	13016	15433	13704	15687	4810	4797	3576	Dehydrogenase
COG1012	91046	109946	103005	97128	105477	104654	100628	109294	104819	91554	104419	98442	102057	92498	106978	107316	98436	104334	Dehydrogenase
COG1052	11692	13579	21587	19112	19896	19997	19405	20766	19518	17889	20784	19548	19475	18100	20242	17848	16677	14774	Dehydrogenase
COG1063	41492	46850	34604	65865	73942	73933	56264	71735	63653	51402	64548	58642	71609	63448	74321	37485	35705	37360	Dehydrogenase
COG1071	15674	17597	10402	26200	26765	27964	19308	26100	23298	19508	25640	20800	26639	24670	26634	15259	14008	15467	Dehydrogenase
COG1250	22591	23085	18729	36252	40472	39525	34122	41371	37934	31805	39269	34336	38213	33630	40267	24869	23430	21617	Dehydrogenase
COG1319	8714	8788	4854	33431	33752	35094	20034	32790	27086	20369	34337	21064	33446	30559	31753	9251	7827	7413	Dehydrogenase
COG2084	23061	28487	24739	23328	23928	24343	24651	25392	26028	20691	25169	24142	23349	22446	24336	23631	20916	23977	Dehydrogenase
COG2133	16819	16986	10223	34087	37669	39419	31305	35759	33701	32112	33640	37123	36776	34275	40961	18937	18474	16133	Dehydrogenase
COG2902	2619	3631	3040	1213	1164	1164	1898	1523	1759	1867	1650	1922	1072	1130	1294	3621	2979	3634	Dehydrogenase
COG4993	11897	13267	17206	34704	37698	39280	27127	38242	32229	24385	25783	28609	36107	32055	37862	15444	13840	11973	Dehydrogenase
COG1028	186950	201886	176936	241949	276276	270243	261050	279862	269986	232748	249851	266563	262240	236464	285164	194098	179667	174537	Dehydrogenase reductase
COG0113	8078	8495	7337	10935	11850	11492	12198	12276	11781	10752	10827	12542	11477	10624	12404	9312	8704	8087	delta-aminolevulinic acid dehydratase
COG3256	2325	1984	1986	2601	2524	2951	2576	2978	3043	2506	2602	2903	2473	2504	2599	2879	2860	2467	denitrification pathway
COG0717	7559	7427	5582	15294	15776	15711	12910	16058	14462	11828	15579	13380	15399	14177	15652	7361	6848	6341	Deoxycytidine triphosphate deaminase
COG3492	648	1180	1741	592	600	618	730	729	697	1091	752	1010	558	569	718	2212	1746	1737	Deoxycytidine triphosphate deaminase
COG0232	7011	7722	5956	9723	11645	11300	10977	11794	10921	9575	10311	11330	10676	9350	12223	7446	6896	6390	deoxyguanosinetriphosphate triphosphohydrolase-like protein
COG1899	3167	2881	1058	17716	18250	18538	11510	18247	14656	11244	18056	11910	18030	15974	17533	2924	3106	1958	deoxyhypusine synthase
COG1428	4295	3170	1011	6684	8583	7791	7205	8541	7304	6767	6921	7429	7596	6505	8526	3066	3685	2543	Deoxynucleoside kinase
COG0415	7240	7872	5824	16096	15706	15721	14137	16287	14617	12845	16013	14074	16020	15355	15917	7872	7261	6342	deoxyribo-dipyrimidine photolyase
COG3046	648	858	633	731	513	550	1361	758	770	1264	890	1331	537	748	774	718	667	421	Deoxyribodipyrimidine photolyase-related protein
COG3269	0	0	0	0	0	0	0	0	0	0	0	0	0	0	0	0	0	0	deoxyribonuclease rho motif-related tram
COG2320	2969	1815	878	1848	2196	2306	1559	1700	1787	2039	1581	1902	2048	1907	2366	1505	1993	1767	dephospho-CoA kinase protein folding accessory domain-containing protein
COG3509	2832	2384	1183	3789	4176	4283	5397	4080	4609	4700	4757	5115	3728	4034	4509	2536	2050	1885	depolymerase
COG5291	0	0	0	1	0	0	0	1	0	0	0	0	0	0	0	0	0	0	Derlin 1
COG1398	5177	4571	2818	13246	13415	13388	14141	13817	13340	11213	11781	14269	13516	12969	14051	5861	5590	4471	desaturase
COG3349	2884	2291	744	9131	8539	8028	8629	8618	8462	6827	7232	8693	9135	9121	8968	2469	2495	1684	Desaturase
COG0605	9820	9805	7944	17328	17684	17829	15992	18386	17064	15098	17967	16674	17471	16580	18028	10620	10191	9125	Destroys radicals which are normally produced within the cells and which are toxic to biological systems (By similarity)
COG2032	2347	2030	2206	2521	2591	3060	2908	3040	3103	2543	2611	2789	2578	2460	2877	1688	1872	1555	Destroys radicals which are normally produced within the cells and which are toxic to biological systems (By similarity)
COG4273	130	74	25	143	112	128	203	169	254	118	121	234	83	106	97	46	48	48	DGC domain protein
COG4240	250	166	84	1079	141	189	1189	436	872	733	479	1015	572	1084	246	153	178	144	D-glycerate 3-kinase
COG0279	7263	7929	6807	12294	14902	14926	11972	14267	12824	10660	11878	12867	14067	12094	15373	7740	7118	6642	D-glycero-D-manno-heptose 7-phosphate metabolic process
COG0818	2712	2749	3734	2408	1536	1554	4098	2165	2987	3353	2818	3592	2071	2561	1810	3659	3508	3126	Diacylglycerol kinase
COG1597	14193	15663	9663	12896	13885	13585	13034	13152	12905	13053	12070	14029	13867	13606	14559	12353	11925	13512	diacylglycerol kinase
COG0412	17892	18217	14463	32446	36917	35612	34657	36628	35370	29930	31794	35875	35062	31691	38548	20233	19631	16440	dienelactone hydrolase
COG2199	39264	39399	44002	65899	76686	72577	66100	75571	68346	63012	67166	69261	72141	61628	78136	48773	46034	39861	Diguanylate cyclase
COG2200	20448	22005	33921	25564	27370	25704	28935	29688	28034	26569	26582	28986	26508	24295	28500	29054	27266	25699	Diguanylate cyclase phosphodiesterase
COG5001	29083	32817	39131	34981	32751	31092	45652	38103	37150	40942	41822	41808	33044	32970	34435	44826	39699	36063	Diguanylate cyclase phosphodiesterase
COG3434	3276	2461	832	7137	8778	8258	7414	8825	7574	6518	7548	7444	7884	6528	8878	2813	2749	1885	Diguanylate phosphodiesterase
COG3804	377	371	159	672	821	981	446	512	572	548	513	644	802	795	854	377	294	440	dihydrodipicolinate reductase
COG0262	25931	30423	18432	23536	28532	27487	25041	26619	25831	23464	24033	27306	26336	24344	29517	23457	22142	24868	dihydrofolate reductase activity
COG1249	33433	33775	24992	47426	51553	50670	47590	52967	50804	42520	47171	50036	49970	45988	53032	31317	30431	28785	dihydrolipoyl dehydrogenase
COG1539	6631	7069	6907	4680	3871	4084	6601	4598	5300	6189	5237	6379	4235	4925	4305	7855	7211	7367	dihydroneopterin aldolase
COG0044	15968	16510	13697	30060	31338	31243	24907	31320	27489	23855	30151	25571	30850	28491	31030	15509	15585	13921	Dihydroorotase
COG0418	1295	1572	3120	957	692	767	1955	1337	1649	1456	1667	1694	642	867	788	2456	2013	1944	Dihydroorotase
COG3964	1091	1251	1751	2138	2379	2759	1556	2251	1948	1519	1719	1859	2327	2248	2520	1069	956	872	dihydro-orotase (EC 3.5.2.3)
COG0294	8831	7981	4802	12441	12786	12588	12978	12906	12859	11603	11466	13591	12808	12245	13507	6744	6889	6283	dihydropteroate synthase
COG2376	7391	10982	11207	3138	2678	2787	2918	2338	2805	2526	2460	3060	3127	3656	2780	7265	6869	8427	Dihydroxyacetone kinase
COG3412	1667	2534	1458	97	135	103	213	130	189	166	119	221	92	106	116	1338	1060	1755	Dihydroxyacetone kinase
COG0129	14230	15575	11941	22217	23020	23555	22580	24215	23290	20280	23911	22554	22516	21268	23717	16662	14665	14148	Dihydroxy-acid dehydratase
COG2846	1281	1648	1838	2452	2693	3005	2613	2782	2760	2368	2357	2789	2752	2606	2824	2427	2357	1960	Di-iron-containing protein involved in the repair of iron-sulfur clusters damaged by oxidative and nitrosative stress conditions (By similarity)
COG1867	158	98	36	5770	4492	4917	2114	4592	3748	2379	5606	2191	5146	5254	3953	108	138	46	Dimethylates a single guanine residue at position 26 of a number of tRNAs using S-adenosyl-L-methionine as donor of the methyl groups (By similarity)
COG2318	12700	9924	6177	22018	28417	26458	21920	27315	23932	20054	21505	23110	25595	21281	28650	10872	11872	8008	dinb family
COG1433	476	495	160	2041	1287	1601	1359	1311	1560	1492	1349	1742	1787	2172	1571	608	537	449	Dinitrogenase iron-molybdenum cofactor biosynthesis protein
COG2175	5353	8046	13013	2810	2100	2197	4589	3089	4434	4319	3755	4317	2340	2889	2302	11734	9633	10482	dioxygenase
COG3670	682	520	255	2902	647	740	2849	1040	2166	1780	1422	2434	1869	3285	849	628	636	479	dioxygenase
COG2355	8828	8058	7887	20379	23978	23105	17123	23248	20136	16353	20350	18157	22341	18803	23620	9559	9445	7659	Dipeptidase
COG4690	76	62	26	68	64	56	248	142	118	121	115	170	63	74	71	55	42	133	dipeptidase activity
COG3407	374	494	356	5340	4731	5081	2159	4854	3732	2437	5673	2290	4871	4649	4171	379	423	118	Diphosphomevalonate decarboxylase
COG1736	123	73	20	5112	4486	4890	1841	4544	3356	2200	5446	1879	4697	4422	3922	93	77	37	diphthamide biosynthesis protein
COG1660	6587	6438	5356	7720	9428	8938	8218	9683	8890	7524	8054	8625	8617	7340	9658	6200	6036	5873	Displays ATPase and GTPase activities (By similarity)
COG3302	128	143	54	232	137	180	678	400	377	481	577	459	184	313	247	169	96	92	dmso reductase anchor subunit
COG2852	4604	6217	4822	8530	6397	6926	5189	6691	6525	5109	7967	5429	7439	7991	6173	3971	3472	4826	DNA (cytosine-5-)-methyltransferase
COG4335	701	1278	1919	411	457	459	983	549	558	840	889	923	458	535	492	2070	2040	1553	DNA alkylation repair
COG4912	6195	6732	3291	6622	8508	7850	6646	8125	6951	6086	6605	6651	7749	6338	8514	4652	4734	5059	DNA alkylation repair enzyme
COG4699	18	26	24	1	1	1	2	1	3	2	1	4	2	1	3	11	71	129	DNA binding protein
COG4228	194	566	1380	56	17	25	78	49	32	201	169	73	23	48	26	1585	1320	1287	DNA circulation
COG5087	0	0	0	0	0	0	0	0	0	0	0	0	0	0	0	0	0	0	DNA damage response protein
COG1636	894	923	1563	1116	1107	1205	1082	1140	1343	855	966	1402	1126	1111	1047	702	688	696	DNA integration recombination invertion protein
COG0658	3002	3976	2014	4723	4269	4860	4520	4277	4203	3875	3892	4757	4486	5099	4632	2887	2690	2706	DNA internalization-related competence protein ComEC Rec2
COG2333	10221	7879	6580	17888	21292	20761	16104	20408	18002	15506	16559	17398	20056	16960	21771	8734	9231	7383	DNA internalization-related competence protein ComEC Rec2
COG1793	16733	21395	11323	28169	31358	31457	21649	30651	25964	21113	28966	22977	29860	26297	31181	16178	13600	16350	DNA ligase
COG3285	9904	11638	6654	10231	12531	12293	10587	12354	11259	9793	10335	11167	11538	10198	13122	9315	8879	9744	DNA ligase
COG0272	8834	9492	10277	11183	12245	11907	12195	12583	12113	11070	11203	12699	11920	10949	12913	10103	9491	9087	DNA ligase (NAD+) activity
COG0827	793	243	58	18	16	17	25	13	30	336	26	31	10	14	19	222	466	467	DNA Methylase
COG1743	2073	2661	1232	6270	5585	6115	3410	5624	4818	3381	6466	3493	5988	5664	5293	1615	1341	2119	DNA methylase
COG2189	1468	1250	400	2367	2377	2505	2803	2462	2884	2447	2358	3151	2476	2369	2603	1329	1137	1023	DNA methylase
COG0863	7776	9237	7028	19880	17008	18658	13130	17162	15704	12635	19099	13709	18717	18854	16797	6991	6136	7324	DNA methylase n-4 n-6 domain protein
COG3727	4636	5165	3768	6878	8935	8210	6399	8308	7190	5862	6505	6822	8098	6395	8810	3802	3756	3655	DNA mismatch endonuclease (vsr)
COG4220	51	40	11	57	59	88	81	98	118	76	82	98	65	75	127	34	37	41	DNA packaging protein
COG5565	49	90	43	40	22	36	60	47	30	119	44	48	23	21	24	71	60	58	DNA packaging protein
COG0417	1249	1796	3032	11324	10202	10961	5396	10295	7808	6024	12376	5357	10601	10261	9056	2716	2348	2029	DNA polymerase
COG0749	9713	10435	7298	10847	12011	11594	11836	12300	11911	10651	10865	12429	11538	10754	12453	8953	8385	8555	DNA polymerase
COG1796	3853	2817	822	8036	10347	9855	7188	9466	8316	6875	7411	7927	9589	7811	10577	2824	3156	2403	DNA polymerase
COG5214	0	0	0	0	0	0	0	0	0	0	0	0	0	0	0	0	0	0	DNA polymerase alpha
COG1665	123	75	20	5116	4491	4890	1845	4546	3361	2204	5449	1880	4699	4424	3924	93	78	39	DNA polymerase beta domain protein region
COG1669	4339	3721	1182	18376	15905	16137	13830	16047	15034	12029	15509	14317	17961	17964	15839	3805	3980	2754	DNA polymerase beta domain protein region
COG1708	1525	1303	1674	10584	9233	10059	4375	9557	7342	5273	11381	4344	9636	9144	8240	2247	2041	1802	DNA polymerase beta domain protein region
COG2413	123	73	20	5111	4486	4888	1841	4543	3356	2200	5446	1879	4696	4420	3922	93	77	37	DNA polymerase beta domain protein region
COG0847	7956	11540	13636	5121	4813	5042	8903	6408	6815	7583	7061	8665	4840	5567	5594	10075	8870	9539	DNA polymerase iii
COG0587	14122	15457	11619	20634	23173	22323	22543	23505	22301	19974	20712	23414	22293	20376	24383	14871	14323	12650	DNA polymerase III (alpha subunit)
COG2927	1589	1940	3303	1168	858	896	2520	1888	2202	2172	2204	2557	809	1065	1040	2911	2332	2295	dna polymerase iii (Chi subunit)
COG1466	7788	7916	5933	11479	11954	11636	11815	12234	12098	10577	10800	12523	11990	11446	12541	7636	7356	6771	DNA polymerase III (delta' subunit)
COG0470	141	94	46	165	113	142	121	232	150	115	134	300	93	228	99	106	127	85	DNA polymerase III subunit delta'
COG3050	203	370	1395	0	1	0	2	3	1	9	3	3	0	5	0	223	205	292	DNA polymerase III subunit psi
COG2812	16226	15980	11849	27221	28285	27997	24978	28561	26831	23229	26640	26084	28137	26251	28876	15418	14951	13776	dna polymerase iii subunits gamma and tau
COG0592	8361	8401	6106	17253	17293	17392	14118	17319	16046	13257	16712	15101	17575	16432	17339	8140	7831	7216	DNA polymerase processivity factor activity
COG4643	418	284	113	565	408	513	885	828	765	778	805	1068	424	534	513	371	271	267	DNA primase
COG0358	8892	8832	6531	17297	18063	18016	16048	18381	16863	14933	18173	16749	17734	16997	18298	8887	8522	7501	DNA primase activity
COG1467	123	73	20	5112	4486	4890	1841	4544	3356	2200	5446	1879	4697	4422	3922	93	77	37	DNA primase is the polymerase that synthesizes small RNA primers for the Okazaki fragments on both template strands at replication forks during chromosomal DNA synthesis (By similarity)
COG2219	246	146	40	10223	8972	9778	3682	9086	6712	4400	10892	3758	9393	8842	7843	186	154	74	DNA primase is the polymerase that synthesizes small RNA primers for the Okazaki fragments on both template strands at replication forks during chromosomal DNA synthesis (By similarity)
COG0758	11018	12215	7970	11111	12238	11926	11819	12394	12012	10961	10906	12501	11779	10898	12785	9839	9241	10199	DNA protecting protein DprA
COG4712	20	14	7	34	58	64	17	34	26	34	20	28	53	47	62	20	18	21	DNA recombination
COG1322	6323	7197	5673	9047	10988	10432	10081	11156	10413	9076	9661	10514	10151	8617	11425	6950	6275	5891	Dna recombination protein
COG5055	0	2	0	0	0	0	0	0	0	0	0	0	0	0	0	2	0	2	DnA repair
COG2003	5121	5613	7359	5956	5098	5480	8448	6172	7419	7614	6865	8663	5499	6619	5927	5921	5776	5180	DNA repair protein (RadC
COG1599	123	73	20	5113	4487	4892	1841	4544	3356	2201	5447	1879	4698	4424	3922	93	77	37	DNA replication
COG5138	0	0	0	0	0	0	0	0	0	0	0	0	0	0	0	0	0	0	DNA replication complex GINS protein
COG5093	0	0	0	0	0	0	0	0	0	0	0	0	0	0	0	0	0	0	DNA replication complex GINS protein psf2
COG1241	125	75	22	5115	4487	4893	1847	4547	3361	2203	5452	1886	4699	4423	3923	94	82	40	dna replication licensing factor
COG3935	947	358	106	51	52	53	51	38	56	395	54	76	40	48	53	362	651	709	DNA replication protein DnaD
COG1864	2348	3612	2828	1653	1076	1134	2272	1380	1631	1994	1915	2023	1407	1952	1185	3559	3038	3284	DNA RNA NON-specific endonuclease
COG3569	2585	3627	2016	6378	5764	6293	4231	6241	5180	4128	7199	4135	5964	5922	5626	2425	2203	2562	Dna topoisomerase
COG0551	2587	2388	1964	6368	8358	7692	5775	7913	6686	5175	5966	6224	7609	5838	8288	2245	2284	1666	DNA topoisomerase type I activity
COG1389	154	100	27	5140	4523	4926	1866	4572	3399	2250	5490	1935	4738	4465	3968	124	93	142	DNA topoisomerase VI subunit B
COG2842	3669	3966	1628	6110	8022	7359	5667	7665	6517	4887	5805	5939	7237	5571	8087	2992	2949	2839	DNA transposition protein
COG1833	124	172	52	287	385	380	185	238	236	242	189	308	483	317	321	132	137	153	DNA-(Apurinic or apyrimidinic site) lyase (EC 4.2.99.18)
COG2818	4453	6184	7039	2737	2744	2858	4335	3279	3746	3963	3675	4210	2599	2835	3151	6770	5957	6222	DNA-3-methyladenine glycosylase activity
COG1661	1099	1149	722	3162	3481	4098	2478	3322	2964	2501	2904	3034	3515	3336	3754	1117	1184	860	DNA-binding protein
COG2118	123	73	20	5112	4486	4890	1841	4544	3356	2200	5446	1879	4697	4422	3922	93	77	37	DNA-binding protein
COG4465	860	278	62	31	27	32	39	23	43	351	36	45	23	26	27	269	509	515	DNA-binding protein that represses the expression of many genes that are induced as cells make the transition from rapid exponential growth to stationary phase. It is a GTP-binding protein that senses the intracellula [illegible]
COG1644	123	73	20	5112	4486	4890	1841	4544	3356	2200	5446	1879	4697	4422	3922	93	77	37	DNA-dependent RNA polymerase catalyzes the transcription of DNA into RNA using the four ribonucleoside triphosphates as substrates
COG1761	123	73	20	5112	4486	4890	1841	4544	3356	2200	5446	1879	4697	4422	3921	93	77	37	DNA-dependent RNA polymerase catalyzes the transcription of DNA into RNA using the four ribonucleoside triphosphates as substrates
COG1996	0	0	0	1	2	1	0	0	0	0	1	0	0	2	4	0	0	0	DNA-dependent RNA polymerase catalyzes the transcription of DNA into RNA using the four ribonucleoside triphosphates as substrates
COG2012	123	73	20	5112	4486	4890	1841	4544	3356	2200	5446	1879	4697	4422	3922	93	77	37	DNA-dependent RNA polymerase catalyzes the transcription of DNA into RNA using the four ribonucleoside triphosphates as substrates
COG0085	7878	7983	5925	15997	16536	16585	13393	16789	15119	12636	16130	14147	16293	15098	16492	7728	7388	6811	DNA-dependent RNA polymerase catalyzes the transcription of DNA into RNA using the four ribonucleoside triphosphates as substrates (By similarity)
COG0086	7991	8040	5967	17040	16692	16838	14322	17023	15734	13116	16490	14931	16911	16207	16756	7782	7470	6849	DNA-dependent RNA polymerase catalyzes the transcription of DNA into RNA using the four ribonucleoside triphosphates as substrates (By similarity)
COG0202	8160	8254	6047	16433	17087	17168	13768	17180	15513	13005	16643	14648	16997	15461	17123	8039	7723	7116	DNA-dependent RNA polymerase catalyzes the transcription of DNA into RNA using the four ribonucleoside triphosphates as substrates (By similarity)
COG1594	123	73	20	5112	4486	4890	1841	4544	3356	2200	5446	1879	4697	4422	3922	93	77	37	DNA-dependent RNA polymerase catalyzes the transcription of DNA into RNA using the four ribonucleoside triphosphates as substrates (By similarity)
COG5108	0	0	0	0	0	0	0	0	0	1	0	0	0	0	0	0	0	0	DNA-dependent RNA polymerase catalyzes the transcription of DNA into RNA using the four ribonucleoside triphosphates as substrates (By similarity)
COG0613	5328	5848	4963	7497	7128	8099	7281	6660	7076	5883	6561	7476	7607	7798	7703	5684	5076	5698	DNA-directed DNA polymerase activity
COG1311	126	74	20	5114	4489	4891	1844	4545	3356	2204	5448	1888	4698	4428	3922	96	80	47	DNA-directed DNA polymerase activity
COG1095	123	73	20	5112	4486	4890	1841	4544	3356	2200	5446	1879	4697	4422	3922	93	77	37	DNA-directed RNA Polymerase
COG5111	0	0	0	0	0	0	0	0	0	0	0	0	0	0	0	0	1	2	dna-directed rna polymerase iii subunit
COG2214	0	0	0	2	0	0	0	0	0	0	1	2	0	2	0	1	2	0	DNAj domain protein
COG1928	2367	2908	1559	6670	5323	5760	3291	5254	4608	3143	6263	3292	5950	6162	4826	1755	1520	2107	dolichyl-phosphate-mannose-protein mannosyltransferase activity
COG5646	2303	2901	1340	2327	2545	2964	2464	2704	2358	2049	2252	2440	2414	2503	2765	1850	1738	2130	Domain of unknown function (DU1801)
COG5649	3409	3219	2426	6487	8429	7722	6917	8402	7667	5810	6585	6956	7564	5977	8428	4041	3778	2956	Domain of unknown function (DU1801)
COG5457	1368	3248	6534	473	546	474	602	554	488	1131	659	844	441	478	546	5770	4668	4578	Domain of unknown function (DUF1127)
COG5467	423	586	334	525	531	487	634	587	570	826	617	777	418	480	576	574	395	438	Domain of unknown function (DUF1476)
COG3930	418	718	1450	196	145	144	280	231	353	389	249	411	163	210	202	1732	1447	1477	Domain of unknown function (DUF1704)
COG5515	1440	2360	1271	64	56	60	156	99	113	160	103	135	50	57	68	1237	1005	1685	Domain of unknown function (DUF1737)
COG5514	191	154	90	79	48	45	260	121	88	265	225	123	46	55	37	206	182	126	Domain of unknown function (DUF1794)
COG5435	248	396	1395	43	42	36	247	120	179	78	150	94	42	48	45	248	216	394	Domain of unknown function (DUF1795)
COG4293	353	254	104	1018	439	525	560	361	719	447	427	682	852	1092	546	299	393	303	Domain of unknown function (DUF1802)
COG3377	945	332	70	72	60	74	63	52	61	356	77	62	57	62	89	326	564	553	Domain of unknown function (DUF1805)
COG5474	60	34	20	859	12	59	813	155	506	356	238	666	472	938	75	27	74	22	Domain of unknown function (DUF1817)
COG4517	230	604	1414	106	87	72	92	83	94	231	127	152	73	87	109	1639	1354	1308	Domain of unknown function (DUF1820)
COG5003	2	3	0	50	6	16	47	23	15	37	111	14	12	35	12	2	2	2	Domain of unknown function (DUF1834)
COG4729	823	314	74	101	127	97	98	98	108	437	91	155	98	114	122	378	453	405	Domain of unknown function (DUF1850)
COG3782	412	710	1486	108	54	43	785	331	295	504	504	343	65	88	59	1938	1502	1391	Domain of unknown function (DUF1853)
COG5255	101	209	187	25	30	24	103	55	56	129	60	97	21	33	43	114	96	68	Domain of unknown function (DUF1868)
COG3955	2	1	0	0	0	1	0	0	0	5	1	4	0	1	0	6	0	1	Domain of unknown function (DUF1919)
COG3878	544	359	150	1280	743	780	736	546	952	646	678	895	1172	1548	768	301	441	427	Domain of unknown function (DUF1963)
COG4762	1946	2617	1488	47	50	66	178	82	151	83	84	150	48	67	57	1295	1100	1790	Domain of unknown function (DUF1990)
COG3812	1437	1602	1937	1058	723	651	2901	1434	1538	2199	2087	2130	648	860	815	2893	2961	2261	Domain of unknown function (DUF1993)
COG3875	3231	2711	870	7992	10290	9888	7017	9360	8227	6457	7149	7897	9555	7351	10684	2857	2987	2314	Domain of unknown function (DUF2088)
COG1547	1504	755	202	2752	2118	2542	2066	2095	2112	1703	1636	2047	2467	2778	2181	767	1078	858	Domain of unknown function (DUF309)
COG2014	74	82	31	225	219	282	161	199	189	221	174	210	204	207	218	75	90	91	Domain of unknown function (DUF364)
COG2028	0	0	0	0	0	0	0	0	0	0	0	0	0	0	0	0	0	0	Domain of unknown function (DUF365)
COG2029	0	0	0	0	0	0	0	0	0	0	0	0	0	0	0	0	0	0	Domain of unknown function (DUF366)
COG2090	6	2	0	7	12	9	6	12	18	5	8	11	5	14	9	4	4	1	Domain of unknown function (DUF371)
COG2237	168	106	28	5160	4538	4968	1909	4630	3467	2252	5507	1962	4757	4490	4035	116	102	73	Domain of unknown function (DUF373)
COG2121	2932	2486	740	7616	9666	9205	6975	9225	7926	6598	7210	7770	8890	7174	10029	2601	2607	1770	Domain of unknown function (DUF374)
COG3597	705	559	287	2219	898	991	1713	868	1599	1173	1055	1858	1758	2570	1142	462	611	433	Domain of unknown function (DUF697)
COG4009	0	0	0	0	0	0	0	0	0	0	0	0	0	0	0	0	0	0	Domain of unknown function (DUF749)
COG4707	17	25	24	1	1	1	2	1	2	2	1	3	1	1	2	10	69	126	Domain of unknown function (DUF771)
COG5519	221	94	28	211	127	138	237	341	303	121	173	437	84	228	110	97	106	79	Domain of unknown function (DUF927)
COG1693	80	92	19	264	264	320	217	236	214	210	232	256	236	261	256	94	110	78	Domain of unknown function DUF128
COG1417	0	0	0	0	0	0	0	0	0	0	0	0	0	0	0	0	0	0	Domain of unknown function DUF22
COG0011	3106	3134	1511	1034	1161	1231	915	865	1036	1291	989	1215	1128	1238	1174	2028	2050	2779	Domain of unknown function DUF77
COG0247	15528	13364	8376	32856	37238	36203	33337	37984	34495	29605	34641	33353	35603	31429	37765	16990	16432	12941	domain protein
COG0426	1800	1219	435	5225	2246	2680	4048	2287	3644	3384	2691	4199	4152	5625	2611	1321	1579	1374	domain protein
COG1235	11060	10310	7312	22949	27284	25286	23451	26849	24267	21532	22644	24767	25655	21861	28352	12554	12294	9343	domain protein
COG1388	7307	8725	5153	2328	2276	2474	3196	2640	2982	3447	2695	3345	2216	2493	2596	6489	6017	7816	domain protein
COG1470	827	686	546	1005	1216	1214	1207	953	1028	1009	1016	1281	1261	1504	1361	620	747	536	domain protein
COG1511	6394	4840	3472	829	781	858	1304	890	1504	2202	1064	1337	749	833	856	3234	3807	4622	domain protein
COG1572	2347	1889	526	6520	7847	7138	5614	7457	6605	4992	5724	6072	7448	6246	7785	1904	2036	1491	domain protein
COG1652	4952	7196	5520	3749	3731	4221	4180	4323	4554	4063	4240	4715	3734	3824	4087	7114	5769	6973	domain protein
COG1827	902	331	91	279	332	348	227	231	281	524	248	310	328	325	341	322	558	532	domain protein
COG2000	1	1	0	9	7	7	17	19	23	17	16	16	10	14	10	1	2	2	domain protein
COG2183	6456	6732	5328	8963	10040	9581	9884	10469	9951	8628	8914	10181	9725	8833	10453	6657	6405	5831	domain protein
COG3103	589	651	1485	714	753	696	974	793	648	694	673	998	625	829	667	606	645	590	domain protein
COG3287	543	517	203	980	850	929	1352	964	1002	1462	1334	1214	965	1109	1083	814	472	557	domain protein
COG3320	1789	1446	1728	1633	1072	1149	1548	1022	1456	1810	1425	1491	1684	1904	1167	2551	2459	2398	domain protein
COG3400	2	0	3	0	0	0	4	2	1	2	12	4	0	1	1	3	2	0	domain protein
COG3480	3698	3213	1553	359	417	440	315	242	392	840	262	321	407	413	405	1964	2205	2849	domain protein
COG3550	10794	12240	11733	14254	17709	16507	16810	18541	17393	13814	15887	16045	15892	13152	18049	10274	9429	9097	domain protein
COG3583	2539	2664	1320	104	141	132	184	112	207	444	123	185	94	126	111	1516	1567	2247	domain protein
COG3584	2259	972	267	814	932	1013	704	684	791	1370	719	922	866	786	1066	1056	1519	1400	domain protein
COG3861	3664	4622	2168	1516	787	916	1662	940	1342	1546	1117	1626	1134	1789	972	2614	2324	3813	domain protein
COG3942	3326	4788	2355	1261	1463	1485	1174	1129	1289	1070	1105	1341	1383	1323	1428	2735	2348	3641	domain protein
COG4109	1155	956	1452	226	212	245	206	218	200	627	245	256	192	205	210	1970	1999	1977	domain protein
COG4297	837	1422	4382	168	192	164	197	200	161	307	211	246	188	276	224	948	782	1060	domain protein
COG4932	814	565	305	5597	5066	5501	2418	5033	3922	2783	5975	2513	5327	5091	4618	600	583	636	domain protein
COG5295	119	83	47	53	22	23	492	228	234	167	232	202	34	30	31	60	52	36	domain protein
COG5260	246	146	40	10222	8972	9776	3682	9086	6712	4400	10890	3758	9392	8840	7842	186	154	74	domain) containing
COG3892	776	1329	3029	377	341	355	828	420	461	739	642	481	391	396	392	2161	1821	1819	Domain-Containing protein
COG5038	210	182	124	37	62	73	48	41	37	43	25	55	56	53	67	69	60	78	Domain-Containing protein
COG5221	0	0	0	0	0	0	0	0	0	0	0	0	0	0	0	0	0	0	Dopey family member
COG2259	23265	27317	22112	34832	41217	38549	34398	41072	37134	30474	35638	35539	38269	32981	41332	25011	23919	22831	doxx family
COG2761	7052	7778	4931	8251	7620	8297	6146	8117	7067	7195	9616	6565	7781	8120	7520	6679	6431	7503	DSBA oxidoreductase
COG3917	1337	1682	1826	1423	1360	1360	2637	1713	1733	2933	2818	2396	1326	1500	1625	3270	2318	2433	dsbA oxidoreductase
COG3048	244	590	1409	5	5	4	16	7	10	153	53	43	3	7	6	1606	1416	1413	d-serine deaminase
COG2044	321	379	275	490	552	617	325	364	409	424	387	461	555	559	582	313	245	268	DsrE family
COG1416	1090	1446	3029	917	1046	984	1548	1314	1281	1700	1376	1819	976	1178	1268	1387	995	1227	DsrE/DsrF-like family
COG3370	315	238	137	5115	4488	4888	1872	4550	3366	2206	5449	1901	4696	4423	3922	140	123	97	DsrE/DsrF-like family
COG1091	8078	8938	7403	12248	13381	13283	13800	13783	13293	12081	12358	14321	12955	12238	14347	9992	9095	8446	Dtdp-4-dehydrorhamnose reductase
COG1088	7466	8302	6423	10977	12230	11887	11598	12186	11691	10154	10600	12199	11850	10765	12888	7919	7955	6795	dTDP-glucose 4-6-dehydratase
COG3148	1244	2039	4313	948	995	1093	1360	1206	1452	1749	1581	1620	1181	1293	1212	4064	3273	3455	Dtw domain containing protein
COG2453	2154	1886	1745	8061	6942	7570	4526	6792	5943	5027	7737	5031	7574	7465	6599	3064	2839	2798	dual specificity phosphatase
COG3752	2106	2768	1472	874	817	890	968	777	966	951	776	1058	837	783	1032	1746	1485	2161	DUF1295 domain protein
COG2428	0	0	0	0	0	0	0	0	0	0	0	0	0	0	0	0	0	0	DUF431 domain-containing protein
COG4508	788	262	60	18	18	18	40	24	43	422	41	78	21	58	25	233	483	491	dUTPase
COG0699	1349	2121	1094	658	16	44	334	67	442	190	189	336	454	840	42	1096	943	1594	Dynamin family
COG4943	714	1439	4199	79	86	70	122	105	87	331	151	167	66	74	92	2223	1819	1967	EAL
COG1980	165	116	34	5187	4567	4957	1899	4608	3411	2244	5497	1928	4753	4474	4006	131	115	57	ec 3.1.3.11
COG3525	7177	6928	4911	15271	19649	18259	17580	19640	17503	15088	15958	18265	17703	15663	20504	6621	7351	4411	ec 3.2.1.52
COG5371	2	2	0	2	0	1	5	2	6	1	1	3	0	3	2	0	0	0	Ectonucleoside triphosphate diphosphohydrolase
COG5569	1446	1470	1839	900	615	555	2886	1518	2335	2058	2401	2114	538	723	746	2637	1999	1851	efflux system protein
COG2025	9055	9066	6443	16814	18100	18398	14847	18472	16152	14291	17846	14729	17425	15711	18124	9989	9199	8482	Electron transfer flavoprotein
COG2086	8994	9101	6556	16514	17764	18051	14706	18284	15930	14011	17560	14457	17059	15370	17725	9899	9158	8357	Electron transfer flavoprotein
COG3043	276	477	1429	99	111	94	109	100	96	199	141	193	89	99	116	381	276	382	Electron transfer subunit of the periplasmic nitrate reductase complex NapAB (By similarity)
COG2863	7446	6987	7244	10173	11436	10860	15104	14332	14544	11630	13121	14490	10524	9277	11744	10482	8704	7780	electron transport chain
COG2878	1453	1710	3068	1321	1016	1123	2307	1702	1981	1944	2080	2215	1166	1209	1073	2665	2225	2217	Electron transport complex
COG4657	734	1316	2894	790	699	811	725	720	717	991	858	916	850	719	730	2187	1848	1975	Electron transport complex
COG4658	784	1327	2890	809	714	824	742	736	728	1014	876	933	859	739	745	2200	1867	2000	Electron transport complex
COG4659	739	1318	2889	804	707	820	736	731	723	1009	868	922	857	728	739	2188	1850	1979	Electron transport complex
COG4660	734	1316	2893	790	699	811	725	720	717	991	858	916	850	719	730	2187	1848	1975	Electron transport complex
COG1999	11813	10097	6079	24486	27585	27223	25573	29136	26334	22434	27027	25800	26033	23272	28268	12482	12317	9573	Electron transport protein SCO1 SenC
COG4888	123	73	20	5111	4486	4888	1841	4543	3356	2200	5445	1879	4696	4420	3921	93	77	37	elongation factor 1 homolog
COG1856	42	41	16	82	94	119	57	51	70	68	57	77	76	96	86	31	42	46	Elp3
COG2100	4	1	1	2	3	1	3	2	1	5	2	10	1	6	0	4	3	11	Elp3
COG4862	1641	531	106	36	35	37	50	32	49	658	49	47	24	28	32	512	938	880	Enables the recognition and targeting of unfolded and aggregated proteins to the ClpC protease or to other proteins involved in proteolysis
COG4193	87	84	55	14	11	15	14	10	16	36	12	24	13	11	12	60	192	317	endo-beta-N-acetylglucosaminidase
COG4724	67	18	6	4	4	7	11	4	13	3	9	9	5	7	4	20	20	14	endo-beta-n-acetylglucosaminidase
COG2840	1917	2426	4743	1410	1133	1156	2652	1967	2225	2380	2322	2571	1054	1309	1313	3253	2626	2789	endodeoxyribonuclease activity
COG1583	362	380	120	894	1002	1069	770	747	756	825	818	944	1138	1005	985	336	410	424	endonuclease activity
COG2374	2706	3594	2194	1934	1127	1198	3196	1593	2008	2115	2069	2574	1653	2377	1457	2450	2260	2478	endonuclease exonuclease phosphatase
COG3021	2791	4092	3715	1270	1067	1156	1665	1002	1157	1391	1094	1527	1064	1142	1266	2369	2205	2761	endonuclease exonuclease phosphatase
COG4222	935	1115	1023	2235	561	629	2481	947	1626	1691	1376	2275	1405	2399	659	1111	917	573	endonuclease exonuclease phosphatase
COG2356	1618	1993	4290	432	408	451	563	442	807	840	460	674	375	441	439	2364	2903	2041	endonuclease I
COG0177	8392	8635	5998	17234	17955	18199	14530	18099	16452	13941	17324	15717	17825	16589	18179	8187	7908	7343	endonuclease III
COG0648	4392	3330	2238	12872	14530	14395	8378	13450	11150	8519	12316	9196	13893	11754	13860	3172	3445	2761	Endonuclease IV plays a role in DNA repair. It cleaves phosphodiester bonds at apurinic or apyrimidinic sites (AP sites) to produce new 5'-ends that are base-free deoxyribose 5-phosphate residues. It preferentially atta [illegible]
COG0164	7883	8001	5936	15964	16476	16516	13430	16754	15099	12653	16170	14142	16211	15021	16418	7768	7380	6813	Endonuclease that specifically degrades the RNA of RNA- DNA hybrids (By similarity)
COG1039	1028	481	150	699	753	930	694	750	753	1076	732	899	747	791	1031	493	773	730	Endonuclease that specifically degrades the RNA of RNA- DNA hybrids (By similarity)
COG3568	9958	12489	11894	8829	8744	9549	11808	9886	10647	10912	10219	12128	8895	9995	9872	14129	13340	12812	Endonuclease/Exonuclease/phosphatase family
COG0251	33310	35805	41236	56888	64187	62932	54162	64767	59969	49606	58230	55952	61161	54224	64779	39949	38282	33312	Endoribonuclease L-PSP
COG1418	8037	7749	6968	7461	9585	8790	8439	9182	8637	8578	7895	8962	8852	7625	9906	7239	7560	6823	Endoribonuclease that initiates mRNA decay (By similarity)
COG3590	11637	10583	4254	24703	32137	29480	24874	31802	27337	21512	24398	25811	28908	23003	32537	9508	9873	6853	endothelin-converting enzyme
COG4056	0	0	0	0	0	0	0	0	0	0	0	0	0	0	0	0	0	0	energy derivation by oxidation of reduced inorganic compounds
COG0623	5959	4911	3528	9908	11047	10506	11279	11672	11294	9958	10247	11573	10631	9840	11428	4873	4746	3726	Enoyl- acyl-carrier-protein reductase NADH
COG1024	47841	47554	38151	58289	65036	64083	68088	68821	72875	60875	68081	66460	61352	54997	66009	53033	46249	45823	Enoyl-CoA hydratase
COG5510	1220	1232	2966	465	382	403	1372	798	1487	890	904	1175	366	372	447	913	781	957	Entericidin
COG5562	21	256	21	77	3	20	88	38	44	182	169	93	49	97	29	12	129	14	envelope protein
COG2371	2394	3624	3221	1838	948	950	3110	1471	1814	2367	2166	2565	1370	1955	1274	3861	3202	3629	enzyme active site formation
COG3450	3997	4927	6830	6630	8378	7684	7103	8386	7552	6423	6835	7093	7542	5988	8415	7527	7311	5782	Enzyme of the cupin superfamily
COG1090	10790	10195	7165	10977	12536	12447	10812	12112	10994	10247	9941	11091	12193	10811	13019	8894	9348	9048	Epimerase
COG0702	28580	30306	19063	42617	47588	45701	43295	47236	43583	37617	42095	43652	45744	41290	48019	25379	23792	23053	epimerase dehydratase
COG1376	13580	14415	10742	18506	20323	20003	16511	17582	17384	15986	15369	19149	19536	17437	21537	14132	12916	12187	ErfK YbiS YcfS YnhG family protein
COG2989	4050	4965	4338	7286	9071	8232	8902	9598	8310	7746	8022	8969	8090	7014	9177	5197	5363	3781	ErfK YbiS YcfS YnhG family protein
COG3034	1849	2475	3505	3617	3716	4155	3377	3754	3255	3335	3545	3875	3533	3632	4058	3464	3053	2785	ErfK ybiS ycfS ynhG family protein
COG3786	987	980	541	1139	1179	1204	1275	1155	1331	1234	1062	1594	1010	830	1393	1027	896	842	ErfK ybiS ycfS ynhG family protein
COG5018	1247	977	1739	967	194	207	966	310	674	982	433	922	606	1113	245	1965	2051	1759	ERI1 exoribonuclease family member
COG2312	3338	3644	2878	709	829	754	1007	792	990	1426	922	1186	715	888	852	3639	4101	4164	erythromycin esterase
COG4842	2593	2659	1264	239	376	386	278	252	414	566	252	369	230	310	364	1694	1602	2235	EssaT-6 like protein
COG4839	2118	2332	1127	20	20	21	85	33	79	343	53	42	15	19	19	1299	1349	2050	Essential cell division protein (By similarity)
COG0206	7951	8102	6113	15854	16260	16377	13334	16612	14978	12511	16050	13961	15918	14905	16306	7759	7382	6766	Essential cell division protein that forms a contractile ring structure (Z ring) at the future cell division site. The regulation of the ring assembly controls the timing and the location of cell division. One of the functions of [illegible]
COG0627	7224	7423	5681	9731	11118	10489	11275	11335	10959	9836	9798	11507	10366	9641	11516	7270	7153	6440	esterase
COG2382	12554	10561	7219	28053	35040	34598	25803	33488	29078	23202	25911	27946	32450	27208	36319	10173	11094	7781	esterase
COG2819	2707	2534	2875	3203	3472	3943	3592	3734	3707	3750	3426	3957	3384	3841	3997	2129	2660	2007	esterase
COG4947	960	966	665	2327	2595	2942	2415	2603	2303	1852	2166	2419	2539	2392	2827	914	1132	727	esterase
COG4302	865	1269	1806	785	783	818	1363	858	947	1339	1337	1163	893	809	999	2442	2027	1975	ethanolamine ammonia-lyase
COG4303	879	1286	1815	797	792	830	1372	871	955	1353	1348	1185	899	826	1013	2463	2042	1999	ethanolamine ammonia-lyase activity
COG4576	2377	1242	344	3664	3109	3761	2956	3211	3103	3319	2776	3334	3793	4225	3610	1318	1698	1484	ethanolamine catabolic process
COG0510	3432	3423	6517	1678	2011	2079	1231	1322	1502	2023	1288	1651	1842	1832	1945	3589	3491	3497	Ethanolamine kinase
COG4812	1	0	1	2	1	1	0	1	1	2	1	3	1	1	2	2	2	0	Ethanolamine utilization cobalamin adenosyltransferase
COG4766	60	151	177	7	8	7	43	19	33	20	12	38	5	7	10	58	54	23	ethanolamine utilization protein
COG4917	24	28	26	7	6	9	5	7	9	18	10	18	6	8	8	26	75	131	ethanolamine utilization protein
COG3192	24	29	26	8	6	10	6	5	9	17	10	15	8	8	7	26	75	131	Ethanolamine utilization protein eutH
COG4820	21	41	11	30	14	26	64	38	45	114	98	87	14	35	35	80	30	23	ethanolamine utilization protein eutJ
COG5053	0	0	0	0	0	0	0	0	0	0	0	0	0	0	0	0	0	0	eukaryotic translation initiation factor
COG0530	3432	2709	3242	4161	3742	3977	3813	3305	4009	4408	3484	4575	4254	4553	4090	3665	3479	3238	exchanger
COG1370	123	73	20	5111	4486	4888	1841	4543	3356	2200	5446	1879	4696	4420	3922	93	77	37	Exchanges the guanine residue with 7-cyano-7- deazaguanine (preQ0) at position 15 in the dihydrouridine loop (D- loop) of archaeal tRNAs (By similarity)
COG2827	4920	6173	5032	9725	11172	10119	11325	11510	10551	10750	10088	12116	11563	10062	11741	6718	6272	5126	Excinuclease abc c subunit domain protein
COG0692	4399	5096	5048	1585	1490	1470	3198	1965	2540	2849	2396	2756	1415	1673	1645	4695	4323	4633	Excises uracil residues from the DNA which can arise as a result of misincorporation of dUMP residues by DNA polymerase or due to deamination of cytosine (By similarity)
COG3315	3421	2640	960	7486	9661	8917	8161	9187	8237	6658	7399	8333	8471	7142	9642	2873	2848	1905	Exhibits S-adenosyl-L-methionine-dependent methyltransferase activity (By similarity)
COG5173	0	0	0	0	0	0	0	0	0	0	0	0	0	0	0	0	0	0	Exocyst complex component
COG2925	893	1279	2920	546	388	428	908	758	732	1027	1043	1074	379	527	455	2331	1908	1890	Exodeoxyribonuclease I
COG0708	11033	12181	9683	11825	12547	12118	14773	14081	13893	13103	13068	14981	11887	11634	13351	11875	10798	10638	Exodeoxyribonuclease III
COG0507	10649	11486	10010	17098	19066	17942	18981	19280	17829	16434	16505	18997	18402	16891	19913	10557	10007	8698	exodeoxyribonuclease v alpha
COG0420	6388	6206	5272	15063	13020	14219	9178	13067	11354	9400	15003	9441	13882	13801	12171	5770	5884	6153	Exonuclease
COG0349	4260	5477	5290	4708	3318	3573	4580	3633	4386	4534	3892	5227	3900	4908	3796	5217	4428	5047	Exonuclease involved in the 3' processing of various precursor tRNAs. Initiates hydrolysis at the 3'-terminus of an RNA molecule and releases 5'-mononucleotides (By similarity)
COG3359	1133	549	164	808	851	973	611	669	698	1002	710	850	838	866	921	555	778	835	Exonuclease-like protein
COG4632	1244	896	253	2519	1669	1848	3494	1774	2214	2229	1993	3120	2038	2916	1916	1155	1347	959	Exopolysaccharide biosynthesis protein
COG3932	877	928	619	2505	1026	1030	2269	1273	1904	1894	1552	2499	1763	2811	1305	956	841	661	Exopolysaccharide synthesis
COG1096	123	73	20	5111	4486	4888	1841	4543	3356	2200	5446	1879	4696	4420	3922	93	77	37	Exosome complex
COG1097	123	73	20	5112	4486	4890	1841	4544	3356	2200	5446	1879	4697	4422	3922	93	77	37	Exosome complex
COG2123	152	197	172	5113	4486	4890	1873	4556	3363	2210	5454	1892	4698	4422	3923	125	105	50	Exosome complex
COG1325	123	73	20	5112	4486	4890	1841	4544	3356	2200	5446	1879	4697	4422	3922	93	77	37	exosome subunit
COG4083	210	179	122	70	89	104	66	74	59	90	84	88	115	184	138	66	60	74	Exosortase EpsH-related protein
COG1430	2449	2098	794	10767	9449	10642	7511	9944	8994	6988	10557	7924	10224	10346	9477	2266	1833	1615	exported protein
COG2911	5112	5595	4733	11401	12453	12409	12516	13162	12249	10593	11275	12718	11973	11299	13161	6314	6065	4528	exported protein
COG3111	643	1021	2826	79	80	87	391	243	596	259	236	357	76	89	127	1835	1590	1596	exported protein
COG3164	1013	1359	2943	815	557	639	1602	1162	1118	1437	1541	1471	583	854	723	2400	1954	1950	exported protein
COG5101	0	0	0	0	0	0	0	0	0	0	0	0	0	0	0	0	0	0	exportin
COG3921	1124	1941	3420	550	585	535	675	635	599	1005	708	943	507	564	664	2649	2054	2147	Extensin Family
COG5155	0	0	0	0	0	0	0	0	0	0	0	0	0	0	0	0	0	0	extra spindle pole bodies homolog 1 (S. cerevisiae)
COG3181	32757	24945	11906	8276	6955	6370	41434	21503	52897	20315	31911	33914	7193	7128	7696	18859	12664	15236	extra-cytoplasmic solute receptor family protein
COG3384	4106	5533	6213	2113	1761	1790	4124	2799	3377	3866	3703	3956	1698	2124	2010	6460	5473	5911	extradiol ring-cleavage dioxygenase class III protein subunit b
COG3885	80	60	30	137	151	204	64	106	100	129	141	137	182	240	239	61	27	44	extradiol ring-cleavage dioxygenase class III protein subunit b
COG4071	1	0	0	0	0	0	0	0	0	0	0	0	0	0	0	0	0	0	F420-0:Gamma-glutamyl ligase
COG1813	2909	2208	1927	6989	6480	6795	5912	7011	6370	5262	8366	5308	6588	6480	5999	3520	3071	2817	Factor 1
COG3806	731	777	757	899	667	657	1658	998	1408	1187	945	1629	584	774	839	577	491	490	Factor ChrR
COG2509	1149	1664	1659	1419	1240	1322	2287	1541	1575	2458	2164	2307	1176	1501	1372	2907	2377	2133	fad dependent oxidoreductase
COG3380	619	942	1591	1122	272	329	1189	460	823	972	725	1141	732	1229	422	1978	1628	1566	fad dependent oxidoreductase
COG0277	33163	36854	30975	46763	49894	50078	46344	50054	48949	41378	47053	48762	48813	46210	51599	34924	31663	32607	FAD linked oxidase domain protein
COG1720	1445	1806	3084	7958	7470	8375	3978	7338	5867	4327	7724	4346	7541	7183	7037	2777	2439	2341	family UPF0066
COG1598	5098	5442	6637	14328	12212	12202	11799	12277	13041	9688	10167	12493	14367	14355	12441	7190	6910	5689	family UPF0150
COG3000	4593	5925	6088	5888	4440	4480	10316	6213	6891	9196	7824	9383	5425	6744	5462	9064	8300	6921	fatty acid hydroxylase
COG4981	2	2	8	1	0	1	8	6	4	6	29	22	2	2	2	5	3	0	Fatty acid synthase
COG4982	4480	7190	4706	407	492	479	1047	548	662	833	717	948	430	526	517	5174	4090	6488	fatty acid synthase
COG2912	1216	1750	3056	1733	1535	1757	3131	2008	1967	2836	2360	2999	1777	1935	2108	2830	2444	2462	F-box protein 21
COG0633	9216	9990	9078	16949	11447	11311	21800	14893	18094	15407	15224	19714	14230	16991	12494	11155	9640	8807	Ferredoxin
COG1141	7022	9468	4618	2448	1976	2253	2117	1462	2130	2057	1535	2349	2379	2793	1993	5319	4555	7640	Ferredoxin
COG3411	3932	3264	1081	11361	10727	10523	11181	10882	10556	9304	9753	11299	11340	11148	11235	3573	3615	2650	Ferredoxin
COG3894	533	546	142	1282	1296	1601	1011	1260	1120	1334	1178	1395	1455	1444	1584	540	498	460	Ferredoxin
COG4739	48	54	3	190	176	210	164	190	153	148	178	186	167	172	174	72	68	37	ferredoxin domain containing protein
COG4802	189	214	52	1256	479	549	1118	545	838	694	572	1096	1064	1344	513	194	231	167	ferredoxin thioredoxin reductase
COG2440	1115	704	331	467	440	498	890	632	637	1086	763	762	431	453	542	717	675	659	ferredoxin-like protein
COG4114	1499	1442	3047	100	91	56	544	232	279	730	411	265	75	101	64	2250	2232	2257	ferric iron reductase
COG2406	283	180	58	5428	4852	5278	2074	4809	3649	2412	5716	2197	5072	4774	4284	196	193	123	ferritin dps family protein
COG1918	2550	2727	2414	5756	5700	6536	6027	5702	5315	5566	5554	6328	5639	5725	6344	2876	2696	2148	ferrous iron transport protein
COG0370	4938	4195	2836	10385	11201	10950	10556	11039	10192	9660	9627	10974	10865	9926	11759	4337	4458	3225	Ferrous iron transport protein b
COG0396	4945	5293	3908	9560	8277	9032	6100	8304	7564	6555	9129	6924	8717	9203	8235	4010	3862	4162	feS assembly ATPase SufC
COG0719	9055	10363	7792	18770	16207	17568	12233	16229	14737	12639	18034	13630	17236	18072	16011	7687	7262	8155	Fe-S assembly protein
COG2975	2968	2613	2019	6171	8040	7229	7357	8394	7540	5802	6715	7051	7159	5735	8026	3695	3568	2641	Fe-S assembly protein IscX
COG2151	10764	12229	7150	15442	17011	17074	13114	16827	15186	12888	16276	13788	16233	14964	17191	10036	9126	9918	fes assembly suf system protein
COG4871	3	3	0	24	18	18	14	17	20	34	17	18	34	16	24	3	5	3	Fe-S cluster domain protein
COG2166	3570	5270	6387	2907	2249	2395	3014	2390	2837	3010	2400	3543	2471	3103	2627	4823	4159	4544	Fe-S metabolism associated
COG3313	2236	3638	7446	1027	727	710	3219	1748	2109	2940	2886	2443	701	871	900	7895	6328	6132	Fe-S protein
COG0727	2237	4342	9048	2137	1317	1407	2579	1714	2481	2931	2166	2978	1950	2373	1566	9265	7796	7475	Fe-S-cluster oxidoreductase
COG1716	14002	15666	7616	21299	18439	19135	20545	17285	18836	17106	15872	21618	21145	21991	20467	11342	10342	13533	fha domain-containing protein
COG3456	1124	1715	4338	616	642	719	604	577	615	994	827	898	672	592	863	2504	2909	2434	fha domain-containing protein
COG3401	1192	974	319	1952	2091	2514	1937	2146	2032	1987	2092	2308	2040	2147	2531	1002	876	818	fibronectin type III domain protein
COG1293	1377	696	205	6771	5296	5874	3252	5313	4537	3707	6394	3392	6032	6263	4959	705	941	922	Fibronectin-binding A domain protein
COG3177	7166	7398	7942	11684	13079	12627	12302	12961	12330	11377	11587	13089	12748	11811	13641	7570	6645	6022	Filamentation induced by cAMP protein fic
COG3210	611	867	1498	5024	517	820	3690	1134	3147	2135	1695	3495	3212	5736	808	1901	1887	1486	filamentous hemagglutinin family outer membrane protein
COG3166	3633	3444	3627	8206	8900	8400	8484	9302	8483	7130	7894	8570	8482	7621	9069	4603	4228	3382	Fimbrial assembly family protein
COG3539	2589	4237	9802	337	172	106	2292	1176	2629	1474	1316	1842	151	205	152	9995	8947	8047	Fimbrial protein
COG4969	1320	1555	3049	1282	1027	1149	2077	1657	1751	1761	1849	2118	1045	1163	1351	2664	2207	2249	Fimbrial protein
COG3882	2758	2143	663	6742	8682	8187	6479	8549	7281	5701	6573	7000	7817	6336	9017	2276	2402	1519	FkbH Like Protein
COG3352	39	9	0	1	1	0	3	0	2	0	6	1	1	3	2	13	14	11	Flagella accessory C family protein
COG3351	0	0	0	1	0	2	0	0	0	0	0	0	1	2	0	0	0	0	flagella protein
COG3418	906	1151	2857	242	105	74	1212	587	989	587	646	726	103	135	101	1997	1678	1687	Flagella synthesis protein FlgN
COG2257	1861	1316	1591	755	525	568	1778	1185	1443	1493	1280	1464	476	697	558	2369	2285	2157	flagellar
COG2874	162	129	40	5163	4574	4940	1917	4615	3457	2357	5523	2058	4769	4575	4026	159	147	91	Flagellar accessory protein FlaH
COG1317	5283	4325	3804	8017	9675	9186	9879	10379	9679	8276	8992	9568	9148	7668	9975	5425	5177	4353	Flagellar assembly protein
COG1955	125	74	22	5116	4488	4895	1846	4547	3361	2204	5453	1886	4700	4425	3924	94	78	37	flagellar assembly protein J
COG1580	4228	5200	5850	2101	1896	2062	3061	2515	2784	3332	2875	3202	2063	2160	2126	6150	5265	6055	Flagellar basal body-associated protein fliI
COG4786	9253	7593	5041	15571	19253	18253	15648	19232	16927	15204	15912	16722	17853	14924	19808	8721	8502	6975	flagellar basal-body rod protein
COG1558	5233	4672	4190	8197	9858	9371	8773	10190	9177	8319	8657	9201	9246	7776	10212	5673	5256	4451	flagellar basal-body rod protein flgc
COG4787	1409	1587	3039	812	481	557	2109	1324	1616	1590	1744	1763	506	713	570	2630	2155	2108	Flagellar basal-body rod protein FlgF
COG1298	5101	4419	3982	7961	9658	9158	8527	9953	8981	8027	8443	8867	9035	7539	9930	5433	5117	4315	Flagellar biosynthesis protein flha
COG1419	1798	1376	1638	1190	899	1128	1868	1500	1408	2097	1892	1814	989	1259	1123	2587	2391	2279	Flagellar biosynthesis regulator FlhF
COG5442	342	477	285	410	449	397	487	464	454	664	509	627	334	390	508	458	292	307	flagellar biosynthesis regulatory protein FlaF
COG5443	397	545	318	448	516	423	533	504	500	713	548	694	387	451	556	598	342	367	Flagellar biosynthesis repressor FlbT
COG1377	5210	4513	4039	8136	9809	9310	8942	10213	9276	8305	8814	9199	9223	7756	10143	5547	5207	4595	Flagellar biosynthetic protein flhB
COG1338	5113	4432	3979	7987	9692	9190	8537	9974	9014	8058	8478	8900	9068	7580	9969	5450	5123	4413	Flagellar biosynthetic protein FliP
COG1987	5141	4467	3995	8038	9745	9243	8578	10018	9047	8124	8517	8977	9108	7629	10018	5491	5148	4459	Flagellar biosynthetic protein fliq
COG1582	3197	2153	615	6127	8082	7485	5453	7581	6425	5144	5649	5813	7338	5668	8133	2142	2478	1731	flagellar FlbD family protein
COG1843	5274	4581	4041	8285	10020	9445	8817	10248	9278	8393	8734	9300	9382	7953	10287	5676	5282	4492	Flagellar hook capping protein
COG1345	5164	4477	5167	7665	9299	8808	8328	9639	8658	7660	8210	8468	8769	7242	9509	5484	5223	4462	flagellar hook-associated
COG1677	5162	4528	4005	8122	9879	9347	8631	10098	9120	8146	8550	9054	9383	7701	10091	5510	5187	4398	flagellar hook-basal body complex protein fliE
COG3144	2231	1998	3144	1300	1138	1219	2075	1612	1507	2152	1902	1995	1247	1256	1177	3115	2815	2771	Flagellar hook-length control protein
COG1886	6833	6149	4560	14216	17818	16559	14779	17966	16149	13084	14615	15324	16294	13236	18105	7243	6631	5217	Flagellar motor switch protein
COG3353	0	0	0	1	0	2	0	0	0	0	0	0	1	2	0	0	0	0	flagellar protein
COG1334	1667	1208	1549	641	451	507	1183	822	1037	1274	1041	1101	407	629	490	2278	2219	2090	flagellar protein FlaG
COG1516	4803	4006	3720	7616	9269	8796	8095	9549	8595	7432	7993	8345	8741	7203	9488	5100	4877	4078	flagellar protein FliS
COG1705	4002	3819	3871	6997	8895	8096	8796	9396	8388	7030	8023	8500	7957	6733	8879	4864	4776	3437	flagellar rod assembly protein muramidase flgj
COG3951	544	673	342	631	644	670	803	719	681	828	677	826	745	594	656	592	499	493	flagellar rod assembly protein muramidase flgj
COG1344	12412	11229	9489	18266	21633	20902	19661	22486	20477	19910	19845	21013	20345	17815	22742	12909	11968	10393	Flagellin
COG3430	0	0	0	2	1	4	0	1	0	1	1	0	2	4	0	0	0	0	flagellin domain protein
COG1157	5409	4593	4065	8089	9753	9220	9162	10269	9691	8265	8824	9313	9126	7646	10025	5557	5200	4498	flagellum-specific ATP synthase
COG4536	1948	1907	3269	1080	1019	981	1988	1468	1468	2061	1677	1773	888	843	1194	2876	2621	2545	flavin adenine dinucleotide binding
COG1036	40	58	14	112	36	47	218	108	105	275	222	201	40	90	77	91	47	44	flavoprotein
COG3847	8069	10830	7287	7429	9508	8705	8617	9671	9014	7510	8096	8403	8689	6962	9682	9889	8308	9673	Flp Fap pilin component
COG3745	6367	8764	5373	3797	4203	4573	4512	4297	4751	3523	3985	4244	4171	3742	4301	6584	6054	7487	Flp pilus assembly protein CpaB
COG2457	201	167	47	5273	4590	5025	2091	4703	3499	2540	5710	2131	4809	4578	4084	234	157	128	FMN binding
COG4939	2	0	1	1	1	0	2	1	1	1	1	2	1	0	2	1	2	1	FMN-binding domain
COG3976	1766	2441	1276	321	372	394	347	292	396	278	310	405	364	388	375	1303	1120	1765	fmN-binding domain protein
COG0285	8551	8190	5989	10990	12111	11827	11673	12324	11845	10924	10885	12401	11734	10882	12775	7919	7762	7243	Folylpolyglutamate synthase
COG1795	440	449	168	926	807	853	1478	1077	1047	1417	1313	1335	933	854	951	539	354	448	Formaldehyde-activating enzyme
COG2421	4763	4152	1621	10178	12278	12051	10032	11859	10681	8919	9763	10567	11295	9724	12590	4249	3766	2973	formamidase
COG1882	812	803	1558	1654	823	913	1557	797	1104	1200	1130	1392	1243	1793	825	755	690	654	formate acetyltransferase
COG3383	12894	14095	10697	19225	23741	23114	19256	22986	20682	17715	19093	20323	22229	18324	24346	13669	12807	12235	Formate dehydrogenase Alpha subunit
COG2116	4295	4687	7773	7704	9931	9345	6852	9346	7919	6576	7234	7971	9505	7488	9893	3993	3885	3598	formate transmembrane transporter activity
COG0184	7697	7413	4588	15936	16437	16502	13385	16730	15064	12473	16064	14065	16242	15040	16434	6141	6069	5539	Forms an intersubunit bridge (bridge B4) with the 23S rRNA of the 50S subunit in the ribosome (By similarity)
COG1029	78	95	28	162	104	137	251	161	143	341	265	253	113	158	163	145	80	94	formylmethanofuran dehydrogenase subunit B
COG2191	2425	1981	550	6493	8423	7790	5915	8050	6844	5225	5981	6418	7722	5915	8335	1952	2069	1278	Formylmethanofuran dehydrogenase subunit E
COG0788	7744	10670	8804	3866	3105	3272	5998	3768	5001	4682	4555	5345	3353	4101	3456	9211	7744	9427	formyltetrahydrofolate deformylase
COG2759	3424	3283	1512	1319	1351	1530	1282	1306	1290	2126	1418	1655	1495	1456	1657	2163	2268	3011	formyltetrahydrofolate synthetase
COG1965	2835	2573	1968	6093	7862	7188	6679	8009	7146	5567	6636	6495	7091	5563	7913	3670	3392	2555	frataxin-like protein
COG3001	921	866	1643	1625	715	813	1850	926	1237	1198	1096	1737	1195	1891	824	680	726	700	Fructosamine
COG3588	684	657	422	928	872	935	1410	1086	1095	1040	1073	1484	849	891	1045	671	734	661	fructose-bisphosphate aldolase
COG1838	3150	2791	2114	7400	9293	8887	7298	9138	8171	6456	7334	7643	8564	6974	9524	2711	2568	1937	fumarate
COG1951	5212	4787	5639	8466	10235	9931	8754	10295	9477	8352	8708	9246	9461	8047	10672	5543	5331	4576	fumarate
COG0114	5592	6484	6729	8464	7729	8339	7156	8607	7817	7084	9720	7277	7844	7963	7699	7254	6548	6788	fumarate hydratase class II
COG3573	533	504	550	251	293	288	183	150	213	208	161	258	332	336	309	373	499	442	fumarate reductase succinate dehydrogenase flavoprotein domain protein
COG3970	3008	3494	1659	8239	7865	8741	5649	8175	7052	5195	8841	5472	8008	7773	7696	2595	2269	2551	fumarylacetoacetate (faa) hydrolase
COG3802	494	954	1659	149	106	86	452	206	212	574	382	322	85	124	133	1926	1617	1489	Fumarylacetoacetate (FAA) hydrolase family
COG0179	29881	36816	29337	32297	35244	35337	32643	36963	34563	29386	36583	31670	34215	31302	34954	30252	26495	30632	Fumarylacetoacetate hydrolase
COG2383	0	3	0	5	5	2	3	2	4	3	2	0	3	2	3	0	1	2	fun14 family
COG1289	12573	14571	23040	13840	17522	15873	17213	18355	17517	14317	15280	16743	15706	12771	17945	17178	16278	14981	Fusaric acid resistance protein
COG3030	1781	1772	3214	840	870	949	761	811	833	1358	861	980	849	859	956	2561	2462	2585	fxsa cytoplasmic membrane protein
COG5024	0	0	0	0	0	0	0	0	0	0	0	0	0	0	0	0	0	0	g2 mitotic-specific
COG5620	98	209	203	51	86	49	108	83	110	156	94	190	73	152	106	136	122	88	GAD-like protein
COG1956	5677	6022	4945	6819	8703	8004	7526	8786	8142	6684	6956	7680	7862	6458	8863	5737	5686	5333	gaf domain protein
COG4250	130	76	40	1566	82	152	1108	244	935	569	434	1018	971	1816	145	60	154	60	GAF sensor signal transduction histidine kinase
COG1085	7186	7310	4212	14529	18066	16859	12728	16714	14708	11171	12897	13729	16912	13682	17996	6034	6165	5054	galactose-1-phosphate uridylyltransferase
COG0405	14518	15464	13877	19150	21671	20781	20657	22018	21671	18963	19307	21411	20595	18795	22685	15879	14379	13862	gamma-glutamyltransferase activity
COG1610	5431	4377	1734	9461	11489	11074	10018	11365	10512	9243	9724	10731	10658	9247	11887	4388	4558	3352	gatB Yqey
COG3375	2474	1984	688	5824	7822	7085	5328	7397	6218	4647	5431	5654	6922	5307	7697	1826	2085	1420	gCN5-related N-acetyltransferase
COG3393	7428	6546	4198	7726	9965	9239	7528	9230	8384	7331	7408	7963	8847	7238	9832	5099	5245	5041	Gcn5-related n-acetyltransferase
COG5352	625	806	435	728	736	666	936	840	805	1163	840	1102	592	692	824	863	579	630	gcrA cell cycle regulator
COG5044	0	0	0	0	0	0	0	0	0	0	0	0	0	0	0	0	0	0	GDP dissociation inhibitor
COG3240	2902	3068	3372	6232	6312	5292	7056	6929	6859	5747	6046	6866	6384	5966	6400	5300	4661	3804	GDSL esterase lipase
COG1080	6343	7344	10641	3643	3771	4066	4712	4005	4432	4683	4233	4939	3801	3723	4359	7796	7714	8014	General (non sugar-specific) component of the phosphoenolpyruvate-dependent sugar phosphotransferase system (sugar PTS). This major carbohydrate active-transport system catalyzes the phosphorylation of incom [illegible]
COG2101	246	146	40	10223	8972	9778	3682	9086	6712	4400	10894	3758	9393	8842	7846	186	154	74	General factor that plays a role in the activation of archaeal genes transcribed by RNA polymerase. Binds specifically to the TATA box promoter element which lies close to the position of transcription initiation (By sim [illegible]
COG3149	778	954	1549	436	281	354	1272	724	696	1133	1191	940	320	358	370	2118	2442	1694	General secretion pathway
COG2165	36343	33681	17595	89812	105375	112769	81906	100574	89143	86471	88064	96352	106356	99510	116328	40350	37247	30700	General secretion pathway protein
COG3267	2156	2394	1971	3438	3384	3741	3982	3847	3392	4369	4134	4640	4109	3533	3620	3701	3192	3075	General secretion pathway protein
COG1450	10650	9479	5430	24491	30108	28897	24768	29687	26670	22292	24689	26220	27657	22843	31356	12114	11368	8742	general secretion pathway protein D
COG4970	4739	4311	4148	5981	5616	6137	7713	5933	6174	6848	6599	7872	5645	4770	6450	7224	6693	6044	general secretion pathway protein h
COG4795	1999	2154	3307	2960	2077	2296	3386	2332	2730	2704	2633	3460	2360	2605	2377	3338	3711	2982	General secretion pathway protein J
COG3871	6445	6442	3130	8062	10406	9667	8103	9531	8596	7290	7707	8595	9356	7513	10544	5073	5404	4789	general stress protein
COG4768	1705	588	149	33	45	36	53	31	53	653	56	50	27	36	42	538	1079	1128	general stress protein

COG4980	4323	3199	1004	7231	8338	7685	7384	8339	7516	6594	6629	7486	7977	7034	8522	2912	3833	2463 general stress protein
COG0644	18135	17300	10449	55047	55054	55829	42399	54362	48640	39846	53379	44589	55488	51745	55140	19714	19366	15607 geranylgeranyl reductase
COG5072	0	0	0	0	0	0	0	0	0	0	0	0	0	0	0	0	0	0 germ cell associated 2 (haspin)
COG5401	2367	2526	1181	1133	359	453	1023	366	786	903	485	950	737	1233	427	1517	1520	2123 germination protein
COG3452	31	58	26	49	48	54	37	46	34	75	39	67	40	41	50	47	36	36 ggdef family
COG1829	123	73	20	5112	4486	4890	1842	4545	3356	2202	5447	1882	4697	4422	3924	93	77	37 Ghmp kinase
COG2605	2767	2397	816	7263	9448	8794	6468	8656	7408	5828	6634	7108	8703	6825	9442	2378	2510	1613 Ghmp kinase
COG4542	69	85	45	19	17	14	45	28	36	77	27	71	13	14	11	66	49	35 GHMP kinase
COG3193	7664	9469	11324	9934	11885	11503	11330	12524	11879	10136	11096	11427	10968	9385	12125	11018	9703	9204 Glcg protein
COG1820	7548	8091	8149	9768	11188	10677	10661	10942	10335	9002	9193	10741	10607	9536	11586	7430	7507	7000 GlcNAc 6-P deacetylase
COG3076	203	370	1395	0	1	0	2	3	1	9	4	3	0	5	0	223	205	292 Globally modulates RNA abundance by binding to RNase E (Rne) and regulating its endonucleolytic activity. Can modulate Rne action in a substrate-dependent manne[illegible]
COG0684	15625	20033	19109	17063	19015	19184	15656	19343	17771	14606	18381	16030	18221	16308	19029	16017	14698	16705 Globally modulates RNA abundance by binding to RNase E (Rne) and regulating its endonucleolytic activity. Can modulate Rne action in a substrate-dependent manne[illegible]
COG3131	1636	2687	5866	1382	1334	1516	1782	1662	1670	2357	2030	2284	1265	1377	1752	4679	3816	3995 glucan biosynthesis protein
COG4282	365	240	350	589	564	574	961	515	539	531	529	866	496	594	564	215	336	141 Glucan synthesis regulatory protein
COG4578	43	55	34	24	37	33	29	21	28	45	23	42	22	30	25	45	41	34 Glucitol operon activator
COG0837	3667	3637	4074	6859	7546	6631	8141	8249	7838	7095	7195	8296	6998	6477	7885	4638	4277	3450 glucokinase (EC 2.7.1.2)
COG2610	8154	10112	13897	1571	1661	1553	3389	2123	2457	4181	2789	3123	1498	1713	1919	12858	11657	12872 Gluconate
COG0364	11594	12579	9257	14999	18069	16586	15995	17882	16408	14238	14829	16660	17065	14503	18419	12074	11418	10785 Glucose-6-phosphate 1-dehydrogenase
COG0166	8355	8296	6102	16107	16699	16713	13734	16890	15571	12711	16257	14359	16447	15301	16662	7852	7567	7057 glucose-6-phosphate isomerase activity
COG5520	4585	3757	1682	8029	10109	9448	9582	10085	9030	8032	8545	9465	9087	8134	10355	3491	4270	2431 glucosylceramidase activity
COG3643	2487	2009	566	6435	8535	7840	5827	8002	6810	5194	5994	6387	7819	6022	8496	1967	2103	1279 Glutamate formiminotransferase
COG0067	399	464	267	5558	4882	5351	2373	4992	3756	2696	5905	2440	5077	4848	4379	460	356	241 glutamate synthase
COG0069	9782	10785	9582	18517	18336	18553	16805	19081	18066	15771	18993	17387	18690	17927	18592	12584	11423	10220 glutamate synthase
COG0070	256	395	252	468	452	519	531	467	402	513	460	573	425	461	509	373	277	206 glutamate synthase
COG0001	11535	10356	7809	30369	29448	30814	20553	29119	25111	20330	30102	21029	30340	28409	28446	11393	11246	10250 Glutamate-1-semialdehyde aminotransferase
COG2918	619	1084	2880	137	63	64	762	351	337	588	679	410	93	127	70	2052	1725	1693 glutamate--cysteine ligase
COG3572	931	847	445	848	876	828	1309	1091	1517	1188	1005	1545	714	811	937	780	536	650 glutamate--cysteine ligase
COG2066	5705	7055	6050	4189	3606	4163	4042	3676	3823	3666	3407	3952	3939	4131	4093	6056	5523	6564 Glutaminase
COG0121	1763	2378	4775	2155	1307	1382	3058	1615	2084	2374	2218	2501	1784	2228	1556	3329	2785	2955 Glutamine amidotransferase
COG2071	6969	7968	7294	9480	11657	11220	10168	11442	10269	9417	10024	10560	10849	9053	12022	8767	8470	7719 Glutamine amidotransferase
COG3823	2800	2606	1102	6563	8383	7643	7043	8417	7486	5772	6502	7120	7451	6065	8432	2538	2593	1464 Glutamine cyclotransferase
COG0034	7812	7939	5928	11051	12203	11908	11560	12159	11679	10427	10693	12139	11768	10944	12791	7651	7315	6781 glutamine phosphoribosylpyrophosphate amidotransferase
COG0174	16978	19725	22002	26564	28339	28086	24760	28631	26220	23374	27065	25685	27566	25416	28763	23854	21661	20565 glutamine synthetase
COG3968	420	669	383	957	1102	1119	1157	890	898	1143	972	1279	1130	1133	1105	605	661	297 glutamine synthetase
COG0278	2083	2278	3491	2481	1278	1316	3989	2536	3244	3192	3016	3863	1655	2421	1558	3374	2696	2595 Glutaredoxin
COG0695	15781	18309	15506	21769	23130	22334	19912	23525	21751	18352	22187	20445	22518	20507	23249	16762	15824	16508 Glutaredoxin
COG2999	317	499	1464	119	82	107	137	126	120	165	105	165	82	86	88	342	338	420 glutaredoxin 2
COG4545	1	1	0	0	2	1	1	1	1	4	1	2	0	1	0	3	1	1 glutaredoxin-related protein
COG3788	213	250	85	280	238	261	360	300	298	465	301	396	197	230	271	243	217	251 glutathione
COG0386	8353	9339	10122	9293	11128	10697	11254	11748	11026	10551	10503	11631	10256	9174	11890	11250	10402	9877 glutathione peroxidase
COG0435	2934	4374	5852	2209	1501	1698	2055	1584	1902	1831	1628	2423	1847	2463	1834	3999	3463	4194 glutathione Stransferase
COG0625	22992	29918	46688	20251	14088	14017	37348	23430	31058	32247	29243	34809	15226	19582	16656	45464	36940	36181 glutathione Stransferase
COG3791	5381	8024	8992	4955	3826	3870	8506	5658	7222	7514	6730	8195	4081	4998	4536	9507	8240	7406 Glutathione-dependent formaldehyde-activating Gfa
COG0754	3422	4135	4356	884	935	1042	1286	1204	1363	1576	1142	1463	798	936	1148	3812	3393	4161 Glutathionylspermidine synthase
COG0057	11578	13215	12860	17558	16953	17103	16128	17764	16473	14845	17563	16290	17223	16724	17056	13704	12864	12455 glyceraldehyde3phosphate dehydrogenase
COG1929	6145	8514	6656	1559	1842	1919	2203	1823	2325	1786	1750	2155	1714	1787	1947	6015	5293	7392 Glycerate kinase
COG4909	18	37	18	7	4	5	12	6	6	26	8	20	5	6	1	24	23	16 glycerol dehydratase
COG2937	795	1625	4245	292	290	288	382	387	446	652	402	591	262	308	308	3518	2950	3040 glycerol3phosphate acyltransferase
COG0240	8050	8137	6076	10943	12039	11672	11941	12395	11965	10610	10960	12442	11618	10751	12590	7816	7417	6898 glycerol-3-phosphate dehydrogenase
COG0578	9467	11009	8958	10005	11439	10920	10595	11397	10953	9729	10194	11108	10856	9875	11760	10448	9646	9687 glycerol-3-phosphate dehydrogenase
COG1954	950	326	69	105	119	139	95	70	102	446	106	128	90	116	108	348	529	468 glycerol-3-phosphate responsive antiterminator
COG0584	16855	18605	12377	13844	15254	14860	17002	15418	15321	15286	14672	16760	14660	13915	15965	15838	15748	16080 glycerophosphoryl diester phosphodiesterase
COG4781	1519	2277	1213	5	4	2	93	24	64	21	33	46	2	6	4	1129	995	1765 glycerophosphoryl diester phosphodiesterase
COG2113	3597	6112	12474	885	830	809	3210	1707	2426	2513	2321	2167	839	829	916	12585	11365	10315 Glycine betaine
COG4175	2066	3344	6629	544	576	559	1497	922	1244	1293	1113	1237	478	482	625	5824	5005	4789 Glycine betaine
COG4176	1914	2915	5431	539	570	549	1538	948	1246	1189	1112	1225	483	486	621	4283	3728	3537 Glycine betaine
COG2716	1044	1821	4347	338	289	320	499	468	571	859	478	694	301	427	411	3611	3037	3096 glycine cleavage system transcriptional repressor
COG1523	13559	14370	10158	16550	19579	18436	16058	18926	17828	14610	15464	17100	18861	16236	20242	11789	11509	11819 Glycogen debranching enzyme
COG3408	12935	15086	6561	18477	21203	20210	20529	21109	19551	16272	17626	20240	20504	18858	22123	10763	10374	10796 Glycogen debranching enzyme
COG2152	4231	4275	1397	8912	11146	10674	10296	11035	9726	8631	9314	10394	10234	9305	11634	3796	4377	2443 Glycosidase
COG5448	265	273	115	242	317	232	336	289	292	467	357	444	209	237	315	367	306	229 glycoside hydrolase (Family
COG3387	9931	9546	5172	18186	21996	21011	17517	20677	19019	15423	16932	18968	20725	17736	22563	8393	7965	6943 glycoside hydrolase 15-related
COG3664	5239	4784	2780	12469	16329	14995	12471	16094	13834	10884	12294	13031	14616	11792	16624	5713	5593	3922 Glycoside hydrolase family
COG5434	12984	13318	9648	26192	32808	31774	29802	33242	29171	23907	26621	29176	30379	26542	34447	13006	13405	10057 Glycoside hydrolase family 28
COG1486	4176	4453	3473	2491	2996	3066	1975	1961	2174	2251	2064	2370	2798	2843	2820	3083	2961	3383 glycoside hydrolase family 4
COG2730	6215	6481	2854	11130	13420	13415	11298	12499	11495	10459	11093	12058	12424	11629	14413	5387	5289	4686 Glycoside hydrolase Family 5
COG3828	10655	11341	4764	18065	23043	22343	16813	21513	18751	15508	16772	18587	21251	17332	23829	9286	9065	8097 glycosyl hydrolase
COG4225	5708	5116	2947	11893	15750	14082	14042	15865	13727	11857	12847	13973	14121	12168	16304	4921	5502	3092 Glycosyl Hydrolase Family 88
COG3551	218	277	105	944	367	389	483	323	645	505	367	653	917	1112	342	218	243	204 Glycosyl transferase
COG4092	1	0	0	0	0	0	1	0	0	1	2	1	0	0	0	1	0	0 glycosyl transferase
COG5597	48	144	154	5	2	2	41	17	14	24	10	24	4	5	8	52	43	24 Glycosyl Transferase
COG0438	123412	124114	82655	237263	241129	241237	224164	239345	231000	194327	214196	233630	245467	234674	249737	122035	121664	106220 Glycosyl transferase (Group 1
COG4745	607	575	190	1438	1743	1889	1090	1332	1303	1186	1211	1486	1775	1532	1884	586	572	503 Glycosyl transferase family protein
COG0122	11887	11738	7274	21783	24558	23928	19175	24509	21694	17578	22183	19919	23502	20559	24569	10686	10973	9884 Glycosylase
COG3774	667	781	1830	731	722	751	771	600	651	796	794	844	630	786	764	752	811	611 glycosyltransferase
COG5017	96	157	168	265	79	122	606	175	188	243	143	437	90	168	122	70	64	56 glycosyltransferase
COG4671	430	636	444	1350	528	583	1254	575	952	982	715	1332	913	1517	648	467	412	321 Glycosyltransferase 28 domain protein
COG3459	1880	1281	630	1588	1604	1766	1602	1645	1609	2122	1717	2076	1505	2128	1818	1179	1274	1152 Glycosyltransferase 36
COG5020	0	0	0	0	0	0	0	0	0	0	0	0	0	0	0	0	0	0 glycosyltransferase family 15 protein
COG0751	4988	4348	3967	8759	9576	9051	9406	10258	9682	8358	8663	9799	9277	8230	9900	5297	5033	4190 Glycyl-tRNA synthetase beta subunit
COG0346	31623	31970	30975	40629	43584	43097	42196	44695	42384	39965	42240	42789	42220	39611	44799	30813	29849	27516 glyoxalase bleomycin resistance protein dioxygenase
COG2514	15267	16685	9049	20548	23795	22799	18842	23106	20267	17355	21104	18989	22246	19174	23751	11995	11605	12694 glyoxalase bleomycin resistance protein dioxygenase
COG2764	17334	18713	13768	21489	26245	25327	24612	25819	24326	21074	22521	24982	24285	21553	27066	18435	17474	16550 glyoxalase bleomycin resistance protein dioxygenase
COG3324	22571	24135	13384	30775	38930	36682	31771	37490	33733	27447	30053	33132	35282	29330	39935	19238	18799	18538 glyoxalase bleomycin resistance protein dioxygenase
COG3565	852	821	1545	1369	422	537	2048	1013	1776	1153	1247	1706	946	1521	680	765	575	570 glyoxalase bleomycin resistance protein dioxygenase
COG3607	5509	5682	4618	2526	2338	2528	4459	2807	3290	4385	3611	4006	2302	2631	2730	5228	5431	5430 glyoxalase bleomycin resistance protein dioxygenase
COG3960	1860	2934	2601	229	188	147	1014	474	498	781	845	619	183	226	185	3091	2477	3077 glyoxylate carboligase
COG1802	40924	54180	61331	24267	28871	27569	32983	30534	33678	27962	29517	31810	26749	22952	29491	45652	39052	45488 GntR Family Transcriptional Regulator
COG2186	31384	42772	41959	13814	16006	15566	19720	17109	19338	16671	16602	18222	14864	13527	16450	36888	32306	39440 GntR family transcriptional regulator
COG2188	23997	26917	24506	13870	16784	15793	19422	17294	19674	16730	16668	18224	14629	13663	16406	24265	21551	24299 GntR family transcriptional regulator
COG5120	0	0	0	0	0	0	0	0	0	0	0	0	0	0	0	0	0	0 golgi transport
COG4422	491	497	215	1656	843	955	1602	904	1307	1495	1277	1804	1330	1965	1261	729	614	527 gp37gp68 family
COG1584	435	552	1473	352	420	431	385	306	364	280	310	398	356	261	479	467	482	541 gpr1 fun34 YaaH family protein
COG3628	2939	3157	4820	5235	6387	5233	5425	6211	5815	4998	5060	5725	6020	5008	6377	3850	3526	3151 GPW Gp25 family protein
COG4775	11249	11924	9364	26131	30563	28890	28533	30971	28256	25230	26420	29874	28843	26437	31969	12376	12662	8668 Gram-negative-bacterium-type cell outer membrane assembly
COG4398	300	263	77	1521	647	889	1549	896	1225	1368	1259	1608	1080	1669	930	371	294	246 growth
COG2266	14	9	1	6	4	7	6	4	10	4	3	4	5	5	5	10	3	21 GTP adenosylcobinamide-phosphate
COG5258	123	74	20	5112	4488	4889	1841	4543	3356	2200	5445	1885	4696	4422	3924	93	77	38 GTP binding protein
COG0302	7879	8621	7446	11021	11578	10992	11506	11726	11506	10482	10378	11976	11793	11008	11977	9405	8519	8042 GTP cyclohydrolase I
COG2262	8224	8240	6030	16516	17133	17217	13736	17015	15426	12949	16434	14458	16890	15589	17193	8017	7758	7124 GTPase that associates with the 50S ribosomal subunit and may have a role during protein synthesis or ribosome biogenesis (By similarity)
COG1160	9366	8568	6099	12415	12559	12343	13487	13309	13449	11823	11768	14117	12573	12272	13184	8345	8173	7512 GTPase that plays an essential role in the late steps of ribosome biogenesis (By similarity)
COG0012	8003	8040	5959	21080	20954	21430	15247	21310	18450	14857	21603	16014	21001	19594	20436	7808	7453	6836 gtp-binding protein
COG1100	596	406	190	7808	4913	5468	4194	5315	5145	3577	6551	3984	6463	7491	4572	452	531	281 GTP-binding Protein
COG1163	190	148	45	5245	4668	5096	1938	4650	3477	2349	5569	2028	4852	4596	4100	165	142	196 GTP-Binding protein
COG1306	1073	461	106	414	477	529	509	418	510	659	472	469	487	488	566	442	622	546 GTP-Binding protein
COG3596	195	148	60	1202	360	463	1003	396	760	638	475	961	764	1280	449	157	184	124 gtp-binding protein
COG5339	485	1015	2800	184	193	206	183	230	233	316	229	236	182	151	227	1889	1582	1632 GTP-Binding protein
COG1217	7507	7670	5853	10164	11235	10786	11065	11648	11156	9935	10086	11575	10854	9925	11721	7400	7073	6591 gtp-binding protein typa
COG2246	9147	9937	8153	14912	15640	15418	12148	15443	13687	11430	14923	12607	15230	14329	15191	8509	7954	8327 Gtra family
COG5422	209	383	71	421	432	537	333	381	463	394	377	481	392	451	572	147	253	159 guanine nucleotide exchange factor
COG2114	11672	13303	6908	33131	29745	29616	26148	28931	28454	24008	27571	28434	32672	33266	30531	11648	10604	9253 Guanylate Cyclase
COG4021	22	16	6	32	38	33	122	41	49	76	107	86	43	60	45	25	11	23 guanylyltransferase
COG3735	1619	1886	1971	1598	1412	1407	3467	2225	2316	3168	2966	3059	1322	1891	1771	1835	1646	1388 gumn family
COG4274	3714	5334	2634	8282	8037	8921	4228	7558	6079	4259	8016	4479	8185	7640	7505	3136	2567	3903 GYD family
COG3277	123	73	20	5112	4486	4890	1841	4544	3356	2200	5446	1879	4697	4422	3922	93	77	37 h aca RNA-protein complex component gar1
COG1563	142	82	57	937	112	139	911	241	589	468	332	778	545	1016	164	76	106	66 H antiporter subunit B

COG3235	343	671	1415	250	190	243	690	442	468	558	563	498	216	216	266	1710	1404	1410	Membrane
COG3238	4057	4955	3523	4365	6241	5040	4701	5987	5184	4601	4758	5170	5369	4056	6152	4705	4300	4336	membrAne
COG3274	2579	3572	5312	681	455	459	2403	868	1252	1040	1137	1584	462	810	579	2243	2006	2747	Membrane
COG3304	2171	3425	4022	383	500	424	498	409	530	754	483	680	353	502	508	3244	2677	3455	membrAne
COG3305	2810	2218	684	6985	9015	8603	6643	8817	7711	5791	6642	7259	8076	6346	9413	2274	2443	1557	Membrane
COG3308	778	895	1516	386	177	182	1532	747	1172	1049	1155	1122	209	286	258	2004	1576	1507	membrAne
COG3333	9826	13571	9224	1579	1655	1555	3283	2162	3215	3128	3021	3246	1502	1632	1740	9273	8141	11201	membrane
COG3371	1449	2185	1101	5128	4518	4907	1909	4573	3431	2241	5491	1928	4711	4444	3950	1189	979	1641	Membrane
COG3431	123	76	42	1726	32	123	1632	315	1016	713	479	1336	949	1882	153	57	152	48	Membrane
COG3463	591	430	183	1731	1088	1128	1645	874	1294	903	853	1475	1425	1716	1109	469	536	400	Membrane
COG3503	4683	5378	3438	6738	8657	7926	6673	8502	7316	6438	6898	7367	7783	6457	8867	5561	4965	4767	Membrane
COG3528	163	439	57	443	426	552	1007	685	610	847	807	938	416	588	603	451	357	218	Membrane
COG3556	281	648	1412	67	17	30	531	254	256	366	442	270	48	50	39	1686	1370	1312	Membrane
COG3601	922	351	97	5210	4599	5023	1920	4608	3443	2614	5529	1991	4785	4528	4023	347	575	547	Membrane
COG3610	925	344	87	84	76	88	181	111	125	524	226	178	115	245	133	353	574	557	Membrane
COG3619	4283	4545	4962	7799	9730	9365	8932	10024	8896	7610	8389	8800	8851	7396	10243	5204	5248	3834	membrAne
COG3637	5879	6709	5473	9286	11393	10250	10383	11699	10337	10062	10155	11418	9899	8730	11595	8210	6317	5561	Membrane
COG3641	742	212	38	24	22	23	25	22	24	320	26	25	17	26	18	210	396	335	Membrane
COG3647	640	411	138	780	590	681	1063	797	1213	893	995	1155	579	822	702	401	362	448	Membrane
COG3652	6099	5739	3719	12220	15797	14035	14772	16170	14671	12199	13141	14686	14022	11418	16265	6681	6596	4689	Membrane
COG3671	487	433	184	641	503	498	1523	887	924	1430	1515	1174	438	596	659	598	437	495	membrane
COG3686	991	1558	2944	771	512	485	1688	904	1329	1489	1225	1642	443	629	661	3713	2937	2879	Membrane
COG3739	2665	2839	1383	292	285	291	555	373	448	803	437	458	250	308	380	1762	1696	2428	Membrane
COG3748	1204	1562	1908	1045	1027	1056	2094	1383	1376	2093	1912	1790	945	926	1316	2841	2298	2252	Membrane
COG3759	688	654	670	367	254	258	576	412	521	612	573	693	242	357	354	544	592	489	Membrane
COG3766	2301	1605	1754	345	342	308	513	369	413	1337	446	520	253	332	398	2596	2652	2566	Membrane
COG3771	203	370	1395	0	1	0	2	3	1	9	4	3	0	5	0	223	205	292	Membrane
COG3776	386	920	2765	0	1	0	6	3	5	143	43	34	0	3	0	1791	1513	1572	Membrane
COG3817	425	575	1471	200	131	158	629	337	376	523	563	441	136	160	168	480	399	488	Membrane
COG3819	425	575	1471	200	131	158	629	337	376	523	563	441	136	160	168	480	399	488	Membrane
COG3821	517	1092	1774	488	451	473	656	565	567	937	800	761	375	430	554	1993	1583	1557	Membrane
COG3924	955	602	1450	12	14	14	20	14	18	322	20	19	7	13	11	430	648	743	Membrane
COG3949	2389	763	154	44	48	54	65	43	67	982	70	78	34	42	44	732	1361	1290	Membrane
COG4129	7339	8157	4194	1011	1196	1222	1251	941	1303	1440	978	1195	1125	1025	1258	4703	4605	6648	Membrane
COG4194	21	28	24	9	4	6	92	18	19	31	78	38	9	12	10	22	73	132	Membrane
COG4241	988	393	106	952	123	188	891	217	590	768	323	775	554	1044	173	370	664	606	membrAne
COG4244	3310	2750	1028	8762	9208	8548	8551	9381	8860	7276	7740	9197	9161	8966	9512	2733	2919	1863	membrane
COG4258	991	1111	1860	896	779	869	1452	1008	1448	1256	1389	1436	741	678	944	2230	1944	1705	Membrane
COG4269	230	322	223	217	234	213	333	258	219	406	311	354	205	229	250	317	176	298	Membrane
COG4272	2382	1882	797	10965	12252	11974	7844	12164	9777	7077	11217	7839	11676	9820	11736	1929	2389	1157	Membrane
COG4291	1421	1898	3318	807	472	434	2515	1153	1222	1606	1441	1813	452	734	572	2709	2206	2176	membrAne
COG4323	1282	1015	1792	389	311	328	1067	655	722	1168	833	794	307	372	441	2035	2016	1797	Membrane
COG4325	2473	2935	1323	557	649	654	481	496	562	963	595	728	648	743	751	1749	1646	2227	Membrane
COG4330	320	269	369	1151	322	388	1092	390	775	682	486	1020	791	1144	475	258	457	242	Membrane
COG4347	892	318	70	17	17	18	25	14	24	332	29	26	11	16	18	306	554	547	membrane
COG4377	5	3	3	3	3	2	13	9	5	11	4	8	1	2	2	6	4	10	Membrane
COG4425	125	140	72	20	38	44	52	30	42	66	31	72	27	38	32	123	108	79	Membrane
COG4452	399	706	1437	378	176	230	993	489	550	895	805	866	273	482	366	1862	1521	1359	membrAne
COG4485	989	1100	249	2721	3285	3509	2404	2898	2506	2132	2310	2716	3186	2988	3185	1077	1066	633	Membrane
COG4648	502	770	1488	326	187	223	735	455	509	752	742	666	201	240	250	1897	1521	1460	Membrane
COG4684	25	10	6	29	25	29	19	17	26	33	18	32	25	27	26	27	14	14	membrAne
COG4708	184	143	66	5198	4588	5014	1904	4598	3430	2289	5513	1977	4778	4522	4013	152	197	222	Membrane
COG4709	441	304	157	244	158	134	542	315	426	463	400	467	123	184	193	376	389	410	Membrane
COG4713	5	3	1	4	6	4	11	7	8	9	21	24	6	9	2	8	4	11	membrAne
COG4760	2155	2911	1462	703	808	884	895	913	1070	829	808	1197	652	850	948	1528	1471	1977	Membrane
COG4836	901	320	70	21	20	22	30	17	31	331	30	25	14	18	19	308	551	545	membrane
COG4852	1547	2474	1331	203	169	160	439	263	299	546	391	405	133	188	228	1459	1116	1827	membrane
COG4872	297	785	1542	693	45	62	345	95	447	403	248	425	489	879	82	1690	1445	1415	Membrane
COG4907	6013	7039	5858	7805	9996	9423	7406	9376	8238	7149	7412	8172	9243	7540	10001	7517	7487	6660	membrane
COG4920	0	0	0	0	0	0	0	0	0	0	0	0	0	0	0	0	0	0	Membrane
COG4929	488	406	269	792	136	127	855	337	993	485	511	726	560	954	144	294	336	377	Membrane
COG4984	160	106	62	87	75	65	233	111	80	210	213	146	61	83	57	176	148	121	Membrane
COG5006	18971	21176	19038	19126	19468	19599	19724	20609	20707	17725	21058	19097	18840	17953	19587	17981	17022	18482	membrane
COG5305	1568	1670	1785	7417	3724	4422	4288	3431	5196	3513	3446	4822	6293	8543	4120	2642	2625	2158	Membrane
COG5373	16874	19561	13648	20328	24540	23724	22215	23843	21709	20398	20829	23060	22917	19678	25851	18731	16676	18384	Membrane
COG5393	1278	1687	4322	641	471	601	1847	1077	1343	1432	1609	1293	524	775	699	2532	2115	2156	Membrane
COG5413	60	34	20	859	12	59	813	155	506	357	239	667	472	938	75	27	74	22	Membrane
COG5416	2780	2713	1252	443	304	326	1528	855	1138	1137	1234	1119	286	492	312	1791	1681	2224	Membrane
COG5478	2961	3004	1930	6437	8471	7670	6698	8240	7061	5742	6349	6951	7553	6102	8517	2658	2975	1735	Membrane
COG5486	2392	3401	2719	1394	1587	1639	1568	1397	1561	1195	1257	1575	1475	1248	1591	3353	2876	3402	Membrane
COG5488	632	630	379	596	453	403	1527	899	939	1269	1336	1257	393	503	542	751	459	408	Membrane
[illegible]																			

COG0216	7777	7911	5921	10870	12037	11660	11608	12254	11806	10531	10718	12371	11615	10767	12616	7662	7343	6802	Peptide chain release factor 1 directs the termination of translation in response to the peptide chain termination codons UAG and UAA (By similarity)
COG1186	10636	11446	10340	14314	15565	15368	16412	16067	15941	14953	15001	17116	15077	14252	16569	11919	11071	10106	Peptide chain release factor 2 directs the termination of translation in response to the peptide chain termination codons UGA and UAA (By similarity)
COG4166	7503	9530	15367	5601	6452	6830	5489	5484	5791	6243	6001	6664	6170	6253	7206	10643	10187	10473	peptide transport
COG4167	203	370	1395	0	1	0	2	3	1	9	4	3	0	5	0	223	205	292	peptide transport system atp-binding protein
COG4171	204	370	1395	1	1	0	2	3	1	9	4	3	0	5	0	223	205	292	peptide transport system permease protein
COG3409	2566	1676	742	4249	2527	2577	3112	2124	3426	2727	2367	3665	3534	4666	2592	1549	1873	1563	Peptidoglycan-binding domain 1 protein
COG5526	74	80	42	680	33	49	338	85	429	285	199	379	465	858	70	63	111	59	Peptidoglycan-binding domain 1 protein
COG4254	1245	1472	1798	1239	990	1127	2272	1604	1925	1997	1960	2007	995	1142	1156	2548	2011	1983	Peptidoglycan-binding LysM
COG1047	2653	3245	6310	8320	6595	7326	6976	8047	7496	6816	10119	6745	7523	8083	6566	5492	4579	4252	peptidylprolyl cistrans isomerase
COG0545	10606	12912	11637	13468	14620	14471	16708	15568	14804	15287	15162	17118	14153	14601	16014	13576	13088	11979	Peptidyl-prolyl cis-trans isomerase
COG0760	16601	16152	16836	28467	31673	30222	31344	33162	31703	27929	29087	32214	30953	27710	32810	20905	20196	16454	peptidyl-prolyl cis-trans isomerase
COG0652	18666	18647	12292	35818	38715	38306	32995	38882	35330	30347	34379	34624	37647	34452	39756	18213	17654	15674	peptidyl-prolyl cis-trans isomerase activity
COG4531	1278	1373	3043	111	113	116	220	171	139	622	192	254	100	119	123	2162	2033	1979	periplasmic
COG0614	18609	17835	22857	27263	25637	26659	21129	24895	23136	21244	26511	21805	26219	25938	25520	16553	17565	17119	Periplasmic binding protein
COG4558	1176	1831	4704	383	417	337	1439	770	1118	981	981	1129	379	535	483	2419	2171	2146	periplasmic binding protein
COG4607	509	354	260	36	26	22	77	37	61	60	55	76	19	52	22	257	344	343	Periplasmic binding protein
COG1879	31438	42079	59871	26368	32567	31167	31813	33260	30154	27719	29540	29747	30104	25647	33529	35807	30835	34587	Periplasmic binding protein LacI transcriptional regulator
COG3420	3456	3442	3644	6834	8908	8189	6399	8380	7310	6077	6561	7118	8078	6587	8991	4301	4122	3583	Periplasmic copper-binding protein
COG3416	954	1517	3139	357	360	318	500	485	559	501	366	613	308	332	410	2201	1782	1850	Periplasmic ligand-binding sensor protein
COG4459	63	87	37	85	107	87	78	87	74	129	88	136	81	84	108	149	63	80	periplasmic nitrate reductase
COG2845	548	667	413	382	387	366	444	416	407	542	453	603	312	358	425	524	360	398	Periplasmic Protein
COG2859	127	148	335	365	393	384	245	277	304	386	295	416	336	438	423	139	261	113	Periplasmic Protein
COG2991	16	10	6	24	14	22	20	31	22	37	40	32	24	32	24	16	18	13	Periplasmic Protein
COG3439	4609	4714	1948	7601	9636	9061	7687	9494	8751	6639	7533	8164	8790	7365	9795	3715	3364	3320	Periplasmic Protein
COG3470	283	119	32	87	59	40	797	401	735	200	328	425	60	57	64	50	38	32	Periplasmic Protein
COG3656	621	432	176	482	460	479	1006	621	955	765	735	846	410	326	580	537	486	409	Periplasmic Protein
COG3678	3000	3272	4730	7261	8203	7640	6762	8111	7205	5894	6464	7040	7864	6801	8358	4107	3848	3160	Periplasmic Protein
COG3904	507	672	395	518	574	432	622	607	659	753	585	932	428	476	578	824	411	434	Periplasmic protein-like protein
COG3471	333	714	1452	176	107	133	774	384	397	723	724	561	137	182	183	1815	1454	1409	periplasmic secreted protein
COG0803	9474	10334	10712	15969	13486	14709	9359	13193	11998	10133	15437	9609	14762	14938	12684	10887	9837	11044	periplasmic solute binding protein
COG1427	3039	2460	693	7305	9477	8852	7034	8866	7719	6096	6889	7572	8661	6894	9586	2583	2757	1830	periplasmic solute-binding protein
COG2107	2822	2246	634	6856	8889	8276	6712	8476	7302	5763	6543	7085	7949	6383	9048	2392	2572	1664	periplasmic solute-binding protein
COG0833	4961	6483	6954	252	250	225	861	412	563	915	618	580	216	267	295	4803	5159	6016	permease
COG2233	8749	11073	17027	2175	2080	2232	4341	2772	3328	4660	4019	3529	2028	2399	2403	13637	12106	13725	permease
COG4200	483	299	103	303	353	364	375	277	334	339	369	389	346	365	383	353	414	416	permease
COG0609	14599	14716	21800	12532	10466	11356	10663	10752	11261	11536	12780	10971	11195	12327	10598	14254	14736	15108	permease protein
COG4211	332	473	1406	10	8	9	11	10	8	22	35	21	8	10	6	326	327	410	permease protein
COG4605	468	340	223	30	22	16	71	31	52	53	39	69	14	36	14	220	302	329	permease protein
COG4606	468	339	223	30	21	15	71	31	52	49	38	68	13	36	14	219	302	329	permease protein
COG0795	7159	7861	8165	13250	13900	13708	16156	15446	14979	14129	14188	16618	13479	12959	14977	9773	8942	7330	Permease yjgp yjgq family
COG2837	4843	4912	7132	6917	9003	8333	6632	8597	7541	5901	6626	7187	8052	6649	8900	6331	6749	5371	peroxidase
COG0678	1371	1155	543	1841	844	895	3178	1728	2828	2203	2116	2834	1259	1802	1097	1153	827	735	peroxiredoxin
COG5574	0	0	0	0	0	0	0	0	0	0	0	0	0	0	0	0	0	0	Peroxisomal biogenesis factor 10
COG3214	3133	4261	4809	1585	1738	1849	1893	1678	1845	1965	1920	1993	1662	1812	1820	3990	3422	4057	Pfam:DUF1006
COG3721	60	61	26	48	55	48	32	44	40	45	38	53	46	55	55	76	42	37	Pfam:DUF1008
COG3554	180	188	364	246	265	300	346	221	294	211	254	220	323	297	289	147	232	136	Pfam:DUF1089
COG5375	474	636	354	520	514	488	728	642	607	921	653	862	417	474	592	614	454	462	Pfam:DUF1239
COG3360	2481	3600	2727	1465	1393	1547	2276	1752	2239	1923	2003	2203	1421	1607	1715	3617	2969	3594	Pfam:DUF1458
COG1810	0	0	0	0	0	0	0	0	0	2	0	3	0	0	0	0	0	0	Pfam:DUF166
COG5465	486	636	353	1177	521	512	912	646	968	1016	789	1096	868	1301	622	607	477	440	Pfam:DUF1790
COG3462	3097	4550	2288	789	963	963	659	658	792	601	719	798	863	896	931	2501	2149	3461	Pfam:DUF2078
COG3472	98	99	46	117	146	138	90	107	115	161	112	175	115	123	150	167	85	109	Pfam:DUF2081
COG3826	3431	3092	2476	6482	8403	7617	7444	8525	7783	6088	6882	7275	7472	5898	8423	4310	4219	3109	Pfam:DUF2086
COG4519	183	554	1376	0	1	0	4	2	4	135	40	33	0	1	0	1575	1311	1280	Pfam:DUF2131
COG4649	447	616	343	532	517	503	687	642	592	876	673	840	464	580	632	588	422	422	Pfam:DUF2133
COG4655	813	957	1533	291	215	253	1514	773	1129	878	1054	844	244	255	293	1999	1636	1515	Pfam:DUF2134
COG5468	433	599	330	485	485	448	615	561	548	800	594	753	380	434	538	570	397	415	Pfam:DUF2159
COG4832	196	171	55	418	411	475	265	267	322	325	326	333	348	384	475	145	105	116	Pfam:DUF2174
COG3602	2059	3658	4366	200	212	156	516	366	467	620	421	628	134	197	207	3206	2856	3333	Pfam:DUF2241
COG4340	341	239	125	320	174	201	676	416	458	747	804	593	192	286	238	381	261	231	Pfam:DUF2257
COG5368	3903	3141	1298	7070	9137	8449	7247	8932	7805	6678	6995	7724	8249	6918	9373	2751	3310	1966	Pfam:DUF2329
COG2164	374	495	1412	5301	4664	5107	2010	4735	3510	2351	5631	2067	4863	4593	4100	379	350	369	Pfam:DUF369
COG3801	1791	2619	1423	545	570	593	706	490	601	704	558	674	565	610	642	1509	1270	2020	Pfam:DUF419
COG3762	1297	1752	2043	1635	1467	1601	2643	2062	1996	2706	2486	2818	1419	1815	1855	2987	2669	2255	Pfam:DUF477
COG4894	105	53	37	15	23	22	29	20	45	33	29	52	23	32	45	45	102	167	Pfam:DUF567
COG4801	306	100	23	91	45	24	449	263	668	86	166	383	25	20	23	25	15	13	Pfam:DUF583
COG4740	0	0	0	0	0	0	0	0	0	0	0	0	0	0	0	0	0	0	Pfam:DUF785
COG1656	561	405	128	6594	5383	5836	3186	5472	4671	3159	6567	3207	5989	6183	4885	441	444	385	Pfam:DUF82
COG4636	11006	8672	3155	69341	23711	24781	53830	27980	49268	34015	30104	52402	52683	77570	27452	8109	11459	6317	Pfam:DUF820
COG2983	820	1522	3127	501	499	458	631	584	565	961	648	800	390	458	547	2365	1935	1990	Pfam:UPF0153
COG0524	38374	42289	40333	68970	75350	74707	57868	74228	65161	53721	69303	59881	72768	65813	74758	37298	36383	34211	pfkb domain protein
COG0693	15743	17258	15730	20894	21249	21690	19914	21707	20384	17763	20892	19864	21438	20573	21497	16465	16534	16068	PfpI family
COG4683	2456	2165	1904	6505	7845	7141	5613	7486	6655	4845	5638	6006	7400	6176	7755	1987	2117	1466	Phage derived protein Gp49-like (DUF891)
COG5323	324	361	130	399	395	371	482	444	452	612	497	627	305	360	441	374	287	311	phage DNA Packaging protein
COG5614	469	629	1515	268	317	293	301	345	358	407	386	457	251	289	390	572	396	505	Phage head-tail adaptor
COG4653	839	1060	1653	878	956	1069	1105	1107	1112	1360	1170	1429	792	958	1221	1070	750	873	Phage major capsid protein
COG4672	411	739	2809	2	1	0	16	12	5	30	15	12	0	7	1	444	416	603	Phage minor tail protein L
COG2369	532	504	1417	205	108	100	552	369	751	194	363	477	149	236	144	268	232	329	Phage Mu protein F like protein
COG4695	3109	2796	2173	11511	12815	12442	8046	12786	10466	7977	11924	8737	12047	10262	12291	2749	2627	1929	Phage Portal Protein
COG5511	947	740	376	1216	1297	1318	1450	1215	1275	1287	1235	1523	1083	840	1400	930	858	759	Phage portal protein lambda family
COG5362	13	34	18	4	0	2	12	5	5	22	6	14	2	1	0	21	21	13	phage protein
COG5410	354	487	1480	198	165	152	289	304	303	353	358	425	115	147	154	346	336	518	phage protein
COG5449	294	282	121	279	322	243	453	469	497	627	497	701	217	256	329	391	222	248	phage protein
COG5556	423	768	2820	49	3	16	58	28	17	69	127	31	10	36	10	463	434	615	phage protein
COG3646	318	636	1419	35	23	20	261	130	182	199	181	115	24	45	25	1645	1447	1464	Phage regulatory protein Rha (Phage_pRha)
COG1842	5186	5715	5406	6142	4149	4804	5923	4402	5141	4565	4179	5500	5408	6566	4575	5422	5107	5350	Phage shock protein A
COG1983	8947	10372	5985	13589	15178	15015	10494	14584	12451	10101	13628	10919	14447	12734	14803	7156	6938	7803	phage shock protein C
COG4223	327	472	285	408	448	392	484	458	450	656	507	617	334	387	494	452	286	307	phage Tail Protein
COG4385	464	1007	2811	77	68	70	198	109	85	385	220	221	58	73	76	1882	1584	1644	phage Tail Protein
COG5283	498	1009	2791	37	48	23	199	58	67	303	168	174	23	33	35	1874	1572	1617	Phage Tail Tape Measure Protein
COG1783	104	87	33	52	19	17	549	160	125	199	230	274	33	65	38	111	106	72	phage terminase (large subunit)
COG3747	535	865	2804	253	239	312	269	305	288	256	280	331	239	265	305	576	524	686	phage terminase small subunit
COG3311	4657	6050	3966	1629	1446	1488	3757	2551	3660	2804	3215	3534	1367	1737	1702	5225	3897	5352	Phage transcriptional regulator AlpA
COG3600	183	379	55	391	374	430	369	340	424	418	353	417	316	404	478	129	235	139	Phage-Associated Protein
COG5377	418	183	154	236	121	126	821	486	842	350	491	669	117	186	157	175	140	96	phage-type endonuclease
COG5490	795	1149	677	865	950	811	998	957	901	1311	945	1307	761	862	985	1256	734	820	phasin family
COG5141	0	0	0	0	0	0	0	0	0	0	0	0	0	0	0	0	0	0	phd finger
COG4313	3514	4179	6354	6285	8174	7430	6422	8111	7167	5838	6311	6755	7270	5675	8145	5874	5237	4639	Phenol degradation-like protein
COG3479	232	376	1395	6	6	5	18	7	13	21	19	14	7	6	5	225	209	297	Phenolic acid decarboxylase
COG3460	3333	3613	3082	570	605	561	1570	879	1271	1380	1083	1152	583	622	607	3530	3182	3639	phenylacetate catabolic process
COG1541	8940	9811	6040	12477	12355	13340	10691	12138	11994	10326	13574	11423	12553	11572	12681	9255	8570	9028	Phenylacetate-CoA ligase
COG3327	3247	3565	2833	1026	1169	1261	1266	1075	1497	1533	1191	1361	1180	1357	1331	3517	3078	3688	phenylacetic acid degradation operon negative regulatory protein
COG0072	8027	7990	5954	15936	16438	16503	13388	16734	15069	12615	16098	14118	16242	15040	16434	7724	8119	6871	phenylalanyl-tRNA synthetase (beta subunit)
COG1702	7705	8075	7235	10227	11235	10845	11178	11633	11136	10065	10167	11548	10852	10069	11824	7665	7357	6960	PhoH family
COG1875	3916	4081	3082	1535	1329	1574	2850	2000	2508	2774	2560	2608	1367	1529	1760	4139	3682	4185	PhoH family
COG4287	238	176	69	396	382	515	589	519	459	675	633	590	395	473	541	241	200	135	PhoPQ-activated pathogenicity-related protein
COG0394	17999	20785	14595	17000	17489	17869	19398	18343	18617	16534	16834	19240	17302	17006	18658	17019	16604	17747	PHOsphatase
COG0631	14448	14556	10905	23178	24479	23931	21680	22937	22505	20181	20812	23518	24631	22980	25937	14561	14526	13335	Phosphatase
COG0671	11214	11817	10667	10477	11983	11348	12238	12036	11419	11403	10982	12358	11429	11068	12335	9969	10171	9746	PHOsphatase
COG4359	1401	1063	1507	1039	398	416	679	435	899	901	552	853	840	1226	425	2042	2057	2018	phosphatase
COG5329	0	0	0	0	0	0	0	0	0	0	0	0	0	0	0	0	0	0	Phosphatase
COG0226	14032	13583	11690	29930	27938	28149	28133	28892	27990	23859	27017	28303	29273	29051	29291	15091	14793	13083	phosphate abc transporter
COG0573	8060	7461	4626	16843	16445	16677	14678	16835	15546	12675	16316	14676	16556	15874	16607	6174	6152	5781	phosphate abc transporter
COG0581	8305	7934	6034	16979	16659	16853	14772	16962	15641	12811	16411	14835	16904	16031	16774	6470	6417	6145	phosphate abc transporter
COG4985	183	554	1376	0	1	0	4	2	4	135	40	33	0	1	0	1575	1311	1280	phosphate ABC transporter (Permease
COG0280	5925	5965	4895	4522	4539	5195	5630	5350	5524	5695	5341	5773	4299	4538	5179	5955	5331	5747	phosphate acetyltransferase
COG1392	5387	5835	2630	13692	14963	14887	10971	14834	12510	10178	14348	11189	14356	12731	14809	4862	4866	4350	Phosphate transport regulator
COG3746	1019	1314	1649	1424	1370	1562	1619	1656	1474	2130	1713	2081	1529	1813	1663	2534	2059	2095	Phosphate-Selective Porin O and P
COG3223	422	953	2851	66	17	16	110	48	48	247	135	103	20	54	38	1817	1629	1738	phosphate-starvation-inducible protein PsiE
COG0170	331	299	91	6367	4939	5389	3435	5141	4255	3022	6157	3162	5740	5786	4415	270	318	229	phosphatidate Cytidylyltransferase
COG0575	7868	7962	5917	15936	16442	16472	13398	16751	15131	12663	16115	14198	16250	15132	16467	7712	7389	6810	phosphatidate Cytidylyltransferase
COG4589	1255	1158	1670	473	456	516	611	442	460	1111	800	724	479	462	637	2379	2044	1883	phosphatidate Cytidylyltransferase
COG1881	3402	3782	6576	3476	3568	3772	5307	3987	4303	4119	4522	4939	3360	3690	3814	4449	3826	3820	phosphatidylethanolamine-binding protein
COG1267	5428	4036	3608	7840	9551	9148	8322	10075	9139	8038	8335	8967	8871	7548	9916	4966	5082	4280	phosphatidylglycerophosphatase a
COG5542	2150	2670	1348	6576	5359	5867	2665	5127	4440	2880	6173	2870	6011	6149	4845	1531	1398	2017	Phosphatidylinositol glycan anchor biosynthesis class V
COG5032	0	0	0	0	0	0	0	0	0	0	0	0	0	0	0	0	0	0	phosphatidylinositol kinase activity
COG5083	4	6	2	25	32	29	24	21	25	42	23	37	36	45	40	7	4	8	phosphatidylinositol transfer protein
COG5253	0	1	0	0	0	0	0	2	1	5	0	5	0	2	0	1	0	2	phosphatidylinositol-4-phosphate 5-kinase
COG0688	5948	5284	4493	9262	11142	10582	10504	11707	10840	9544	9978	11127	10217	8831	11496	6250	6720	4859	Phosphatidylserine decarboxylase
COG3200	2831	3844	3208	990	992	983	1173	1025	1210	1381	1075	1489	914	1001	1089	3615	3676	3797	phospho-2-dehydro-3-deoxyheptonate aldolase
COG2877	5141	4863	4304	8618	10524	9978	9717	10858	9893	9009	9307	10008	9749	8178	10944	5776	5560	4343	Phospho-2-dehydro-3-deoxyoctonate aldolase
COG3969	0	0	0	0	0	2	0	0	1	1	1	1	0	0	0	2	1	1	phosphoadenosine phosphosulfate reductase
COG5270	0	0	0	0	0	0	0	0	0	0	0	0	0	0	0	0	0	0	phosphoadenosine phosphosulfate reductase
COG1925	10679	11581	9285	4178	4283	4554	5341	4486	5059	5686	4820	5569	4207	4121	4923	9011	8378	10423	phosphocarrier protein (HPr)
COG5212	143	392	326	264	263	241	330	252	291	401	351	464	247	373	366	154	377	119	Phosphodiesterase
COG1866	2667	3184	5070	3659	3333	3677	3201	3255	3163	3233	2944	3453	3768	3777	3560	3828	3802	3344	Phosphoenolpyruvate Carboxylase
COG2352	6236	6843	5392	9240	10335	9709	10281	10583	10127	8641	9250	10405	9842	8940	10601	6860	6307	5786	phosphoenolpyruvate carboxylase activity
COG3605	1531	2049	3299	2776	2868	3281	2275	2963	2613	2293	2370	2596	2693	2589	2975	2900	2544	2357	phosphoenolpyruvate--protein phosphotransferase
COG4186	162	165	334	109	118	103	86	97	81	114	87	122	105	127	117	176	215	125	Phosphoesterase or phosphohydrolase
COG0618	4502	3623	1284	14790	15582	15645	10838	14839	12685	10559	13944	11562	15591	13973	15326	3667	4065	2850	phosphoesterase RecJ domain protein
COG1107	211	371	1393	18	19	13	16	13	12	29	23	19	12	18	26	225	210	297	phosphoesterase RecJ domain protein
COG0033	3424	4524	4599	3131	2997	3468	3324	3431	3166	2845	2972	3551	2854	3010	3243	4278	3670	4305	phosphoglucomutase
COG0126	7936	8142	6100	15972	16452	16528	13498	16787	15124	12740	16213	14193	16258	15073	16471	7829	7441	6849	phosphoglycerate kinase
COG0406	22847	25391	19872	14930	13505	14637	16712	13031	15131	16180	14504	17055	14248	15883	14640	21261	20196	22720	Phosphoglycerate mutase
COG4192	0	0	0	0	0	0	0	0	0	0	0	0	0	0	0	0	0	0	phosphoglycerate transport regulatory protein pgtB
COG0205	9076	8446	6161	13485	14411	14349	12731	13522	13028	12269	12073	14096	14511	13743	15302	7261	7654	6713	phosphohexokinase
COG2062	6390	7281	5749	9701	10657	10213	10522	11056	10201	9435	9716	10892	10259	9521	11209	7024	6488	6089	phosphohistidine phosphatase activity
COG4341	804	1426	2829	417	389	365	1119	664	1032	1168	1022	990	408	526	500	3477	2852	2730	Phosphohydrolase
COG3957	4882	5651	4785	8632	9018	8437	8596	9047	8650	6628	7177	8782	9120	8458	9066	5443	5124	4936	Phosphoketolase
COG2829	831	979	1597	537	372	397	1224	770	1187	973	1097	1125	411	539	525	726	663	566	Phospholipase
COG3511	10602	9137	5588	23347	30804	27651	25880	31060	27355	21337	24249	25449	27539	21564	31135	11325	11191	7754	phospholipase C
COG0400	9714	11382	10229	10432	11506	10972	11800	12176	11837	10668	11037	12445	11227	10444	11965	10864	9928	9948	phospholipase carboxylesterase
COG4099	2168	2425	2631	4709	5430	6121	4236	4935	4496	4037	4155	4973	5310	4971	5895	2027	2387	1709	phospholipase Carboxylesterase
COG3963	1609	2244	3756	1404	1561	1646	1695	1726	1741	1874	1603	2163	1315	1358	1810	1728	1531	1650	Phospholipid N-methyltransferase
COG0351	9491	10185	6925	15902	16449	16431	14244	16835	15542	12895	16692	14313	16180	15006	16356	9096	8446	8632	phosphomethylpyrimidine kinase
COG1992	123	73	20	5111	4486	4888	1841	4543	3356	2200	5445	1879	4696	4420	3921	93	77	37	Phosphomethylpyrimidine kinase
COG3890	0	0	0	0	0	0	1	2	0	2	0	2	0	0	2	0	0	0	Phosphomevalonate kinase
COG0472	13425	13846	11381	20808	19868	20238	19162	20754	20228	17463	20571	19865	20420	20418	20290	13423	12937	12338	phospho-N-acetylmuramoyl-pentapeptide-transferase activity
COG3639	5146	3887	5250	7067	5676	6018	5071	6318	6202	5873	8477	4742	6215	6526	5245	4671	4381	4150	Phosphonate ABC transporter
COG3624	470	795	1651	223	214	197	525	334	361	479	420	455	183	224	245	638	441	505	Phosphonate C-P lyase system protein PhnG
COG3625	470	795	1651	224	215	197	525	334	361	479	420	455	184	225	245	638	441	505	phosphonate C-P lyase system protein PhnH
COG4107	498	843	1672	253	216	212	590	370	401	599	617	527	206	251	259	721	483	521	phosphonate C-P lyase system protein PhnK
COG4778	470	795	1651	224	215	197	525	334	360	479	420	455	184	225	245	638	441	505	phosphonate c-p lyase system protein phnl
COG3627	470	795	1651	224	215	197	525	334	361	479	420	455	184	225	245	638	441	505	phosphonate metabolism
COG3626	470	795	1651	224	215	197	525	334	361	479	420	455	184	225	245	638	441	505	phosphonate metabolism protein
COG3454	797	1220	1906	688	776	722	1056	808	842	1039	1002	1114	667	714	832	1006	717	759	phosphonate metabolism protein PhnM
COG0452	8088	8203	6015	11497	12815	12600	12008	12852	12314	11112	11256	12863	12520	11612	13561	7912	7612	7130	Phosphopantothenoylcysteine decarboxylase
COG0120	2192	2330	3395	7918	6388	6819	5773	7092	6706	5339	8573	5828	6877	7521	6192	3318	2772	2585	phosphoriboisomerase A
COG0462	9128	8850	6149	16423	16981	17036	14117	17263	15661	13880	17037	14965	16757	15803	17135	8496	8363	7624	phosphoribosyl pyrophosphate synthase
COG0026	7318	7482	6080	9394	10531	9994	10455	10852	10425	9046	9261	10645	9929	9048	10871	7160	7001	6286	phosphoribosylaminoimidazole carboxylase atpase subunit
COG0150	7723	7855	5910	10745	11920	11572	11343	11943	11447	10113	10381	11849	11496	10553	12432	7539	7266	6731	phosphoribosylaminoimidazole synthetase
COG0139	7953	8455	7276	15808	16330	16435	13158	16465	14776	12502	15900	13768	16156	14951	16311	9219	8641	8020	Phosphoribosyl-amp cyclohydrolase
COG0140	4062	4453	3350	1882	1624	1717	3203	2138	2475	3027	2521	3091	1395	1789	1759	4445	3723	4320	phosphoribosyl-ATP pyrophosphatase
COG0106	7800	7919	5899	15817	16329	16418	13179	16475	14788	12376	15883	13762	16166	14960	16307	7655	7347	6780	phosphoribosylformimino-5-aminoimidazole carboxamide ribotide isomerase
COG0046	7808	7949	5923	11062	12228	12016	11734	12380	11849	10569	10846	12330	11779	10921	12899	7659	7334	6785	phosphoribosylformylglycinamidine synthase
COG0047	6624	6282	2927	9981	11372	10970	9419	10892	10026	8737	8976	10306	10935	9834	11803	5070	5178	4849	phosphoribosylformylglycinamidine synthase
COG1828	5955	5668	2490	8441	9772	9195	7958	9236	8584	7240	7464	8584	9262	8135	10080	4456	4522	4428	phosphoribosylformylglycinamidine synthase
COG0299	7875	8493	7329	10757	11945	11600	11468	11984	11189	10463	10655	11846	11516	10594	12455	9338	8730	8092	phosphoribosylglycinamide formyltransferase
COG0151	7939	8032	5960	11289	12493	12209	11743	12345	11915	10579	10877	12389	12039	11142	13074	7742	7414	6856	Phosphoribosylglycinamide synthetase
COG1926	4515	6000	3949	8377	7146	7826	5473	7461	6642	5177	8396	5730	7731	7979	6940	5092	5077	5593	phosphoribosyltransferase
COG2236	1190	897	380	6743	5196	5770	4539	5932	5411	3922	7233	4117	5821	6150	4850	895	712	660	phosphoribosyltransferase
COG3954	566	715	1503	519	417	477	780	744	599	827	800	965	399	588	521	748	513	637	phosphoribulokinase (EC 2.7.1.19)
COG0689	7655	7526	5597	15682	16143	16292	12549	16330	14661	11972	15570	13357	15949	14618	16085	7322	6886	6694	Phosphorolytic exoribonuclease that removes nucleotide residues following the -CCA terminus of tRNA and adds nucleotides to the ends of RNA molecules by using nu [illegible]
COG5290	3	1	0	2	2	1	2	2	0	4	2	9	0	6	0	3	2	10	phosphorylase kinase regulator activity
COG0125	12079	12094	7460	28261	30290	30266	21224	29792	25872	20386	28017	22845	29359	25979	29984	11079	10514	10272	Phosphorylation of dTMP to form dTDP in both de novo and salvage pathways of dTTP synthesis (By similarity)
COG1340	144	116	40	5132	4516	4924	1860	4564	3376	2243	5462	1914	4720	4442	3948	125	106	66	phosphoserine phosphatase
COG0560	12577	15043	16556	15050	18562	17163	17271	18941	17934	15673	16507	17938	17001	14420	19060	16040	14427	14370	phosphoserine phosphatase activity
COG1809	704	408	85	423	476	335	1305	755	1190	801	730	1206	393	600	480	396	446	256	phosphosulfolactate synthase activity
COG1015	1826	1379	1855	717	744	754	888	721	811	1101	745	972	670	590	795	1291	1377	1474	Phosphotransfer between the C1 and C5 carbon atoms of pentose (By similarity)
COG3178	1753	1916	2292	2001	1732	1842	2991	2346	2684	2761	2702	3075	1592	1767	2051	3061	2597	2422	Phosphotransferase
COG5050	100	96	33	105	102	131	90	110	90	175	95	93	89	76	89	90	85	95	phosphotransferase 1
COG1735	1087	1277	2097	606	747	770	436	455	493	480	428	515	622	629	623	866	846	780	phosphotriesterase-related protein
COG0434	642	821	1660	6461	5014	5653	3176	5309	4470	3135	6259	3197	5676	5942	4786	565	542	504	photosystem i assembly btpa
COG5285	5877	6710	9020	7916	7814	8412	9448	7891	8379	8685	8094	9888	8237	8620	8794	9663	9474	8054	phytanoyl-CoA dioxygenase
COG4247	403	323	385	421	149	164	1509	478	455	802	651	894	154	323	222	423	536	282	Phytase
COG1233	18540	16412	9948	38004	42208	39840	33600	39925	36288	31001	34335	35575	41721	37165	42325	16907	17220	14051	phytoene
COG1562	9582	7871	4267	22897	25156	24497	21511	25576	23068	18853	23410	21778	24101	21197	25352	7951	7721	5778	phytoene synthase
COG4374	1840	1469	443	4266	6026	4982	4330	5694	4883	3912	4293	4507	5301	3835	5994	1437	1517	948	PilT protein
COG1569	3742	2948	840	11314	11427	11511	11168	11792	10800	8818	9850	11035	11538	11079	11905	3129	3234	2000	PilT protein domain protein
COG3744	5104	4264	1508	14899	16339	15217	14017	16196	14978	11201	12569	14030	16197	14162	16764	4013	4414	2686	PilT protein domain protein
COG4956	2030	1270	376	4246	3790	4498	3263	3502	3456	2973	2975	3575	4125	4364	4194	1336	1664	1324	PilT protein domain protein
COG5573	2612	2111	618	7130	8487	7850	6633	8302	7335	5449	6300	6991	8211	6764	8535	2125	2351	1463	PilT protein domain protein
COG5611	332	548	504	462	284	396	589	472	523	603	555	643	317	399	448	300	240	188	PilT protein domain protein
COG3168	752	957	1555	912	623	766	1269	1078	1017	1417	1480	1449	652	861	728	2192	1795	1750	Pilus assembly protein
COG3170	1852	2040	3165	1528	1217	1387	2686	1909	2288	2378	2456	2617	1180	1338	1485	4381	3637	3284	Pilus assembly protein
COG4726	1063	1152	1648	1078	777	890	1719	1292	1186	1747	1799	1690	781	1004	912	2519	2059	1836	Pilus assembly protein
COG4963	4799	6416	4238	3305	3629	4004	3881	3771	3892	3183	3527	3676	3694	3374	3833	5475	5244	5832	Pilus assembly protein
COG4966	1404	1498	1766	2284	1712	2073	3665	2646	2576	3319	3310	3574	1925	2320	2190	2835	2296	2194	Pilus assembly protein
COG3167	974	1122	1606	1491	1149	1389	1747	1549	1594	1938	1934	2101	1145	1444	1414	2347	1924	1900	pilus assembly protein pilo
COG3419	948	1096	1597	1377	922	1156	1876	1544	1533	2114	2264	2170	1021	1329	1231	2393	1907	1948	Pilus assembly protein tip-associated adhesin
COG5461	485	681	372	509	532	458	643	569	567	834	617	799	412	472	563	712	455	476	Pilus Caulobacter type biogenesis lipoprotein CpaO
COG5378	26	26	2	14	21	19	25	20	25	15	19	30	27	27	16	11	6	7	PIN domain
COG1741	11126	12369	15795	12137	12739	12185	17400	14901	15200	14909	14863	16624	12409	12238	13650	15117	14475	12925	pirin domain protein
COG3128	3864	3725	3665	7278	8783	8133	8344	9343	8437	7119	7626	8480	7846	6533	8878	4824	4257	3574	PKHD-type hydroxylase
COG3093	5673	5875	6997	10278	11321	10848	11629	12198	11118	9485	10167	11959	11349	9876	11629	6512	5938	5147	Plasmid maintenance system antidote protein
COG3549	4426	4225	3852	9011	10100	9626	9052	10495	9581	7757	8606	9616	9908	8544	10354	5078	4857	3910	Plasmid maintenance system killer
COG3496	2230	2956	1621	1210	1115	1200	1584	1256	1393	1655	1593	1674	1106	1042	1364	2124	1734	2343	plasmid partition ParA protein
COG4691	629	901	1609	418	271	299	943	731	783	591	655	739	224	380	254	1825	1566	1461	Plasmid stability protein
COG4188	1775	1851	2243	4523	2264	2596	4216	2348	3621	3469	2722	4469	3589	5186	2966	2956	2735	2481	Platelet-activating factor acetylhydrolase plasma intracellular isoform II
COG3303	414	379	119	1170	1152	1428	1035	1203	1113	1219	1135	1339	1177	1229	1503	471	464	566	Plays a role in nitrite reduction (By similarity)
COG0704	9915	9341	6134	35431	35916	36214	22450	35729	29663	22580	36238	23680	35688	31950	34037	9146	8750	7847	Plays a role in the regulation of phosphate uptake
COG3143	1356	1587	2993	1105	781	859	2202	1550	1828	1730	1937	1970	761	1021	907	2660	2099	2100	Plays an important role in bacterial chemotaxis signal transduction pathway by accelerating the dephosphorylation of phosphorylated CheY (CheY-P) (By similarity)
COG0104	8202	8280	6363	15937	16473	16537	13340	16604	14939	12532	16036	13948	16275	15082	16498	7874	7640	6943	Plays an important role in the de novo pathway of purine nucleotide biosynthesis
COG4028	0	0	0	0	0	0	0	0	0	0	0	0	0	0	0	0	0	0	P-loop ATPase GTPase-like protein
COG4928	1535	2396	1291	276	295	251	397	303	354	499	346	471	259	384	339	1355	1102	1797	p-loop domain protein
COG5186	12	4	3	3	4	2	4	3	6	6	5	14	2	7	1	5	4	11	polyA polymerase
COG0687	11185	16874	29900	13529	11469	12591	12749	12783	12578	12294	14632	12113	12280	13027	11662	24011	20866	20680	polyamine binding
COG4891	124	91	24	114	145	114	673	288	244	376	353	440	103	172	167	82	106	78	polyketide cyclase dehydrase
COG2326	7095	8512	7541	10239	11386	10909	11703	11782	11093	10490	10570	11911	10864	10079	12108	9375	9156	8076	Polyphosphate kinase 2
COG1086	7350	7552	5827	10043	11163	10694	11244	11577	11329	10127	10305	11331	10731	9872	11485	8614	8342	7518	polysaccharide biosynthesis protein
COG2244	28069	24914	18568	45087	55366	51537	48227	54575	48142	43385	44703	49217	50867	42885	56233	23601	24511	19363	Polysaccharide biosynthesis protein
COG2861	1665	1741	3110	746	806	799	698	675	713	1342	801	921	620	730	864	2585	2322	2340	polysaccharide de-acetylase
COG3524	4370	3481	991	10090	13741	11954	10336	13493	11830	8885	10210	10706	12107	9055	13675	3399	3498	2164	polysaccharide export
COG2327	3236	3471	3045	2094	1105	1290	2804	1412	1755	2216	1582	2432	1470	2259	1374	2359	2466	2903	Polysaccharide pyruvyl transferase
COG5039	116	197	169	288	276	321	192	195	212	299	246	263	274	413	338	96	76	60	Polysaccharide pyruvyl transferase
COG1596	18281	17349	11345	43047	52046	48256	44116	52026	46205	38589	41707	46070	48665	40946	53403	19195	19085	13384	polysaccharide transmembrane transporter activity
COG4881	123	73	20	5111	4486	4888	1841	4543	3356	2204	5445	1879	4696	4420	3921	93	77	37	polysulphide reductase NrfD
COG5557	5929	5130	1688	16280	20454	19860	14833	19557	16698	13469	15330	16095	18764	15485	21121	5269	5577	3781	polysulphide reductase NrfD
COG0389	19540	20802	16137	29472	34721	33322	28432	34718	30588	26861	30700	29846	32497	28223	35217	18614	18538	16970	Poorly processive error-prone DNA polymerase involved in untargeted mutagenesis. Copies undamaged DNA at stalled replication forks which arise in vivo from misma [illegible]
COG5518	482	812	2835	104	98	94	336	189	240	250	259	209	114	135	138	513	487	729	portal protein
COG2909	5478	5753	7175	8082	9156	9651	6982	6705	7291	6939	7276	8385	9263	8771	9215	7700	6915	6373	positive regulation of carbohydrate metabolic process
COG1933	123	73	20	5112	4486	4890	1841	4544	3356	2200	5446	1879	4697	4422	3922	93	77	37	Possesses two activities a DNA synthesis (polymerase) and an exonucleolytic activity that degrades single stranded DNA in the 3'- to 5'-direction. Has a template-prime [illegible]
COG3824	2640	3508	1805	1924	2222	2252	1547	1691	1788	1925	1687	2086	2244	2134	2267	2299	1854	2798	Possibl zinc metallo-peptidase
COG1753	123	73	20	5111	4486	4888	1841	4543	3356	2200	5445	1879	4696	4420	3921	93	77	37	Possibly the antitoxin component of a toxin-antitoxin (TA) module
COG2880	0	0	0	0	0	0	0	0	0	0	0	0	0	0	0	0	0	0	Possibly the antitoxin component of a toxin-antitoxin (TA) module
COG5302	50	172	177	12	8	5	69	31	25	65	26	49	8	6	5	67	57	30	Post-segregation antitoxin CcdA
COG0490	3707	4640	2266	194	301	276	251	192	307	482	211	271	169	200	213	2628	2429	3810	potassium ion transport
COG3263	2552	2061	3203	894	1067	1039	695	761	797	1698	899	1019	1173	1129	1040	2866	2800	2791	Potassium proton antiporter
COG0569	15671	16150	10575	16569	14568	14656	16029	14182	15648	14268	12950	16584	16096	16669	15276	14220	13261	14829	Potassium uptake protein
COG1226	1650	2745	2508	769	197	146	518	238	641	607	436	723	577	1075	225	2769	2360	2947	PotAssium voltage-gated channel
COG5651	140	83	43	52	175	273	128	121	80	84	108	134	82	110	141	100	87	142	ppe family
COG1606	3049	2513	773	8607	9958	9567	7556	9246	8417	6589	7229	8220	9721	8252	10273	2538	2687	1905	pp-loop domain protein
COG0248	9973	11710	11331	11144	12114	11770	12407	12450	12028	11460	11270	12760	11604	10994	12773	11196	10249	10674	ppx gppa phosphatase
COG3881	946	344	69	314	133	195	600	183	201	592	182	460	142	214	208	360	513	453	PRC-barrel domain protein
COG2243	1620	2434	3513	2148	1330	1403	2496	1592	2103	2342	2006	2500	1871	2227	1444	4437	3696	3582	Precorrin-2 c20-methyltransferase
COG1010	1109	1392	1853	1755	908	949	2076	1180	1690	1660	1420	1934	1305	1815	1029	2444	2046	1988	Precorrin-3B C17-methyltransferase
COG2875	1099	1489	1876	1909	1194	1228	2074	1254	1678	1798	1513	2025	1717	2036	1276	2536	2132	2095	precorrin-4 C(11)-methyltransferase
COG2099	867	1293	1776	1520	843	837	1647	965	1383	1314	1042	1660	1294	1625	879	2241	1912	1882	precorrin-6x reductase
COG2082	1139	1505	1888	2734	1173	1247	2858	1382	2144	2104	1722	2648	2147	2943	1300	2550	2190	2106	Precorrin-8x methylmutase
COG5572	975	1259	1861	524	346	298	1272	777	1241	970	910	1120	281	388	380	2175	1696	1630	Predicted integral membrane protein (DUF2282)
COG3356	150	92	29	5160	4573	4985	1868	4590	3393	2248	5475	1925	4768	4487	4003	120	100	71	Predicted membrane protein (DUF2070)
COG3815	769	243	47	135	143	171	73	89	88	395	106	115	147	134	175	232	387	350	Predicted membrane protein (DUF2085)
COG4025	0	0	0	0	0	0	0	0	0	0	0	0	0	0	0	0	0	0	Predicted membrane protein (DUF2101)
COG4035	0	0	0	0	0	0	0	0	0	0	0	0	0	0	0	0	0	0	Predicted membrane protein (DUF2104)
COG4036	0	0	0	0	0	0	0	0	0	0	0	0	0	0	0	0	0	0	Predicted membrane protein (DUF2105)
COG4039	0	0	0	0	0	0	0	0	0	0	0	0	0	0	0	0	0	0	Predicted membrane protein (DUF2108)
COG4040	0	0	0	0	0	0	0	0	0	0	0	0	0	0	0	0	0	0	Predicted membrane protein (DUF2109)
COG4331	20	36	18	8	5	6	24	15	11	35	11	24	5	8	5	25	24	15	Predicted membrane protein (DUF2127)
COG4854	0	0	0	0	0	0	0	0	0	0	0	0	0	0	0	0	0	0	Predicted membrane protein (DUF2178)
COG4906	6	2	0	8	12	11	6	12	18	5	8	11	6	16	8	4	4	1	Predicted membrane protein (DUF2206)
COG4329	2141	2466	1287	32	41	35	128	58	117	358	74	101	28	41	45	1332	1362	2058	Predicted membrane protein (DUF2243)
COG5395	295	383	248	234	174	190	425	287	298	451	333	380	154	206	247	260	293	235	Predicted membrane protein (DUF2306)
COG5346	1	0	0	1	0	0	1	0	1	2	2	3	0	2	0	0	1	2	Predicted membrane protein (DUF2335)
COG4031	0	0	0	0	0	0	0	1	0	3	0	0	0	0	1	0	0	0	Predicted metal-binding protein (DUF2103)
COG5423	98	137	34	255	327	322	162	243	211	200	168	266	453	250	263	108	103	115	Predicted metal-binding protein (DUF2284)
COG3900	360	431	256	845	899	1174	946	1046	984	938	953	1128	824	833	1250	375	391	309	Predicted periplasmic protein (DUF2092)
COG0701	5557	4340	3790	9897	10555	10270	8821	10072	9492	8388	8321	9411	10660	9795	10914	5139	5367	4457	Predicted permease
COG3368	0	0	0	1	0	0	2	0	0	0	0	0	1	2	0	0	0	0	Predicted permease (DUF2074)
COG5497	76	214	190	13	4	6	65	29	24	70	24	54	9	7	11	96	86	50	Predicted secreted protein (DUF2259)
COG5130	0	0	0	0	0	0	0	0	0	0	0	0	0	0	0	0	0	0	Prenylated RAB acceptor
COG1646	1169	768	127	5493	4924	5291	2170	4865	3734	3023	5844	2421	5073	4945	4375	688	865	671	Prenyltransferase that catalyzes the transfer of the geranylgeranyl moiety of geranylgeranyl diphosphate (GGPP) to the C3 hydroxyl of sn-glycerol-1-phosphate (G1P). [illegible]
COG0077	9635	10211	7035	16072	16656	16757	13705	16828	15560	12597	16212	14285	16469	15278	16648	8850	9080	8519	Prephenate dehydratase
COG0287	7813	7624	4701	16157	16741	16833	13460	16771	15093	12504	16143	14085	16554	15333	16766	6223	6126	5566	prephenate dehydrogenase
COG4937	0	0	0	0	0	0	0	0	0	0	0	0	0	0	0	0	0	0	prephenate dehydrogenase
COG0690	7112	6681	4128	9798	10920	10377	10442	10976	10523	8994	9432	10699	10521	9495	11177	5595	5277	5040	preprotein translocase subunit SecE
COG1374	0	0	0	0	0	0	0	0	0	0	0	0	0	0	0	0	0	0	pre-rRNA processing and 60S ribosome subunit assembly (By similarity)
COG2042	12	7	6	22	14	28	20	30	22	29	32	24	32	25	38	14	14	8	pre-rRNA processing protein involved in ribosome biogenesis (By similarity)
COG0260	8632	8922	7735	11256	12224	11732	12195	12715	12291	11106	11143	13037	11674	10727	12664	8259	7878	7610	Presumably involved in the processing and regular turnover of intracellular proteins. Catalyzes the removal of unsubstituted N-terminal amino acids from various pept [illegible]
COG2161	1972	2551	6040	2323	1284	1587	1965	1639	1756	1708	1582	2390	1770	2523	1467	2978	3463	2892	prevent-host-death family
COG4118	5591	4893	1902	14200	17175	16144	13231	16551	14580	11422	12802	13725	16364	13497	17512	4509	4667	3191	prevent-host-death family
COG0459	10917	11902	8012	28785	27725	28525	19387	27681	24032	19342	28802	20810	28633	27043	26573	10525	10346	9748	Prevents misfolding and promotes the refolding and proper assembly of unfolded polypeptides generated under stress conditions (By similarity)
COG0851	1429	1758	3245	1697	720	840	2661	1451	2129	1806	1749	2435	1144	1794	889	2703	2190	2124	Prevents the cell division inhibition by proteins MinC and MinD at internal division sites while permitting inhibition at polar sites. This ensures cell division at the prope [illegible]
COG1198	7990	8258	7308	15903	16152	16235	13745	16647	14945	12637	15978	14181	15974	14861	16172	7867	7505	7046	Primosomal protein n'
COG2965	803	705	1508	449	226	263	1465	822	1047	914	1224	1105	244	459	284	670	533	545	primosomal replication protein N
COG3923	203	370	1395	0	1	0	2	3	1	9	3	3	0	5	0	223	205	292	primosomal replication protein N
COG1971	3291	1781	3037	519	406	464	486	410	545	1378	547	604	344	441	446	1488	2046	2148	Probably functions as a manganese efflux pump (By similarity)
COG2917	1642	2105	3361	1259	1088	1045	2421	1774	1964	2210	2150	2319	956	1206	1222	3199	2465	2447	probably involved in intracellular septation (By similarity)
COG1780	2001	2928	2806	22	21	25	170	61	115	67	96	123	19	22	28	1543	1376	2196	Probably involved in ribonucleotide reductase function (By similarity)
COG0375	3182	2842	2125	13999	13825	13857	10195	13777	11893	9211	13078	10424	14013	13160	13492	2811	2767	2034	Probably plays a role in a hydrogenase nickel cofactor insertion step (By similarity)
COG0355	7938	8056	5965	10955	12135	11776	11715	12531	11930	10566	10801	12612	11878	10863	12668	7764	7469	6927	Produces ATP from ADP in the presence of a proton gradient across the membrane (By similarity)
COG1390	168	92	33	5181	4512	4936	1915	4594	3403	2265	5589	1939	4740	4489	3970	116	93	55	Produces ATP from ADP in the presence of a proton gradient across the membrane (By similarity)
COG1394	343	316	87	5721	5154	5609	2232	4981	3825	2691	5969	2445	5441	5048	4561	306	252	315	Produces ATP from ADP in the presence of a proton gradient across the membrane (By similarity)
COG0056	7944	8077	5968	10957	12132	11774	11720	12543	11933	10578	10802	12609	11879	10864	12669	7778	7485	6936	Produces ATP from ADP in the presence of a proton gradient across the membrane. The alpha chain is a regulatory subunit (By similarity)
COG0055	8768	8359	6010	10968	12144	11786	11738	12554	11948	10888	10820	12624	11886	10872	12679	8049	7941	7325	Produces ATP from ADP in the presence of a proton gradient across the membrane. The catalytic sites are hosted primarily by the beta subunits (By similarity)
COG0224	7944	8076	5968	10957	12132	11773	11721	12542	11932	10577	10802	12610	11878	10864	12669	7778	7485	6937	Produces ATP from ADP in the presence of a proton gradient across the membrane. The gamma chain is believed to be important in regulating ATPase activity and the [illegible]
COG1155	343	317	87	5727	5156	5615	2238	4991	3831	2698	5978	2451	5446	5056	4566	306	252	316	Produces ATP from ADP in the presence of a proton gradient across the membrane. The V-type alpha chain is a catalytic subunit (By similarity)
COG1156	343	319	87	5726	5159	5612	2235	4984	3828	2695	5970	2445	5444	5050	4565	306	253	317	Produces ATP from ADP in the presence of a proton gradient across the membrane. The V-type beta chain is a regulatory subunit (By similarity)
COG4043	7	2	1	16	20	21	18	26	31	13	21	25	25	24	32	4	4	2	ProFAR isomerase
COG0506	4635	4014	4027	7719	9671	9007	7883	9254	8076	7033	7453	8187	8750	7498	9723	3676	4006	3107	Proline dehydrogenase
COG3938	1830	2246	3858	1321	1380	1414	2747	1700	1779	2028	2053	2124	1298	1267	1601	3174	3474	2567	Proline racemase
COG1505	7769	7840	4597	14753	17803	16576	15018	17618	15793	12819	14054	15471	16544	13896	18269	6684	6686	5480	prolyl oligopeptidase
COG3760	410	470	311	378	365	334	670	489	448	747	638	629	293	344	412	477	373	317	prolyl-tRNA synthetase
COG1758	4824	5157	4921	7702	6200	6721	5327	7156	6761	5586	8461	5523	6788	7166	5939	4915	4371	5072	Promotes RNA polymerase assembly. Latches the N- and C- terminal regions of the beta' subunit thereby facilitating its interaction with the beta and alpha subunits (B [illegible]
COG5584	753	232	57	13	15	14	20	12	19	327	18	18	9	9	13	208	450	455	propeptide pepSY amd peptidase M4
COG4387	30	28	6	126	59	69	165	107	119	150	205	96	55	102	91	24	24	25	prophage protein
COG4933	263	446	1409	149	113	130	613	264	211	415	363	404	163	316	208	300	280	361	prophage protein
COG4935	381	929	1404	190	351	323	214	283	263	417	292	398	250	296	322	1854	1550	1359	proprotein convertase subtilisin kexin type
COG3052	214	377	1391	12	6	8	30	14	17	85	54	62	8	15	7	242	215	303	prosthetic group binding
COG3697	207	368	1390	2	2	2	3	3	3	25	5	13	1	2	3	228	204	293	prosthetic group biosynthetic process
COG0793	15046	15018	10079	33550	35111	35705	36532	35456	33614	31201	31650	37221	35002	34449	37364	16787	17319	12256	protease
COG4946	2309	2844	1317	2678	3063	3487	2041	2749	2515	1754	2134	2406	2927	2690	3298	1894	1780	2229	protease
COG5640	775	701	423	1607	1765	1882	942	1185	1267	1476	1255	1421	1878	1868	1927	699	486	798	protease
COG0501	17141	16651	12938	33076	34637	33871	30870	34929	32686	27692	32014	31816	34206	31787	35464	16388	15436	14471	Protease HtpX homolog
COG5405	5143	4475	3978	8125	9902	9327	8789	10311	9401	8341	8747	9414	9163	7605	10165	5420	5116	4388	Protease subunit of a proteasome-like degradation complex believed to be a general protein degrading machinery (By similarity)
COG0068	2977	2650	2077	12704	13335	13311	8698	13167	11014	8406	12451	9205	13116	11854	12963	2613	2608	1995	protein carbamoylation
COG2127	4435	5691	5336	5132	3442	3740	6449	4235	5251	5185	4764	6389	4222	5554	4007	5506	4798	4997	protein catabolic process
COG3196	184	170	91	256	305	323	228	215	242	176	177	280	279	146	378	201	298	318	protein conserved in bacteria
COG4908	2009	2628	1558	739	708	791	1318	716	783	1019	1036	1013	720	817	797	1757	1538	2131	protein containing a NRPS condensation (Elongation)
COG4310	182	229	66	240	358	294	180	237	226	236	198	336	452	259	291	254	157	161	Protein containing aminopeptidase domain
COG3870	1249	637	185	843	1028	1101	493	607	642	838	601	677	931	850	993	611	806	774	protein from nitrogen regulatory protein P-II
COG2961	1344	1743	3234	876	714	700	2132	1370	1776	1910	1809	1888	695	904	920	2698	2097	2113	protein involved in catabolism of external DNA
COG3914	3200	2922	1347	6714	5324	5758	7403	5121	6062	5758	5533	7286	5875	5846	6636	3625	3707	3163	protein N-acetylglucosaminyltransferase activity
COG1822	0	0	0	0	0	0	0	0	0	0	0	0	0	0	0	0	0	0	Protein of unknown function DUF70
COG3494	2923	2614	899	7385	9454	8946	6892	9133	7813	6514	7100	7664	8685	6975	9792	2650	2629	1859	Protein of unknown function (DUF1009)
COG3820	507	634	370	552	532	485	825	803	786	1008	786	1080	428	483	585	622	444	450	Protein of unknown function (DUF1013)
COG4382	19	20	2	4	2	2	10	7	6	23	5	13	3	5	9	20	15	11	Protein of unknown function (DUF1018)
COG5480	281	451	287	255	323	241	300	294	285	416	349	453	231	250	328	472	252	248	Protein of unknown function (DUF1036)
COG4737	702	1264	2989	289	177	189	758	456	583	500	493	536	166	201	198	2012	1697	1749	Protein of unknown function (DUF1044)
COG5321	399	569	320	409	431	396	499	479	445	695	500	675	337	368	460	544	374	393	Protein of unknown function (DUF1052)
COG4493	920	345	75	48	55	54	52	42	67	374	69	71	50	58	58	336	568	649	Protein of unknown function (DUF1054)
COG5466	142	252	204	215	222	260	102	106	111	118	116	109	293	255	242	135	106	107	Protein of unknown function (DUF1059)
COG4332	119	231	459	22	31	24	58	43	59	48	38	66	16	34	24	109	205	35	Protein of unknown function (DUF1062)
COG5294	806	246	66	16	22	18	26	14	24	317	27	25	10	14	18	234	476	489	Protein of unknown function (DUF1093)
COG4944	623	1034	1963	422	362	305	686	524	579	644	638	713	259	341	400	669	493	565	Protein of unknown function (DUF1109)
COG4905	32	31	11	48	52	58	33	41	53	75	68	76	57	63	55	62	23	109	Protein of unknown function (DUF1113)
COG4353	0	0	0	0	0	0	0	0	0	0	0	0	0	0	0	0	0	0	Protein of unknown function (DUF1122)
COG4835	1	0	1	1	1	0	1	0	2	0	0	2	1	0	1	1	1	1	Protein of unknown function (DUF1149)
COG5568	252	375	256	235	291	229	307	283	276	427	341	421	202	231	311	360	215	217	Protein of unknown function (DUF1150)
COG4034	58	25	12	57	68	78	31	55	66	41	50	60	51	59	48	24	27	32	Protein of unknown function (DUF1152)
COG3234	453	361	122	1892	2034	2454	1253	1942	1582	1084	1411	1406	1999	1861	2144	397	427	280	Protein of unknown function (DUF1175)
COG5319	784	819	389	680	595	586	1729	1116	1433	1408	1387	1516	544	614	722	817	531	543	Protein of unknown function (DUF1178)
COG4017	0	0	0	0	0	0	0	0	0	0	0	0	0	0	0	0	0	0	Protein of unknown function (DUF1188)
COG5509	244	381	256	190	196	158	302	216	229	352	218	311	149	192	228	354	222	217	Protein of unknown function (DUF1192)
COG5402	226	358	246	215	265	214	246	248	240	336	307	373	190	208	279	334	184	182	Protein of unknown function (DUF1214)
COG5361	1289	2094	2312	1175	1211	1302	2396	1710	1889	2080	1914	2033	1298	1272	1349	2525	2161	1944	Protein of unknown function (DUF1254)
COG3813	696	899	1735	282	244	195	760	386	356	763	653	531	190	258	263	873	665	646	Protein of unknown function (DUF1272)
COG3816	605	1139	1726	486	488	446	619	563	551	938	632	787	380	448	528	2141	1722	1697	Protein of unknown function (DUF1285)
COG3755	2297	3099	5988	2240	2390	2773	1923	2435	2092	2385	2167	2089	2254	2162	2519	4546	4231	4025	Protein of unknown function (DUF1311)
COG5588	2206	2934	1406	1542	1897	1936	1554	1453	1634	1150	1343	1576	1906	1473	1839	1876	1659	2243	Protein of unknown function (DUF1326)
COG5470	1580	1897	2358	1261	1352	1279	2616	1831	2211	2150	2157	2417	1224	1354	1439	1718	1281	1266	Protein of unknown function (DUF1330)
COG3506	578	580	623	391	450	456	298	285	344	348	299	412	346	413	431	362	438	273	Protein of unknown function (DUF1349)
COG3756	130	124	51	80	23	23	169	133	209	92	71	194	18	21	19	69	50	34	Protein of unknown function (DUF1376)
COG3310	493	342	148	482	382	418	1328	822	877	973	1181	1022	379	424	465	485	320	272	Protein of unknown function (DUF1415)
COG4807	1278	1489	2875	182	70	54	779	318	393	871	419	603	101	180	105	2305	2219	2068	Protein of unknown function (DUF1456)
COG2441	139	85	26	5142	4542	4952	1858	4576	3380	2232	5464	1906	4748	4466	3980	110	91	52	Protein of unknown function (DUF1464)
COG5317	424	590	326	468	464	431	587	525	516	765	569	720	357	408	514	561	387	406	Protein of unknown function (DUF1465)
COG5454	436	606	337	487	476	454	650	583	543	804	594	779	390	431	533	579	412	416	Protein of unknown function (DUF1467)
COG5516	3919	4156	2280	2415	2986	2955	2179	2195	3010	1715	1920	2743	2643	2637	2740	2535	2161	2888	Protein of unknown function (DUF1470)
COG5447	426	593	325	476	476	440	597	541	526	783	577	738	370	420	526	565	393	413	Protein of unknown function (DUF1491)
COG3822	652	858	1671	897	1102	1127	653	730	774	685	731	860	1065	1107	1059	577	567	556	Protein of unknown function (DUF1498)
COG4446	160	125	62	1061	252	271	1010	371	714	657	547	976	649	1129	322	146	151	133	Protein of unknown function (DUF1499)
COG4046	123	73	20	5111	4486	4888	1841	4543	3356	2200	5446	1879	4696	4420	3922	93	77	37	Protein of unknown function (DUF1512)
COG4371	2590	2082	626	7197	8053	7526	7581	8214	7381	5747	6298	7357	7752	6728	8310	2113	2278	1412	Protein of unknown function (DUF1517)
COG3367	503	647	1474	1034	403	424	657	482	846	583	454	834	959	1164	392	457	435	503	Protein of unknown function (DUF1611)
COG4089	91	90	39	307	117	139	632	219	211	294	167	489	119	195	151	153	80	86	Protein of unknown function (DUF1614)
COG4743	0	0	0	1	0	2	0	0	0	0	0	0	1	2	0	0	0	0	Protein of unknown function (DUF1616)
COG4828	1408	2164	1099	329	220	198	709	257	343	380	241	618	166	340	242	1182	974	1677	Protein of unknown function (DUF1622)
COG5469	259	305	138	1081	284	272	1059	418	753	728	560	1055	669	1152	349	355	266	210	Protein of unknown function (DUF1636)
COG2427	264	202	79	90	91	116	110	125	170	105	88	143	92	111	155	166	298	427	Protein of unknown function (DUF1641)
COG4333	45	44	39	85	63	74	121	101	90	119	195	123	77	113	87	35	52	27	Protein of unknown function (DUF1643)
COG4728	945	1046	1628	568	384	414	1206	677	1087	980	1125	993	334	456	416	2015	1623	1516	Protein of unknown function (DUF1653)
COG5508	314	448	303	376	401	344	473	412	425	634	494	626	300	360	459	447	269	283	Protein of unknown function (DUF1674)
COG3358	5498	6757	3348	5999	7868	7109	5805	7661	6634	5139	5829	6194	7093	5632	7837	4314	4043	4502	Protein of unknown function (DUF1684)
COG5506	737	208	33	13	14	13	18	12	18	311	16	16	7	8	11	199	377	324	Protein of unknown function (DUF1694)
COG3797	4570	5166	2621	6807	8741	8238	6696	8465	7328	5932	6826	6914	8000	6588	9042	3776	3793	3419	Protein of unknown function (DUF1697)
COG4453	530	448	220	1467	548	782	1653	924	1200	1104	953	1530	986	1567	850	303	331	282	Protein of unknown function (DUF1778)
COG4703	736	208	33	12	13	13	18	11	17	310	16	16	7	8	11	197	375	323	Protein of unknown function (DUF1797)
COG5267	4126	3643	1510	9087	11259	10982	9672	11039	9968	8579	9309	10154	10210	8511	11968	3940	3777	2717	Protein of unknown function (DUF1800)
COG5579	1740	3095	1455	379	431	417	528	480	558	591	438	720	385	510	487	1580	1555	2035	Protein of unknown function (DUF1810)
COG4954	612	852	1746	163	204	166	207	181	187	238	169	291	162	193	196	557	413	501	Protein of unknown function (DUF2000)
COG3877	470	396	155	973	1197	1245	627	741	805	611	721	862	1108	1040	1137	376	404	410	Protein of unknown function (DUF2089)
COG4859	9	2	5	4	3	3	8	6	4	12	17	20	7	10	8	10	8	14	Protein of unknown function (DUF2185)
COG5639	519	436	156	411	386	358	788	581	912	692	597	800	298	339	418	366	303	327	Protein of unknown function (DUF2274)
COG5559	78	122	27	192	278	252	121	179	163	159	127	218	400	207	219	93	84	98	Protein of unknown function (DUF2281)
COG5428	261	254	55	5521	4983	5381	2146	4928	3689	2524	5763	2307	5282	4825	4349	264	242	182	Protein of unknown function (DUF2283)
COG5626	435	700	1457	853	147	194	852	382	753	630	677	607	603	985	193	1786	1530	1406	Protein of unknown function (DUF2288)
COG5451	252	374	256	233	288	227	303	280	274	420	339	418	201	230	309	358	213	218	Protein of unknown function (DUF2385)
COG5589	301	774	1561	131	97	102	248	166	160	451	208	208	72	102	134	1708	1431	1404	Protein of unknown function (DUF2390)
COG3827	436	585	327	487	471	432	694	703	733	874	717	994	376	439	528	560	389	396	Protein of unknown function (DUF2497)
COG4019	0	0	0	0	0	0	0	0	0	0	0	0	0	0	0	0	0	0	Protein of unknown function (DUF3236)
COG1728	907	325	72	24	23	25	32	21	33	356	33	42	16	21	26	321	558	552	Protein of unknown function (DUF327)
COG3218	1293	1525	1866	1074	924	923	2117	1557	1779	1775	1627	1968	809	984	1037	2576	2072	2001	Protein of unknown function (DUF330)
COG1817	152	89	26	5178	4548	4962	1880	4600	3402	2244	5501	1933	4768	4475	3974	104	92	55	Protein of unknown function (DUF354)
COG1844	0	0	0	0	0	0	0	0	0	0	0	0	0	0	0	0	0	0	Protein of unknown function (DUF356)
COG5397	194	330	218	169	94	118	297	180	174	415	473	288	107	171	145	334	186	134	Protein of unknown function (DUF3620)
COG1906	81	92	20	265	265	321	217	236	214	219	233	261	238	263	254	105	107	79	Protein of unknown function (DUF401)
COG2306	1087	461	117	310	395	398	258	262	335	537	276	360	366	378	411	469	642	544	Protein of unknown function (DUF402)
COG2412	123	73	20	5112	4486	4890	1841	4544	3356	2200	5446	1879	4697	4422	3922	93	77	37	Protein of unknown function (DUF424)
COG2410	854	278	49	5164	4498	4914	1900	4574	3381	2541	5569	1906	4710	4461	3937	285	443	343	Protein of unknown function (DUF429)
COG4328	204	159	66	971	381	424	504	311	685	419	419	650	807	1192	425	146	195	115	Protein of unknown function (DUF429)
COG4923	125	162	70	112	150	118	116	115	108	177	96	172	118	128	136	221	124	125	Protein of unknown function (DUF429)
COG2430	0	0	0	0	0	0	0	0	0	0	1	0	0	0	1	0	0	0	Protein of unknown function (DUF432)
COG2442	2657	2984	1052	12363	7496	8400	9503	7349	9256	6875	6819	9956	11799	13706	7876	2336	2880	1918	Protein of unknown function (DUF433)
COG2830	0	1	1	0	0	1	0	0	1	0	0	0	0	0	0	0	0	1	Protein of unknown function (DUF452)
COG2833	798	664	347	627	401	467	1843	1072	1457	1370	1435	1433	444	513	559	719	448	402	Protein of unknown function (DUF455)
COG1978	1017	436	332	326	384	336	861	500	460	838	559	733	308	445	373	472	833	481	Protein of unknown function (DUF458)
COG2433	819	266	46	188	229	234	222	257	290	587	225	337	225	273	293	289	459	414	Protein of unknown function (DUF460)
COG2841	465	805	1437	497	477	490	556	559	627	685	501	751	609	432	482	1824	1472	1465	Protein of unknown function (DUF465)
COG5481	631	664	392	587	482	456	977	729	786	1040	881	1031	394	475	532	645	506	466	Protein of unknown function (DUF465)
COG5570	333	460	278	354	347	321	477	405	424	546	459	573	268	316	394	369	291	287	Protein of unknown function (DUF465)
COG1935	0	0	0	0	0	0	0	0	0	0	0	0	0	0	0	0	0	0	Protein of unknown function (DUF473)
COG1790	0	0	0	0	0	0	0	0	0	0	0	0	0	0	0	0	0	0	Protein of unknown function (DUF483)
COG2929	3191	2813	1076	8914	9469	8880	8040	9304	8605	6878	7417	8626	9879	8759	9574	2673	2861	1922	Protein of unknown function (DUF497)
COG1504	179	145	36	442	481	527	366	402	399	323	405	452	474	475	492	159	170	105	Protein of unknown function (DUF498/DUF598)
COG3737	1216	1007	507	1135	897	898	2332	1642	1945	1823	1974	2194	787	1009	1014	1106	764	714	Protein of unknown function (DUF498/DUF598)
COG1550	4834	4618	1820	7232	9488	8879	6224	8503	7392	5969	6458	6862	8749	6848	9391	3540	3618	3687	Protein of unknown function (DUF503)
COG1542	132	92	32	252	306	318	196	210	247	172	218	268	308	304	319	91	102	68	Protein of unknown function (DUF505)
COG2952	2214	1781	492	5984	7913	7260	5514	7605	6381	4826	5644	5846	7106	5500	7924	1784	1910	1121	Protein of unknown function (DUF507)
COG1627	3	2	1	0	0	0	0	0	0	0	0	1	0	0	0	5	4	0	Protein of unknown function (DUF515)
COG5418	1	0	0	1	1	0	0	0	1	3	0	4	1	1	1	1	1	0	Protein of unknown function (DUF523)
COG1700	148	101	36	273	338	353	207	226	263	195	230	296	331	328	347	105	116	96	Protein of unknown function (DUF524)
COG1771	0	0	0	0	0	0	0	0	0	0	0	0	0	0	0	0	0	0	Protein of unknown function (DUF530)
COG1772	0	0	0	0	0	0	0	0	0	0	0	0	0	0	0	0	0	0	Protein of unknown function (DUF531)
COG2450	0	0	0	1	0	2	0	0	0	0	2	0	1	2	1	0	0	0	Protein of unknown function (DUF552)
COG4701	326	642	1429	166	107	84	230	197	279	260	161	269	82	98	118	1648	1360	1313	Protein of unknown function (DUF721)
COG5389	505	634	370	552	530	486	824	803	784	1002	777	1075	426	479	583	622	444	448	Protein of unknown function (DUF721)
COG5489	1102	1251	533	926	843	815	1436	1197	1479	1634	1227	1611	689	757	910	1135	873	890	Protein of unknown function (DUF736)
COG1415	403	507	538	5555	4965	5353	2785	5099	3878	2859	6055	2710	5119	4987	4415	549	601	290	Protein of unknown function (DUF763)
COG3564	2950	3377	1899	423	364	374	902	558	637	1304	801	836	343	493	468	1896	2123	2538	Protein of unknown function (DUF779)
COG3372	218	149	63	1185	420	491	1066	439	818	640	528	1030	891	1364	530	151	200	130	Protein of unknown function (DUF790)
COG5505	236	416	77	259	218	204	374	327	405	494	333	529	222	293	259	315	227	205	Protein of unknown function (DUF819)
COG3502	559	666	373	554	707	619	639	646	683	890	653	955	537	660	729	666	517	514	Protein of unknown function (DUF952)
COG4550	911	324	72	27	25	29	36	22	41	333	31	29	19	23	23	312	559	553	Protein of unknown function (DUF964)
COG3536	3636	3420	2409	8176	10103	9690	8328	10030	8761	7559	8548	8754	9204	7797	10514	4602	4232	3376	Protein of unknown function (DUF971)
COG3374	6	2	2	14	14	11	8	7	9	10	5	9	9	12	14	5	5	5	Protein of unknown function (DUF981)
COG2245	158	112	36	5188	4579	5002	1896	4590	3420	2271	5502	1956	4768	4513	4005	122	117	80	Protein of unknown function (DUF996)
COG4530	264	400	263	243	301	237	318	298	288	450	352	440	213	243	321	380	237	236	Protein of unknown function (FYDLN_acid)
COG1852	305	327	100	689	799	836	547	622	589	522	529	733	892	566	846	366	386	331	Protein of unknown function DUF116
COG2034	0	0	0	1	0	2	0	0	0	0	0	0	1	2	0	0	0	0	Protein of unknown function DUF131
COG1568	161	119	40	321	417	412	228	266	304	208	261	321	359	364	380	120	124	89	Protein of unknown function DUF43
COG2361	3644	3197	1160	11777	10104	9726	10176	10002	10332	8300	8627	10576	11850	12189	10478	3097	3364	2223	Protein of unknown function DUF86
COG2445	1388	831	173	6362	5761	6353	2839	5691	4368	3497	6502	3093	6027	5632	5145	850	1097	949	Protein of unknown function DUF86
COG1578	139	76	26	200	179	227	130	246	161	127	164	318	134	284	151	88	126	92	Protein of unknown function DUF89
COG1076	3824	4110	5112	6915	8618	7852	8354	9256	8471	7117	7922	8213	7731	6372	8784	6081	5457	4603	protein oligomerization
COG5023	0	0	0	0	0	0	0	0	0	0	1	0	0	0	1	0	0	0	protein polymerization
COG0185	7878	7965	5939	15936	16438	16502	13388	16732	15068	12607	16096	14096	16242	15040	16434	7713	7372	6799	Protein S19 forms a complex with S13 that binds strongly to the 16S ribosomal RNA (By similarity)
COG4794	284	144	86	100	91	73	586	301	671	163	305	388	90	83	89	105	67	73	protein secretion
COG3031	419	802	1470	454	419	505	446	500	500	743	485	653	482	478	536	1854	1534	1615	protein secretion by the type II secretion system
COG2208	24610	18684	8513	56084	67270	63925	49854	63273	57259	44872	48734	54157	63265	53309	68427	19657	20857	14953	protein serine threonine phosphatase
COG2900	838	1397	3105	328	216	175	720	423	455	734	669	661	190	286	230	2309	1851	1853	Protein slyX homolog
COG2922	822	743	1533	610	409	464	1094	812	1068	956	1147	1147	411	595	501	741	582	599	Protein smg homolog
COG2443	0	0	0	1	0	2	0	0	0	0	2	0	1	2	1	0	0	0	protein transport protein SEC61 gamma subunit homolog
COG3017	1182	1380	2975	732	454	500	1815	1185	1496	1306	1515	1554	455	658	552	2371	1946	1906	protein transporter activity
COG5599	0	1	1	16	15	24	21	27	22	17	28	24	39	18	47	0	0	0	protein tyrosine phosphatase
COG5198	0	0	0	0	0	0	0	0	0	0	0	0	0	0	0	0	0	0	Protein tyrosine phosphatase-like
COG2365	3807	4871	3827	3086	3544	3763	3320	3032	3058	3498	3285	3671	3319	3258	3705	4860	4236	4695	protein tyrosine serine phosphatase
COG3862	49	47	21	99	115	135	66	61	76	114	74	114	89	114	105	59	50	52	protein with conserved CXXC pairs
COG4372	1361	1504	2825	1147	401	495	1233	569	956	1281	713	1196	802	1275	511	3510	3199	3018	protein with myosin-like
COG4248	477	354	120	1169	1105	1245	1291	951	1051	987	853	1381	980	994	1301	401	399	371	protein with protein kinase and helix-hairpin-helix DNA-binding
COG3171	384	919	2740	0	1	0	5	3	5	152	40	36	0	2	0	1789	1505	1552	Protein with unknown function (DUF469)
COG3531	914	1583	3134	322	303	252	629	586	545	827	707	951	229	467	286	3467	3663	2809	Protein-disulfide isomerase
COG2201	8001	8267	7978	13661	13976	13839	15743	15052	14816	14660	14285	16200	14217	13853	15087	11493	10454	9793	protein-glutamate methylesterase activity
COG1440	882	1340	4390	33	38	35	63	28	52	116	40	83	25	27	28	777	785	1135	protein-N(PI)-phosphohistidine-sugar phosphotransferase activity
COG3859	794	277	75	96	113	136	77	64	86	397	81	107	86	109	100	251	495	500	Proton-coupled thiamine transporter YuaJ
COG1232	12320	10659	6667	21579	26765	24969	20223	25453	22415	18521	19907	21384	24834	20261	27090	9317	9756	8200	protoporphyrinogen oxidase
COG4635	4223	6699	4546	283	334	348	394	280	421	272	321	370	328	342	356	3419	2800	5101	protoporphyrinogen oxidase
COG0796	7069	7470	5737	9610	10636	10149	10083	10665	9823	9168	9347	10368	10318	9376	11072	7247	7028	6525	Provides the (R)-glutamate required for cell wall biosynthesis (By similarity)
COG0208	6269	7068	7003	7327	8382	7401	8595	8760	8655	7465	7878	8863	7950	7095	8536	6595	6183	5874	Provides the precursors necessary for DNA synthesis. Catalyzes the biosynthesis of deoxyribonucleotides from the corresponding ribonucleotides (By similarity)
COG0209	9660	9702	8159	22522	23041	23649	17971	23516	20483	17137	24085	18601	22525	20700	22777	9564	9015	8361	Provides the precursors necessary for DNA synthesis. Catalyzes the biosynthesis of deoxyribonucleotides from the corresponding ribonucleotides (By similarity)
COG1372	60	56	20	701	55	80	310	91	441	254	223	389	508	900	113	53	83	120	Provides the precursors necessary for DNA synthesis. Catalyzes the biosynthesis of deoxyribonucleotides from the corresponding ribonucleotides (By similarity)
COG0207	4767	5432	5062	3064	2239	2419	4475	3156	3855	4307	3797	4536	2522	3428	2782	5258	4865	5125	Provides the sole de novo source of dTMP for DNA biosynthesis (By similarity)
COG5104	0	0	0	0	0	0	0	0	0	0	0	0	0	0	0	0	0	0	PRP40 pre-mRNA processing factor 40 homolog
COG5178	5947	6013	3730	6244	8251	7303	7371	8010	7542	7395	7253	7843	7335	6250	8629	6063	5369	5907	PRP8 pre-mRNA processing factor 8 homolog (S. cerevisiae)
COG4539	654	1149	1889	435	282	223	1546	701	783	1184	1171	1095	316	438	359	2141	2022	1536	PRS2 protein
COG3980	461	884	1654	177	175	138	417	222	218	543	366	314	115	158	175	1854	1678	1710	pseudaminic acid biosynthesis-associated protein PseG
COG0564	19021	19332	18153	24880	26987	26703	27947	28387	27357	26632	26120	29786	26243	24909	29012	20087	19720	18363	pseudouridine synthase activity
COG1187	14323	13950	14494	17517	17308	17688	20570	19071	19629	18358	18543	20683	17196	17222	18635	15532	15477	13496	pseudouridine synthase activity
COG1774	1508	1167	509	1318	1527	1531	1486	1304	1210	1721	1291	1629	1548	1467	1455	1119	1425	1001	PSP1 C-terminal conserved region
COG1263	8952	8256	15491	668	724	730	1084	733	997	3014	899	1100	584	521	843	8062	8341	9308	PTS System
COG1264	1870	2946	2549	495	559	569	556	459	579	683	484	646	448	363	651	3023	2593	3210	pts system
COG1299	3884	5206	9784	386	406	407	1004	503	578	1039	609	819	380	291	494	4381	4081	5059	pts system
COG1445	806	1268	4253	271	319	335	247	227	264	244	203	317	287	157	390	877	862	1091	PTS System
COG1455	798	1230	4228	32	39	36	38	23	44	105	48	62	29	28	33	814	832	1160	pts system
COG1762	7524	9499	11865	4258	4451	5005	4935	4680	4842	5425	4973	5562	4374	4656	5269	7542	6621	8424	PTS System
COG2190	4310	4910	8199	498	563	564	831	566	767	1403	620	758	442	362	625	4377	4222	5074	pts system
COG2893	1256	1070	512	1318	1019	1092	2500	1835	2132	2127	2189	2425	954	1193	1205	1199	834	870	pts system
COG3414	2892	3411	4175	26	27	24	130	46	93	387	74	93	16	29	22	1924	1900	2759	PTS System
COG3444	258	410	1410	104	104	115	87	89	94	148	117	143	97	115	133	302	250	423	pts system
COG3715	256	409	1409	102	100	109	81	87	93	142	115	140	94	111	131	297	247	422	PTS System
COG4668	1008	1267	3131	278	328	338	284	245	293	227	208	349	288	175	397	848	853	976	pts system
COG3037	2630	3135	3933	21	18	18	109	44	98	368	72	78	15	21	22	1802	1805	2665	PTS system ascorbate-specific transporter subunit IIC
COG3925	0	0	0	0	0	0	0	0	0	0	0	0	0	0	0	0	0	0	Pts system fructose-specific
COG3731	23	40	20	8	3	5	14	8	9	29	10	24	5	3	5	27	30	21	PTS system glucitol sorbitol-specific IIA component
COG2213	1957	2848	2737	28	46	36	118	54	119	74	66	106	24	40	53	1515	1300	2072	PTS system mannitol-specific
COG3716	261	411	1412	109	113	116	87	87	99	146	117	148	102	119	143	303	251	426	PTS system mannose fructose sorbose family iid component
COG0474	13792	15931	12722	19873	18361	18490	17479	18637	17762	15220	18585	17790	19458	19458	18255	12126	12622	13109	P-type atpase
COG2217	24360	25175	20572	33383	33744	33900	34460	35056	33929	32122	34209	35266	34044	33401	35302	25592	26463	23433	p-type ATPase
COG1549	29	16	6	47	50	67	19	39	35	28	35	30	44	37	38	12	15	18	PUA domain containing protein
COG4945	53	63	20	104	127	143	76	72	97	112	96	127	105	135	120	56	54	153	pullulanase
COG5099	209	152	48	537	621	675	339	392	452	366	401	480	563	595	644	158	144	116	pumilio homolog
COG3808	4253	3918	1595	14370	15772	15741	11068	15461	12999	11006	14816	12092	15214	13484	15663	4079	3965	2764	pump that utilizes the energy of pyrophosphate hydrolysis as the driving force for
COG0513	22870	24198	23623	28479	27385	28072	28388	29820	28455	28092	32559	28231	27869	27679	27821	24979	24480	22676	purine NTP-dependent helicase activity
COG1201	8215	8715	6049	18868	22206	21223	15992	21864	18413	14983	19314	16642	20876	17462	21900	8588	8142	7063	purine NTP-dependent helicase activity
COG5042	2764	2458	1930	5946	7808	7128	6163	7879	6768	5149	5875	6165	6982	5377	7822	3401	3981	2512	Purine nucleoside permease
COG0813	1337	826	1784	119	76	74	229	175	296	460	140	288	60	83	90	624	965	902	purine nucleoside phosphorylase DeoD-type
COG4196	830	1313	1760	955	962	1039	1391	1144	1228	1612	1366	1453	930	867	1281	2396	1929	1899	Putative amidoligase enzyme (DUF2126)
COG2738	1549	1183	543	1494	1690	1852	1703	1481	1461	1997	1562	1905	1548	1715	1941	1156	1484	1048	Putative neutral zinc metallopeptidase
COG4318	153	173	75	142	154	129	358	235	262	245	284	252	162	244	201	238	116	115	Putative ParB-like nuclease
COG4267	120	51	15	132	88	105	199	234	114	144	189	299	66	198	76	74	94	59	Putative trans-membrane protein
COG3466	0	0	0	0	0	0	0	0	0	0	0	0	0	0	0	0	0	0	Putative transposon-encoded protein (DUF2080)
COG0856	2	5	2	4	4	12	1	3	3	6	3	6	6	6	9	4	1	4	pyrE-like protein
COG0446	11831	16367	12242	10751	10260	11274	8987	10879	9974	8988	11969	9103	10418	10310	10226	15129	12857	16021	pyridine nucleotide-disulfide oxidoreductase
COG2210	1363	809	211	5953	5442	5846	2503	5323	4112	3295	6205	2810	5910	5300	4821	770	998	1014	pyridine nucleotide-disulfide oxidoreductase
COG2240	1222	1648	3217	582	610	590	1238	831	1200	904	880	1106	484	551	650	2258	1922	2038	Pyridoxal kinase
COG5015	62	46	11	251	249	288	126	143	169	179	167	191	204	236	255	61	33	35	pyridoxamine 5'-phosphate
COG5135	60	34	20	859	12	59	813	155	506	356	238	666	472	938	75	27	74	22	pyridoxamine 5'-phosphate
COG0748	757	1181	1719	1744	833	996	2015	1150	1508	1405	1273	1713	1364	1839	1060	2179	1771	1811	pyridoxamine 5-phosphate
COG0221	6782	7670	7449	9792	10856	10248	10668	11167	10804	9259	9712	11173	10317	9624	11161	7199	6766	6413	Pyrophosphate phospho-hydrolase
COG4284	790	1128	1539	1509	1804	1935	1253	1435	1400	1622	1414	1716	1861	1853	2045	2181	1920	1802	pyrophosphorylase
COG0345	10443	9321	7717	10899	12089	11754	11660	12231	11793	11416	10714	12289	11706	10941	12753	8677	8870	8196	pyrroline-5-carboxylate reductase activity
COG3961	895	935	1666	688	835	877	644	666	759	593	582	867	802	682	1014	765	724	763	Pyruvate decarboxylase

COG4012	162	127	48	342	411	456	223	264	298	226	284	325	410	390	461	122	114	93	pyruvate format-lyase activating enzyme
COG0469	9230	9676	9148	18048	16865	17003	15813	17515	16360	14076	17282	16098	17561	17288	17030	10442	9822	9060	Pyruvate kinase
COG3848	2	5	2	2	4	11	1	2	4	5	4	6	5	4	10	4	4	4	Pyruvate kinase
COG1945	2213	1804	510	6024	8027	7311	5420	7566	6301	4792	5548	5781	7347	5486	7846	1790	1904	1132	Pyruvoyl-dependent arginine decarboxylase
COG1365	0	0	0	0	0	0	0	0	0	0	0	0	0	0	0	0	0	0	Queuosine synthesis-like protein
COG3916	1519	2763	6170	634	556	523	1043	971	764	1190	1011	1209	435	735	513	4388	3665	3609	quorum sensing
COG3457	72	317	63	92	111	131	76	68	106	118	89	144	89	126	125	79	196	76	Racemase
COG5432	0	0	0	0	0	0	0	0	0	0	0	0	0	0	0	0	0	0	RAD18 homolog (S. cerevisiae)
COG0535	19372	17737	10640	48488	56946	55592	43765	53901	47985	40411	43044	48334	55416	46641	58528	19798	19543	15357	radical SAM domain protein
COG0641	2098	2837	4852	4900	4217	4869	4688	4111	4173	4007	3657	4904	4445	4918	4680	4802	3614	3968	radical SAM domain protein
COG0731	509	399	114	6746	5402	6037	2977	5623	4632	3354	6610	3438	6151	6363	5032	422	462	339	radical SAM domain protein
COG1032	23352	20149	7542	77427	85623	84217	61562	82434	71495	56578	70747	66185	84968	72876	85448	21295	22240	15500	radical SAM domain protein
COG1244	200	164	73	338	386	431	272	293	310	327	285	379	394	364	500	210	246	210	radical SAM domain protein
COG1313	248	195	53	5530	4872	5354	2169	4907	3679	2553	5784	2260	5076	4808	4300	234	229	156	radical SAM domain protein
COG1533	9666	9722	5521	9789	12046	11533	10521	11671	11011	10109	10181	11207	11337	9766	12546	8581	8220	8500	radical SAM domain protein
COG1964	6208	6015	2472	17281	20576	19715	13227	19992	16437	12159	17024	13958	19095	15526	20194	5143	5315	4129	radical SAM domain protein
COG2108	3	1	0	2	2	3	0	2	1	9	2	7	2	3	1	6	1	1	radical SAM domain protein
COG2516	310	606	1608	159	157	190	222	157	163	309	244	250	153	208	205	389	344	409	radical SAM domain protein
COG4001	0	0	0	0	0	0	0	0	0	0	0	0	0	0	0	0	0	0	radical SAM domain protein
COG4277	704	1002	704	929	1000	980	1602	1082	1074	1360	1243	1483	830	1101	1064	1006	991	516	radical SAM domain protein
COG5011	422	392	132	1623	934	1010	1351	814	1168	1006	843	1468	1433	1776	963	381	402	410	radical SAM domain protein
COG5014	0	1	0	4	0	0	88	14	14	17	74	21	4	7	4	4	1	1	radical SAM domain protein
COG1242	1143	600	126	796	822	881	1060	784	710	1117	794	965	909	768	783	642	889	758	radical SAM protein
COG5238	31	27	13	46	45	59	134	70	48	116	113	91	56	45	73	36	30	24	RAN GTPase activating protein 1
COG2962	10620	12674	15411	9033	10744	10011	11578	11640	11169	11600	11233	11674	9843	8782	11009	16948	16397	14389	rard protein
COG0797	7174	7531	6458	15942	18200	16850	15652	18282	16898	14170	15069	16718	17287	15438	18465	9158	8708	6620	rare lipoprotein A
COG4305	39	38	9	70	95	86	63	70	103	112	92	155	87	121	117	59	27	132	rare lipoprotein a
COG2980	1439	1799	4372	781	512	579	1896	1288	1627	1372	1581	1664	519	732	669	2612	2179	2232	Rare lipoprotein B
COG4154	745	933	695	475	486	480	978	597	634	765	873	775	472	369	572	875	736	548	RbsD or FucU transport
COG1714	8822	11038	7722	5582	4910	5412	6645	5341	6097	6752	5625	7488	5195	6109	5926	9584	8835	10565	rdd domain containing protein
COG4819	31	37	32	5	4	4	6	4	14	4	3	11	2	3	6	20	82	134	Reactivating factor for ethanolamine ammonia lyase
COG5471	66	73	29	100	98	120	84	99	69	162	86	96	78	77	107	78	56	94	reca rada recombinase
COG3598	941	1030	1690	1317	1162	1243	1854	1183	1303	1462	1186	1928	1075	1222	1468	1043	807	914	RecA-family
COG2251	1924	2493	1331	997	207	200	1312	443	1202	590	480	1157	613	1165	262	1292	1165	1797	RecB family nuclease
COG4080	0	0	0	0	0	0	0	0	0	0	0	0	0	0	0	0	0	0	RecB-family nuclease-like protein
COG1629	59134	65404	48351	108356	125635	115982	159351	143148	129257	132381	128998	150303	114656	110106	131162	75714	78554	51637	receptor
COG4771	24372	25919	24828	47071	57821	53266	63137	62133	56037	51510	54128	60468	52493	46968	60200	27375	28698	18730	receptor
COG4772	1081	1785	3044	976	792	816	2007	1263	1147	2060	1872	1971	790	1205	961	2851	2453	2244	receptor
COG4773	7869	11243	23707	6347	4969	5925	13697	9228	9741	11304	10926	13002	5235	8758	6640	17097	14930	13899	receptor
COG4774	7067	7244	8962	9605	10978	10503	12379	12946	12302	11218	11955	12952	10000	9533	11635	11811	11040	8929	Receptor
COG5052	0	0	0	0	0	0	0	0	0	0	0	0	0	0	0	0	0	0	receptor accessory protein
COG2256	7995	8107	5974	15554	16767	16844	13292	16748	14755	12542	16065	13835	16096	14385	16811	7922	7534	7013	Recombination factor protein RarA
COG3723	384	209	315	34	41	21	323	165	505	49	114	248	22	30	22	145	250	102	recT protein
COG1281	3546	2822	3519	3021	1928	2170	3991	2564	3358	4064	3164	3875	2309	2974	2413	3818	3665	3615	Redox regulated molecular chaperone. Protects both thermally unfolding and oxidatively damaged proteins from irr [illegible]
COG0656	18273	22266	19285	17215	18963	19212	15043	19044	17873	14177	17935	15602	18149	16210	18868	17089	15282	18779	reductase
COG3007	761	1220	1522	1033	1026	1180	1189	1111	1260	1147	1097	1341	944	1059	1311	2126	1970	1650	reductase (By
COG0175	13882	14914	11361	25901	26638	26287	24447	27655	25923	22269	26261	25296	26480	25246	27234	14871	13799	12723	Reduction of activated sulfate into sulfite (By similarity)
COG1438	5018	4832	3193	6431	8434	7774	5841	7851	6812	5481	5933	6248	7586	6004	8457	3644	3781	3889	Regulates arginine biosynthesis genes (By similarity)
COG1551	2901	3068	4742	3089	3644	3898	2169	2762	2731	3020	2469	3067	4059	3396	3692	4974	4571	4479	regulation of carbohydrate metabolic process
COG1792	5895	5487	4761	10653	11686	11398	11314	12017	11463	10153	10469	11909	11354	10412	12258	6160	6185	4905	regulation of cell shape
COG0745	139064	138473	116117	215877	229414	223642	228866	231068	227558	199127	205209	234046	226747	212546	240391	151168	147604	131022	regulatoR
COG3279	19489	20044	11072	33203	41432	38435	42022	42720	37808	37659	37752	41531	37498	34378	42621	18474	20817	13015	Regulator
COG3835	4791	4640	4470	2358	2890	3019	1416	1697	2052	2313	1796	2007	2674	2565	2716	4465	4344	5072	regulatoR
COG5566	107	59	12	3	3	4	7	4	5	11	11	10	3	6	7	80	73	55	regulatoR
COG5184	2510	2543	1111	5078	5491	6681	4742	5708	5493	6048	5518	6513	5923	6200	7217	2052	2039	2309	regulator of chromosome condensation
COG3107	878	1151	2931	423	258	271	700	488	551	694	679	640	261	299	312	2084	2502	1810	Regulator of peptidoglycan synthesis that is essential for the function of penicillin-binding protein 1A (PBP1a) (By sim [illegible]
COG3417	499	640	1480	626	660	653	995	671	718	718	686	937	760	755	657	506	477	582	Regulator of peptidoglycan synthesis that is essential for the function of penicillin-binding protein 1B (PBP1b) (By sim [illegible]
COG5037	0	0	0	0	0	0	0	0	0	0	0	0	0	0	0	0	0	0	Regulatory protein
COG3725	582	1039	2824	273	220	232	346	354	443	389	291	446	206	187	264	1914	1593	1649	regulatory protein AmpE
COG3901	260	143	68	127	62	42	369	205	270	236	260	287	46	65	47	185	156	101	Regulatory protein NosR
COG2357	4478	4252	1771	4412	6252	5142	4432	5775	5011	4305	4400	4714	5524	3918	6029	3063	3279	3396	relA SpoT domain protein
COG3843	592	545	184	504	538	444	907	707	1073	900	716	1050	421	546	587	546	413	434	relaxase mobilization nuclease domain protein
COG2039	1632	1127	1722	1392	600	657	1430	669	1036	1355	973	1381	1022	1496	675	944	1058	1122	Removes 5-oxoproline from various penultimate amino acid residues except L-proline (By similarity)
COG1859	724	1057	1933	1041	506	523	769	463	844	729	645	915	891	1194	637	2023	1944	1647	Removes the 2'-phosphate from RNA via an intermediate in which the phosphate is ADP-ribosylated by NAD followe [illegible]
COG0242	14872	15611	10991	17079	18947	17604	19313	19662	19155	16886	17185	19695	18192	16543	19533	14139	13409	13534	Removes the formyl group from the N-terminal Met of newly synthesized proteins. Requires at least a dipeptide for a [illegible]
COG0024	14158	15381	12229	19749	19692	20183	17452	20055	18621	16128	19417	17915	19843	19140	19842	13763	13012	13444	Removes the N-terminal methionine from nascent proteins (By similarity)
COG4705	1232	1440	2039	1384	1443	1547	1889	1406	1575	1304	1523	1607	1479	1398	1637	1118	1298	1084	Repeat of Unknown Function (DUF347)
COG0457	5009	4354	1462	14561	14557	14866	14202	14444	14098	11336	11713	14702	15308	14625	15652	4446	4521	3417	repeat-containing protein
COG5534	705	568	192	631	580	543	1145	973	1232	869	876	1204	458	626	585	585	443	435	replication
COG5275	0	0	0	0	0	0	0	0	0	0	0	0	0	0	0	0	0	0	replication factor c
COG3611	895	321	72	20	20	17	40	21	40	348	36	58	13	20	22	308	557	556	replication initiation and membrane attachment
COG2946	50	55	35	192	177	213	167	190	157	150	178	187	170	174	176	73	92	39	Replication initiation factor
COG4195	23	49	27	23	20	39	30	29	24	63	40	47	33	34	52	35	31	29	Replication Protein
COG5235	0	0	0	0	0	0	0	0	0	0	0	0	0	0	0	0	0	0	Replication Protein
COG5527	630	658	1645	245	255	168	831	433	445	725	676	515	221	365	226	632	658	542	replication protein
COG5655	64	88	48	42	63	36	45	46	46	69	42	82	45	47	61	132	119	186	Replication Protein
COG0305	9246	9310	7804	13213	13961	13761	13145	13627	13640	12000	12277	14260	13931	13257	14697	8919	8498	7912	Replicative dna helicase
COG2932	2360	2954	4883	2103	1924	1867	3791	2585	2768	3078	2737	3619	1818	1905	1981	3875	3159	3114	Repressor
COG1094	123	73	20	5112	4486	4890	1841	4544	3356	2200	5446	1879	4697	4422	3922	93	77	37	Required for 40S ribosome biogenesis. Involved in nucleolar processing of pre-18S ribosomal RNA and ribosome asse [illegible]
COG1161	2082	832	241	1172	199	277	1368	437	855	1613	958	1150	671	1216	318	951	1400	1294	Required for a late step of 50S ribosomal subunit assembly. Has GTPase activity (By similarity)
COG1196	24295	24380	14922	67310	63119	65943	45930	63670	55768	45886	67387	48155	65605	63547	61675	24606	24016	23495	Required for chromosome condensation and partitioning (By similarity)
COG1495	3021	3054	4863	1368	1056	1050	3044	2197	2837	2921	2570	2996	946	1210	1230	4976	4429	4467	Required for disulfide bond formation in some periplasmic proteins. Acts by oxidizing the DsbA protein (By similarity)
COG5623	0	0	0	0	0	0	0	0	0	0	0	0	0	0	0	0	0	0	Required for endonucleolytic cleavage during polyadenylation-dependent pre-mRNA 3'-end formation (By similarity)
COG0779	6786	7000	5450	9339	10133	9653	10355	10787	10352	9145	9369	10645	9689	9019	10538	6884	6603	6143	Required for maturation of 30S ribosomal subunits (By similarity)
COG0829	2779	3815	3276	2098	1331	1305	3353	1725	2473	2560	2388	2957	1638	2245	1568	3991	3327	3858	Required for maturation of urease via the functional incorporation of the urease nickel metallocenter (By similarity)
COG0830	2633	3800	3276	2079	1314	1303	3067	1588	2004	2545	2298	2749	1628	2244	1562	4046	3375	3900	Required for maturation of urease via the functional incorporation of the urease nickel metallocenter (By similarity)
COG1723	168	164	60	189	220	157	272	351	398	401	301	553	192	285	256	258	174	200	Required for meiotic nuclear division 1 homolog (S. cerevisiae)
COG5109	0	0	0	0	0	0	0	0	0	0	0	0	0	0	0	0	0	0	Required for meiotic nuclear division 5 homolog
COG2176	3553	3811	3625	2213	2257	2503	2242	2071	2527	3342	2538	2731	2133	2360	2523	5306	5154	4352	Required for replicative DNA synthesis. This DNA polymerase also exhibits 3' to 5' exonuclease activity (By similarity)
COG2386	3837	3845	3822	7409	9284	8586	7907	9396	8161	7277	7797	8465	8369	7102	9462	4853	4466	3572	Required for the export of heme to the periplasm for the biogenesis of c-type cytochromes (By similarity)
COG3705	2252	1910	1961	2061	1000	1122	3096	1824	2399	2847	2365	2927	1422	2154	1294	3042	2748	2687	Required for the first step of histidine biosynthesis. May allow the feedback regulation of ATP phosphoribosyltransfe [illegible]
COG0533	7924	7982	5949	15950	16450	16521	13406	16745	15097	12624	16111	14122	16257	15055	16446	7737	7382	6808	Required for the formation of a threonylcarbamoyl group on adenosine at position 37 (t(6)A37) in tRNAs that read co [illegible]
COG3642	594	289	93	10448	9127	9925	4273	9448	7497	4644	11181	4307	9526	8898	8004	275	208	128	Required for the formation of a threonylcarbamoyl group on adenosine at position 37 (t(6)A37) in tRNAs that read co [illegible]
COG3117	1185	1382	2975	732	455	500	1816	1186	1496	1311	1516	1557	457	659	553	2377	1952	1906	Required for the translocation of lipopolysaccharide (LPS) from the inner membrane to the outer membrane (By simi [illegible]
COG4232	8250	8459	8212	16445	20423	19282	18927	20963	18876	16118	17567	19342	18797	15712	21054	10461	10058	7523	Required to facilitate the formation of correct disulfide bonds in some periplasmic proteins and for the assembly of t [illegible]
COG5249	0	0	0	0	0	0	0	0	0	0	0	0	0	0	0	0	0	0	RER1 retention in endoplasmic reticulum 1 homolog (S. cerevisiae)
COG5654	3272	3524	3813	6333	8184	7480	7073	8493	7636	6009	6571	7186	7408	6102	8289	4103	3893	2971	RES domain-containing protein
COG3036	386	920	2765	0	1	0	6	3	5	143	43	34	0	2	0	1791	1513	1572	rescue of stalled ribosome
COG1961	25338	27484	16598	30539	35172	34006	32550	35205	31996	28963	30147	34241	33346	30206	36254	23005	21452	22485	Resolvase
COG2452	7047	5351	1747	22711	23906	24013	15398	19994	19552	13931	19102	18144	24358	23219	23449	5356	5659	3730	resolvase
COG0784	8396	9865	5070	21263	20846	21516	22161	21859	21552	21422	20128	24578	20960	22758	23577	10387	9859	7708	response regulator
COG3437	12132	11154	6055	29545	32470	31439	28345	31487	28639	25811	27735	30077	32020	28565	33438	13697	13610	10119	Response Regulator
COG3706	29713	27438	24700	63386	62778	60462	65606	65492	63729	56959	59918	66512	64753	62272	65682	35949	33792	28342	response regulator
COG3707	17763	27760	16447	2474	2568	2714	3422	2364	2939	3280	3291	3128	2437	2634	2742	16340	13006	22041	response regulator
COG4567	1392	1632	1905	1244	996	980	2479	1750	2007	2233	2042	2388	896	1188	1190	2762	2131	2156	response regulator
COG3947	656	630	378	579	632	661	499	627	580	539	502	806	532	567	718	643	780	878	response regulator receiver and SARP domain protein
COG3558	3607	3675	4040	7279	8994	8486	8322	9140	8003	7078	7717	8181	8250	6884	9388	4615	4369	3338	Response regulator receiver domain protein
COG3685	5057	5014	2548	11021	13525	13293	12567	13512	12274	9976	11094	12558	12453	10875	14172	4812	5234	3122	response to DNA damage stimulus
COG0071	17242	18645	13292	39900	39748	41317	28917	39313	33556	29657	41614	30763	40453	37937	38445	17439	16584	16100	response to heat
COG3247	7455	8397	5254	10207	12423	12245	9705	11878	10583	8292	9316	10397	11556	9803	12483	5912	5461	6062	response to pH
COG0585	699	1161	2851	670	625	738	653	753	786	830	668	882	648	657	716	2080	1724	1960	Responsible for synthesis of pseudouridine from uracil- 13 in transfer RNAs (By similarity)
COG1258	123	73	20	5112	4486	4890	1841	4544	3356	2200	5446	1879	4697	4422	3922	93	77	37	Responsible for synthesis of pseudouridine from uracil- 54 and uracil-55 in the psi GC loop of transfer RNAs (By simila [illegible]
COG0130	7043	7628	5864	15732	16129	16234	13221	16546	14883	12133	15948	13858	15834	14833	16201	7419	6918	6395	Responsible for synthesis of pseudouridine from uracil- 55 in the psi GC loop of transfer RNAs (By similarity)
COG0189	12942	10871	10546	26773	26165	26988	21017	26688	23983	20884	26728	22448	26545	25646	25954	13634	14160	11547	Responsible for the addition of glutamate residues to the C-terminus of ribosomal protein S6 (By similarity)
COG0233	7755	7892	5919	10824	11951	11612	11548	12190	11712	10408	10650	12219	11545	10618	12515	7620	7295	6762	Responsible for the release of ribosomes from messenger RNA at the termination of protein biosynthesis. May increa [illegible]
COG1715	2443	1748	638	3295	3067	3206	2862	2684	3086	2941	2695	3519	3702	3387	3207	1673	1869	1660	restriction
COG1401	2515	2670	1253	1476	1043	1025	1119	847	1291	1515	1080	1472	1361	1943	1129	1612	1699	2292	restriction endodeoxyribonuclease activity
COG1787	876	323	76	76	87	87	79	82	89	376	66	96	72	92	84	336	467	426	restriction endonuclease
COG2810	132	86	29	5112	4487	4889	1855	4548	3362	2210	5462	1894	4697	4422	3923	107	86	40	restriction endonuclease
COG4127	1055	840	1424	134	94	96	335	224	290	643	289	331	106	194	149	1820	1774	1738	restriction endonuclease
COG0732	5687	5542	3736	12679	12294	12128	12488	12445	12140	11209	11298	13245	12834	12828	12832	6093	6431	4325	Restriction modification system DNA (Specificity
COG5222	3	1	0	2	2	2	2	2	2	4	3	9	0	6	0	3	6	10	Retinoblastoma binding protein 6
COG2801	82635	79820	43206	32089	33510	33357	84058	56328	100185	34824	47774	69058	31128	31608	33495	48893	42139	55961	Retrotransposon protein
COG3330	425	400	141	1652	975	1063	1349	779	1163	966	790	1452	1478	1648	1072	429	477	498	Rho termination
COG0607	31099	32548	24105	36369	40044	39594	40180	40981	39024	36329	37116	41087	38764	36082	42499	29521	28336	28184	Rhodanese domain protein
COG5524	78	288	52	280	88	136	635	210	190	278	149	494	109	193	135	64	193	68	rhodopsin
COG0705	16512	16494	9473	33623	37310	36292	29989	36087	32699	27714	31673	32287	36347	32909	37972	15044	15914	12699	rhomboid family
COG3501	7156	8242	12226	9941	10863	10354	12795	11890	11464	11763	12240	11903	10738	9989	11695	13556	13683	10489	Rhs element vgr protein
COG4253	479	275	197	157	83	41	1070	420	369	545	698	388	89	119	49	458	399	261	Rhs element vgr protein
COG0196	7940	8042	5985	11050	12265	11929	11673	12228	11758	10444	10708	12258	11821	10760	12883	7812	7529	6959	riboflavin biosynthesis protein ribF
COG1731	0	0	0	1	0	2	0	0	0	0	0	0	1	2	0	0	0	0	riboflavin synthase (EC 2.5.1.9)
COG1530	9492	9367	8870	11060	11702	11416	13252	13124	12924	11967	11932	13550	11369	10753	12453	10097	9483	8844	ribonuclease
COG3341	263	209	28	274	328	333	189	213	227	229	235	281	233	281	304	338	166	127	ribonuclease
COG3719	766	1235	1783	501	514	495	783	658	642	1023	770	892	426	493	590	2277	1842	1776	ribonuclease
COG4290	1691	2344	1186	337	165	195	1098	575	731	745	941	828	192	284	252	1398	1100	1786	ribonuclease
COG1295	16693	16250	10645	25731	30183	28532	27262	30169	28215	24147	25660	28132	28571	25273	31115	14876	14598	12764	ribonuclease BN
COG0328	8857	8515	6244	16462	16159	16250	13865	16777	15424	13215	16643	14390	16514	15550	16177	8138	8111	7566	ribonuclease H activity
COG1658	1978	772	165	5352	4708	5154	2080	4778	3594	3045	5697	2156	4902	4639	4146	798	1255	1168	ribonuclease M5 activity
COG1841	7319	7463	5757	13843	15073	14881	11402	15188	13227	10935	14579	11797	14313	12770	14714	7170	6767	6315	ribosomal large subunit biogenesis
COG1383	123	73	20	5112	4486	4890	1841	4544	3356	2200	5446	1879	4697	4422	3922	93	77	37	Ribosomal protein
COG1552	123	73	20	5112	4486	4890	1841	4544	3356	2200	5446	1879	4697	4422	3921	93	77	37	Ribosomal protein
COG1631	123	73	20	5112	4486	4890	1841	4544	3356	2200	5446	1879	4697	4422	3922	93	77	37	Ribosomal protein
COG1727	123	73	20	5112	4486	4890	1841	4544	3356	2200	5446	1879	4697	4422	3922	93	77	37	Ribosomal protein
COG1890	123	73	20	5112	4486	4890	1841	4544	3356	2200	5446	1879	4697	4422	3922	93	77	37	Ribosomal protein
COG1997	123	73	20	5113	4487	4892	1841	4544	3356	2201	5447	1879	4698	4424	3922	93	77	37	Ribosomal protein
COG2053	123	73	20	5112	4486	4890	1841	4544	3356	2200	5446	1879	4697	4422	3922	93	77	37	Ribosomal protein
COG2058	123	73	20	5112	4486	4890	1841	4544	3356	2200	5446	1879	4697	4422	3922	93	77	37	Ribosomal protein
COG2075	123	73	20	5112	4486	4890	1841	4544	3356	2200	5446	1879	4697	4422	3922	93	77	37	Ribosomal protein
COG2157	0	0	0	1	0	2	0	0	0	0	0	0	1	2	0	0	0	0	ribosomal protein
COG2167	0	0	0	1	0	2	0	0	0	0	0	0	1	2	0	0	0	0	Ribosomal protein
COG2238	123	73	20	5112	4486	4890	1841	4544	3356	2200	5446	1879	4697	4422	3922	93	77	37	Ribosomal protein
COG2451	0	0	0	0	0	0	0	0	0	0	0	0	0	0	0	0	0	0	Ribosomal protein
COG4830	123	73	20	5111	4486	4888	1841	4543	3356	2200	5446	1879	4696	4420	3922	93	77	37	Ribosomal protein
COG5045	0	0	0	0	0	0	0	0	0	0	0	0	0	0	0	0	0	0	Ribosomal protein
COG4076	248	164	67	156	213	124	859	358	332	507	521	586	154	295	213	238	242	158	Ribosomal protein L11 methyltransferase (PrmA)
COG1632	123	73	20	5112	4486	4890	1841	4544	3356	2200	5446	1879	4697	4422	3922	93	77	37	Ribosomal protein L15
COG2147	123	73	20	5112	4486	4890	1841	4544	3356	2200	5446	1879	4697	4422	3922	93	77	37	ribosomal protein L19
COG4901	0	0	0	0	0	0	0	0	0	1	0	0	0	0	1	0	0	0	Ribosomal protein S25
COG4919	123	73	20	5111	4486	4888	1841	4543	3356	2200	5445	1879	4696	4420	3921	93	77	37	Ribosomal protein S30
COG4067	967	2007	4228	1182	496	498	757	668	966	1128	705	1168	1062	1436	483	5147	4258	4059	ribosomal protein S6 modification protein
COG5192	9	3	2	1	2	1	2	2	5	2	3	5	1	1	1	2	2	1	Ribosome biogenesis protein bms1
COG5154	0	0	0	0	0	0	0	0	0	0	0	0	0	0	0	0	0	0	ribosome biogenesis protein brx1
COG1500	123	73	20	5112	4486	4890	1841	4544	3356	2200	5446	1879	4697	4422	3922	93	77	37	Ribosome maturation protein
COG1069	4586	5215	3779	7010	9224	8547	7057	8802	7612	6205	6856	7449	8239	6762	9361	3995	3948	3559	ribulokinase
COG0036	8226	8632	7526	11421	12612	12427	12135	12735	12230	11064	11344	12864	12212	11432	13393	8135	7736	7207	ribulose-phosphate 3-epimerase activity
COG4638	20155	26482	22701	18269	14834	15480	24750	19321	21921	20449	19237	22571	16209	17397	16343	24499	21352	24709	rieske 2fe-2S domain-containing protein
COG5152	0	0	0	0	0	0	0	0	0	0	0	0	0	0	0	0	0	0	Ring finger protein
COG5540	123	73	20	5111	4486	4888	1841	4543	3356	2200	5445	1879	4696	4420	3921	93	77	37	RING finger ubiquitin ligase
COG1098	2397	769	162	65	63	78	84	63	88	1026	100	99	60	69	85	734	1382	1322	RNA binding s1 domain protein
COG1041	1197	598	125	5886	5166	5598	2848	5214	4030	3229	6050	2936	5503	5188	4647	640	764	757	RNA methylase
COG1460	123	73	20	5112	4486	4890	1841	4544	3356	2200	5446	1879	4697	4422	3922	93	77	37	rna polymerase
COG1595	142137	130103	74506	248749	305444	299037	262766	294435	265415	235961	247963	275449	284782	250417	317898	138712	141625	113764	RNA Polymerase
COG4941	5338	5086	3640	10243	12759	12266	9442	11364	10649	9334	9802	10811	11620	10057	13261	6134	5812	4810	rna polymerase
COG5507	1132	1614	3143	637	531	489	1291	875	1275	1341	1295	1325	496	666	662	2535	1957	2066	RNA signal recognition particle 4.5S RNA
COG0724	2626	2517	1043	10526	6867	7954	10819	7351	8612	7859	7495	10660	9090	11284	8138	2673	3173	1974	Rna-binding protein
COG1491	123	73	20	5112	4486	4890	1841	4544	3356	2200	5447	1884	4697	4422	3922	93	77	37	Rna-binding protein
COG1532	47	53	3	189	176	210	164	190	153	142	176	182	168	172	175	69	68	36	Rna-binding protein
COG1534	2313	1905	3118	1355	1131	1359	2444	1882	2198	2419	2235	2426	1168	1431	1536	2957	2784	2797	Rna-binding protein
COG2016	123	73	20	5112	4486	4890	1841	4544	3356	2200	5446	1879	4697	4422	3922	93	77	37	Rna-binding protein
COG3344	12647	9596	4834	23062	24446	24179	22514	21415	22480	18261	18641	23838	25631	22970	24520	9667	10467	8120	RNA-directed DNA polymerase
COG0594	6091	5531	4761	10013	11150	10730	10706	11358	10779	9478	9584	11099	10795	9807	11658	6172	6146	4984	RNaseP catalyzes the removal of the 5'-leader sequence from pre-tRNA to produce the mature 5'-terminus. It can als [illegible]
COG1033	2994	3431	3321	4315	4626	4995	4473	4436	4378	5546	4816	5638	4753	4711	5132	5682	4710	4771	RND superfamily
COG2018	1600	1179	500	4720	3133	3306	3079	2265	3715	2737	2632	3851	3990	5349	3180	1251	1098	1099	Roadblock lc7 family protein
COG2891	2185	1767	3074	1703	573	703	2756	1477	2166	2069	1885	2368	1039	1717	830	2731	2596	2504	Rod shape-determining protein MreD
COG1940	45560	52663	39031	54621	66219	62842	50741	62163	55951	45407	52288	53797	61494	52798	66372	36347	34666	37464	ROK family
COG1684	5107	4426	3977	7971	9663	9155	8531	9960	8997	8048	8464	8894	9045	7565	9942	5440	5117	4415	Role in flagellar biosynthesis (By similarity)
COG0820	7076	6306	4945	11890	13076	12960	12595	13136	12582	11608	11875	13381	12696	11824	14096	7301	7250	6033	rRNA (adenine-C2-)-methyltransferase activity
COG1576	5279	4867	4308	8362	10179	9607	9351	10464	9597	8531	8903	9779	9306	7832	10453	5812	5701	4588	rRNA processing
COG2260	0	0	0	1	0	2	0	0	0	0	2	0	1	2	1	0	0	0	rRNA processing
COG1773	2010	2013	1934	3947	2347	2511	5461	3217	4151	4160	3989	4942	3458	4315	2584	3255	2753	2453	rubredoxin
COG1592	2761	2305	652	7998	9135	8718	7015	8793	8007	6461	7264	7627	8901	7580	9323	2423	2341	1714	Rubrerythrin
COG1633	2160	1625	591	3376	3730	4247	2686	3410	3320	2749	2688	3204	3872	3227	3849	1549	1662	1551	Rubrerythrin
COG1224	0	0	0	1	0	2	0	0	0	0	0	0	1	2	0	0	0	0	ruvB-like
COG5061	0	0	0	0	0	0	0	0	0	0	0	0	0	0	0	0	0	0	S. cerevisiae
COG5081	0	0	0	0	0	0	0	0	0	0	0	0	0	0	1	0	0	0	S. cerevisiae
COG2996	1280	1325	1771	647	671	788	599	655	691	1297	744	901	612	826	849	2251	2267	1957	S1 RNA binding domain protein
COG2302	994	397	110	958	127	197	894	222	599	779	333	786	562	1050	179	381	674	612	s4 domain protein
COG2501	3365	3465	2911	1686	653	762	2884	1160	1638	1989	1667	2070	1163	1765	804	2309	2345	2967	s4 domain protein
COG1748	2176	2946	3812	7800	6222	6738	5548	6596	6587	4853	7888	5378	6763	7099	5971	3250	3228	2845	saccharopine dehydrogenase
COG3268	969	1241	1790	1207	1180	1190	1594	1095	1286	1342	1205	1620	1132	1236	1251	2238	1813	1923	saccharopine dehydrogenase
COG5379	556	623	347	687	799	824	812	678	711	690	667	874	694	466	964	601	649	506	S-adenosylmethionine diacylglycerol 3-amino-3-carboxypropyl
COG4091	345	530	301	522	326	319	927	449	404	742	484	827	287	399	385	523	309	297	saf domain-containing protein
COG0152	7967	8245	6182	10973	12139	11769	11732	12353	11866	10679	10804	12459	11678	10789	12703	7952	7544	7068	SAICAR synthetase
COG3137	1187	1628	4361	831	766	846	1016	964	950	1003	926	1156	909	719	764	1227	1104	1339	Salt-induced outer membrane protein
COG2384	369	678	1422	28	23	25	38	25	47	203	74	104	23	29	26	1720	1510	1538	sam-dependent methyltransferase
COG2949	1247	1256	2265	1426	1626	1774	1377	1409	1427	1274	1331	1599	1453	1254	1877	1347	1415	1080	SanA protein
COG4311	2505	3632	3087	515	464	367	1567	869	1183	1224	1147	1126	378	425	441	3562	2865	3364	sarcosine oxidase delta subunit
COG2340	9465	8072	4270	11433	12246	11756	10908	11361	11050	11143	9889	11845	12319	11647	12700	8105	8363	8068	Scp-like extracellular
COG3204	475	1180	2769	139	106	78	235	150	149	580	303	305	121	159	108	3195	2673	2580	sdia-regulated domain-containing protein
COG5226	0	0	0	0	0	0	0	0	0	0	0	0	0	0	0	0	0	0	Second step of mRNA capping. Transfer of the GMP moiety of GTP to the 5'-end of RNA via an enzyme-GMP covalen [illegible]
COG0432	5825	5190	4160	16999	17225	17405	12860	16632	14980	12403	15829	13887	17604	16375	17340	6333	6067	5040	Secondary thiamine-phosphate synthase enzyme
COG5621	1092	1410	1815	1179	1307	1311	1171	1143	1087	1323	1364	1512	1187	1163	1292	2621	2178	2061	secreted hydrolase-like protein
COG2847	2186	2567	3431	6761	5957	6398	4707	6572	5866	4924	8222	4698	6069	5948	5594	4747	3745	3624	Secreted protein
COG3533	7498	6724	3657	16434	20826	19805	17423	20236	17701	14438	16301	17692	18907	16331	21267	6381	6890	4243	Secreted protein
COG3544	7195	8462	4377	9204	10483	10068	9437	10172	9638	7802	8481	9518	9889	9186	10793	5727	5597	5740	Secreted protein
COG4086	759	235	57	15	18	18	22	13	20	342	20	31	12	11	14	217	451	458	Secreted protein
COG4805	10659	9860	3879	20644	25660	24172	21769	26139	23098	18707	20413	22460	23460	19150	26758	8660	8739	6829	secreted protein
COG5341	51	50	22	171	133	168	145	113	121	181	222	187	131	179	144	64	55	55	Secreted protein
COG5429	2544	2277	1072	6218	8146	7384	6376	7988	6742	5614	6227	6571	7295	5859	8256	2278	2304	1402	secreted protein
COG5513	218	576	1390	9	11	27	19	15	23	148	51	56	12	12	18	1591	1329	1294	Secreted protein-like protein
COG3926	613	794	1584	339	305	274	757	505	439	651	601	642	259	285	310	679	575	613	secretion activator protein
COG1566	43294	46131	51522	92214	100845	96158	97192	103768	97260	82701	87243	99269	98279	89097	104775	58676	57086	44384	secretion protein HlyD family
COG3029	10	13	3	14	6	8	33	19	17	60	67	64	9	14	13	11	14	12	Seems to be involved in the anchoring of the catalytic components of the fumarate reductase complex to the cytopla [illegible]
COG3080	10	13	3	14	6	8	33	19	17	60	67	64	9	14	13	11	14	12	Seems to be involved in the anchoring of the catalytic components of the fumarate reductase complex to the cytopla [illegible]
COG0222	7830	7921	5930	10837	11961	11627	11655	12216	11745	10447	10752	12277	11559	10644	12533	7654	7320	6792	Seems to be the binding site for several of the factors involved in protein synthesis and appears to be essential for ac [illegible]
COG0790	8929	10384	11599	15380	17152	17340	17369	18481	16367	17025	17220	18374	15610	14662	18635	17380	15580	13218	Sel1 domain protein repeat-containing protein
COG1515	1158	1124	1922	7215	6060	6534	3962	5838	5028	3610	6780	4023	6527	6604	5560	1008	1112	974	Selectively cleaves double-stranded DNA at the second phosphodiester bond 3' to a deoxyinosine leaving behind the [illegible]
COG3276	1046	1036	1610	2587	2846	3324	2026	2671	2381	1737	2169	2176	2786	2622	3005	942	945	931	Selenocysteine-specific translation elongation factor
COG3526	1795	1709	3082	5647	5051	5395	3194	5272	4462	3420	6303	3055	5253	5124	4558	2504	2363	2147	selenoprotein
COG3447	439	644	1606	5790	4501	4923	2280	4645	3786	2516	5716	2295	5158	5267	3974	394	406	377	sensor protein
COG1507	1908	2571	1428	323	382	402	256	208	328	196	207	267	381	383	373	1360	1157	1876	Septum formation initiator family protein
COG1395	250	153	85	123	150	130	257	145	155	198	227	172	105	120	97	201	175	121	sequence-specific DNA binding
COG3066	204	370	1395	0	2	1	2	3	2	13	4	6	0	5	1	224	205	292	Sequence-specific endonuclease that cleaves unmethylated GATC sequences. It is involved in DNA mismatch repair (I [illegible]
COG4221	30608	35455	27070	46036	54096	51728	50918	54192	49959	42770	46144	51162	50985	45489	55617	30978	29981	26731	serine 3-dehydrogenase activity
COG1045	9311	10312	9514	13811	13184	13019	15864	14290	14623	13838	13751	15681	13678	13977	14093	10844	10232	9416	serine acetyltransferase
COG0265	33395	33313	19873	64914	68127	68483	60553	66768	61895	54544	61340	64159	68721	62815	70612	31723	30557	28815	Serine protease
[illegible]	[illegible]	[illegible]	[illegible]	[illegible]	[illegible]	[illegible]	[illegible]	[illegible]	[illegible]	[illegible]	[illegible]	[illegible]	[illegible]	[illegible]	[illegible]	[illegible]	[illegible]	[illegible]	[illegible]

COG5641	0	0	0	0	0	0	0	0	0	0	0	0	0	0	0	0	0	0	Transcription factor
COG1734	10981	11645	13469	13241	15271	15322	14149	15699	14577	13602	13931	15169	14268	12786	15960	13174	12307	12019	Transcription factor that acts by binding directly to the RNA polymerase (RNAP). Required for negative regulation of rRNA expression and positive regulation of several amino acid biosynthesis promoters
COG5095	0	0	0	0	0	0	0	0	0	0	0	0	0	0	0	0	0	0	Transcription initiation factor TFIID
COG5179	0	0	0	0	0	0	0	0	0	0	0	0	0	0	0	0	0	0	transcription initiation factor tfiid subunit
COG5625	123	73	20	5111	4486	4888	1841	4543	3356	2200	5445	1879	4696	4420	3921	93	77	37	Transcription regulator
COG1221	718	1397	4175	1113	261	343	956	303	679	689	442	894	675	1173	329	2099	1832	1908	transcriptional
COG1316	11058	11623	5862	4362	3516	3684	3083	2258	3631	3300	2430	3478	4078	4932	3350	6986	7049	9772	transcriptional
COG3933	12	6	6	5	10	9	8	4	8	37	9	23	6	5	7	23	11	14	transcriptional
COG5114	0	0	0	0	0	0	0	0	0	0	0	0	0	0	0	0	0	0	transcriptional
COG5453	93	210	179	9	3	4	54	24	23	48	22	43	6	6	8	107	82	40	transcriptional activator HlyU
COG0583	171638	224549	369682	117226	125212	120336	215369	166795	192269	166940	176488	183029	117383	110519	133647	297712	264051	256702	Transcriptional regulator
COG0789	47875	48314	36899	49054	57767	55265	52860	56859	55797	48051	49861	54757	53484	48075	58970	46446	44126	44143	Transcriptional regulator
COG0864	1477	909	264	6237	5682	6261	2986	5792	4560	4013	6728	3330	5961	5674	5307	1024	1025	1038	Transcriptional regulator
COG1309	144693	160747	141883	150524	178972	172565	168435	172555	170339	148708	153642	171640	165688	149252	182961	153604	142366	144838	Transcriptional regulator
COG1356	0	0	0	0	0	0	0	0	0	0	0	0	0	0	0	0	0	0	Transcriptional regulator
COG1378	949	539	188	15727	13974	15150	6116	14096	10957	6911	16759	6325	14524	13711	12248	547	534	435	Transcriptional regulator
COG1396	28961	33369	31622	27730	33260	31561	32824	33733	33126	28403	29314	32209	30566	27102	34340	29277	27125	28748	Transcriptional regulator
COG1476	15256	13276	11566	20391	21515	20771	20000	21147	20869	19004	18544	20888	21589	20576	22485	13755	14544	13185	Transcriptional regulator
COG1497	0	0	0	1	1	2	0	1	0	4	0	0	1	2	0	0	0	0	Transcriptional regulator
COG1510	1597	1877	924	1987	2240	2361	2790	2243	2482	2473	2569	2843	2055	2163	2508	1670	1724	1305	Transcriptional regulator
COG1609	104660	128033	103240	89209	109793	108316	95477	106415	98697	89597	93104	100334	102452	98507	116949	89541	83324	95772	Transcriptional regulator
COG1695	53703	46253	21453	120640	149287	142937	104166	142087	120976	92258	111404	109751	136566	111609	148482	42261	44362	33336	Transcriptional regulator
COG1737	11721	12096	23455	7640	9407	8667	10620	10313	10075	9981	9469	9571	8377	6918	9311	13932	13793	12736	Transcriptional regulator
COG1846	78516	87506	68649	64966	76771	74232	77941	75879	77466	68099	70120	78132	70780	64288	78466	75845	70103	74630	Transcriptional regulator
COG2169	6768	7652	6690	10349	12667	12521	10409	12298	10931	9361	10281	10914	11638	9947	13119	8634	7948	7594	Transcriptional regulator
COG2345	4273	3946	2163	12908	11199	12075	6202	10932	9330	6498	12640	6466	11823	11703	10077	2938	3110	3294	Transcriptional regulator
COG2378	13416	12529	8248	16317	18722	18469	16183	16795	17004	15328	15584	18225	17733	17109	19129	11753	11416	10677	Transcriptional regulator
COG2390	7061	8686	9488	1576	1813	1842	2514	1921	2060	2599	2041	2258	1579	1613	1824	6348	5882	7221	Transcriptional regulator
COG2508	11553	13494	7073	3168	3875	4060	1867	2081	2762	2456	2034	2343	3604	3494	3419	7647	7206	11054	Transcriptional regulator
COG2512	598	409	193	15382	13528	14738	5596	13681	10120	6690	16396	5731	14145	13320	11830	359	306	209	Transcriptional regulator
COG2522	271	155	46	10225	8974	9779	3690	9090	6722	4402	10894	3770	9396	8843	7846	189	157	76	Transcriptional regulator
COG2808	6262	6447	5233	6985	8954	8253	7965	9150	8775	6938	7399	8091	8008	6356	9099	6182	5887	5645	Transcriptional regulator
COG2865	4547	4022	2651	9751	11141	10847	9352	10642	9653	8586	8988	9936	11005	9826	11575	3694	3998	2709	Transcriptional regulator
COG2944	5310	5554	8190	8526	9499	8895	9796	10287	10140	7705	8423	9542	9148	8003	9714	6933	7046	5739	Transcriptional regulator
COG3054	203	370	1395	1	1	0	2	3	1	9	4	3	0	5	0	223	205	292	Transcriptional regulator
COG3283	604	1601	4296	15	16	11	49	24	24	297	93	87	10	16	16	3402	2857	2868	Transcriptional regulator
COG3284	6840	10223	10864	1768	1606	1669	3785	2358	2958	3868	3582	3536	1527	1808	1921	9014	7733	9631	Transcriptional regulator
COG3355	6	3	4	15	15	13	8	8	8	12	5	9	11	14	15	6	9	8	Transcriptional regulator
COG3398	288	170	78	5199	4560	4965	2142	4660	3452	2379	5721	2004	4767	4496	3972	252	217	131	Transcriptional regulator
COG3423	224	391	1399	50	5	16	59	29	21	63	120	31	12	44	16	245	223	303	Transcriptional regulator
COG3449	1216	2017	4737	403	356	338	1344	767	1081	1002	869	1008	395	498	518	2445	2041	2158	Transcriptional regulator
COG3604	8399	9415	8577	15997	20026	19102	15294	19394	16751	14718	15537	16857	18666	15300	20612	10133	10219	8941	Transcriptional regulator
COG3629	4339	5678	3120	2587	3262	3197	1652	1915	2622	1873	1861	2552	2695	3037	3048	3500	2755	4320	Transcriptional regulator
COG3655	9924	14393	8357	1740	1718	1786	3012	1914	2149	2817	2211	2631	1530	1893	1879	9105	7925	12188	Transcriptional regulator
COG3708	4589	2951	3534	321	351	288	904	447	472	2178	665	816	294	382	336	5105	6027	4847	Transcriptional regulator
COG3710	8932	7157	2089	22158	29724	26759	21223	28775	24533	18572	21416	22398	26576	20289	30111	7084	7541	4577	Transcriptional regulator
COG3800	2700	3866	2513	878	936	900	1165	1024	1052	1366	1039	1472	757	854	996	2303	2111	2579	Transcriptional regulator
COG3829	10217	7728	7054	11346	12111	12166	12134	12767	12732	14000	11988	12989	12238	11285	13288	10793	10710	10711	Transcriptional regulator
COG3860	2704	2781	1340	320	385	384	367	297	408	624	361	458	360	389	388	1639	1718	2350	Transcriptional regulator
COG3888	44	32	8	49	52	80	68	86	110	50	60	82	61	70	113	22	24	33	Transcriptional regulator
COG3899	8704	8294	5078	17306	17162	17255	14613	14795	15799	13642	13399	16293	18470	18617	18041	7222	6553	7465	Transcriptional regulator
COG3903	2627	3344	4175	3508	4510	4436	2356	2711	3077	2585	2586	3331	3979	3767	4147	4972	4179	4083	transcriptional regulator
COG4189	1820	1627	763	7637	7308	8103	3654	7041	5604	3900	7420	4016	7491	7085	6866	1424	1326	1065	Transcriptional regulator
COG4190	298	146	75	156	68	49	809	413	479	298	455	420	73	84	57	179	147	97	Transcriptional regulator
COG4470	892	320	71	15	17	16	25	13	23	329	28	22	10	13	16	305	555	545	Transcriptional regulator
COG4650	367	714	1452	301	350	375	276	262	288	399	286	392	343	227	462	1790	1557	1502	Transcriptional regulator
COG4738	0	0	0	1	0	2	0	0	0	0	0	0	1	2	0	0	0	0	Transcriptional regulator
COG4742	291	160	42	10229	8983	9787	3692	9096	6734	4415	10910	3782	9400	8855	7875	206	172	93	Transcriptional regulator
COG4957	1192	1583	734	1459	1377	1351	1770	1632	1572	2390	1659	2097	1144	1322	1512	1565	1149	1277	Transcriptional regulator
COG5499	954	1678	2899	1573	639	648	3246	1211	1502	1930	1787	2247	1315	1845	792	2483	2966	1931	Transcriptional regulator
COG5631	332	220	68	99	98	54	733	336	698	290	384	453	74	73	97	203	109	98	Transcriptional regulator
COG4753	14286	10554	6213	5019	4885	5569	7260	5358	5352	7688	6554	7161	5068	5668	6219	10666	12285	10850	Transcriptional regulator (AraC family)
COG1329	3704	3888	1958	2971	3176	3596	2464	3170	2913	2823	2604	2971	3056	2963	3337	2715	2496	3220	Transcriptional regulator (CarD family)
COG1709	1476	2186	1122	5168	4601	4949	1971	4640	3524	2345	5571	2080	4775	4592	4037	1220	1023	1677	Transcriptional regulator (XRE family
COG4800	0	0	0	0	0	0	1	0	0	2	0	2	0	1	0	0	0	0	Transcriptional regulator (XRE family
COG5606	2926	2611	1993	6482	8412	7734	6314	8519	7045	5378	6236	6922	7625	6145	8270	3656	3547	2671	Transcriptional regulator (XRE family
COG2002	5744	3831	1514	6949	8665	8187	6392	8515	7268	6780	6420	7128	8000	6367	8755	3508	4166	3166	Transcriptional regulator AbrB family
COG2207	71904	90339	135697	72365	80894	79630	117228	91408	90174	96898	94212	107278	76559	79269	88348	117077	117280	93742	Transcriptional Regulator AraC Family
COG1522	39354	49035	44939	76361	71205	75024	55603	76266	67537	53174	88482	51734	71729	68292	65962	46446	40905	43213	transcriptional regulator AsnC family
COG3357	0	0	0	1	4	11	4	3	2	6	4	6	2	2	4	1	4	2	transcriptional regulator containing an hth domain fused to a
COG1349	26923	32775	33780	21139	27473	24899	24587	26893	24845	21557	22612	24433	24668	20546	27684	25466	24892	26409	transcriptional regulator DeoR family
COG3620	6360	7330	6822	8694	9882	9409	10072	10308	9366	8896	9191	9673	9535	8907	10453	6861	6525	6239	transcriptional regulator XRE family
COG0217	8233	8713	7385	11534	12659	12500	12293	12958	12464	11440	11709	13182	12270	11434	13514	9561	8944	8406	transcriptional regulatory protein
COG4565	7056	8474	6733	647	727	684	1268	680	872	1438	783	977	628	721	698	4853	4755	7088	transcriptional regulatory protein
COG1937	7567	8129	6388	4363	3767	4114	4459	3661	4236	4482	3797	4994	3891	4515	4082	6789	6459	7696	Transcriptional repressor
COG1197	8516	8167	5954	10913	12055	11698	11602	12280	11785	10800	10727	12315	11638	10725	12612	7964	7661	7020	transcriptioN-repair coupling factor
COG5314	866	795	409	549	477	411	1819	1018	1369	1082	1194	1230	393	442	513	737	551	484	transfer protein trbl
COG3846	1446	1346	756	1410	1453	1479	2384	1768	2234	2064	2038	2281	1416	1502	1644	1303	959	1065	transfer protein trbl
COG0663	9937	9878	9356	17700	16341	16433	15891	17255	16309	14841	17605	15962	16827	16884	16453	11788	11886	10163	Transferase
COG1819	19584	20352	8379	41000	49933	47401	35479	47531	40933	31627	38650	37699	46449	38671	49763	15886	15697	14179	Transferase
COG2977	737	1104	2883	144	182	201	176	187	263	364	225	329	162	200	256	1949	2396	1755	Transferase
COG0746	6757	7154	5630	15129	15708	15706	12562	15854	14246	11443	15207	12955	15611	14179	15628	6853	7205	6320	Transfers a GMP moiety from GTP to Mo-molybdopterin (Mo- MPT) cofactor (Moco or molybdenum cofactor) to form Mo- molybdopterin guanine dinucleotide (Mo-MGD) cofactor (By similarity)
COG0809	6017	5463	4519	10802	11977	11537	11749	12327	11864	10758	10834	12507	11623	10886	12621	6420	6306	5057	Transfers and isomerizes the ribose moiety from AdoMet to the 7-aminomethyl group of 7-deazaguanine (preQ1-tRNA) to give epoxyqueuosine (oQ-tRNA) (By similarity)
COG0736	7633	7505	5575	9825	11712	11384	10910	11828	10895	9544	10127	11333	10890	9399	12143	5870	5848	5693	Transfers the 4'-phosphopantetheine moiety from coenzyme A to a Ser of acyl-carrier-protein (By similarity)
COG0815	7122	6554	5120	14539	17468	16018	15561	17531	16121	13460	14526	16163	16274	13759	17979	7405	7270	5521	Transfers the fatty acyl group on membrane lipoproteins (By similarity)
COG0682	11942	12354	7991	17595	20783	19764	18233	20604	19000	16075	17064	19165	19501	16834	21468	11190	10911	10078	Transfers the N-acyl diglyceride group on what will become the N-terminal cysteine of membrane lipoproteins (By similarity)
COG2988	836	1145	2881	367	349	352	881	495	869	555	601	602	284	320	326	2025	1714	1706	Transforms N(2)-succinylglutamate into succinate and glutamate (By similarity)
COG1305	22196	24293	15101	31163	34624	33769	33440	34596	32205	29848	30419	34364	33576	30200	36534	22871	21994	20969	transglutaminase domain-containing protein
COG0741	17598	17061	15231	29238	33290	32439	32724	35438	32366	28907	31184	32914	31143	27681	34837	16988	15497	13968	transglycosylase
COG2261	7565	11258	8055	2171	2359	2278	2932	2329	2904	2947	2735	3252	2129	2257	2390	8021	6307	9379	Transglycosylase-associated protein
COG0021	9412	9576	10081	13942	14429	14451	14671	14819	14414	12337	12934	14863	14522	13730	15057	9058	8534	8405	Transketolase
COG3958	1605	1928	2314	1405	1475	1426	3094	1870	1826	2176	2186	2466	1638	1689	1600	1861	1828	1133	Transketolase
COG3959	1605	1928	2314	1407	1476	1427	3095	1873	1825	2183	2188	2468	1639	1690	1601	1863	1829	1133	Transketolase
COG0023	1277	1889	3294	7189	5777	6381	4500	6081	5226	4580	7545	4491	6390	6853	5591	2924	2680	2242	translation initiation factor
COG1093	123	73	20	5112	4486	4890	1841	4544	3356	2200	5446	1879	4697	4422	3922	93	77	37	translation initiation factor
COG1184	124	73	20	5132	4498	4899	1860	4561	3369	2214	5464	1906	4725	4443	3932	94	79	40	translation initiation factor
COG1601	123	73	20	5112	4486	4890	1841	4544	3356	2200	5446	1879	4697	4422	3922	93	77	37	translation Initiation Factor
COG5257	123	73	20	5112	4486	4890	1841	4544	3356	2200	5446	1879	4697	4422	3922	93	77	37	translation initiation factor
COG2178	272	236	81	636	792	808	370	477	482	356	439	495	726	632	730	224	190	157	translin
COG5232	123	73	20	5111	4486	4888	1841	4543	3356	2200	5445	1879	4696	4420	3921	93	77	37	translocation protein
COG5407	8	5	3	4	4	4	4	6	2	10	12	46	1	7	8	20	11	13	translocation protein SEC63
COG4125	879	1138	2921	145	107	93	776	377	750	520	658	579	116	140	110	1996	2414	1733	transmembrane pair domain-containing protein
COG5548	242	191	93	1153	380	455	1067	414	782	569	447	982	820	1137	532	233	316	227	Transmembrane protein 14
COG2119	1255	1404	1761	3757	1208	1429	4125	1808	2839	2839	2353	3671	2698	4135	1440	2530	2335	2177	transmembrane protein 165
COG5594	0	0	0	0	0	0	0	0	0	0	0	0	0	0	0	0	0	0	transmembrane protein 63C
COG3169	862	873	386	1792	1848	2061	1738	1903	1820	2158	2132	2498	1794	1860	2197	973	848	733	transmembrane signal peptide protein
COG3158	2438	3464	5480	2100	1900	1802	4016	2876	3106	3829	3591	4003	1805	2530	2175	4136	3497	3221	Transport of potassium into the cell (By similarity)
COG1133	310	592	1611	23	8	20	109	70	43	81	49	93	29	22	25	343	331	339	transport protein
COG2985	759	858	1901	890	972	1040	1037	843	840	908	819	1081	890	725	1151	790	916	819	transport protein
COG5047	0	0	0	0	0	0	0	0	0	0	0	0	0	0	0	0	0	0	transport protein
COG3225	1197	1604	1855	2836	1999	2279	3275	2606	2835	2827	2508	3586	2502	3250	2303	2535	2327	1910	transport system
COG2823	16591	14966	12582	34548	43892	40836	37577	44940	39421	32024	36733	38162	39913	32883	44568	17907	17808	12722	Transport-associated protein
COG0471	4903	5797	5436	5344	4267	4660	5321	4562	5379	5705	4916	6139	5565	6145	4905	4347	4055	4579	Transporter
COG0733	4384	3771	2775	976	871	932	1244	958	952	2345	1180	1194	913	1087	1072	3844	4010	4510	Transporter
COG0738	15261	16700	12273	24449	30875	28795	28293	30916	27514	23706	25630	28357	28139	24465	31618	14917	15348	11988	Transporter
COG1292	6152	5400	5871	1048	1205	1140	1440	1180	1634	2662	1314	1528	1233	1100	1149	6075	6101	6841	Transporter
COG2223	16311	19872	21641	17083	17386	17260	17988	18717	18044	16044	18945	16915	17193	16067	17687	20069	17781	19498	Transporter
COG2271	47271	55960	73343	72303	83140	82052	72971	85953	75921	63323	77574	69208	77906	67869	83964	62031	56863	56010	transporter
COG2978	1930	983	1543	515	517	625	624	629	618	1309	632	706	476	598	697	823	1167	1217	Transporter
COG3104	2314	2796	2930	2534	2321	2439	4406	3154	3409	3735	3348	4183	2234	2651	2907	2462	2760	2095	transporter
COG4594	360	482	1432	4	4	3	24	14	13	24	34	25	5	9	5	327	377	507	Transporter
COG1176	9724	13554	21487	12027	10812	11849	11318	12063	11510	10535	13441	10662	11121	11377	10818	15693	13857	13714	transporter activity
COG1653	72443	83836	54065	37294	37688	38890	34009	32771	36181	30718	37320	34852	37903	39323	36469	57361	52003	63482	transporter activity
COG3689	1423	730	305	1241	501	542	1171	478	896	968	530	1117	858	1213	580	647	945	858	transporter substrate-binding protein
COG3101	204	370	1426	6	4	2	3	9	3	14	4	5	1	16	1	227	228	293	-transporting atpase
COG0675	17888	13006	6155	61216	37209	39267	43353	28716	45885	31055	32814	48889	54905	70845	38615	12528	15089	9737	Transposase
COG1943	16501	13847	7163	37883	38382	36023	38338	38308	37421	31582	31656	39225	40106	36410	39538	15908	17512	12011	transposase
COG2826	30835	46249	25412	9056	10047	9564	10426	10491	10568	8399	9164	10054	9644	8816	10377	26549	21234	35910	transposase
COG2963	23738	31735	24084	8348	8407	9056	15290	11436	15345	9963	11452	13585	8082	8778	9079	22421	17905	26660	transposase
COG3039	3926	3837	2355	7742	9282	9193	10352	10820	10161	8588	9815	9836	8775	7377	10534	5266	4366	3624	Transposase
COG3293	17671	15782	9036	30366	23262	23765	30112	27971	26895	24434	22883	36816	25580	33346	23553	14346	13670	12749	transposase
COG3316	7880	7191	4197	14173	16383	16722	12251	12950	12974	12198	12552	15497	15464	15647	16402	7679	6536	5719	transposase
COG3328	16768	22636	10983	6819	6172	6315	12081	7438	8036	8153	7931	9216	5914	6525	6720	13587	11145	17498	transposase
COG3335	2184	2481	1793	6989	2633	2931	7840	3530	5035	4492	3668	7141	4926	7355	3001	2048	2070	1559	transposase
COG3385	2269	1580	1760	1451	806	760	2515	1221	1517	2269	1440	2017	1191	1631	772	2578	2693	2402	transposase
COG3436	7826	9216	16011	8986	9463	10044	10563	9639	9916	10520	9523	11175	9030	8289	10755	10902	8582	10449	Transposase
COG3464	13516	12971	7341	19509	19008	18785	17869	16861	18379	15491	14582	19412	21031	19730	19458	12805	12823	12443	transposase
COG3547	52205	61217	30099	48522	58283	55625	59488	60241	58370	45835	50928	55035	54572	46342	59282	42672	38554	45896	Transposase
COG3666	8227	7777	4294	7242	7563	7763	10055	7723	7597	9584	8408	9804	6918	6889	8343	9251	7742	8300	transposase
COG4584	13206	16262	7537	6287	5803	6719	9128	7321	7016	8785	7579	7956	6094	6210	6818	10682	9237	13489	Transposase
COG4644	2564	2250	2095	2946	2553	2665	4964	3254	3758	3433	3418	4528	2340	2643	2516	2274	1810	1723	Transposase
COG5421	3644	4006	1265	14897	8760	8853	9154	6849	10815	7807	6840	11484	16208	16674	8592	3544	4126	3517	transposase
COG5433	2565	2309	925	6837	4389	4711	9301	4834	5706	6051	5773	7538	6395	7304	5036	3148	3042	2129	Transposase
COG5444	3664	1030	148	45	51	54	78	49	72	1556	72	61	29	32	40	969	1849	1550	transposase
COG5464	2854	3470	8836	9023	2165	2575	8440	3778	6484	5126	4488	7878	6557	10460	2982	2616	2962	2892	transposase
COG5558	0	0	0	0	0	0	0	0	0	0	0	0	0	0	0	0	0	0	Transposase
COG5659	4488	4541	2102	8593	5204	5514	7694	4764	6871	5438	4821	8106	7524	9322	5337	3434	3171	3598	transposase
COG1593	9535	9689	7739	3322	3151	3207	7203	4965	7872	7051	6681	7057	2862	3193	2979	3576	9798	7865	9436 trap dicarboxylate transporter dctm subunit
COG4664	1595	1559	829	1895	1195	1211	2776	1870	3133	2434	2527	2957	1557	1998	1439	1546	947	910	trap dicarboxylate transporter dctm subunit
COG1626	3001	3049	3652	6444	8302	7690	7034	8532	7336	5827	6626	7005	7523	6228	8548	2573	2714	1887	trehalase
COG1877	2769	3778	3215	1673	1781	1870	1988	1676	1766	1993	2052	2159	1907	1758	1916	2503	1985	2984	trehalose biosynthetic process
COG3281	1126	948	433	1460	1772	1787	1353	1277	1471	1162	1223	1637	1731	1405	1868	929	905	913	Trehalose synthase
COG4813	2759	3196	1754	641	799	804	594	581	602	872	566	664	777	804	771	1864	1824	2312	Trehalose utilization protein
COG1075	10482	12029	8517	11408	12088	11766	11340	11702	11331	10503	10963	11806	12323	11408	12763	9951	9260	10417	Triacylglycerol Lipase
COG5598	217	314	154	345	324	359	400	425	325	512	390	526	289	270	369	281	243	193	Trimethylamine methyltransferase
COG0149	10444	11115	8904	16061	16506	16601	13815	16926	15318	13133	16374	14345	16131	15164	16585	9360	8982	9130	triosephosphate isomerase
COG3090	7846	6665	5155	2707	2537	2469	6280	4051	7047	5796	5649	5930	2414	2622	2786	6872	5530	6246	Tripartite ATP-independent periplasmic transporter dctq component
COG4665	1833	1664	847	1995	1317	1312	3075	2059	3656	2547	2681	3239	1633	2078	1541	1598	965	956	Tripartite ATP-independent periplasmic transporter dctq component
COG1767	1120	1749	4321	5569	4777	5240	3170	5234	3999	3608	6720	2851	5024	4858	4348	2748	2162	2274	triphosphoribosyl-dephospho-CoA synthase
COG3273	0	0	0	1	0	2	0	0	0	0	0	0	1	2	0	0	0	0	trka-c domain protein
COG4121	948	1787	3366	1494	492	575	2617	1140	1502	1938	1713	2054	976	1613	724	2591	2304	2019	tRNA (5-methylaminomethyl-2-thiouridylate)-methyltransferase activity
COG2419	0	0	0	0	0	0	0	0	0	0	0	0	0	0	0	0	0	0	tRNA -(guanine-N1)-methyltransferase
COG2023	123	73	20	5112	4486	4890	1841	4544	3356	2200	5446	1879	4697	4422	3922	93	77	37	tRNA 5'-leader removal
COG0051	7906	7979	5945	15982	16486	16566	13407	16770	15103	12630	16130	14124	16285	15075	16471	7724	7385	6817	tRNA binding
COG5324	0	0	0	0	0	0	0	0	0	0	0	0	0	0	0	0	0	0	TRNA ligase
COG2519	4707	5108	3381	11721	13026	12875	7801	12526	10218	7757	11706	8305	12404	10567	12477	5023	5326	4573	trna methyltransferase
COG2923	427	972	2790	140	111	140	116	89	93	260	219	146	86	140	115	1832	1566	1620	tRNA processing
COG3098	386	920	2765	1	1	0	6	3	5	143	43	35	0	2	0	1791	1513	1572	tRNA pseudouridine synthase C
COG5131	123	73	20	5111	4486	4888	1841	4543	3356	2200	5445	1879	4696	4420	3921	93	77	37	tRNA thio-modification
COG4445	846	1802	3235	1408	637	640	1927	823	1235	1419	1052	1784	1059	1607	862	2436	2473	2036	TRNA-(Ms 2 io 6 A)-hydroxylase
COG2517	0	0	0	1	0	2	0	0	0	0	0	0	1	2	0	0	0	0	tRNA-binding protein
COG1590	0	0	0	1	0	2	0	0	0	0	0	0	1	2	0	0	0	0	tRNA-yW synthesizing protein 3 homolog (S. cerevisiae)
COG3033	478	395	381	1850	2011	2400	1221	1921	1567	1093	1402	1380	2000	1948	2109	389	546	248	tryptophanase EC 4.1.99.1
COG0180	8466	8368	6159	16095	16630	16647	13924	16993	15409	12894	16486	14388	16363	15199	16573	8078	7705	7081	tryptophanyltRNA synthetase
COG3476	3519	5322	2622	1737	1182	1223	1629	1229	1748	1503	1451	1952	1553	1973	1418	3333	2782	3878	tspo and mbr like protein
COG2805	6589	5763	4448	14897	15286	15944	13231	14446	13848	12888	12977	15001	15326	14553	17050	7041	7024	6244	twitching motility protein
COG5008	1056	758	359	1376	881	1014	2253	1582	1634	2243	2541	2240	1097	1241	1053	1164	852	719	twitching motility protein
COG0710	1192	1218	1661	7863	7522	8374	3723	7202	5657	3871	7474	4078	7707	6975	7034	1150	1195	976	Type I DHQase
COG1524	11332	11289	7328	19228	23889	23198	20749	23384	20862	18423	19355	21551	22018	18776	25061	10998	11424	9120	type i phosphodiesterase nucleotide pyrophosphatase
COG3379	853	832	235	4008	3183	3706	2467	2957	3193	2299	2461	3016	4230	4479	3382	857	879	651	type i phosphodiesterase nucleotide pyrophosphatase
COG4748	557	840	1719	1098	551	601	956	686	998	1050	894	1137	896	1471	689	1887	1768	1463	type I restriction enzyme
COG0286	8979	9426	6705	17262	15688	15916	16212	15905	16318	15114	15206	17864	16917	18019	16898	9299	9161	7694	type I restriction-modification system
COG4096	1347	1861	1884	2590	2046	2267	2356	2071	2722	2496	2363	2967	2709	3149	2405	2397	2274	2015	type I restriction-modification system R subunit
COG0610	4790	3724	1415	9673	10719	10297	9686	10784	9880	8950	9214	10461	10254	9352	11137	3899	4014	2611	Type I site-specific deoxyribonuclease
COG4796	1637	1735	3166	2167	998	1200	3013	1980	2115	2690	2896	2807	1510	2360	1260	3042	2617	2440	Type II and III secretion system protein
COG4964	4044	4073	2849	7178	9163	8345	8372	9463	8937	7181	7844	8309	8308	6576	9218	4985	4308	3555	Type II and III secretion system protein
COG1459	9640	8547	7751	16896	17951	18909	17868	18702	17527	17137	17676	18768	17990	16508	19667	12375	11650	10288	Type ii secretion system
COG2064	6256	8620	5349	3519	3831	4290	4072	3964	4174	3376	3756	3882	3877	3548	4050	6585	6229	7513	type ii secretion system
COG4965	11044	14400	8567	4796	5322	5838	5323	5109	5939	4221	4770	5168	5280	4907	5480	9948	9326	11757	Type ii secretion system
COG0630	810	553	215	10582	9260	10091	4706	9791	7738	5196	11610	4754	9636	9127	8211	581	413	332	type II secretion system protein
COG2804	10636	9341	8104	19325	19143	20354	20748	20409	19597	19922	20528	21488	19752	19202	21241	13622	12501	11174	type ii secretion system protein e
COG3156	1544	2218	3200	1848	1820	2026	2656	2302	2319	2922	2679	2917	1922	1824	2223	4593	3690	3608	type ii secretion system protein k
COG3421	15	25	3	14	28	30	8	12	13	18	12	12	20	14	23	10	7	9	type iii
COG3587	394	212	41	503	618	588	533	481	566	474	472	601	678	599	581	198	165	170	type iii
COG1061	7592	8317	5470	12684	9651	10500	7994	9057	9484	7897	10426	8797	11777	13080	9541	5988	5674	6448	type iii restriction protein res subunit
COG3886	76	69	11	272	277	308	151	164	199	214	207	229	231	267	288	73	39	133	type iii restriction protein res subunit
COG4669	310	174	91	133	133	108	614	330	709	217	350	455	127	135	129	144	85	197	type iii secretion
COG4789	308	171	90	133	132	108	613	329	708	217	349	453	127	135	128	138	84	195	Type III secretion
COG4791	313	174	92	143	156	136	618	339	720	222	354	452	145	143	151	142	88	193	type iii secretion
COG4792	249	126	46	69	74	52	332	184	511	78	127	268	59	57	56	69	46	45	type iii secretion
COG4790	308	170	90	133	132	108	613	328	707	216	347	453	127	134	128	138	84	195	Type III secretion system protein
COG4972	5127	4410	3893	10062	11059	10806	9952	11030	10070	9135	9554	10418	10927	9551	11577	5543	5524	4701	type IV pilus assembly protein pilm
COG4968	3232	2432	1989	7187	5614	6868	7096	6437	6447	6487	7184	7195	6223	7252	7323	3792	3428	3105	Type IV pilus biogenesis protein PilE
COG3063	31172	27403	14600	84585	93571	91879	74561	91096	81725	67198	75432	80913	92625	81662	96085	32580	32686	24667	type IV pilus biogenesis stability protein PilW
COG4967	2194	1898	3162	1443	966	1145	2338	1672	1650	2856	2676	2374	1047	1382	1322	3181	2777	2662	type IV pilus modification protein PilV
COG3451	7860	8405	7608	9947	10914	10149	15144	12640	13101	11173	11760	13383	10434	9935	11400	7941	7119	6561	type IV secretion system protein
COG3702	545	429	157	292	214	206	803	582	964	646	540	866	169	205	231	361	290	266	type IV secretion system protein
COG3704	817	568	323	523	402	340	1709	1279	1698	1248	1168	1791	329	394	406	575	422	425	type IV secretion system protein
COG3736	699	531	227	416	292	266	1472	1019	1431	1026	987	1412	231	300	327	519	364	355	Type IV secretion system protein VirB8
COG3521	1034	1525	4283	211	135	132	1129	516	536	752	861	574	157	192	161	2325	2686	2084	Type VI secretion
COG3519	1798	2549	5831	803	707	769	2562	1411	1816	1707	1808	1713	746	689	974	4431	3746	3730	type VI secretion protein
COG3523	2050	2941	7257	885	775	821	2385	1381	1850	1732	1804	1811	780	744	1007	4671	3975	4038	Type VI secretion protein (IcmF)
COG3515	1927	2877	7204	745	678	734	2097	1184	1593	1577	1604	1531	697	635	913	4617	3926	3989	type VI secretion-associated protein
COG4499	1514	425	68	36	40	36	51	30	44	661	56	56	22	31	36	414	764	646	Type VII secretion protein EssB
COG5350	390	499	308	470	570	527	476	462	480	509	485	643	466	353	650	524	413	371	Tyrosine phosphatase
COG3663	3906	3213	2870	4967	6528	5454	6598	6834	6199	5561	5900	6103	5628	4402	6499	2908	3228	2303	U mismatch-specific DNA glycosylase
COG2136	0	0	0	0	0	0	0	0	0	0	0	0	0	0	0	0	0	0	u3 small nucleolar ribonucleoprotein
COG5384	0	0	0	0	0	0	0	0	0	0	0	0	0	0	0	0	0	0	u3 small nucleolar ribonucleoprotein
COG1179	2966	2862	4950	1964	1765	1958	3643	2496	3006	3462	3050	3441	1722	2118	2212	3608	3658	3334	uba thif-type nad fad binding protein
COG4015	0	0	0	0	0	0	0	0	0	0	0	0	0	0	0	0	0	0	UBA THIF-type NAD FAD binding protein
COG5452	425	594	330	491	475	454	659	591	557	812	604	784	388	432	540	572	407	411	Ubiquinol-cytochrome C chaperone
COG5590	318	303	133	450	410	396	689	687	680	843	686	901	329	371	461	325	276	292	ubiquinone biosynthesis protein Coq9
COG5272	29	16	6	46	50	65	19	38	35	28	34	30	44	35	38	12	15	18	ubiquitin
COG5077	4	3	0	8	8	12	12	15	14	17	16	23	20	9	24	2	1	1	ubiquitin carboxyl-terminal hydrolase
COG5207	329	246	159	104	152	169	250	168	199	139	190	218	139	155	200	122	103	133	ubiquitin carboxyl-terminal hydrolase
COG5533	0	0	0	0	0	0	0	0	0	0	0	0	0	0	0	0	0	0	ubiquitin carboxyl-terminal hydrolase
COG5560	0	0	0	0	0	0	1	1	1	2	0	0	0	2	0	0	0	0	ubiquitin carboxyl-terminal hydrolase
COG5113	0	0	0	0	0	0	0	0	0	0	0	0	0	0	0	0	0	0	Ubiquitin conjugation factor E4
COG5140	9	3	2	1	2	1	2	2	5	2	3	5	1	1	1	2	2	1	Ubiquitin fusion degradation protein
COG5021	0	0	0	0	0	0	0	0	0	0	0	0	0	0	0	0	0	0	ubiquitin protein ligase
COG5078	142	85	26	5142	4542	4953	1858	4578	3382	2236	5466	1910	4752	4467	3985	112	94	54	ubiquitin-conjugating enzyme
COG0562	2153	2793	1364	1168	1322	1492	1226	1265	1364	1395	1232	1651	1196	1421	1710	1690	1477	2107	UDP-galactopyranose mutase
COG1087	8688	9007	6833	17625	17728	17902	14396	17603	15945	13867	17252	15317	18132	17321	17892	7572	7106	6335	udp-glucose 4-epimerase
COG1004	11536	11781	9779	24632	27149	26904	21286	26714	23603	19563	23809	22199	25964	23150	27258	13751	12814	11098	UDP-glucose 6-dehydrogenase
COG4468	151	92	20	14	12	14	12	9	13	33	23	19	11	14	12	118	119	109	UDP-glucose-hexose-1-phosphate uridylyltransferase
COG0381	7182	7261	5730	9978	11015	10649	9808	10892	10398	8946	9238	10374	10645	9713	11153	6936	6887	6370	UDP-N-acetylglucosamine 2-epimerase
COG0528	8040	8112	5999	16052	16610	16632	13511	16864	15201	12753	16203	14286	16384	15191	16584	7922	7494	6943	UMP kinase activity
COG2886	334	284	97	7503	4820	5283	3427	5008	4843	3121	6220	3445	6507	7284	4326	254	362	191	Uncharacterised protein family (UPF0175)
COG1615	2368	2787	1508	6398	4988	5434	3484	5199	4862	2881	6165	3161	5632	5825	4460	1556	1353	1982	Uncharacterised protein family (UPF0182)
COG1745	0	0	0	0	0	0	0	0	0	0	0	0	0	0	0	0	0	0	Uncharacterised protein family UPF0058
COG3361	4184	4628	2121	6499	8597	7979	6697	8394	7245	5692	6432	6996	7682	6189	8805	3320	3649	3214	Uncharacterized conserved protein (COG2071)
COG3410	96	64	35	28	39	23	51	35	49	79	69	91	29	40	26	46	78	59	Uncharacterized conserved protein (DUF2075)
COG4628	616	1172	1777	774	609	770	1559	989	916	1685	1586	1427	690	1030	930	2256	2012	1549	Uncharacterized conserved protein (DUF2132)
COG4744	441	370	138	827	935	999	507	720	630	514	568	859	1074	829	938	412	452	433	Uncharacterized conserved protein (DUF2149)
COG5482	1004	567	254	101	120	101	138	114	109	464	117	179	91	98	120	521	604	508	Uncharacterized conserved protein (DUF2161)
COG5460	2946	2451	2061	4129	5836	4739	4689	5735	4914	4471	4540	4630	5061	3636	5744	3429	3432	2698	Uncharacterized conserved protein (DUF2164)
COG4831	61	42	13	121	134	162	122	166	177	128	116	160	127	99	167	51	38	44	Uncharacterized conserved protein (DUF2173)
COG4895	944	376	56	144	133	88	678	260	266	796	384	510	160	290	179	439	666	479	Uncharacterized conserved protein (DUF2196)
COG4911	2329	1845	531	6110	8079	7443	5538	7655	6476	4978	5730	5992	7285	5676	8200	1865	1943	1276	Uncharacterized conserved protein (DUF2203)
COG4309	570	458	170	718	773	778	821	720	853	881	899	1003	928	880	795	511	427	358	Uncharacterized conserved protein (DUF2249)
COG5502	394	444	295	1267	598	558	1165	539	982	891	646	1326	941	1540	692	448	330	410	Uncharacterized conserved protein (DUF2267)
COG5552	2408	3115	1600	2246	2530	2904	1578	2227	1974	1393	1704	1783	2464	2325	2575	1969	1717	2362	Uncharacterized conserved protein (DUF2277)
COG5634	227	198	179	191	147	177	371	355	299	137	260	347	120	239	113	118	134	91	Uncharacterized conserved protein (DUF2278)
COG5419	321	226	76	127	99	81	490	259	615	279	231	383	69	95	123	144	137	120	Uncharacterized conserved protein (DUF2285)
COG5619	3	1	1	2	3	1	2	2	0	4	2	9	0	6	0	3	2	10	Uncharacterized conserved protein (DUF2290)
COG5586	94	103	47	62	75	58	59	59	57	88	51	90	59	66	67	100	67	64	Uncharacterized conserved protein (DUF2293)
COG5609	1240	622	189	1460	952	1084	608	566	900	957	625	827	1630	1712	934	571	844	891	Uncharacterized conserved protein (DUF2294)
COG5440	0	0	0	0	0	0	0	0	0	0	0	0	0	0	0	0	0	0	Uncharacterized conserved protein (DUF2299)
COG5532	7	8	2	2	2	1	13	8	5	18	4	9	0	3	4	9	2	18	Uncharacterized conserved protein (DUF2303)
COG2456	14	5	3	14	24	20	14	19	23	16	14	25	8	27	17	11	10	17	Uncharacterized conserved protein (DUF2304)
COG3286	0	0	0	0	0	0	0	0	0	0	0	0	0	0	0	0	0	0	Uncharacterized protein conserved in archaea (DUF2067)
COG4003	123	73	20	5111	4486	4888	1841	4543	3356	2200	5445	1879	4696	4420	3921	93	77	37	Uncharacterized protein conserved in archaea (DUF2095)
COG4013	0	0	0	0	0	0	0	0	0	0	0	0	0	0	0	0	0	0	Uncharacterized protein conserved in archaea (DUF2097)
COG4014	0	0	0	0	0	0	0	0	0	0	0	0	0	0	0	0	0	0	Uncharacterized protein conserved in archaea (DUF2098)
COG4024	0	0	0	0	0	0	0	0	0	0	0	0	0	0	0	0	0	0	Uncharacterized protein conserved in archaea (DUF2100)
COG4069	0	0	0	0	0	0	0	0	0	0	0	0	0	0	0	0	0	0	Uncharacterized protein conserved in archaea (DUF2117)
COG4072	0	0	0	0	0	0	0	0	0	0	0	0	0	0	0	0	0	0	Uncharacterized protein conserved in archaea (DUF2118)
COG4073	0	0	0	0	0	0	0	0	0	0	0	0	0	0	0	0	0	0	Uncharacterized protein conserved in archaea (DUF2119)
COG4090	0	0	0	0	0	0	0	0	0	0	0	0	0	0	0	0	0	0	Uncharacterized protein conserved in archaea (DUF2124)
COG4697	0	0	0	1	0	2	0	0	0	0	0	0	1	2	0	0	0	0	Uncharacterized protein conserved in archaea (DUF2139)
COG4749	0	0	0	0	0	0	0	0	0	0	0	0	0	0	0	0	0	0	Uncharacterized protein conserved in archaea (DUF2150)
COG4755	0	0	0	0	0	0	0	0	0	0	0	0	0	0	0	0	0	0	Uncharacterized protein conserved in archaea (DUF2153)
COG4847	0	0	0	0	0	0	0	0	0	0	0	0	0	0	0	0	0	0	Uncharacterized protein conserved in archaea (DUF2175)
COG4879	0	0	0	0	0	0	0	0	0	0	0	0	0	0	0	0	0	0	Uncharacterized protein conserved in archaea (DUF2192)
COG4883	0	0	0	0	0	0	0	0	0	0	0	0	0	0	0	0	0	0	Uncharacterized protein conserved in archaea (DUF2193)
COG4921	0	0	0	0	0	0	0	0	0	0	0	0	0	0	0	0	0	0	Uncharacterized protein conserved in archaea (DUF2209)
COG1667	0	0	0	0	0	0	0	0	0	0	0	0	0	0	0	0	0	0	Uncharacterized protein conserved in archaea (DUF2226)
COG3612	0	0	0	1	0	2	0	0	0	0	0	0	1	2	0	0	0	0	Uncharacterized protein conserved in archaea (DUF2240)
COG4344	0	0	0	1	0	2	0	0	0	0	0	0	1	2	0	0	0	0	Uncharacterized protein conserved in archaea (DUF2250)
COG5399	0	0	0	0	0	0	0	0	0	0	0	0	0	0	0	0	0	0	Uncharacterized protein conserved in archaea (DUF2286)
COG3184	563	1035	1658	352	311	293	536	466	585	662	458	691	319	486	438	2135	1550	1513	Uncharacterized protein conserved in bacteria (DUF2059)
COG3216	1423	2056	2227	2934	2167	2510	2757	2401	2580	2775	2381	3324	2652	3085	2559	2840	2512	2299	Uncharacterized protein conserved in bacteria (DUF2062)
COG3219	585	940	1730	208	96	78	669	365	783	415	354	612	102	141	120	1722	1415	1359	Uncharacterized protein conserved in bacteria (DUF2063)
COG3222	590	705	152	1765	1044	1182	1903	1337	1762	1520	1333	2143	1625	2167	1334	533	553	346	Uncharacterized protein conserved in bacteria (DUF2064)
COG3249	452	791	1485	357	327	319	421	441	514	522	367	584	289	268	369	1864	1472	1448	Uncharacterized protein conserved in bacteria (DUF2066)
COG3908	430	589	326	457	438	412	599	522	508	788	568	692	338	389	488	565	392	416	Uncharacterized protein conserved in bacteria (DUF2093)
COG4093	288	411	263	350	387	328	444	398	405	579	462	539	281	332	426	398	240	256	Uncharacterized protein conserved in bacteria (DUF2125)
COG4487	319	400	128	461	596	534	757	616	706	684	759	807	704	617	640	517	401	311	Uncharacterized protein conserved in bacteria (DUF2130)
COG4685	3	2	26	0	0	0	1	0	0	1	6	1	0	0	0	2	8	19	Uncharacterized protein conserved in bacteria (DUF2138)
COG4698	752	232	54	12	12	12	19	12	19	326	17	18	8	8	12	205	442	443	Uncharacterized protein conserved in bacteria (DUF2140)
COG4704	841	1042	252	3056	2505	2904	2690	2706	2638	2337	2451	2750	2846	3190	2703	948	946	589	Uncharacterized protein conserved in bacteria (DUF2141)
COG4727	204	170	73	177	87	94	551	234	227	574	662	376	98	155	135	310	205	141	Uncharacterized protein conserved in bacteria (DUF2145)
COG4765	441	592	338	504	500	468	663	609	567	805	625	797	414	463	561	570	401	409	Uncharacterized protein conserved in bacteria (DUF2155)
COG3789	348	710	1548	23	29	24	108	54	54	245	98	119	18	29	41	1647	1377	1332	Uncharacterized protein conserved in bacteria (DUF2170)
COG3798	506	846	1716	263	272	257	366	320	288	481	272	442	242	262	284	661	538	620	Uncharacterized protein conserved in bacteria (DUF2171)
COG4843	860	296	72	48	43	34	53	40	41	340	47	58	46	45	46	290	525	514	Uncharacterized protein conserved in bacteria (DUF2179)
COG4861	293	232	133	71	61	75	290	148	207	88	161	125	54	51	47	89	102	110	Uncharacterized protein conserved in bacteria (DUF2186)
COG4873	24	29	27	5	5	3	3	3	4	19	3	6	4	3	5	16	78	143	Uncharacterized protein conserved in bacteria (DUF2187)
COG4876	135	87	16	3	2	2	13	4	5	11	21	8	2	4	5	98	111	95	Uncharacterized protein conserved in bacteria (DUF2188)
COG4878	1	0	1	0	0	0	0	0	0	0	0	0	0	0	0	1	5	4	Uncharacterized protein conserved in bacteria (DUF2194)
COG4893	2	3	1	0	0	0	0	0	0	1	0	0	0	0	0	1	1	1	Uncharacterized protein conserved in bacteria (DUF2195)
COG4896	753	233	57	12	14	13	19	12	18	327	17	17	8	9	12	208	449	455	Uncharacterized protein conserved in bacteria (DUF2197)
COG4897	750	232	53	11	11	12	18	11	16	325	16	15	8	8	11	203	440	442	Uncharacterized protein conserved in bacteria (DUF2198)
COG4899	82	84	297	88	73	68	157	99	120	187	115	129	49	81	105	62	183	67	Uncharacterized protein conserved in bacteria (DUF2199)
COG3553	311	447	311	307	288	328	396	386	390	487	315	476	262	311	358	351	316	306	Uncharacterized protein conserved in bacteria (DUF2218)
COG1655	871	336	92	15	15	17	23	16	21	350	25	36	11	13	19	311	605	652	Uncharacterized protein conserved in bacteria (DUF2225)
COG3662	4203	6077	5491	1405	1669	1716	1406	1246	1591	1311	1273	1521	1504	1517	1628	4803	3920	5471	Uncharacterized protein conserved in bacteria (DUF2236)
COG3651	492	411	213	1600	786	951	1491	786	1208	1123	896	1477	1299	1683	1112	475	552	473	Uncharacterized protein conserved in bacteria (DUF2237)
COG3644	384	242	86	222	230	170	791	445	832	411	440	565	172	211	239	261	147	173	Uncharacterized protein conserved in bacteria (DUF2239)
COG4304	40	10	0	1	3	2	7	1	5	1	6	3	1	5	2	14	15	12	Uncharacterized protein conserved in bacteria (DUF2247)
COG4307	819	1292	1823	606	574	620	818	597	615	1060	901	865	569	505	721	2303	1837	1796	Uncharacterized protein conserved in bacteria (DUF2248)
COG4316	76	88	28	60	74	92	110	121	150	88	86	142	88	96	130	50	52	48	Uncharacterized protein conserved in bacteria (DUF2251)
COG4320	2906	2367	935	6717	8454	7827	7292	8493	7456	5613	6517	7105	7565	5983	8584	2260	2411	1672	Uncharacterized protein conserved in bacteria (DUF2252)
COG4296	3	1	1	4	1	2	1	2	1	10	3	8	2	3	2	9	1	1	Uncharacterized protein conserved in bacteria (DUF2262)
COG4322	292	445	1422	112	91	83	309	154	156	336	277	276	72	110	133	312	284	372	Uncharacterized protein conserved in bacteria (DUF2272)
COG3779	213	221	396	265	311	336	246	227	252	220	192	318	290	155	396	231	407	212	Uncharacterized protein conserved in bacteria (DUF2314)
COG4367	0	0	0	0	0	0	0	0	0	0	0	0	0	0	0	0	0	0	Uncharacterized protein conserved in bacteria (DUF2316)
COG5338	549	731	363	850	872	947	1019	1051	989	1350	1098	1308	769	1002	1107	760	509	518	Uncharacterized protein conserved in bacteria (DUF2320)
COG4306	44	32	8	48	52	77	69	86	110	51	60	84	60	68	113	22	24	33	Uncharacterized protein conserved in bacteria (DUF2321)
COG4390	49	21	12	98	35	42	358	165	215	203	217	166	42	74	67	26	20	15	Uncharacterized protein conserved in bacteria (DUF2322)
COG4378	1032	408	71	289	326	295	534	372	708	639	323	542	446	274	279	322	465	421	Uncharacterized protein conserved in bacteria (DUF2325)
COG5293	28	34	66	95	88	113	60	94	70	140	128	116	125	201	168	38	54	53	Uncharacterized protein conserved in bacteria (DUF2326)
COG5385	408	568	319	435	429	389	582	509	511	736	555	690	331	385	479	540	377	387	Uncharacterized protein conserved in bacteria (DUF2328)
COG4402	216	214	90	1337	491	616	1237	635	973	1141	790	1321	1099	1650	896	286	228	248	Uncharacterized protein conserved in bacteria (DUF2330)
COG4427	497	457	246	198	203	201	286	232	284	328	215	391	161	202	220	332	334	316	Uncharacterized protein conserved in bacteria (DUF2332)
COG5298	160	112	21	32	40	37	29	27	42	53	52	57	38	46	44	132	121	200	Uncharacterized protein conserved in bacteria (DUF2334)
COG5330	951	1239	679	1120	1270	1042	1352	1234	1239	1826	1331	1687	942	1104	1373	1490	817	918	Uncharacterized protein conserved in bacteria (DUF2336)
COG4408	86	20	1	2	2	1	7	1	5	1	12	3	2	7	3	30	34	25	Uncharacterized protein conserved in bacteria (DUF2338)
COG4924	247	134	94	134	56	40	366	197	282	214	238	271	49	77	53	180	148	94	Uncharacterized protein conserved in bacteria N-term (DUF3322)
COG4068	0	0	0	1	0	2	0	0	0	0	0	0	1	2	0	0	0	0	Uncharacterized protein containing a Zn-ribbon (DUF2116)
COG4902	71	64	26	131	130	160	283	189	147	174	180	210	149	239	180	42	52	54	Uncharacterized protein domain (DUF2202)
COG5583	1077	871	1425	82	62	59	127	77	108	549	144	118	42	66	79	1893	1802	1698	Uncharacterized small protein (DUF2292)
COG0589	41795	51295	35182	61369	62707	62082	58493	63776	58980	49785	61457	58167	62931	59006	62325	43545	41081	42988	Universal stress protein
COG1742	3540	4388	4469	1267	1301	1335	1732	1323	1433	2121	1667	1760	1358	1280	1342	4061	3677	4450	UPF0060 membrane protein
COG0397	5469	6975	5849	2385	1369	1453	3825	2410	3137	3382	2823	3707	1734	2585	1743	5472	5022	6264	UPF0061 protein
COG2976	1368	1486	3004	981	625	714	2172	1525	1929	1523	1748	1893	613	842	789	2441	2014	1983	UPF0070 protein
COG0792	6670	7079	4504	11006	12149	11950	11492	12364	11509	10185	10783	12261	11717	10876	12814	5866	5571	5065	UPF0102 protein
COG1355	824	745	197	7754	7334	8224	3672	7160	5555	3954	7538	4007	7644	7068	6887	775	782	670	UPF0103 protein
COG1786	516	357	207	96	94	74	661	340	1002	124	235	510	70	55	72	138	92	59	UPF0107 protein
COG1837	7585	6747	2632	12841	15865	14475	12024	15026	13552	10640	11561	12772	14788	11960	16262	5471	5777	5075	UPF0109 protein
COG2862	1284	1588	3007	865	723	708	1972	1358	1580	1753	1882	1894	641	877	845	2775	2100	2138	UPF0114 protein
COG2914	1117	960	1662	903	541	625	1995	1330	1634	1373	1698	1711	549	777	656	920	759	774	UPF0125 protein
COG1851	0	0	0	0	0	0	0	0	0	0	0	0	0	0	0	0	0	0	UPF0128 protein
COG0393	4449	3574	2400	7953	9816	9292	8297	9634	8530	7473	7699	8713	8732	7504	10041	4803	4700	3720	UPF0145 protein
COG1255	0	0	0	0	0	0	0	0	0	0	0	0	0	0	0	0	0	0	UPF0146 protein
COG1698	123	73	20	5112	4486	4890	1841	4544	3356	2200	5446	1879	4697	4422	3922	93	77	37	UPF0147 protein
COG3079	540	999	2841	207	152	181	294	302	390	355	247	402	156	149	187	1880	1584	1634	UPF0149 protein
COG3763	801	244	58	17	16	15	36	19	39	343	31	47	13	13	22	226	469	470	UPF0154 protein
COG4286	103	198	171	61	68	85	72	79	67	58	71	77	77	56	73	86	67	39	UPF0160 protein
COG4735	341	525	1442	1051	291	311	936	337	670	524	369	888	872	1149	294	343	363	412	UPF0174 protein
COG1054	4672	5392	5098	2656	1936	2004	4365	2914	3747	3847	3407	4248	2222	2699	2468	5158	4923	5123	UPF0176 protein
COG1671	2044	2115	3313	994	942	924	1460	1136	1107	1939	1407	1431	979	924	967	3010	2759	2774	UPF0178 protein
COG1860	0	0	0	1	0	2	0	0	0	0	0	0	1	2	0	0	0	0	UPF0179 protein
COG3140	203	370	1395	0	1	0	2	3	1	9	3	3	0	5	0	223	205	292	UPF0181 protein
COG3781	3744	4192	4728	7873	8899	8207	9205	9257	8710	7472	7798	8915	8727	7725	9082	4553	5097	3361	UPF0187 protein
COG1931	123	73	20	5112	4486	4890	1841	4544	3356	2200	5446	1879	4697	4422	3922	93	77	37	UPF0201 protein
COG3092	203	370	1395	0	1	0	2	3	1	9	4	3	0	5	0	223	205	292	UPF0208 membrane protein
COG2848	10	5	3	11	10	14	11	13	13	36	30	40	14	12	12	23	6	5	UPF0210 protein
COG1885	0	0	0	0	0	0	0	0	0	0	0	0	0	0	0	0	0	0	UPF0212 protein
COG1628	128	79	22	5137	4518	4919	1865	4564	3381	2244	5470	1916	4733	4467	3962	101	81	45	UPF0215 protein
COG2083	123	73	20	5111	4486	4888	1841	4543	3356	2200	5445	1879	4696	4420	3921	93	77	37	UPF0216 protein
COG1909	123	73	20	5112	4486	4890	1841	4544	3356	2200	5446	1879	4697	4422	3922	93	77	37	UPF0218 protein
COG4476	754	233	57	13	15	14	20	12	20	327	18	19	9	9	13	209	450	456	UPF0223 protein
COG3012	3613	4380	4691	1121	917	1015	1937	1331	1519	2175	1824	1786	936	1203	1155	4005	3781	4262	UPF0225 protein
COG3150	1131	1632	4311	508	229	257	1955	770	1119	1204	1225	1324	282	445	356	2465	2022	2206	UPF0227 protein
COG2718	2160	2004	3250	690	622	662	1789	1025	1325	1832	1515	1382	649	692	752	2999	2660	2720	UPF0229 protein
COG3112	203	366	1389	0	1	0	1	1	0	7	3	2	0	1	0	216	202	292	UPF0231 protein
COG5512	1986	2628	1430	1208	403	487	1201	508	932	644	567	1076	776	1279	423	1421	1279	1878	UPF0232 protein
COG1666	6588	6418	5089	8563	9592	9020	9383	9807	9415	7747	8291	9364	9341	8178	9900	6290	6049	5807	UPF0234 protein
COG1872	4069	3963	2618	9552	10720	10370	8824	10426	9383	8354	8868	9764	10297	9228	11108	5056	4646	3804	UPF0235 protein
COG3830	92	70	64	165	122	164	133	139	138	204	152	210	158	179	202	92	94	65	UPF0237 protein
COG3022	3012	4291	4707	777	702	746	1839	1047	1509	1479	1461	1457	704	800	775	3932	3388	3868	UPF0246 protein
COG1531	78	122	27	193	278	254	121	180	163	160	128	218	401	209	219	93	84	98	UPF0248 protein
COG3394	3641	3380	2197	7501	9602	9018	7614	9348	8556	6792	7466	8132	8843	7133	9929	4457	4201	3402	UPF0249 protein
COG2921	1242	1411	2995	842	593	658	1847	1288	1583	1344	1542	1620	594	780	674	2383	1988	2037	UPF0250 protein
COG1342	38	27	10	156	118	164	121	125	121	191	142	202	150	167	191	64	39	35	UPF0251 protein
COG4016	0	0	0	0	0	0	0	0	0	0	0	0	0	0	0	0	0	0	UPF0254 protein
COG5441	627	824	642	446	553	534	780	570	899	479	449	798	476	341	606	565	484	385	UPF0261 protein
COG5328	451	619	332	485	485	448	617	562	553	800	598	759	380	435	537	600	411	417	UPF0262 protein
COG3099	203	366	1389	0	1	0	1	1	0	8	4	2	0	1	0	216	202	292	UPF0263 protein
COG1891	109	105	34	173	118	149	267	177	173	347	278	273	123	176	175	149	87	95	UPF0264 protein
COG2926	203	366	1389	0	1	0	1	1	0	7	3	2	0	1	0	216	202	292	UPF0265 protein
COG4811	203	370	1395	0	1	0	2	3	1	9	3	3	0	5	0	223	205	292	UPF0266 membrane protein
COG3097	0	0	0	0	0	0	0	0	0	0	0	0	0	0	0	0	0	0	UPF0267 protein
COG3089	387	923	2771	1	2	0	6	5	5	145	43	38	0	6	1	1798	1516	1572	UPF0270 protein
COG1641	3244	2647	839	8868	10258	9892	7781	9445	8640	6758	7410	8477	9999	8398	10648	2738	2930	2110	UPF0272 protein
COG3220	4084	4305	4255	7832	9628	9017	8391	9669	9018	7621	8062	8733	8951	7140	10090	6380	5782	4897	UPF0276 protein
COG1458	125	175	30	381	453	461	285	368	315	301	303	399	567	379	392	160	151	134	UPF0278 protein
COG2248	0	0	0	1	0	2	0	0	0	0	0	0	1	2	0	0	0	0	UPF0282 protein
COG3768	423	716	1629	215	264	206	252	250	240	338	304	364	186	206	272	545	385	473	UPF0283 membrane protein
COG4020	0	0	0	0	0	0	0	0	0	0	0	0	0	0	0	0	0	0	UPF0285 protein
COG4070	0	0	0	0	0	0	0	1	1	0	2	0	0	2	0	0	0	0	UPF0288 protein
COG4224	992	400	90	26	26	28	33	21	33	350	44	45	19	23	25	409	650	635	UPF0291 protein
COG2052	1033	427	88	1153	307	421	1068	416	756	920	513	968	733	1229	358	442	670	522	UPF0296 protein
COG4471	892	320	71	15	17	16	25	13	23	329	28	22	10	13	16	305	555	545	UPF0298 protein
COG1678	2393	2953	3845	2659	2645	2784	4182	3325	3591	3884	3651	4350	2684	2930	3083	3884	3407	3069	UPF0301 protein
COG5582	1492	440	86	27	28	31	40	26	42	637	34	34	18	20	24	402	818	768	UPF0302 protein
COG4702	3586	4153	3111	4055	5779	4674	4879	5824	5017	3919	4479	4587	5016	3571	5666	2870	2772	2856	UPF0303 protein
COG3013	225	401	1398	5	6	12	12	8	15	22	21	18	5	7	12	230	215	301	UPF0304 protein
COG4066	0	0	0	0	0	0	0	0	0	0	0	0	0	0	0	0	0	0	UPF0305 protein
COG3787	209	371	1396	8	13	9	7	14	19	19	12	16	6	19	9	227	209	293	UPF0306 protein
COG3028	1139	1338	2961	656	378	443	1758	1123	1437	1263	1469	1506	400	606	471	2334	1914	1888	UPF0307 protein
COG4821	1323	769	344	1105	1355	1471	888	1064	1069	1271	1000	1160	1197	1334	1390	631	862	769	UPF0309 protein
COG1673	124	73	20	5111	4486	4888	1841	4543	3356	2200	5445	1880	4697	4420	3922	93	78	37	UPF0310 protein
COG4336	1982	2587	1381	194	211	128	944	455	882	375	523	560	154	181	158	1535	1215	1826	UPF0317 protein
COG3110	203	366	1420	0	1	0	1	2	1	9	4	2	0	1	0	217	225	293	UPF0319 protein
COG3750	650	836	458	727	704	623	1054	997	987	1294	979	1327	569	658	772	873	592	614	UPF0335 protein
COG3422	5136	8506	7916	281	172	156	1333	576	1007	1054	751	977	154	262	213	7113	6804	7839	UPF0339 protein
COG4475	957	364	79	267	263	297	151	154	193	489	186	200	211	246	268	358	582	578	UPF0340 protein
COG3679	1723	605	140	41	39	44	54	36	55	670	50	57	28	35	38	589	1065	1053	UPF0342 protein
COG3123	880	1288	2942	628	493	634	1276	846	763	1349	1390	1084	537	813	750	2351	2041	1985	UPF0345 protein
COG4479	753	233	56	12	14	14	19	12	19	313	17	18	8	9	13	207	444	452	UPF0346 protein
COG1323	859	315	73	23	24	26	26	20	28	353	33	42	19	19	20	311	543	537	UPF0348 protein
COG4844	1766	613	134	38	37	42	56	32	58	648	52	47	27	35	34	591	1067	1046	UPF0349 protein
COG3082	203	366	1389	0	1	0	1	1	0	7	3	2	0	1	0	216	202	292	UPF0352 protein
COG4848	786	256	99	109	87	119	96	86	69	423	169	91	87	116	104	248	504	494	UPF0354 protein
COG5503	753	232	57	13	15	14	20	12	19	327	18	19	9	9	13	208	445	452	UPF0356 protein
COG4838	892	320	71	15	16	16	25	13	22	329	28	22	10	13	15	305	555	545	UPF0358 protein
COG4864	1289	766	178	1218	1349	1536	1374	1267	1175	1845	1247	1592	1646	1544	1513	707	925	904	UPF0365 protein
COG4868	60	47	9	284	279	332	146	177	194	213	229	221	266	368	335	55	38	34	UPF0371 protein
COG3557	2646	2756	1326	387	460	508	363	323	440	605	371	447	453	447	528	1595	1656	2338	UPF0374 protein
COG3691	203	370	1395	0	1	0	2	3	1	9	4	3	0	5	0	223	205	292	UPF0381 protein
COG3811	148	257	229	108	84	82	231	144	166	254	157	190	65	100	132	161	162	105	UPF0386 protein
COG4720	955	378	102	5229	4624	5063	1900	4621	3446	2604	5555	1993	4825	4537	4091	381	578	551	UPF0397 protein
COG4474	896	324	75	26	24	32	30	22	31	323	42	29	21	25	22	321	561	548	UPF0398 protein
COG2835	3597	4659	4549	7760	6990	7603	5288	7473	6635	5424	8651	5557	7251	7051	6772	4622	3732	4520	UPF0434 protein
COG4840	891	319	70	15	16	15	24	13	21	328	27	20	10	13	14	305	553	544	UPF0435 protein
COG3085	203	366	1389	0	1	0	2	2	0	8	4	3	0	2	1	216	203	292	UPF0438 protein yifE
COG5463	602	1176	2988	191	109	101	380	258	358	428	253	407	84	136	149	1950	1628	1688	UPF0441 protein
COG3906	775	259	80	13	16	15	21	13	22	329	18	21	10	9	14	220	516	580	UPF0473 protein
COG3132	3159	3177	3472	6814	8736	8255	7180	8626	7448	6328	7158	7242	8012	6463	9072	4300	4072	3178	UPF0502 protein
COG3681	26	20	9	75	55	81	49	75	57	69	53	65	59	54	50	36	20	19	UPF0597 protein
COG4492	907	325	70	28	24	31	33	23	36	352	39	42	22	27	24	327	552	540	UPF0735 ACT domain-containing protein
COG4365	3983	2679	908	6084	8031	7286	6186	7837	6656	5940	6009	6412	7225	5779	8093	2594	3250	2030	UPF0747 protein
COG3002	524	433	192	576	560	645	705	790	606	597	595	866	546	652	625	409	328	303	UPF0753 protein
COG4399	872	323	125	990	195	213	960	309	697	908	406	937	636	1209	344	324	629	569	UPF0754 membrane protein
COG2707	1134	677	1474	23	22	23	35	21	34	347	40	35	16	27	21	549	732	727	UPF0756 membrane protein
COG4975	1766	1412	401	4050	5859	4728	4216	5621	4814	3808	4233	4534	5096	3786	5813	1383	1496	873	uptake protein
COG1573	11926	10139	3756	36182	40129	39508	30028	39926	34504	27437	36356	31725	38372	33772	40041	10583	10042	7554	uracil-dna glycosylase
COG0804	3131	3986	3328	2457	1730	1831	3727	2189	2917	2958	2809	3452	1973	2655	2074	4155	4208	4036	Urea amidohydrolase subunit alpha
COG0832	2751	3799	3254	2178	1385	1504	3516	1916	2635	2679	2492	3102	1737	2403	1773	3954	3320	3810	Urea amidohydrolase subunit beta
COG0831	3010	3988	3334	2615	1894	2019	3762	2231	2972	3008	2864	3470	2217	2867	2266	4180	3480	4020	Urea amidohydrolase subunit gamma
COG3665	5865	8574	12544	4618	4565	5461	4351	4866	4308	4006	4255	4800	4426	4738	5090	6540	5624	7495	Urea carboxylase-associated protein
COG4413	669	903	1802	517	585	603	562	577	491	591	555	723	474	481	596	2037	1912	1641	UreA transporter
COG3194	924	1191	1789	256	218	180	1242	649	1025	628	760	775	200	231	219	2048	1613	1540	Ureidoglycolate hydrolase
COG0572	5816	5962	5089	9042	9406	8948	8742	9225	8821	7448	7547	8884	9501	9061	9774	4493	4889	4624	uridine kinase
COG2820	826	1187	2091	6299	5236	5513	3088	5301	4442	3281	6346	3298	5947	6195	4699	986	1219	688	Uridine phosphorylase
COG2987	5957	6552	5521	7039	9016	8262	7323	8828	7742	6878	7135	7507	8299	6594	8995	6145	5968	5693	Urocanate hydratase
COG1904	4589	5157	3632	7121	9196	8603	7253	8951	7733	6496	7017	7633	8281	6790	9457	3977	4009	3660	Uronic isomerase
COG2959	1187	1383	2946	741	464	510	1820	1186	1502	1311	1519	1559	463	659	565	2372	1929	1909	uroporphyrin-III c-methyltransferase
COG5425	377	634	454	358	392	332	494	426	415	648	487	588	289	334	432	513	336	305	Usg family
COG4810	24	28	26	8	6	9	5	7	9	18	10	18	6	8	8	26	75	131	Utilization Protein
COG4816	37	39	33	9	8	13	8	8	18	19	11	23	8	10	10	32	85	134	Utilization Protein
COG1210	10870	10457	8130	14718	15274	15154	12015	15664	14008	11802	15058	12450	14961	13883	15055	9114	8865	9271	utp--glucose-1-phosphate uridylyltransferase
COG4294	1956	730	169	380	462	416	508	375	440	1090	513	557	425	530	471	798	1150	945	UV damage endonuclease
COG3880	1327	742	214	998	1217	1407	868	1035	993	1360	933	1194	1307	1156	1453	768	1027	980	Uvrb UvrC protein
COG1379	997	429	93	364	382	448	311	338	331	690	386	404	382	497	460	430	670	638	Uvrd rep helicase
COG5167	0	0	0	0	0	0	0	0	0	0	0	0	0	0	0	0	0	0	vacuolar import and degradation protein
COG5043	0	0	0	0	0	0	0	0	0	0	0	0	0	0	0	0	0	0	Vacuolar Protein
COG5158	0	0	0	0	0	0	0	0	0	0	0	0	0	0	0	0	0	0	Vacuolar Protein
COG5036	4	3	2	4	6	4	1	4	3	3	3	4	2	4	4	2	2	3	Vacuolar transporter chaperone
COG3977	269	405	1412	878	31	73	825	171	516	384	251	683	483	962	92	252	281	320	Valine--pyruvate aminotransferase
COG2720	2576	1298	428	1523	1613	1685	1412	1090	1237	1732	1056	1568	1545	1443	1705	1351	1820	1844	VanW family
COG4767	3392	3345	1667	523	574	617	399	322	449	703	398	469	568	596	632	2063	2116	2866	Vanz family
COG5652	3944	2704	781	7381	9186	8764	7495	9249	7841	6964	7720	7749	8494	7089	9338	2902	3118	2264	vanz family
COG4466	1635	528	102	31	33	35	47	28	47	653	47	51	22	27	29	506	923	857	Veg protein
COG4260	2152	1753	543	4586	6362	5330	5269	6173	5287	4535	4990	5323	5557	4072	6430	1908	2057	1334	virion core protein (Lumpy skin disease
COG5005	36	33	9	152	91	101	175	125	119	165	218	118	102	136	125	36	29	30	virion morphogenesis protein
COG2990	222	405	1410	55	6	18	70	34	22	96	144	52	14	39	14	244	231	311	VirK protein
COG4457	216	400	1406	4	1	2	12	6	5	29	10	15	2	2	0	237	223	304	virulence
COG4458	216	400	1406	4	1	2	12	6	5	29	10	15	2	2	0	237	223	304	virulence
COG3946	633	1169	1691	423	360	344	998	657	707	982	721	958	336	460	410	2204	1835	1787	virulence factor family protein
COG4455	468	596	1477	352	390	448	376	362	412	366	451	447	392	275	542	535	502	529	Virulence Protein SciE Type
COG4456	2113	1840	792	4916	5928	4879	5407	6199	5678	4284	4827	5289	5573	4658	5895	1609	1738	1011	Virulence-associated protein
COG3309	0	0	0	0	0	0	0	0	2	0	0	0	0	0	0	0	0	0	Virulence-associated protein D
COG5545	97	111	16	266	232	265	642	359	300	362	374	470	231	273	242	132	120	164	Virulence-associated protein e
COG4243	4275	4420	2002	10987	11478	10922	7610	11041	9582	7051	10646	7988	11417	10127	11088	3212	3207	3226	Vitamin K epoxide reductase
COG4245	1586	1004	223	2064	1347	1487	1914	1169	1984	1533	1206	2009	1535	2003	1476	740	1066	779	von Willebrand factor type A
COG4548	1451	1166	1502	798	636	801	774	976	813	1342	1107	1274	601	961	837	2345	2102	2023	von Willebrand factor type A
COG3552	1354	1168	497	7139	6859	7271	3455	6260	5189	4078	7354	3957	6930	6589	6277	1384	1000	1075	Vwa containing coxe family protein
COG3825	1078	1306	1694	1420	672	691	2090	1209	1848	1875	1726	1899	1081	1539	812	2479	1989	1905	Vwa containing coxe family protein
COG2425	4738	4334	3898	9373	11838	11563	8142	10978	9423	8078	8642	9138	10916	8903	12046	5270	5172	4412	VWA domain protein interacting with AAA ATPase
COG3864	245	171	68	321	409	399	229	278	327	292	291	371	276	354	381	149	197	224	VWA-like domain (DUF2201)
COG5608	456	1167	2771	92	72	65	494	256	271	454	311	285	91	84	84	3203	2655	2687	water Stress and Hypersensitive response
COG2319	11690	9862	3627	45498	33925	35567	33978	30512	35530	27925	29908	37390	43297	48122	35671	10363	11226	8570	wd repeat
COG3831	362	677	576	226	247	234	304	257	262	370	265	386	257	310	296	414	279	338	WGR domain-containing protein
COG4642	343	673	1423	354	222	261	757	371	374	582	340	695	222	262	294	1688	1418	1364	whole genome shotgun sequence
COG3373	0	0	0	0	0	0	0	0	0	0	0	0	0	0	0	0	0	0	Winged helix DNA-binding domain (DUF1495)
COG1975	8828	9896	10500	16164	17599	17544	14891	17509	16054	13666	17379	15009	16993	15096	17729	10507	9903	9101	xanthine dehydrogenase
COG4630	1077	1632	3157	545	506	469	1591	837	801	1373	1371	1087	623	521	521	2556	2133	2037	xanthine dehydrogenase
COG4631	1130	1746	3185	875	816	899	2377	1377	1272	1894	1887	1749	913	914	977	2702	3049	2172	xanthine dehydrogenase
COG2252	10455	10602	10449	9455	10879	10456	9802	10667	10354	9251	8935	10092	10424	9249	11200	10127	9696	10337	Xanthine uracil vitamin C permease
COG4317	1496	1677	4270	75	82	52	239	126	107	723	269	246	59	73	90	2442	2277	2321	XapX domain protein
COG2936	7566	6807	4381	16771	20494	19409	15194	18767	17338	13926	15095	16704	19065	16370	20863	6346	6821	4707	X-Pro dipeptidyl-peptidase C-terminal non-catalytic domain
COG2115	6420	7650	4988	7813	10052	9549	7742	9569	8418	6941	7611	8294	9095	7634	10339	5344	5133	5421	Xylose isomerase
COG1082	60675	69109	51351	86098	104164	104268	77754	97672	86926	73450	81364	85809	98393	85781	107633	56411	55011	53827	Xylose isomerase domain protein TIM barrel
COG4130	456	1106	2915	5	2	2	50	21	21	173	55	66	3	3	2	1875	1570	1580	Xylose isomerase Domain-Containing protein
COG4681	1020	1222	1743	830	652	733	1691	1055	1174	1447	1516	1449	668	615	861	2336	1926	1922	Yaeq family
COG2606	11798	14468	15188	16400	17998	18232	13766	17826	16259	12555	16955	13944	17159	15353	17719	12343	10907	12691	YbaK ebsC protein
COG4856	1229	608	154	877	990	1051	637	759	715	943	692	782	938	877	922	625	839	756	ybbr family
COG2353	15840	16365	12537	24142	29932	27937	28391	30392	27636	24949	26613	28427	27408	23722	30927	16170	16063	13610	YceI family
COG1724	1875	2119	3151	4600	3287	3885	3921	3579	4325	2902	3004	4122	4261	4609	3628	2918	2759	2555	ycfa family
COG3100	487	964	2829	102	39	31	178	142	227	231	127	255	32	57	41	1834	1551	1589	YcgL domain
COG2350	9809	11672	10929	8367	9316	8624	10454	10273	10713	8136	8736	10088	8806	8032	9508	8755	8173	9203	YciI-Like Protein
COG3795	13118	11055	5831	24763	31113	29531	24442	29584	27167	22244	24285	26366	28375	23295	32037	12185	12177	9239	ycii-related protein
COG3209	8985	9926	16312	13295	15816	15908	14411	15620	14505	13499	13812	15624	14926	13130	17344	12462	11713	11005	YD repeat protein
COG3141	386	920	2765	1	1	0	6	3	5	143	42	35	0	2	0	1791	1513	1572	YebG family
COG3318	1452	2153	4432	981	814	880	1913	1215	1266	1902	1814	1638	865	784	1015	4375	3605	3594	Yeca family
COG3102	203	366	1389	0	1	0	1	1	0	7	3	2	0	1	0	216	202	292	YecM protein
COG2391	3012	2982	2537	1677	1537	1454	3351	2566	2628	3564	2941	3871	1352	2181	1658	5229	4756	4082	YeeE YedE family protein
COG2731	669	1157	2835	503	369	521	1159	699	609	854	961	881	442	712	606	702	813	749	YhcH YjgK YiaL family protein
COG2964	2259	3499	5294	144	153	132	823	410	518	371	454	386	104	121	114	3188	3406	3563	YheO domain protein
COG3714	1552	1665	2946	249	249	209	645	443	580	979	487	616	205	292	271	2231	2344	2239	Yhhn family
COG3350	784	754	263	6284	5859	6272	2884	5629	4451	3398	6543	3306	6122	5592	5392	823	781	773	YHS domain protein
COG4298	210	311	242	791	140	138	479	204	558	466	490	580	578	986	166	309	217	137	YiaAB two helix domain-containing protein
COG4682	1185	619	520	142	101	104	853	318	303	693	475	454	111	164	158	629	803	644	YiaAB two helix domain-containing protein
COG5438	1571	2294	1205	268	312	355	169	167	259	203	194	222	359	348	333	1261	1181	2022	YibE F family protein
COG1561	5235	4792	4244	8671	10536	10042	9647	10854	9921	8988	9330	10148	9701	8391	11005	5757	5602	4465	YicC domain protein
COG2881	325	262	106	548	659	684	453	458	535	402	440	604	633	502	753	314	349	367	Yip1 domain
COG5197	0	0	0	0	0	0	0	0	0	0	0	0	0	0	0	0	0	0	yip1 interacting factor homolog
COG3403	886	352	109	71	81	67	85	82	89	390	73	116	63	80	78	313	556	549	Yqcl/YcgG family
COG4863	892	320	71	15	16	16	25	13	22	329	28	22	10	13	15	305	555	545	YqcH protein
COG4853	892	320	71	15	17	16	25	14	22	329	28	22	10	13	15	305	555	545	yycI protein
COG5427	416	333	119	918	1128	1152	584	703	750	557	668	790	1053	964	1083	326	303	240	YYY membrane protein
COG4185	647	1276	1534	642	428	537	1827	905	862	1252	1362	1316	475	645	586	2233	2091	1659	Zeta toxin
COG2173	1773	1838	1897	4004	3459	3872	4177	3534	3738	3025	3154	4042	3694	3988	3770	1816	1929	1522	zinc D-Ala-D-Ala carboxypeptidase activity
COG4357	739	225	32	9	15	12	27	26	36	317	18	40	9	11	11	195	372	311	zinc finger
COG5082	0	0	0	0	0	0	0	0	0	0	0	0	0	0	0	0	0	0	zinc finger
COG4855	0	0	0	4	8	4	1	2	3	0	2	2	2	4	6	0	0	0	Zinc finger protein
COG5236	9	3	2	1	2	1	2	2	5	2	3	5	1	1	1	2	2	1	zinc finger protein
COG5431	123	73	20	5111	4486	4888	1841	4543	3356	2200	5445	1879	4696	4420	3921	93	77	37	Zinc finger SWIM domain protein

COG4715	1289	615	192	1507	1087	1072	938	750	1166	1081	828	1135	1414	1738	1011	620	811	627	zinc finger SWIM domain-containing protein
COG1779	0	0	0	1	0	2	0	0	0	0	0	0	1	2	0	0	0	0	zinc ion binding
COG5243	168	106	28	5163	4537	4965	1997	4643	3481	2266	5581	1988	4760	4496	4038	119	102	71	zinc ion binding
COG2321	5738	6604	5671	7390	9338	8453	8681	9327	8669	7405	7932	8824	8107	6912	9221	6246	5922	5411	zinc metallopeptidase
COG3227	4289	4031	3638	1215	1401	1249	1956	1420	1738	2276	1886	2159	1271	1624	1439	2973	3053	3557	zinc metalloprotease
COG1913	2086	2758	1508	7491	7030	7941	3527	6952	5420	3795	7340	3829	7197	6803	6605	1790	1692	2315	zinc metalloprotease whose natural substrate is
COG4324	678	978	1553	512	446	480	913	581	672	1003	961	916	455	376	595	2154	1790	1683	zinc protease protein
COG0428	6650	6220	3555	14102	14468	14376	11516	14748	13615	10522	14310	11909	14441	13267	14220	6468	6031	5851	zinc transporter
COG4391	1138	867	469	1085	879	844	2303	1628	1909	1713	1916	2092	774	990	1000	949	668	593	Zinc-finger domain
COG5349	1067	1698	1158	1073	1120	996	1459	1272	1248	1889	1333	1791	879	1078	1245	1409	1064	1007	zinc-finger protein
COG5504	2423	796	196	287	360	357	253	246	301	1125	274	331	331	337	353	720	1441	1419	Zn-dependent protease
COG1326	32	15	8	18	30	35	15	16	22	14	11	17	25	19	26	10	9	11	Zn-finger protein-like protein
COG3364	0	0	0	0	0	0	0	0	0	0	0	0	0	0	0	0	0	0	Zn-ribbon containing protein (DUF2072)
COG1579	5105	5659	2693	13121	14705	14615	9550	14122	11713	9183	13021	10221	14192	12270	14638	4255	4222	3773	Zn-ribbon protein
COG2888	0	0	0	1	0	2	0	0	0	0	0	0	1	2	0	0	0	0	zn-ribbon RNA-binding protein
